# One-carbon homologation of alkenes

**DOI:** 10.1038/s41586-025-09159-9

**Published:** 2025-05-20

**Authors:** Marcus C. Grocott, Matthew J. Gaunt

**Affiliations:** Yusuf Hamied Department of Chemistry, University of Cambridge, Cambridge, UK

**Keywords:** Synthetic chemistry methodology, Natural product synthesis

## Abstract

One-carbon homologues are structurally related and functionally identical organic molecules whose chain lengths differ by a single methylene (–CH_2_–) unit^1^. Across many classes of molecule—including pharmaceutical agents, natural products, agrochemicals, fragrances and petroleum products—the physicochemical characteristics exhibited by members of a homologous series subtly differ from one compound to another, which can impart remarkable differences to their function^2^. The efficient generation of homologues is, therefore, an important strategy in molecular discovery programmes^3,4^. Despite the availability of homologation strategies for several functional groups^5,6^, direct and general methods for one-carbon chain extension in alkenes remain an unmet synthetic need^7,8^. Here we report a catalytic one-carbon homologation process that is effective for many classes of alkene in simple and complex molecules. By leveraging the intrinsic reactivity of a new multifaceted allylsulfone reagent, a streamlined one-pot process, involving cross-metathesis and a fragmentation–retro-ene cascade, formally inserts a single methylene unit into the alkene chain. Among the applications of this process to several structurally and functionally complex molecules, we demonstrate how this practical transformation generates previously unexplored homologues of cyclosporine A^9^. These homologues exhibit modulated pharmacological and biological properties and could provide promising leads as cyclophilin inhibitors, a target that has great potential in many disease areas^10^.

## Abstract (web summary)

A new method for extending alkenes by one or two carbons is shown to be useful in attenuating the immunosuppressive properties of a medicine, making it potentially applicable for targeting disease areas where immunosuppresion is unwanted.

## Main

**Fig. 1:**
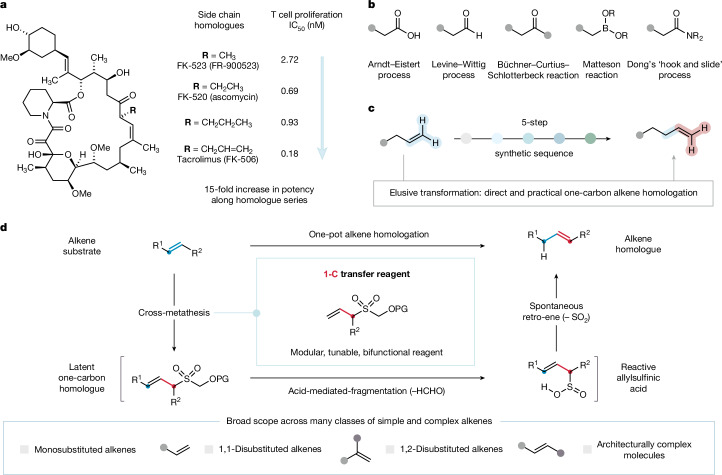
Evolution of a strategy for one-carbon alkene homologation. **a**, The effect of homologues on biological activity: side chain length compared with immunosuppressive activity in a family of natural products. **b**, Homologation processes for common functional groups. **c**, Generally adopted sequence for alkene homologation. **d**, Design plan for a catalytic one-carbon alkene homologation process.

Nature embraces one-carbon homologues to modulate biological function. For example, several proteinogenic amino acids differ by a single carbon atom and fulfil distinct roles in the cellular machinery. Many natural products also exist as a series of homologues and often exhibit comparatively different biological activities (Fig. 1a). As an exemplar of this phenomena, tacrolimus (FK-506) exhibits potent immunosuppressant activity, which can be attributed to the length of its hydrocarbon side chain in relation to those of its homologues FK-523 and FK-520 (ref. ^4^). Nature, however, does not generally use its cellular machinery to incrementally extend structures one carbon atom at a time, and instead harnesses the available building blocks with the appropriate carbon content to feed biosynthetic pathways, constructing homologues in a de novo fashion. Conversely, chemical strategies for one-carbon homologation are frequently deployed during synthesis campaigns when restrictions in the available feedstock pool require the addition of further carbon atoms to advance assembly of the target molecule^11,12^. From a strategic perspective, iterative homologation strategies could be pivotal in unravelling the mode of action or optimization of bioactive molecules directly from a lead candidate or advanced intermediate^3,4^. This approach, however, is rarely adopted in discovery programmes because practical and effective one-carbon homologation processes are restricted to a narrow range of commonly used functional groups^5,6,13–18^ (Fig. 1b). Therefore, extending the chain length of functional hydrocarbons usually requires multi-step syntheses. Although these processes can be executed on small molecules, they are often incompatible or synthetically intractable with more complex structures.

There is no straightforward and broadly applicable method for the one-carbon homologation of alkenes, a cornerstone functional group in organic chemistry^7,8^ (Fig. 1c). Instead, chain extension of alkenes usually requires oxidative cleavage of the carbon–carbon double-bond, Levine–Wittig reaction to install an enol-ether, acid-mediated hydrolysis and, finally, Wittig methenylation to install the additional carbon atom^4^. Notably, this stepwise process requires functional group interconversion of alkene to aldehyde, thereby exploiting an available homologation method^14,19,20^. Although this type of sequence is practised frequently, the minimum four- or five-step process is cumbersome and often incompatible with oxidatively sensitive functionality^4,21^. Therefore, a mild method for one-carbon homologation directly from the alkene would represent a powerful synthetic transformation and have immediate application in areas ranging from medicinal chemistry to feedstock upgrading.

At the core of our alkene-homologation strategy is the design of a versatile one-carbon transfer reagent (1CTR) based on an allylsulfone framework (Fig. 1d). As part of a one-pot transformation, the carbon–carbon double-bond of the 1CTR can be coupled to a substrate alkene by an olefin cross-metathesis reaction^22,23^, forming a 1,2-disubstituted allylsulfone (latent homologue). In the case of a terminal alkene, the intrinsic loss of ethylene during cross-metathesis results in the net incorporation of a single carbon atom into the latent homologue (denoted by a red dot). To avoid uncontrollable alkene elongation, a two-stage homologation process was deemed essential. Following completion of the metathesis reaction, the second stage of the homologation process can be initiated by in situ cleavage of the oxygen-protecting group and a fragmentation to eliminate formaldehyde^24^. The resulting allylsulfinic acid is primed to undergo a spontaneous retro-ene reaction^25^, migrating the alkene from its internal position in the latent homologue to the chain-extended terminal alkene product.

**Fig. 2:**
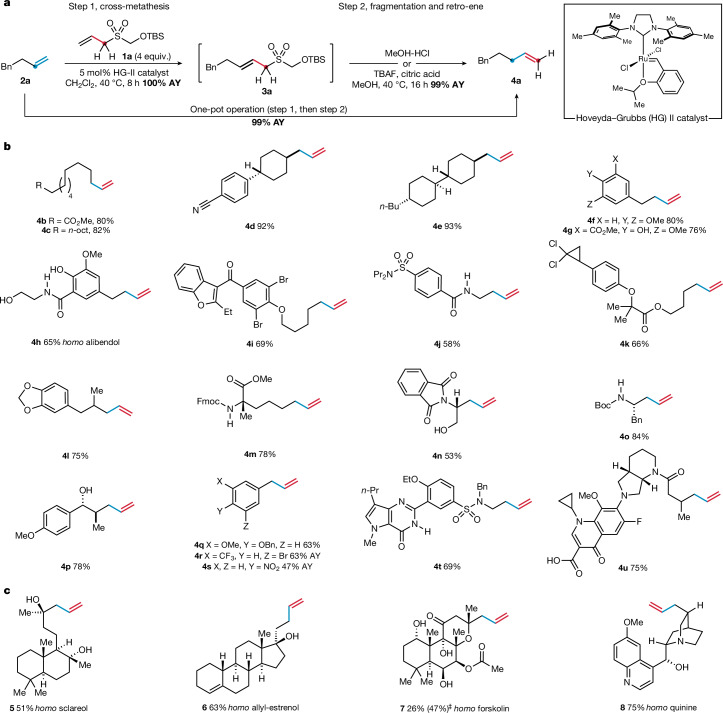
Development of a one-carbon alkene-homologation process. **a**, Optimized conditions for the one-pot alkene homologation process. **b**, Scope of monosubstituted alkenes. **c**, Natural products and bioactive molecules. AY, assay yield (^1^H NMR); ^‡^based on recovered starting material.

A *tert*-butyl dimethylsilyl (TBS) protecting group was selected to mask the α-hydroxysulfone motif of the 1CTR. The TBS group can be cleaved under a variety of mildly acidic conditions, simultaneously protonating the incipient allylsulfinate to promote the retro-ene reaction^24^. The 1CTR (**1a**) was synthesized on a multi-gram scale from the commodity chemical rongalite. Various catalysts, solvents and reagent stoichiometries were evaluated for the cross-metathesis of alkene **2a** and 1CTR **1a** (Supplementary Information). The optimal conditions were found to involve 5 mol% of the Hoveyda–Grubbs second-generation (HG-II) catalyst^26,27^ and 4 equivalents (equiv.) of 1CTR **1a**, which delivered a quantitative assay yield of the latent homologue **3a** (Fig. 2a). After assessment of several conditions for the acid-mediated desilylation–fragmentation–retro-ene cascade, treatment of **3a** with methanolic HCl solution most effectively triggered the formation of alkene homologue **4a**. Notably, this deprotection-driven cascade can be coupled to the cross-metathesis reaction, forging a one-pot homologation process. Accordingly, stirring a dichloromethane solution of alkene **2a**, 4 equiv. of 1CTR **1a** and 5 mol% HG-II catalyst at 40 °C for 8 h, followed by the addition of a 2 M solution of methanolic HCl and stirring for a further 16 h, yielded the homologated alkene **4a** in near-quantitative assay yield.

The scope of the one-carbon homologation process was evaluated using an array of alkenes, containing structural and functional groups commonly encountered in everyday chemical synthesis applications (Fig. 2b). In most cases, the standard homologation conditions proved effective. However, for alkenes containing acid-sensitive groups, such as esters (**4k**) and carbamates (**4o**), a milder retro-ene protocol consisting of tetrabutylammonium fluoride (TBAF) and citric acid was used. Notable examples include the formation of alkene **4p**, which exemplifies the ability for the homologation process to access an organic scaffold that would otherwise require a bespoke multi-step synthesis^21^. Styrenes were also amenable to the homologation process and underwent efficient deconjugation using modified retro-ene conditions (90 °C, TFA-H_2_O), yielding allylbenzenes **4q**–**s**. Alkenes bearing polar functionalities, including amine-containing heterocycles (**4t**) and carboxylic acids (**4u**), underwent the one-pot homologation process in good yields, validating the broad functional group compatibility of the methodology. In cases in which cross-metathesis did not reach completion, resulting in inseparable traces of starting materials, purification of the more polar latent-homologue intermediate before the retro-ene reaction enabled clean isolation of the desired homologation products (Supplementary Information). Several alkene-containing natural products, including the terpenes sclareol and forskolin, as well as the steroid derivative allylestrenol, underwent successful homologation (Fig. 2c; **5**–**7**). The alkaloid quinine could also be converted to homoquinine (**8**) when masked as its protonated ammonium salt. To the best of our knowledge, the homologues of these bioactive molecules have not been previously reported.

**Fig. 3:**
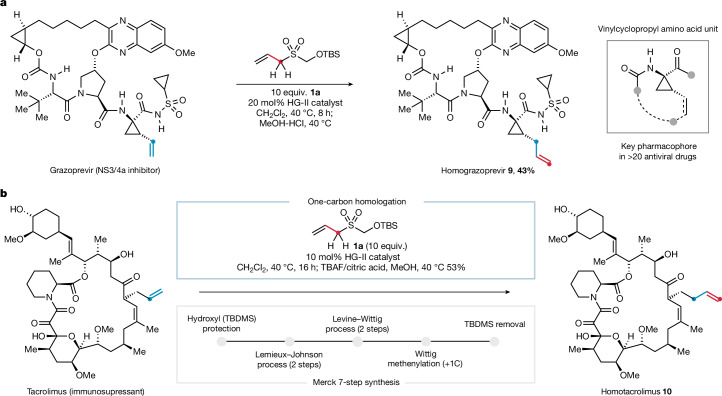
One-carbon alkene homologation of architecturally complex molecules. **a**, One-carbon homologation of the vinyl ACCA motif, common to many antiviral drugs. **b**, Streamlined one-carbon homologation of the alkene side chain in tacrolimus and its comparison to a stepwise synthesis.

Although several compounds in Fig. 2c exhibit drug-like characteristics, we sought to demonstrate the wider compatibility of the homologation methodology through its deployment on complex alkene-containing pharmaceuticals, which often contain multiple polar and sensitive functionalities. Grazoprevir, a selective inhibitor of hepatitis C virus NS3/4a protease, presents several polar functionalities alongside its 1-amino-2-vinylcyclopropane carboxamide (ACCA) motif—a privileged pharmacophore found in more than 20 antiviral drugs^28–30^. The one-carbon homologation protocol transformed the ACCA unit of grazoprevir into an allylcyclopropane analogue (**9**) in 43% isolated yield (Fig. 3a), a structural motif that would be difficult to obtain through conventional synthetic methods. The alkene within the ACCA pharmacophore has been exploited as a handle for macrocyclization using ring-closing metathesis (RCM), a key strategy in the synthesis of several of these macrocyclic drugs^30^. Several pharmaceutical agents also feature ethyl-ACCA motifs, whose homologues can be readily obtained through a subsequent hydrogenation of the chain-extended alkene. This homologation strategy, therefore, could provide a rapid and efficient means to access ring-expanded or homologated variants of these antiviral candidates, directly from the ACCA motif.

A further demonstration of the efficacy of the one-carbon homologation is illustrated by its application to tacrolimus, an essential medicine of the World Health Organization (WHO) and an important degrader tool compound^31,32^. Several side chain analogues of the tacrolimus macrolide have been synthesized as part of extensive structure–activity relationship studies, and the one-carbon homologue of tacrolimus (**10)** required a seven-step synthesis from its parent olefin^4^. Through the implementation of our one-carbon homologation process, the transformation of tacrolimus into **10** could be achieved in 53% isolated yield, demonstrating how this new strategy can markedly streamline the synthesis of closely related analogues in complex settings (Fig. 3b).

**Fig. 4:**
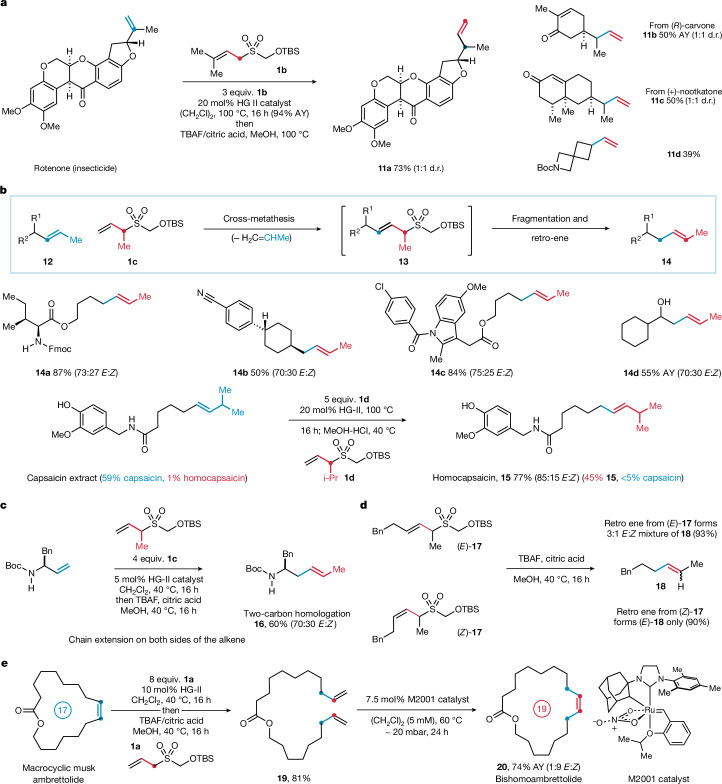
Homologation of disubstituted alkenes. **a**, Chain extension of 1,1-disubstituted alkenes with 1CTR **1b** and scope. **b**, Homologation of 1,2-disubstituted alkenes and application to feedstock upgrading. **c**, Two-carbon homologation of terminal alkenes: introduction of carbons into the alkene chain and at double-bond terminus. **d**, Experiments to explore the *E*/*Z* selectivity of the retro-ene process. **e**, Ring expansion of macrocycles. AY, assay yield (^1^H NMR).

Next, we questioned whether the homologation methodology could be applied to disubstituted alkenes (Fig. 4). Initial attempts at cross-metathesis between a 1,1-disubstituted alkene (rotenone) and 1CTR **1a** resulted in low conversion to the desired trisubstituted allylsulfone intermediate (Supplementary Information). Notably, 1CTR **1a** exhibited near-quantitative conversion to its corresponding homodimer, implying a mismatch in cross-metathesis selectivity^27^. After extensive investigation of the parameters of the reaction, the introduction of geminal methyl substituents to the alkene of the 1CTR (to form the type-III alkene, **1b**) facilitated cross-metathesis of rotenone to its latent homologue in excellent yield (94%). The subsequent retro-ene reaction delivered the chain-extended, α-branched alkene **11a** in 73% isolated yield from rotenone (Fig. 4a). Through the metathesis–retro-ene process, several 1,1-disubstituted alkenes could be converted to the corresponding α-branched products (**11a**–**d**) using 1CTR **1b**. These homologation products represent new analogues of natural products that are used as active ingredients in agrochemicals, fragrances and flavourings, as well as being valuable new building blocks.

The modular nature of the 1CTR allows carbon substituents to be incorporated at the allylic position (Fig. 4b). We predicted that a 1,2-disubstituted alkene (**12**) (terminated with a methyl group) would undergo cross-metathesis with methyl-substituted 1CTR **1c** to form latent homologue **13**. The subsequent retro-ene reaction would then evoke transposition of the double bond along the carbon chain by one atom, yielding the one-carbon homologated 1,2-disubstituted alkene (**14**); no direct method for the one-carbon homologation of internal alkenes has been reported, to the best of our knowledge. The cross-metathesis between α-Me-1CTR **1c** and an amino acid-derived alkene successfully yielded the latent homologue, which, on treatment with TBAF/citric acid, underwent the retro-ene reaction to deliver the homologated internal alkene **14a** in 87% yield (approximately 3:1 *E*/*Z*). The internal alkene homologation process was applicable to several simple 1,2-disubstituted alkenes and generated the chain-extended products in good yields (**14a**–**d**). Using the α-iPr variant of the 1CTR (**1d**), we applied the homologation process to natural capsaicin extract (containing 58.8% capsaicin and 1% homocapsaicin), which enriched the extract to 45% homocapsaicin (**15**, 5:1 *E*/*Z*) and <5% capsaicin. Homocapsaicin is a valuable 1,2-disubstituted-alkene (seven steps from an expensive starting material^33^) that possesses a substantially different Scoville heat unit value to capsaicin. Several capsaicin analogues are also now under investigation for oncological and analgesic applications^34^. This transformation highlights the potential for the direct valorization of abundant feedstocks into higher-value or distinct olefin products. It was also possible to affect a two-carbon chain extension of a terminal alkene using 1CTR **1c** in good yield (Fig. 4c). Here, the metathesis–retro-ene process not only extends the alkene chain by one carbon, but a second carbon is added to the double bond, resulting in the conversion of a terminal alkene to a 1,2-disubstituted alkene containing two addition carbon atoms (**16**).

The geometry of the homologated internal alkenes (**14**) generated by the retro-ene reaction was not retained with respect to the *E*/*Z* ratio in the allylsulfone intermediates (**13**). This observation is in general agreement with previous reports of the retro-ene reaction of allylsulfinic acids^35,36^, which also demonstrated that increasing the size of the allylic substituent favours preferential formation of the *E*-isomer. To further probe this observation, we prepared both geometric isomers of the latent homologue **17** by de novo routes. When (*E*)-**17** was exposed to the standard fragmentation–retro-ene conditions, we observed the homologated alkene product **18** in a 3:1 mixture of *E* and *Z* isomers (Fig. 4d). However, when (*Z*)-**17** was reacted under the same conditions, exclusive conversion to the *E*-isomer of **18** was observed. The origin of this unexpected observation remains unclear. However, the differing reactivity of the two geometric isomers is proposed to arise from destabilizing 1,3-diaxial interactions that affect the energies of the corresponding retro-ene transition states (Supplementary Information).

The synthetic use of the homologation process was further demonstrated by its use for macrocyclic alkene ring expansion. Ambrettolide, a 17-membered macrocyclic lactone, belongs to a valuable class of alkene-containing musks that are widely used in the fragrance industry^37^. Application of the two-step homologation process to ambrettolide yielded intermediate diene **19** by a ring-opening cross-metathesis sequence using 1CTR **1a**. Subsequent RCM of **19** yielded the *Z*-isomer of the 19-membered macrocycle **20** in excellent yield, using the M2001 catalyst^38^. Alternatively, RCM could be performed using UltraNitroCat^39^, which led to the preferential formation of the *E*-isomer of **20** (Supplementary Information). This two-carbon macrocycle expansion strategy constitutes a straightforward method to access higher-order ring-homologues, which may exhibit subtle differences in conformational properties.

**Fig. 5:**
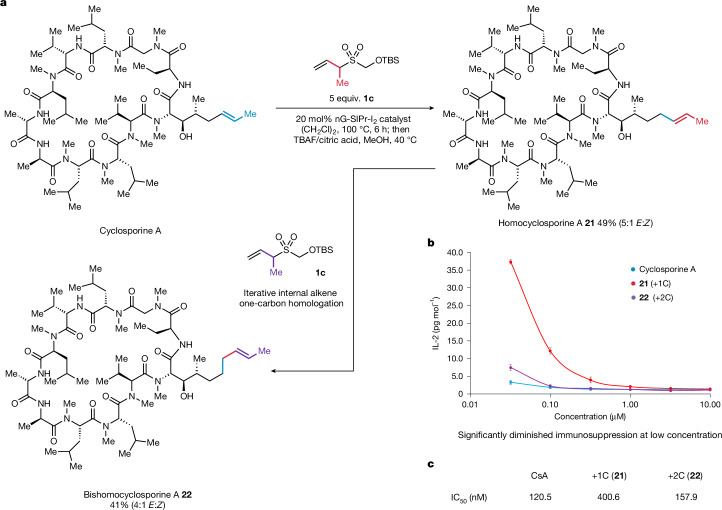
Homologation of cyclosporine A and biological evaluation of its corresponding homologues. **a**, Iterative one-carbon homologation of cyclosporine A (CsA). **b**, IL-2 release from Jurkat T cells for CsA, **21** and **22** (center values represent the means of three replicates; error bars represent s.e.m.). **c**, Biochemical assay for calcineurin inhibition for CsA, **21** and **22** (assays were performed in duplicate, with IC_50_ values calculated from mean averages).

Finally, we examined the application of the one-carbon homologation reaction to cyclosporine A, a complex macrocyclic peptide that is a potent immunosuppressant and an essential medicine of the WHO (Fig. 5). Cyclosporine A exerts its biological activity through binding to the immunophilin protein cyclophilin A (refs. ^40,41^). The resulting binary complex has an affinity for the protein phosphatase calcineurin, forming a ternary assembly that inhibits interleukin-2 (IL-2) transcription and thereby preventing T cell activation^42^. In the cyclophilin-bound conformation of cyclosporine A, its alkene side chain projects outwards from the binary complex, presenting itself and other key residues for interaction with the calcineurin-binding domain^43^. Programmable synthetic extension of the alkene side chain in cyclosporine A could yield analogues that can fine-tune interactions between the binary complex and calcineurin, thereby offering a means to modulate immunosuppressive potency. Through the application of the new internal alkene homologation strategy, the alkene-containing side chain of cyclosporine A was extended by one carbon atom using 1CTR **1c** under slightly modified conditions (Fig. 5a), generating a new homologue (**21**) in reasonable yield. Moreover, the process could be iterated on **21** to deliver the two-carbon homologue (**22**). In both cases, good selectivity for the *E*-alkene product was obtained, and the geometric isomers were readily separable by high-performance liquid chromatography.

To assess the biological significance of one-carbon homologation on cyclosporine A, we evaluated the immunosuppressive potency of homologues **21** and **22** by measuring IL-2 release from Jurkat T cells^43^ (Fig. 5b and Supplementary Information). At high concentrations (≥1 µM), cyclosporine A, **21** and **22** effectively suppressed IL-2 secretion. However, differences in immunosuppressive potency became apparent at lower concentrations (≤320 nM) for homologue **21**. Notably, at 32 nM, **21** exhibited a 13-fold reduction in immunosuppressive potency relative to cyclosporine A. To investigate whether the relative reduction in IL-2 release for homologue **21** was a consequence of altered calcineurin inhibition, we conducted in vitro calcineurin phosphatase activity assays^44^ (Fig. 5c). The one-carbon homologue (**21**) exhibited a 3.5-fold decrease in phosphatase activity compared with cyclosporine A, whereas the two-carbon homologue (**22**) retained comparable potency to cyclosporine A.

**Fig. 6:**
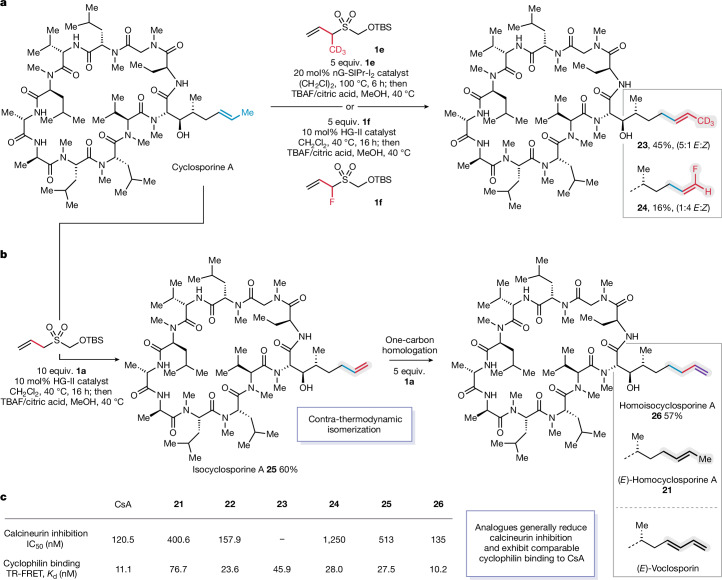
Synthesis of new cyclosporine A analogues and their biological evaluation. **a**, Exploiting the modularity of the 1CTR for side chain functionalization. **b**, Contra-thermodynamic isomerization of cyclosporine A (CsA) and its homologation to homoisocyclosporine. **c**, Biochemical assays for calcineurin inhibition and cyclophilin binding for compounds **21**–**26 **(assays were performed in duplicate, with IC_50_ and *K*_d_ values calculated from mean averages).

Inspired by the distinctive change in immunosuppressive activity brought about by the side chain homologue (**21**), we questioned whether other subtle changes to the alkene could impart further enhancement of biological properties. The modularity of the 1CTR platform enables facile incorporation of allylic substituents, allowing the corresponding homologation products to either retain the substituent of the parent alkene (as in **21** and **22**) or be programmed to introduce new functionality. To evaluate this approach, we synthesized 1CTRs bearing allylic CD_3_ and F substituents (**1e** and **1f**) and subjected these reagents to the metathesis–retro-ene sequence with cyclosporine A (Fig. 6a). Reaction of the CD_3_-substituted 1CTR **1e** with cyclosporine A afforded deuteromethyl homologue **23** in reasonable yield, as a 5:1 (*E*/*Z*) mixture of isomers. Although formally a homologation, this transformation not only extended the side chain but also replaced the methyl group with a CD_3_ substituent. By contrast, when the F-substituted 1CTR **1f** was deployed in the reaction with cyclosporine A, fluorinated isomer **24** was produced in modest yield, with selectivity for the *Z*-isomer 1:4 (*E*/*Z*). In this case, the carbon content is preserved with respect to the parent molecule, whereas the position of the double bond is transposed, incorporating a new fluorine substituent at the alkene terminus. We also investigated the reaction of the original, unsubstituted 1CTR **1a** in combination with the 1,2-disubstituted alkene of cyclosporine A. Treatment of cyclosporine A with 1CTR **1a**, followed by the retro-ene process, delivered isocyclosporine A (**25**) in good yield (Fig. 6b). Although the use of 1CTR **1a** in this context generates a new cyclosporine A analogue, it also represents a new strategy for the contra-thermodynamic isomerization of non-activated 1,2-disubstituted alkenes—a transformation that has broad use in organic synthesis^45–47^. Following the successful synthesis of isocyclosporine A (**25**), subsequent subjection of this material to terminal alkene homologation conditions with 1CTR **1a** afforded homoisocyclosporine A (**26**) in good yield.

With straightforward access to several cyclosporine A analogues, we further evaluated the calcineurin inhibition abilities of **21**–**26** (Fig. 6c). The fluorinated cyclosporine A analogue **24** exhibited a substantial (10-fold) decrease in phosphatase activity compared with cyclosporine A. Notably, the terminal alkene analogue isocyclosporine A (**25**) exhibited an approximate 4-fold reduction in phosphatase activity, whereas further homologation to **26** restored calcineurin inhibition to levels comparable with those of cyclosporine A. Notably, homoisocyclosporine A (**26**) constitutes a dihydro analogue of a voclosporin^48^, a recently approved calcineurin inhibitor bearing a terminal 1,3-diene of equivalent chain length. Collectively, the activity profiles for alkene analogues **22**–**26** underscore the delicate interplay of molecular structure within the ternary molecular glue complex, in which even subtle modifications such as chain length, alkene position and single-atom substitution can profoundly influence interactions between the binary complex and calcineurin.

To confirm that our cyclosporine A analogues retained affinity for cyclophilin A, we performed a TR-FRET assay to evaluate their ability to displace a fluorescently labelled cyclosporine A ligand^49^. Analogues **22**–**26** exhibited competitive binding to cyclophilin A (<80 nM). Although the TR-FRET assay does not directly assess the ability of these homologues to inhibit the enzymatic activity of cyclophilin A, their binding affinities are commensurate with other pre-clinical cyclophilin A inhibitors assessed using this type of assay^49^. Cyclophilin inhibitors that attenuate the phosphatase activity of calcineurin have recently emerged as a promising class of therapeutics for viral infections, cancer and inflammatory disorders, offering efficacy without the complications associated with immunosuppression^10^. Although several cyclosporine A analogues have demonstrated non-immunosuppressive properties^50–53^, these molecules typically require modifications to amino acid residues on the macrocyclic peptide, necessitating total syntheses or cumbersome multi-step routes for their preparation. The reduced immunosuppressive potency for homologues **21**, **24** and **25**, compared with cyclosporine A, coupled with their straightforward modular synthesis, could provide a new strategy for designing cyclosporine-derived cyclophilin inhibitors.

Subtle, systematic structural modifications brought about by one-carbon homologation can rarely be explored in complex molecules because of the absence of mild, practical and selective synthetic methods. Consequently, valuable structure–activity relationships and biological advances often remain unexplored and overlooked. The alkene homologation strategy described here provides a streamlined and versatile method for accessing previously unexplored alkene homologues, including derivatives of cyclosporine A, in which the functional nature and length of the alkene side chain can be systematically programmed. This approach holds promise for extension to other calcineurin inhibitors, such as homotacrolimus (**10**; Fig. 3b), which shows diminished immunosuppressive activity in vitro^4,54^. We anticipate that this catalytic alkene-homologation method, and complementary isomerization strategies, will serve as a valuable expansion of the synthetic toolkit for constructing structurally tailored complex molecules, with broad use in both academic and industrial institutions.

## Online content

Any methods, additional references, Nature Portfolio reporting summaries, source data, extended data, supplementary information, acknowledgements, peer review information; details of author contributions and competing interests; and statements of data and code availability are available at 10.1038/s41586-025-09159-9.

## Supplementary information

Supplementary Information This file contains Supplementary Sections 1–8, including Supplementary Tables 1–11, Supplementary Figs. 1–7, NMR Spectra Data and Supplementary References; see the Contents page for details.

## Data Availability

All data are available in the paper or the Supplementary Information.
