# Supplementary material for: One-carbon homologation of alkenes
Source: Nature. 2025 May 20;643(8070):130–8. doi: 10.1038/s41586-025-09159-9 (PMC12221989; doi:10.1038/s41586-025-09159-9)
Supplement: Supplementary file 1 — This file contains Supplementary Sections 1–8, including Supplementary Tables 1–11, Supplementary Figs. 1–7, NMR Spectra Data and Supplementary References; see the Contents page for details. [file 41586_2025_9159_MOESM1_ESM.pdf]

nature portfolio

https://doi.org/10.1038/s41586-025-09159-9

Supplementary information

One-carbon homologation of alkenes

In the format provided by the authors and unedited

Supplementary Information for

One-carbon homologation of alkenes

Marcus C Grocott and Matthew J Gaunt*

Correspondence to: mjg32@cam.ac.uk

Summary of Contents

1. Materials and methods 1
2. General Considerations 2
3. Reaction Optimization & Procedures 3
   3.1. Monosubstituted alkene optimisation studies 3
   3.2. General procedure for the one-carbon homologation of monosubstituted alkenes 6
   3.3. 1,1-disubstituted alkene optimisation studies 7
   3.4. General procedure for the one-carbon homologation of 1,1-disubstitutued alkenes 8
   3.5. General procedure for the one-carbon homologation of methyl terminated 1,2-disubstituted alkenes 9
   3.6. Alkene selectivity experiments 10
4. Synthesis of 1-C Transfer Reagents (1CTR's) 11
5. Substrate Scope 17
6. Biochemical assays 63
7. NMR Spectra 72
8. References 126

1. Materials and methods

All reactions were run under an inert atmosphere (N_2) unless otherwise stated, with oven-dried glassware, using Schlenk standard techniques. Anhydrous EtOAc, DCM, DCE, DMC, PhMe, 2-MeTHF, and MeOH were purchased from Sigma Aldrich and transferred via syringe using standard techniques unless otherwise stated. All commercial reagents were used as supplied unless otherwise stated. Petroleum ether 40° - 60° was used in all cases where the PE abbreviation is used.

One-carbon transfer reagents (1CTR's) were stored in the freezer under N_2 and were stable for over 6 months.

Analytical thin-layer chromatography (TLC) was performed on Merck Kieselgel 60 F254 0.20 mm precoated, glass backed silica gel plates. Visualisation of the developed chromatogram was performed by UV absorbance (λ_{max} = 254 nm), and/or by aqueous $KMnO_4$. Flash column chromatography was performed using silica gel (Merck Geduran Si 60 [40–63 μm]) with the indicated solvent system, silica gel impregnated with ~10wt% $AgNO_3$ with the indicated solvent system or performed on a Teledyne ISCO CombiFlash nextgen 300+ using Redisep silica or Redisep gold silica high performance cartridges, unless stated. Reverse phase column chromatography was performed on a Teledyne ISCO CombiFlash nextgen using C18 Redisep RF gold cartridges.

Nuclear magnetic resonance (NMR) spectra were recorded on a Bruker AM 400 (400 MHz) or Avance 500 (500 MHz) spectrometer or Avance 700 (700 MHz). Chemical shifts (δ) for ^1H NMR spectra are recorded in ppm from Me_4Si with the solvent resonance as the internal standard ($CDCl_3$ = 7.26 ppm, DMSO-*d*6 = 2.50, C6D6 = 7.16 ppm, CD3OD = 3.31 ppm). Data is reported as follows: chemical shift [integration, multiplicity (s = singlet, d = doublet, t = triplet, q = quartet, quint = quintet, sext = sextet, sept = septet, m = multiplet, br = broad), coupling constant and proton count]. ^{13}C NMR spectra are reported in ppm from Me_4Si with the solvent resonance as the internal standard ($CDCl_3$ = 77.16 ppm, DMSO-d_6 = 39.52, C_6D_6 = 128.06 ppm, CD_3OD = 49.00 ppm).

Low resolution mass spectrometry was performed on a Shimadzu LCMS-2020 with an eluent of 5−95% of C in D over 3.5 min at 0.5 mL/min on a Kinetex C18 LC column (100 Å, 2.6 μm, 2.1×50 mm), where C is a 95:5 mixture of MeCN:H_2O with 0.1% formic acid, and D is a mixture of H_2O with 0.1% formic acid and 0.05% ammonium formate. High-resolution mass spectra (HRMS) were measured on a Micromass Q-TOF or a Shimadzu QTOF LCMS-9030 spectrometer using ESI (electrospray ionisation) techniques at the Department of Chemistry, University of Cambridge.

Optical rotations were measured on an Anton Paar Polarimeter (MCP 100) at 20 °C, λ=589 nm. Infrared (IR) spectra were recorded on a Perkin Elmer FT-IR Spectrometer fitted with an ATR sampling accessory as solids or films, either through direct application or deposited in $CHCl_3$, with absorptions reported in wavenumbers (cm^{-1}).

2. General Considerations

The cross-metathesis reaction is sensitive to the type of reaction vessel used. Lower yields (5-15%) were observed using sealed microwave vessels, which can be attributed to the build-up of ethylene and accelerated catalyst decomposition. Optimal results can be obtained using a round bottom flask fitted to a reflux condenser, with Teflon tape around the ground glass joint. The reflux condenser should be maintained under an N_2 atmosphere, with an additional outlet needle to help purge ethylene from the reaction vessel.

1C homologs were generally found to be inseparable from their starting olefins by normal phase silica gel chromatography (if cross-metathesis did not reach full completion). If the starting olefin and 1C homolog were found to be separable by LC-MS (C18) 5-95% H_2O-MeCN (5 min), one-carbon homologs were found to be separable by reverse phase chromatography using a Teledyne ISCO CombiFlash nextgen fitted with C18 Redisep RF gold cartridge.

If purification of the cross-metathesis reaction is required and the 1CTR/1CTR homodimer coelutes with the latent homolog, equivalents of HCl-MeOH or Citric acid-TBAF used for the retro-ene reaction should be scaled relative to total equivalents of 1CTR reagent used in the cross-metathesis reaction.

Electron rich tertiary amines were generally found to be incompatible with the cross-metathesis reaction due to coordination and decomposition of the ruthenium catalyst. In these cases, temporary protection of the amine-containing substrate as its quaternary ammonium salt (hydrochloride or tosylate) was found to be effective.

3. Reaction Optimization & Procedures

3.1. Monosubstituted alkene optimization studies

A 5 mL round-bottom flask was charged with a magnetic stirrer bar, 1CTR **1a** (*n* equiv) and 4-phenyl-1-butene (15 µL, 0.1 mmol, 1.00 equiv). The round-bottom flask was fitted to a reflux condenser (Teflon tape around the ground glass joint, suba-seal on condenser outlet, connected to a N_2 line with a needle through the suba-seal) and heated to 40 °C in an oil bath. Solvent (0.25 mL) was added through the top of the condenser to dissolve the starting materials. A stock solution of catalyst (*n* mg/mL) was prepared in solvent and 0.5 mL of the stock solution was added through the suba-seal, followed by additional solvent (0.25 mL). The reaction was heated for 8 h. The solvent was removed *in vacuo* and conversion was measured by ^{1}H NMR using methyl benzoate (12.6 µL, 0.1 mmol, 1.00 equiv) as an internal standard.

Table S1. Screening equivalents of 1CTR **1a**

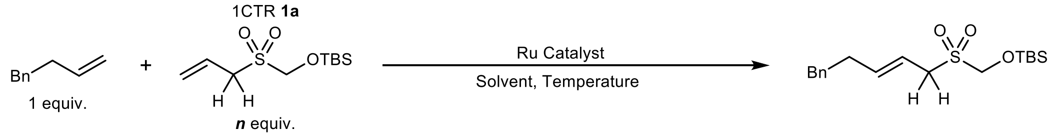

Entry	Solvent	Concentration	Catalyst	Temperature	1a equiv	Yield$^{\perp}$
1	DCM	0.1 M	HG-II (5 mol%)	40 °C	1	66%
2	DCM	0.1 M	HG-II (5 mol%)	40 °C	2	89%
3	DCM	0.1 M	HG-II (5 mol%)	40 °C	3	95%
4	DCM	0.1 M	HG-II (5 mol%)	40 °C	4	>99%

0.1 mmol scale alkene substrate, $^{\perp}$ ^{1}H NMR yields relative to methyl benzoate internal standard

Table S2. Solvent screen using 1CTR **1a**

Entry	Solvent	Concentration	Catalyst	Temperature	1a equiv	Yield$^{\perp}$
1	DCM	0.1 M	HG-II (5 mol%)	40 °C	4	>99%
2	DCE	0.1 M	HG-II (5 mol%)	40 °C	4	85%
3	PhMe	0.1 M	HG-II (5 mol%)	40 °C	4	80%
4	EtOAc	0.1 M	HG-II (5 mol%)	40 °C	4	79%
5	DMC	0.1 M	HG-II (5 mol%)	40 °C	4	83%
6	2-MeTHF	0.1 M	HG-II (5 mol%)	40 °C	4	67%

0.1 mmol scale alkene substrate, $^{\perp}$ ^{1}H NMR yields relative to methyl benzoate internal standard

Table S3. Catalyst screen using 1CTR **1a**

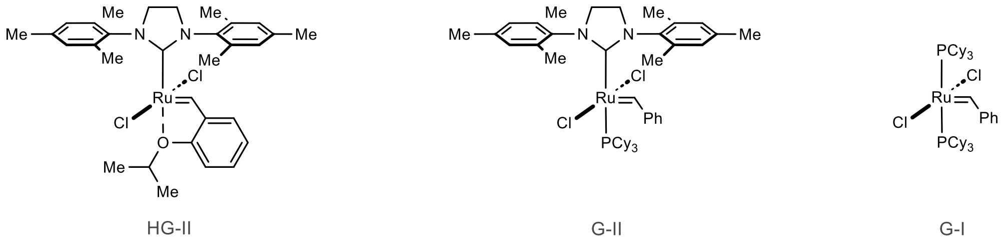

Entry	Solvent	Concentration	Catalyst	Temperature	1a equiv	Yield[⊥]
1	DCM	0.1 M	HG-II (5 mol%)	40 °C	4	>99%
2	DCM	0.1 M	G-II (5 mol%)	40 °C	4	97%
3	DCM	0.1 M	G-I (5 mol%)	40 °C	4	11%

0.1 mmol scale alkene substrate, ⊥ ^{1}H NMR yields relative to methyl benzoate internal standard

Table S4. 1CTR **1a** *vs* **1b** screen

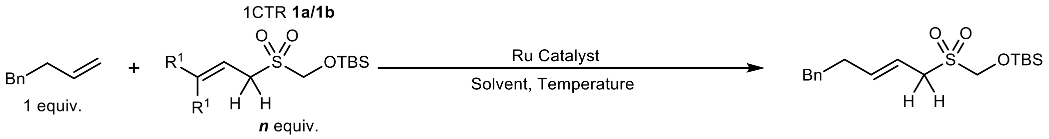

Entry	Solvent	Concentration	Catalyst	Temperature	1CTR	1CTR equiv	Yield[⊥]
1	DCM	0.1 M	HG-II (5 mol%)	40 °C	**1a** R^1=H (allyl)	4	>99%
2	DCM	0.1 M	HG-II (5 mol%)	40 °C	**1b** R^1=Me (prenyl)	3	53%
3	PhMe	0.1 M	HG-II (5 mol%)	100 °C	**1b** R^1=Me (prenyl)	3	53%

0.1 mmol scale alkene substrate, ⊥ ^{1}H NMR yields relative to methyl benzoate internal standard

Table S5. Monosubstituted alkene retro-ene optimisation

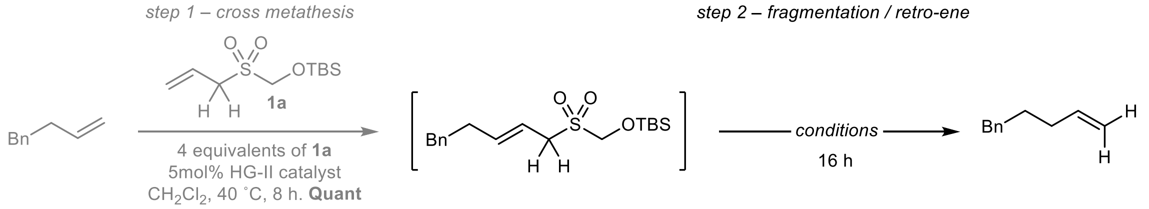

Entry	Solvent	Acid/F⁻ Source	Equiv/mL*	Temperature	Yield⊥
1	MeOH	Pyridine·HF	3	40 °C	0%
2	MeOH	TBAF·3H_2O	1.25	40 °C	0%
3	TFE	TBAF·3H_2O	1.25	40 °C	8%
4	MeOH	Succinic acid: TBAF·3H_2O	3:1.25	40 °C	28%
5	MeOH	Citric acid:TBAF·3H_2O	3:1.25	40 °C	>99%
6	MeOH	Camphorsulfonic acid	3	40 °C	52%
7	MeOH	Trifluoroacetic acid	3	40 °C	57%
8	-	2M $HCl_{(aq)}$ in MeOH	0.5 mL	40 °C	>99%

0.1 mmol scale alkene substrate, *equivalents relative to 1CTR used in reaction, ⊥ ^{1}H NMR yields relative to methyl benzoate internal standard

3.2. General procedure for the one-carbon homologation of monosubstituted alkenes

Step 1: A 5 mL round-bottom flask was charged with a magnetic stirrer bar, 1CTR **1a** (200 mg, 0.8 mmol, 4.00 equiv) and alkene substrate (0.2 mmol, 1.00 equiv). The round-bottom flask was fitted to a reflux condenser (Teflon tape around the ground glass joint, suba-seal on condenser outlet, connected to a N_2 line with a needle through the suba-seal) and evacuate-refilled with N_2 (3 cycles). The flask was then heated to 40 °C in an oil bath, and dichloromethane (0.25 mL) was added through the top of the condenser to dissolve the starting materials. A stock solution of HG-II catalyst (6.26 mg/mL, 0.05 equiv) was prepared in dichloromethane and 1 mL of the stock solution was added, followed by dichloromethane (0.25 mL). An outlet needle was pierced through the suba-seal to help purge the reaction vessel of ethylene, and the reaction was heated for the specified amount of time with stirring.

Step 2: Following the completion of the metathesis reaction (monitored by TLC or ^{1}H NMR) one of two possible retro-ene conditions was used:

Retro-ene condition A (0.2 mmol alkene substrate): A stock solution of $HCl_{(aq)}$ (12M) in MeOH was made up in the ratio of 5:1 MeOH:$HCl_{(aq)}$ (final concentration 2M in MeOH). MeOH-HCl (1 mL) was added through the top of the condenser using a syringe. The reaction was heated at 40 °C in an oil bath for 16 h.

The reaction mixture was transferred to a separatory funnel containing $NaHCO_3$ (approx. 50 mL). The aqueous phase was extracted 3x using Et_2O or EtOAc (if the alkene substrate is insoluble in Et_2O). The combined organic phases were dried over $MgSO_4$, filtered and concentrated *in vacuo*. The resulting crude alkene was taken up in $CDCl_3$ (approx. 1.5 mL) followed by the addition of methyl benzoate (25 μL, 0.2 mmol, 1.00 equiv) or 1,1,2,2-tetrachloroethane (21 μL, 0.2 mmol, 1.00 equiv) as an internal standard and an aliquot was analysed by ^{1}H NMR to determine the assay yield.

Retro-ene condition B (0.2 mmol alkene substrate): Citric acid (461 mg, 2.4 mmol, 3.00 equiv*) and TBAF·$3H_2O$ (316 mg, 1 mmol, 1.25 equiv*) were dissolved in 2 mL MeOH (sonication required). The solution was added through the top of the condenser using a syringe. The reaction was heated at 40 °C in an oil bath for 16 h. **equivalents relative to 1CTR used in cross-metathesis reaction*

The reaction mixture was transferred to a separatory funnel containing $NaHCO_3$ (approx. 50 mL). The aqueous phase was extracted 3x using Et_2O or EtOAc (if the alkene substrate is insoluble in Et_2O). The combined organic phases were dried over $MgSO_4$, filtered and concentrated *in vacuo*. The resulting crude alkene was taken up in $CDCl_3$ (approx. 1.5 mL) followed by the addition of methyl benzoate (25 μL, 0.2 mmol, 1.00 equiv) or 1,1,2,2-tetrachloroethane (21 μL, 0.2 mmol, 1.00 equiv) as an internal standard and an aliquot was analysed by ^{1}H NMR to determine the assay yield.

3.3. 1,1-disubstituted alkene optimisation studies

A 10 mL microwave vial was charged with a magnetic stirrer bar, 1CTR **1b** (*n* equiv), rotenone (19.7 mg, 0.05 mmol, 1.00 equiv), and catalyst (n equiv). The vial was sealed using a crimper and evacuate-refilled with N_2 (3-cycles). Solvent (0.5 mL) was added via a syringe and the reaction was heated at the specified temperature for a specified amount of time with stirring, after which the catalyst was quenched using butyl vinyl ether (~0.1 mL). The solvent was removed *in vacuo* and conversion was measured by ^{1}H NMR via the addition of an internal standard, methyl benzoate (6.3 µL, 0.05 mmol, 1.00 equiv).

Table S6. 1,1-disubstituted alkene cross-metathesis optimisation

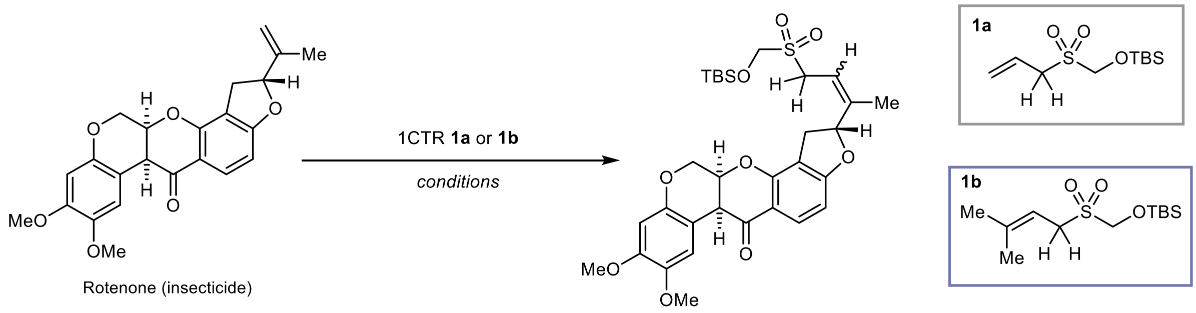

Entry	1CTR	Concentration	1CTR equiv	Catalyst (20 mol%)	Solvent	Temperature	Time	Yield$^{\perp}$
1	**1a**	0.1 M	10	HG-II	DCM	40 °C	16 h	44%
2	**1a**	0.1 M	10	HG-II	DCE	100 °C	5 h	48%
3	**1a**	0.1 M	10	HG-II	DCE	100 °C	16 h	38%
4	**1a**	0.1 M	10	M721	DCE	100 °C	5 h	trace
5	**1a**	0.1 M	10	nitro-Grela I_2 SiPr	DCE	100 °C	5 h	5%
6	**1b**	0.1 M	2	HG-II	DCE	100 °C	16 h	76%
7	**1b**	0.1 M	3	HG-II	DCE	100 °C	16 h	94%
8	**1b**	0.1 M	5	HG-II	DCE	100 °C	16 h	90%

0.05 mmol scale alkene substrate, $^{\perp}$ ^{1}H NMR yields relative to methyl benzoate internal standard

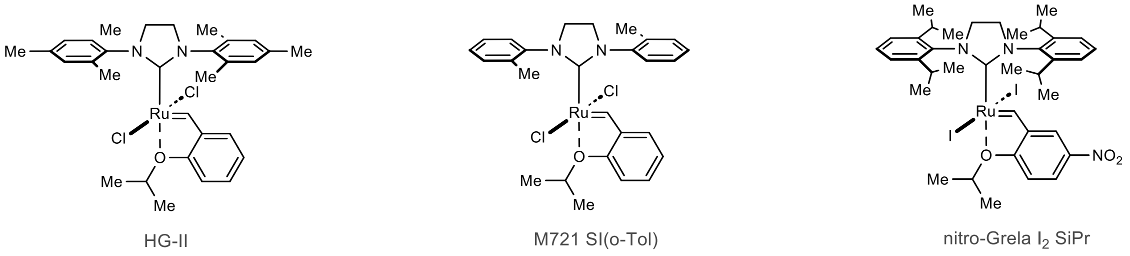

Table S7. 1,1-disubstituted alkene retro-ene optimisation

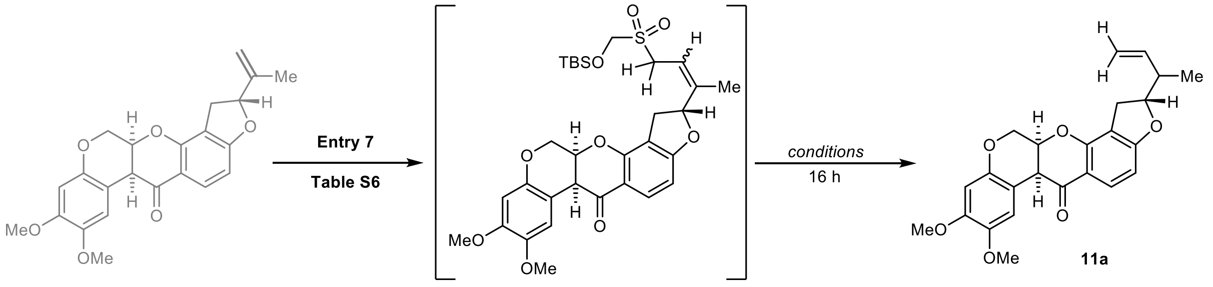

Entry	Solvent	Acid/F⁻ Source	Equivalents/mL*	Temperature	Yield⊥
1	-	2M $HCl_{(aq)}$ in MeOH	0.25 mL	40 °C	0%
2	MeOH	Citric acid: TBAF·$3H_2O$	3:1.25	40 °C	Trace (LC-MS)
3	MeOH	Citric acid: TBAF·$3H_2O$	3:1.25	80 °C	54%
4	MeOH	Citric acid:TBAF·$3H_2O$	3:1.25	100 °C	80%

0.1 mmol scale alkene substrate, *equivalents relative to 1CTR used in reaction, ⊥ ^{1}H NMR yields relative to methyl benzoate internal standard

3.4. General procedure for the one-carbon homologation of 1,1-disubstitutued alkenes

Step 1:

A 20 mL microwave vial was charged with a magnetic stirrer bar, 1CTR **1b** (167 mg, 0.6 mmol, 3.00 equiv), HG-II catalyst (25 mg, 0.04 mmol, 0.20 equiv) and alkene substrate (0.2 mmol, 1.00 equiv). The vial was sealed using a crimper and evacuate-refilled with N_2 (3-cycles). DCE or PhMe (1.5 mL) was added via a syringe and the reaction was heated at 100 °C for the specified amount of time with stirring, after which the catalyst was quenched using butyl vinyl ether (~0.1 mL).

Step 2:

Retro-ene condition C (0.2 mmol alkene substrate): Citric acid (346 mg, 1.80 mmol, 3.00 equiv*) and TBAF·$3H_2O$ (237 mg, 0.75 mmol, 1.25 equiv*) were dissolved in 1.5 mL MeOH (sonication required). The solution was added through the septum of the microwave vial and the reaction was heated and stirred at 100 °C in an oil bath for 16 h. **equivalents relative to 1CTR used in cross-metathesis reaction*

The reaction mixture was transferred to a separatory funnel containing $NaHCO_3$ (approx. 50 mL). The aqueous phase was extracted 3x using Et_2O. The combined organic phases were dried over $MgSO_4$, filtered and concentrated *in vacuo*. The resulting crude alkene was taken up in $CDCl_3$ (approx. 1.5 mL) followed by the addition of methyl benzoate (25 µL, 0.2 mmol, 1.00 equiv) or 1,1,2,2-tetrachloroethane (21 µL, 0.2 mmol, 1.00 equiv) as an internal standard. An an aliquot was analysed by ^{1}H NMR to determine the assay yield.

3.5. General procedure for the one-carbon homologation of methyl terminated 1,2-disubstituted alkenes

Step 1:

A 5 mL round-bottom flask was charged with a magnetic stirrer bar, 1CTR **1c** (132 mg, 0.5 mmol, 5.00 equiv) and alkene substrate (0.1 mmol, 1.00 equiv). The round-bottom flask was fitted to a reflux condenser (Teflon tape around the ground glass joint, suba-seal on condenser outlet, connected to a N_2 line with a needle through the suba-seal) and evacuate-refilled with N_2 (3 cycles). The flask was then heated to 40 °C in an oil bath and dichloromethane (0.25 mL) was added through the top of the condenser to dissolve the starting materials. A stock solution of HG-II (12.6 mg/mL, 0.10 equiv) was prepared in dichloromethane and 0.5 mL of the stock solution was added, followed by dichloromethane (0.25 mL). An outlet needle was pierced through the suba-seal to help purge the reaction vessel of ethylene/propylene, and the reaction was heated for the specified amount of time with stirring. After reaction completion, the catalyst was quenched using ethyl vinyl ether (~0.1 mL).

Note: Methyl-terminated 1,2-disubstitued alkenes underwent one-pot homologation successfully. However, 1,2-alkene isomerization ~5% was observed. This phenomenon was attributed to the formation of ruthenium hydride species derived from catalyst decomposition. Quenching of the cross-metathesis reaction with ethyl vinyl ether, followed by chromatographic purification, was effective in removing the ruthenium catalyst and preventing isomerisation from occurring in the retro-ene reaction.

Step 2:

After chromatographic purification, the latent homolog was concentrated into a 10 mL microwave vial. The vial was sealed using a crimper, back-refilled with N_2 (3-cycles), charged with dichloromethane (1 mL) and heated to 40 °C.

Retro-ene condition D (0.1 mmol alkene substrate): Citric acid (288 mg, 1.5 mmol, 3.00 equiv*) and TBAF·3H_2O (197 mg, 0.625 mmol, 1.25 equiv*) were dissolved in 2 mL MeOH (sonication required). The solution was added through the septum of the microwave vial and the reaction was heated and stirred at 40 °C in an oil bath for 16 h. **equivalents relative to 1CTR used in cross-metathesis reaction if 1CTR/1CTR dimer elutes with latent homolog after purification of the cross-metathesis reaction.*

The reaction mixture was transferred to a separatory funnel containing $NaHCO_3$ (approx. 50 mL). The aqueous phase was extracted 3x using Et_2O. The combined organic phases were dried over $MgSO_4$, filtered and concentrated *in vacuo*. The resulting crude alkene was taken up in $CDCl_3$ (approx. 1.5 mL) followed by the addition of methyl benzoate (12.6 µL, 0.1 mmol, 1.00 equiv) or 1,1,2,2-tetrachloroethane (10.5 µL, 0.1 mmol, 1.00 equiv) as an internal standard and an aliquot analysed by ^{1}H NMR to determine the assay yield.

3.6. Alkene selectivity experiments

Fig. S1. Monosubstituted *vs* 1,1-disubstituted alkene selectivity in cross-metathesis.

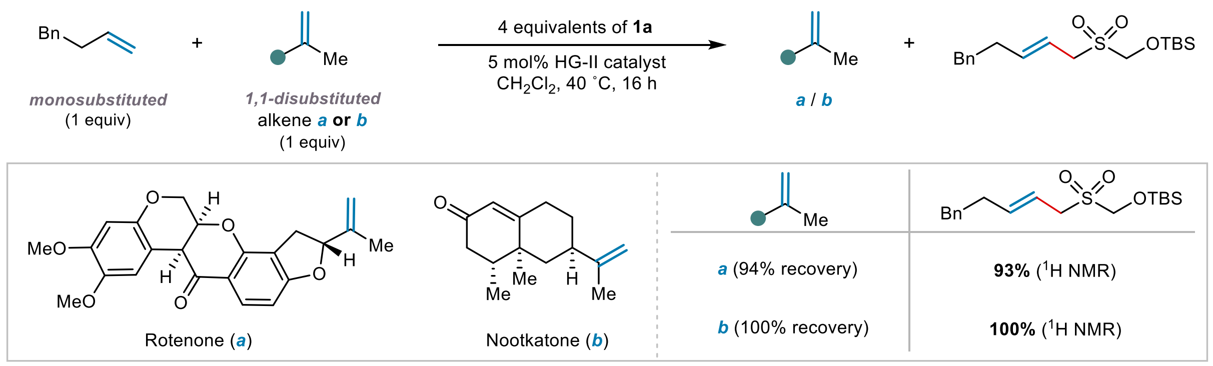

1,1-disubstituted alkenes were found to be possess low reactivity in cross-metathesis with 1CTR **1a** (Table S6, Entries 1–5). It was found that selective cross-metathesis of a monosubstitued alkene could be performed in the presence of equimolar 1,1-disubstitued alkene (**Fig. S1.**).

Fig. S2. *E*- *vs* *Z*- latent homolog in retro-ene reaction

A 10 mL microwave vial was charged with a magnetic stirrer bar and *E*/*Z*-**17** (36.8 mg, 0.1 mmol, 1.00 equiv). The vial was evacuate-refilled with N_2 (3-cycles), charged with dichloromethane (0.5 mL), and heated to 40 °C in an oil bath. Citric acid (57.6 mg, 0.3 mmol, 3.00 equiv) and TBAF·$3H_2O$ (39.4 mg, 0.125 mmol, 1.25 equiv) were dissolved in MeOH (0.5 mL). The solution was added through the septum of the microwave vial and the reaction was heated and stirred at 40 °C in an oil bath for 16 h. After completion of the reaction, methyl benzoate (12.6 µL, 0.1 mmol, 1.00 equiv) was added, and an aliquot was taken from the crude to determine conversion by ^{1}H NMR. The *E*/*Z* ratio of the retro-ene products (**18**) were determined by GC-MS analysis of the crude reaction.

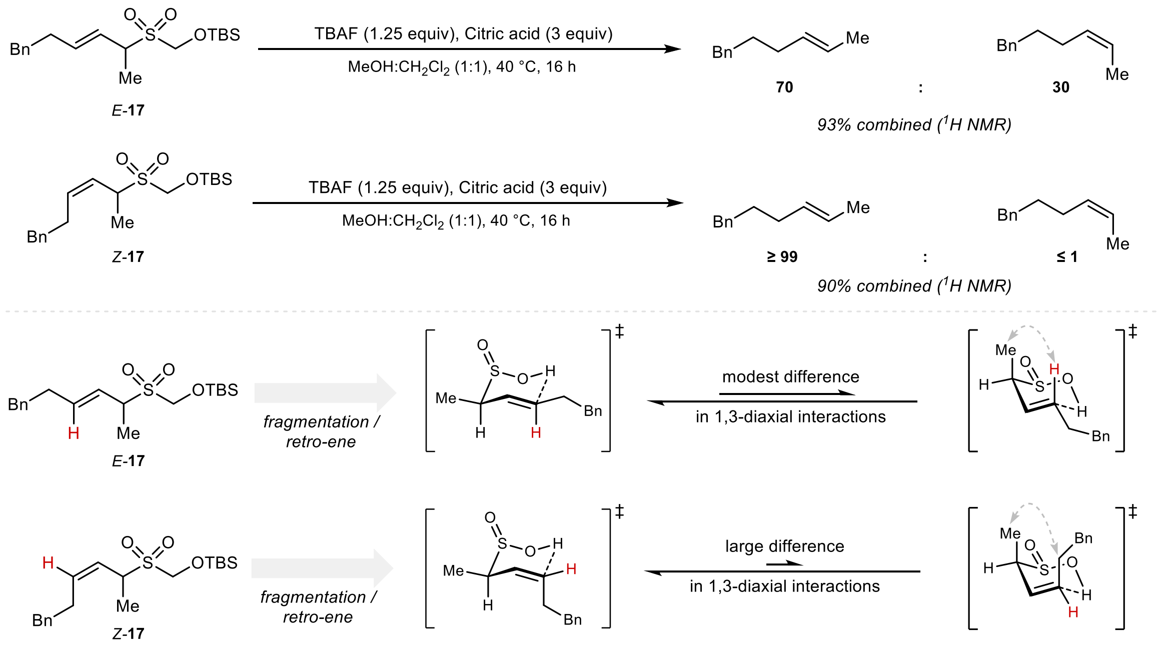

4. Synthesis of 1-C Transfer Reagents (1CTR's)

TBS protection of Rongalite™

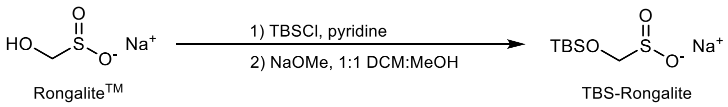

TBS-Rongalite was synthesised according to modification of a reported procedure.[1]

tert-Butyldimethylchlorosilane (108.5 g, 0.72 mol, 4.00 equiv) and sodium hydroxymethanesulfinate dihydrate (27.8 g, 0.18 mol, 1.00 equiv) were charged to a 1 L 3-neck round-bottom flask containing a magnetic stirrer bar, and distributed evenly. The round-bottom flask was fitted with a pressure-equalising dropping funnel and charged with pyridine (180 mL, 2.23 mol, 12.40 equiv). The round-bottom flask was cooled to 0 °C using an ice bath and pyridine was added dropwise over 15 minutes. The reaction was left to stir overnight.

Pyridine was removed *in vacuo* (5 mbar). The white slurry was filtered through a pad of celite, and the filter cake was washed with cold hexane (2 x 50 mL). The filtrate was concentrated *in vacuo* to yield a yellow oil (if there is a significant quantity of precipitate, further filtration may be necessary). To a round-bottom flask containing the yellow oil, anhydrous DCM (200 mL) and MeOH (200 mL) were added. The round-bottom flask was cooled to 0 °C using an ice bath. Sodium methoxide (39 mL, 0.17 mol, 25 wt% in MeOH) and MeOH (~40 mL) were charged to the pressure equalising dropping funnel. The sodium methoxide solution was added dropwise to the reaction vessel over 30 minutes with stirring. After the addition was complete, the reaction was stirred for a further 15 minutes at 0 °C, followed by a further 3 h at room temperature. Solvent was removed under reduced pressure (down to 1 mbar), yielding a white solid. To aid removal of TBSOH, Toluene (200 mL) was added to the flask and volatiles were removed *in vacuo*. The white solid was dried under a stream of N_2 overnight, followed by a high vacuum for 2 days, yielding TBS-Rongalite as a white solid (39.0 g, 0.168 mol, 93%).

((allylsulfonyl)methoxy)(tert-butyl)dimethylsilane - 1CTR (1a)

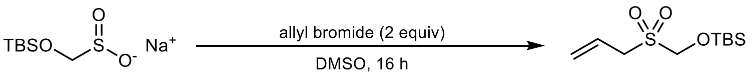

1CTR **1a**

TBS-Rongalite (10 g, 43.1 mmol, 1.00 equiv) was added to a 250 mL round bottom flask containing a stirrer bar and back-refilled with N_2 (3-cycles). DMSO (200 mL) was added via a cannula, and the reaction was stirred for 30 min. Allyl bromide (7.5 mL, 86.2 mmol, 2.00 equiv) was added and the reaction was stirred for 16 h. The reaction was cooled to 0 °C using an ice bath and quenched with $NaHCO_3$ (25 mL). The reaction mixture was transferred to a 1L separatory funnel containing H_2O (300 mL). The product was extracted into PE (50 mL x 4). The combined organic fractions were washed with brine, dried over $MgSO_4$, and concentrated *in vacuo*. The crude oil was purified by flash column chromatography (0-15% EA in 40-60 PE) to give the product as a colourless oil (7.61 g, 30.4 mmol, 70%).

^{1}H NMR (400 MHz, Chloroform-*d*) δ 5.93 (ddt, J = 16.9, 10.2, 7.4 Hz, 1H), 5.60 – 5.39 (m, 2H), 4.55 (s, 2H), 3.77 (d, *J* = 7.5 Hz, 2H), 0.96 (s, 9H), 0.21 (s, 6H).
^{13}C NMR (101 MHz, Chloroform-*d*) δ 124.7, 124.6, 76.0, 53.6, 25.5, 18.2, -5.4 .
HRMS (ESI+) m/z calculated for $C_{10}H_{23}O_3SiS$ $[M+H]^+$ 251.11317, found 251.11315 (Δ = 0.08 ppm).
IR (film, cm^{-1}) 2954, 2930, 2887, 2858, 1330, 1309, 1115, 830, 781

tert-butyldimethyl(((3-methylbut-2-en-1-yl)sulfonyl)methoxy)silane - 1CTR (1b)

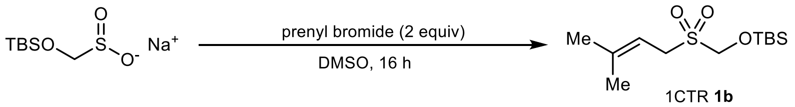

1CTR **1b**

TBS-Rongalite (6.5 g, 28 mmol, 1.00 equiv) was added to a 250 mL round bottom flask containing a stirrer bar and back-refilled with N_2 (3-cycles). DMSO (120 mL) was added, and the reaction was stirred for 30 min. Prenyl bromide (6.5 mL, 56 mmol, 2.00 equiv) was added and the reaction was stirred for 16 h. The reaction was cooled to 0 °C using an ice bath and quenched with $NaHCO_3$ (25 mL). The reaction mixture was transferred to a 500 mL separatory funnel containing H_2O (200 mL). The product was extracted into PE (4 x 30 mL). The combined organic fractions were washed with brine, dried over $MgSO_4$, and concentrated *in vacuo*. The crude oil was purified by flash column chromatography (0-10% EA in 40-60 PE) to give the product as a yellow oil (5.85 g, 21 mmol, 75%).

^{1}H NMR (500 MHz, Chloroform-*d*) δ 5.33 – 5.26 (m, 1H), 4.49 (s, 2H), 3.75 (d, *J* = 7.9 Hz, 2H), 1.85 (d, *J* = 1.3 Hz, 3H), 1.77 (d, *J* = 1.6 Hz, 3H), 0.95 (s, 9H), 0.21 (s, 6H).
^{13}C NMR (126 MHz, Chloroform-*d*) δ 142.8, 110.0, 76.0 , 49.0, 26.1, 25.6, 18.4, 18.2, -5.3.
HRMS (ESI+) m/z calculated for $C_{12}H_{27}O_3SiS$ $[M+H]^+$ 279.1450, found 279.1462 (Δ = 4.3 ppm).
IR (film, cm^{-1}) 2953, 2929, 2857, 1302, 1114, 830, 815, 781

((but-3-en-2-ylsulfonyl)methoxy)(tert-butyl)dimethylsilane - 1CTR (1c)

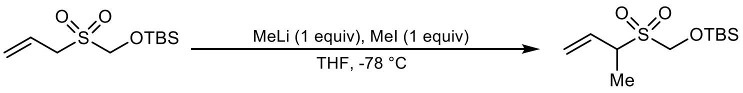

1CTR (**1a**) (5.00 g, 20 mmol, 1.00 equiv) was added to a 250 mL round bottom flask containing a stirrer bar and back-refilled with N_2 (3-cycles). THF (100 mL) was added, and the reaction was cooled to −78 °C using a dry ice bath. Methyllithium solution (12.5 mL, 20 mmol, 1.6M in Et_2O, 1.00 equiv) was added via a syringe and the reaction was stirred for 1 hour at − 78 °C. Iodomethane (1.25 mL, 20 mmol, 1.00 equiv) was added and the reaction was stirred for a further hour at − 78 °C before removing the ice bath and allowing the reaction to warm to room temperature for a further 2 hours. The reaction was quenched by the addition of aqueous ammonium chloride (1 mL). THF was removed *in vacuo* and the crude was transferred to a separatory funnel where the product was extracted using diethyl ether. The crude oil was purified by flash column chromatography (0-15% EA in 40-60 PE) to give the product as a colourless oil (2.97 g, 11.25 mmol, 56%)

^{1}H NMR (400 MHz, Chloroform-*d*) δ 5.90 (ddd, *J* = 17.0, 10.3, 8.5 Hz, 1H), 5.50 – 5.40 (m, 2H), 4.75 (d, *J* = 11.5 Hz, 1H), 4.45 (d, *J* = 11.6 Hz, 1H), 3.97 – 3.85 (m, 1H), 1.52 (d, *J* = 7.1 Hz, 3H), 0.95 (s, 9H), 0.21 (s, 3H), 0.20 (s, 3H).
^{13}C NMR (101 MHz, Chloroform-*d*) δ 131.8, 121.8, 75.3, 58.0, 25.5, 18.2, 11.6, -5.3, -5.5.
HRMS (ESI+) m/z calculated for $C_{11}H_{24}O_3SSiNa$ $[M+Na]^+$ 287.1108, found 287.1099 (Δ = -3.2 ppm).
IR (film, cm^{-1}): 2953, 2930, 2886, 2858, 1305, 1113, 830, 815, 781.

tert-butyldimethyl(((4-methylpent-1-en-3-yl)sulfonyl)methoxy)silane - 1CTR (1d)

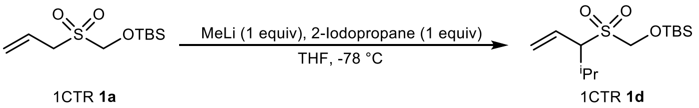

1CTR (**1a**) (4.1 g, 16.4 mmol, 1.00 equiv) was added to a 250 mL round bottom flask containing a stirrer bar and back-refilled with N_2 (3-cycles). THF (80 mL) was added and the reaction was cooled to − 78 °C using a dry ice bath. Methyllithium solution (10.3 mL, 16.4 mmol, 1.6M in Et_2O, 1.00 equiv) was added via a syringe and the reaction was stirred for 1 hour at −78 °C. 2-iodopropane (1.64 mL, 16.4 mmol, 1.00 equiv) was added and the reaction was stirred for a further hour at − 78 °C before removing the ice bath and allowing the reaction to warm to room temperature for a further 2 hours. The reaction was quenched by the addition of aqueous ammonium chloride (1 mL). THF was removed *in vacuo* and the crude was transferred to a separatory funnel where the product was extracted using diethyl ether. The crude was purified by flash column chromatography (0-15% EA in 40-60 PE) to give the product as a colourless oil (2.04 g, 6.99 mmol, 43%).

^{1}H NMR (700 MHz, $CDCl_3$) δ 5.94 (dt, *J* = 17.1, 10.3 Hz, 1H), 5.52 (dd, *J* = 10.2, 1.5 Hz, 1H), 5.39 (dd, *J* = 17.0, 0.9 Hz, 1H), 4.78 (d, *J* = 11.4 Hz, 1H), 4.25 (d, *J* = 11.4 Hz, 1H), 3.68 – 3.63 (m, 1H), 2.69 – 2.60 (m, 1H), 1.13 (d, *J* = 6.9 Hz, 3H), 1.04 (d, *J* = 6.9 Hz, 3H), 0.94 (s, 9H), 0.21 (s, 3H), 0.18 (s, 3H).
^{13}C NMR (176 MHz, Chloroform-*d*) δ 128.5, 124.1, 75.6, 67.2, 25.6, 25.6, 21.7, 18.2, 18.2, -5.2, -5.6.
HRMS (ESI+) m/z calculated for $C_{13}H_{28}O_3SSi$ $[M+Na]^+$ 315.1426 found 315.1425 (Δ = - 0.3 ppm).
IR (film, cm^{-1}): 2957, 2930, 2858, 1472, 1326, 1304, 1116, 997.

(((but-3-en-2-yl-1,1,1-d_3)sulfonyl)methoxy)(tert-butyl)dimethylsilane – 1CTR (1e)

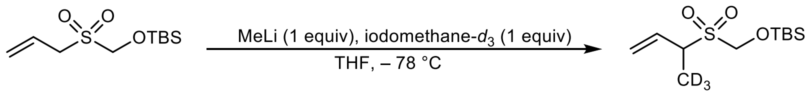

1CTR (**1a**) (5.00 g, 20 mmol, 1.00 equiv) was added to a 250 mL round bottom flask containing a stirrer bar and back-refilled with N_2 (3-cycles). THF (100 mL) was added, and the reaction was cooled to −78 °C using a dry ice bath. Methyllithium solution (12.5 mL, 20 mmol, 1.6M in Et_2O, 1.00 equiv) was added via a syringe and the reaction was stirred for 1 hour at −78 °C. Iodomethane-d_3 (1.25 mL, 20 mmol, 1.00 equiv) was added and the reaction was stirred for a further hour at −78 °C before removing the ice bath and allowing the reaction to warm to room temperature for a further 2 hours. The reaction was quenched by the addition of aqueous ammonium chloride (1 mL). THF was removed *in vacuo* and the crude was transferred to a separatory funnel where the product was extracted using diethyl ether. The crude was purified by flash column chromatography (0-15% EA in 40-60 PE) to give the product as a colourless oil (2.73 g, 10.21 mmol, 51%)

^{1}H NMR (400 MHz, $CDCl_3$) δ 5.91 (ddd, J = 17.2, 10.2, 8.6 Hz, 1H), 5.50 – 5.40 (m, 2H), 4.75 (d, J = 11.5 Hz, 1H), 4.46 (d, J = 11.5 Hz, 1H), 3.90 (d, J = 8.6 Hz, 1H), 0.95 (s, 9H), 0.21 (s, 3H), 0.20 (s, 3H).
^{13}C NMR (101 MHz, $CDCl_3$) δ 131.7, 121.8, 75.3, 57.8, 25.5, 18.2, 10.9, -5.3, -5.5.
HRMS (ESI+) m/z calculated for $C_{11}H_{21}D_3O_3NaSSi$ $[M+Na]^+$ 290.1296, found 290.1288 (Δ = -2.7 ppm).
IR (film, cm^{-1}): 2953, 2930, 2887, 2857, 1472, 1305, 1114, 830

tert-butyl(((1-fluoroallyl)sulfonyl)methoxy)dimethylsilane- 1CTR (1f)

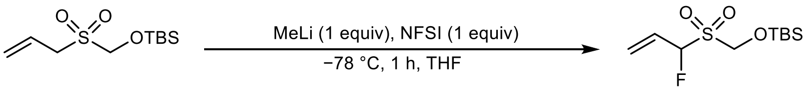

1CTR (**1a**) (5.00 g, 20 mmol, 1.00 equiv) was added to a 250 mL round bottom flask containing a stirrer bar and back-refilled with N_2 (3-cycles). THF (100 mL) was added, and the reaction was cooled to −78 °C using a dry ice bath. Methyllithium solution (12.5 mL, 20 mmol, 1.6M in Et_2O, 1.00 equiv) was added via a syringe and the reaction was stirred for 1 hour at −78 °C. NFSI (6.3 g, 20 mmol, 1.00 equiv) in 15 mL THF was and to the reaction, and stirred for a further hour at −78 °C before removing the ice bath and allowing the reaction to warm to room temperature for a further 2 hours. The reaction was quenched by the addition of aqueous ammonium chloride (1 mL). THF was removed *in vacuo* and the crude was transferred to a separatory funnel where the product was extracted using diethyl ether. The crude was purified by C18 reverse phase chromatography (0-100% Water-Acetonitrile) to give the product as a colourless oil (2.18 g, 8.13 mmol, 41%)

^{1}H NMR (500 MHz, $CDCl_3$) δ 6.15 – 6.01 (m, 1H), 5.88 – 5.72 (m, 3H), 4.95 (dd, *J* = 11.8, 2.3 Hz, 1H), 4.58 (dd, *J* = 11.8, 1.2 Hz, 1H), 0.96 (s, 9H), 0.22 (s, 3H), 0.22 (s, 3H).
^{13}C NMR (126 MHz, $CDCl_3$) δ 125.9 (d, $^{3}J_{CF}$ = 11.1 Hz), 123.3 (d, $^{2}J_{CF}$ = 18.4 Hz), 96.8 (d, $^{1}J_{CF}$ = 238.2 Hz), 75.5, 25.5, 18.2, -5.3, -5.6.
^{19}F NMR (471 MHz, $CDCl_3$) δ -180.1.
IR (film, cm^{-1}) 2931, 2887, 2858, 1472, 1339, 1118, 829, 783
HRMS (ESI+) m/z calculated for $C_{10}H_{21}O_3FNaSSi$ $[M+Na]^+$ 291.0857, found 291.0847 (Δ = −3.4 ppm).

5. Substrate Scope

Methyl dodec-11-enoate (4b)

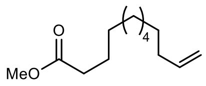

Prepared according to the general procedure for homologation of monosubstituted alkenes (3.2) from methyl 10-undecenoate (45.1 µL, 0.2 mmol, 1.00 equiv) and stirred for 8 h, followed by retro-ene condition A. The residue was purified by flash column chromatography on silica gel (5% Et_2O in 40-60 PE) to give the product as a colourless oil (80%, 34.0 mg, 0.160 mmol).

^{1}H NMR (500 MHz, Chloroform-d) δ 5.83 (ddt, J = 17.0, 10.2, 6.7 Hz, 1H), 5.06 – 4.90 (m, 2H), 3.69 (s, 3H), 2.32 (t, J = 7.6 Hz, 2H), 2.10 – 2.01 (m, 2H), 1.68 – 1.61 (m, 2H), 1.42 – 1.28 (m, 12H).
^{13}C NMR (126 MHz, Chloroform-*d*) δ 174.4, 139.2, 114.1, 51.4, 34.1, 33.8, 29.4, 29.4, 29.2, 29.1, 29.1, 28.9, 25.0.
HRMS (ESI+) m/z calculated for $C_{13}H_{25}O_2$ $[M+H]^+$ 213.18491, found 213.18505 (Δ = 0.6 ppm).
IR (film, cm^{-1}) 3076, 2924, 2854, 1740, 1436, 1195, 1169, 993, 908.

Nonadec-1-ene (4c)

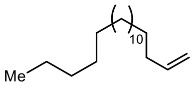

Prepared according to the general procedure for homologation of monosubstituted alkenes (3.2) from 1-octadecene (64 µL, 0.2 mmol, 1.00 equiv) and stirred for 8 h, followed by retro-ene condition A. The residue was purified by flash column chromatography on silica gel (100% pentane) to give the product as a colourless oil (82%, 43.7 mg, 0.164 mmol).

^{1}H NMR (500 MHz, Chloroform-*d*) δ 5.84 (ddt, *J* = 17.0, 10.2, 6.7 Hz, 1H), 5.06 – 4.92 (m, 2H), 2.11 – 2.03 (m, 2H), 1.46 – 1.14 (m, 30H), 0.91 (t, *J* = 7.0 Hz, 3H).
^{13}C NMR (126 MHz, Chloroform-*d*) δ 139.3, 114.1, 77.3, 77.0, 76.8, 33.8, 32.0, 29.7, 29.7, 29.7, 29.7, 29.7, 29.7, 29.7, 29.6, 29.5, 29.4, 29.2, 29.0, 22.7, 14.1.

4c is a known compound and its NMR spectra are in accord with published data.[2]

4-(4-allylcyclohexyl)benzonitrile (4d)

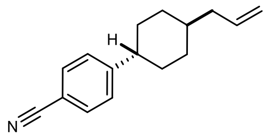

Prepared according to the general procedure for homologation of monosubstituted alkenes (3.2) from trans-4-(4-Vinylcyclohexyl)benzonitrile (42.3 mg, 0.2 mmol, 1.00 equiv) and stirred for 8 h, followed by retro-ene condition A. The residue was purified by flash column chromatography on silica gel PE:Et_2O 20:1 to give the product as a colourless oil (92%, 41.9 mg, 0.186 mmol).

^{1}H NMR (500 MHz, Chloroform-*d*) δ 7.59 (d, *J* = 8.4 Hz, 2H), 7.32 (d, *J* = 8.3 Hz, 2H), 5.83 (ddt, *J* = 17.2, 10.2, 7.1 Hz, 1H), 5.10 – 4.97 (m, 2H), 2.54 (tt, *J* = 12.2, 3.2 Hz, 1H), 2.09 – 2.00 (m, 2H), 1.96 – 1.87 (m, 4H), 1.53 – 1.39 (m, 3H), 1.20 – 1.05 (m, 2H).
^{13}C NMR (126 MHz, Chloroform-*d*) δ 153.2, 137.2, 132.2, 127.1, 119.2, 115.7, 108.9, 44.7, 41.6, 37.2, 33.8, 32.9.
HRMS (ESI+) m/z calculated for $C_{16}H_{20}N$ $[M+H]^+$ 226.15903, found 226.15852 (Δ = 2.3 ppm)
IR (film, cm^{-1}): 3074, 2920, 2850, 2226, 1639, 1607, 1503, 1447, 1415, 1371.

4-Allyl-4'-butyl-1,1'-bi(cyclohexane) (4e)

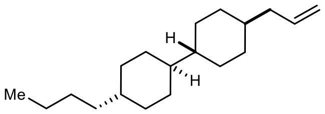

Prepared according to the general procedure for homologation of monosubstituted alkenes (3.2) from trans,trans-4-Butyl-4'-vinyl-bicyclohexyl (49.7 mg, 0.2 mmol, 1.00 equiv) and stirred for 8 h, followed by retro-ene condition A. The residue was purified by flash column chromatography on silica gel (100% pentane) to give the product as a colourless oil (93%, 48.9 mg, 0.186 mmol).

^{1}H NMR (400 MHz, Chloroform-*d*) δ 5.81 (ddt, *J* = 17.2, 10.1, 7.1 Hz, 1H), 5.04 – 4.93 (m, 2H), 1.96 (t, *J* = 7.0 Hz, 2H), 1.80 – 1.68 (m, 8H), 1.33 – 1.24 (m, 5H), 1.20 – 1.11 (m, 3H), 1.03 – 0.83 (m, 13H).
^{13}C NMR (101 MHz, Chloroform-*d*) δ 137.8, 115.1, 43.5, 43.4, 41.9, 38.0, 37.9, 37.2, 33.7, 33.3, 30.1, 30.0, 29.3, 23.0, 14.2.
HRMS (ESI+) m/z calculated for $C_{19}H_{35}$ $[M+H]^+$ 263.2739, found 263.2727 (Δ = -4.6 ppm)
IR (film, cm^{-1}) 3076, 2910, 2847, 1446, 993, 910

4-(But-3-en-1-yl)-1,2-dimethoxybenzene (4f)

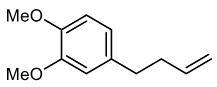

Prepared according to the general procedure for homologation of monosubstituted alkenes (3.2) from methyl eugenol (34.5 µL, 0.2 mmol, 1.00 equiv), with the exception that 1CTR **1a** (250 mg, 1 mmol, 5.00 equiv) was used. The reaction was stirred for 16 h, followed by retro-ene condition A. The residue was purified by flash column chromatography on silica gel (0-20% EtOAc in 40-60 PE) to give the product as a colourless oil (30.7 mg, 0.160 mmol, 80%).

^{1}H NMR (400 MHz, Chloroform-*d*) δ 6.80 (d, *J* = 8.7 Hz, 1H), 6.73 (d, *J* = 7.2 Hz, 2H), 5.86 (ddt, *J* = 17.0, 10.3, 6.5 Hz, 1H), 5.10 – 4.95 (m, 2H), 3.87 (s, 3H), 3.86 (s, 3H), 2.66 (dd, *J* = 9.0, 6.6 Hz, 2H), 2.41 – 2.31 (m, 1H).
^{13}C NMR (101 MHz, Chloroform-*d*) δ 148.8, 147.2, 138.2, 134.6, 120.2, 114.9, 111.8, 111.2, 55.9, 55.8, 35.7, 35.0.

4f is a known compound and its NMR spectra are in accord with published data.[3]

methyl 5-(but-3-en-1-yl)-2-hydroxy-3-methoxybenzoate (4g)

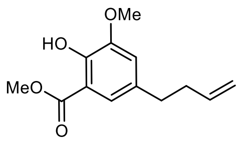

A 5 mL round-bottom flask was charged with a magnetic stirrer bar, 1CTR **1a** (250 mg, 1 mmol, 5.00 equiv) and methyl 5-allyl-3-methoxysalicylate (44.5 mg, 0.2 mmol, 1.00 equiv). The round-bottom flask was fitted to a reflux condenser (Teflon tape around the ground glass joint, suba-seal on condenser outlet, connected to a N2 line with a needle through the suba-seal) and evacuate-refilled with N_2 (3 cycles). The flask was then heated to 40 °C in an oil bath and dichloromethane (0.25 mL) was added through the top of the condenser to dissolve the starting materials. A stock solution of HG-II (7.6 mg/mL, 0.06 equiv) was prepared in dichloromethane and 1 mL of the stock solution was added, followed by dichloromethane (0.25 mL). The reaction was heated and stirred for 6 h, after which additional HG-II catalyst (0.5 mL, 7.6 mg/mL, 0.03 equiv) and 1CTR **1a** (125 mg, 0.5 mmol, 2.50 equiv) were added. The reaction was stirred for a further 12 hours, followed by retro-ene condition A (3.2). The residue was purified by flash column chromatography on $AgNO_3$(~10 wt%)-impregnated silica gel (10-25% Et_2O in 40-60 PE) to give the product as a colourless oil (35.9 mg, 0.152 mmol, 76%).

^{1}H NMR (500 MHz, Chloroform-*d*) δ 10.85 (app. d, *J* = 0.6 Hz, 1H), 7.27 (d, *J* = 2.0 Hz, 1H), 6.90 (d, *J* = 2.1 Hz, 1H), 5.86 (ddt, *J* = 16.9, 10.2, 6.6 Hz, 1H), 5.12 – 4.91 (m, 2H), 3.97 (s, 3H), 3.92 (s, 3H), 2.67 (dd, *J* = 8.8, 6.7 Hz, 2H), 2.42 – 2.33 (m, 2H).

^{13}C NMR (126 MHz, Chloroform-*d*) δ 170.9, 150.3, 148.3, 137.7, 132.1, 120.1, 117.3, 115.2, 112.1, 56.2, 52.3, 35.5, 34.9.

HRMS (ESI+) m/z calculated for $C_{13}H_{17}O_4$ $[M+H]^+$ 237.11214, found 237.11215 (Δ = 0.04 ppm)

IR (film, cm^{-1}): 3096, 3074, 3040, 2937, 2863, 1609, 1500, 1459, 1033, 760.

5-(but-3-en-1-yl)-2-hydroxy-N-(2-hydroxyethyl)-3-methoxybenzamide (4h)

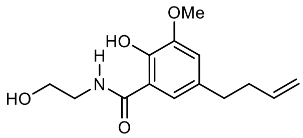

Prepared according to the general procedure for homologation of monosubstituted alkenes (3.2) from alibendol (50.3 mg, 0.2 mmol, 1.00 equiv), with the exception that 1CTR **1a** (250 mg, 1.00 mmol, 5.00 equiv) and HG-II stock solution (12.6 mg/mL, 0.10 equiv) were used. The reaction was stirred for 16 h, followed by retro-ene condition A. The residue was purified by flash column chromatography on silica gel (0-5% MeOH in DCM) to give the product as a brown solid (34.7 mg, 0.131 mmol, 65%).

^{1}H NMR (400 MHz, Chloroform-*d*) δ 11.12 (s, 1H), 7.12 (br.s, 1H), 6.99 – 6.94 (m, 1H), 6.87 – 6.82 (m, 1H), 5.93 – 5.78 (m, 1H), 5.11 – 5.01 (m, 2H), 3.92 (s, 3H), 3.90 – 3.83 (m, 2H), 3.66 (dt, J = 5.7, 4.3 Hz, 2H), 2.71 – 2.62 (m, 2H), 2.42 – 2.32 (m, 3H).

^{13}C NMR (126 MHz, Chloroform-*d*) δ 169.9, 148.5, 148.5, 137.7, 132.1, 117.5, 115.3, 115.3, 114.7, 62.1, 56.2, 42.3, 35.6, 35.1.

HRMS (ESI+) m/z calculated for $C_{14}H_{20}NO_4$ $[M+H]^+$ 266.13868, found 266.13880 (Δ = 0.5 ppm)

IR (film, cm^{-1}): 3369, 3076, 2980, 2970, 2929, 1639, 1592, 1544, 1463, 1267, 1058

(3,5-dibromo-4-(hept-6-en-1-yloxy)phenyl)(2-ethylbenzofuran-3-yl)methanone (4i)

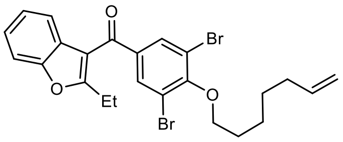

Prepared according to the general procedure for homologation of monosubstituted alkenes (3.2) from (3,5-dibromo-4-(hex-5-en-1-yloxy)phenyl)(2-ethylbenzofuran-3-yl)methanone (98.0 mg, 0.19 mmol, 1.00 equiv), with the exception that 1CTR **1a** (250 mg, 1 mmol, 5.00 equiv) was used. The reaction was stirred for 16 h.

Owing to the incomplete consumption of starting material in the cross-metathesis reaction and inseparability of the homolog from the starting olefin, the latent homolog was separated from the starting olefin by flash column chromatography on silica gel (10-20% EtOAc in PE). The fractions containing the latent homolog and dimer of the homologating reagent, which co-eluted, were concentrated into a 10 mL microwave vial containing a magnetic stir bar. The vial was sealed using a crimper and evacuate-refilled with N_2 (3 cycles). Dichloromethane (1 mL) was added via a syringe and the vial was heated to 40 °C, followed by retro-ene condition A. The residue was purified by flash column chromatography on silica gel (0-20% EtOAc in 40-60 PE) to give the product as a colourless oil (69.3 mg, 0.134 mmol, 69%).

^{1}H NMR (500 MHz, Chloroform-*d*) δ 8.00 (s, 2H), 7.52 (d, *J* = 8.2 Hz, 1H), 7.44 (d, *J* = 7.8 Hz, 1H), 7.34 (t, *J* = 7.7 Hz, 1H), 7.29 – 7.23 (m, 1H), 5.87 (ddt, *J* = 17.0, 10.2, 6.7 Hz, 1H), 5.12 – 4.93 (m, 2H), 4.13 (t, *J* = 6.5 Hz, 2H), 2.92 (q, *J* = 7.6 Hz, 2H), 2.18 – 2.10 (m, 2H), 2.00 – 1.90 (m, 2H), 1.65 – 1.58 (m, 2H), 1.56 – 1.49 (m, 2H), 1.38 (t, *J* = 7.5 Hz, 3H).
^{13}C NMR (126 MHz, Chloroform-*d*) δ 188.2, 166.9, 157.1, 153.7, 138.8, 137.0, 133.6, 126.5, 124.7, 123.9, 121.1, 118.7, 115.4, 114.5, 111.2, 73.9, 33.7, 29.9, 28.7, 25.3, 22.0, 12.2.
HRMS (ESI+) m/z calculated for $C_{24}H_{25}O_3Br_2$ $[M+H]^+$ 519.01650, found 519.01647 (Δ = -0.1 ppm)
IR (film, cm^{-1}): 3072, 2974, 2933, 2878, 2855, 1647, 1452, 1376, 1255, 1238, 1174, 953, 911, 746.

N-(but-3-en-1-yl)-4-(N,N-dipropylsulfamoyl)benzamide (4j)

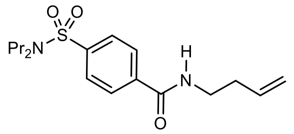

Prepared according to the general procedure for homologation of monosubstituted alkenes (3.2) from *N*-allyl-4-(*N*,*N*-dipropylsulfamoyl)benzamide (64.8 mg, 0.2 mmol, 1.00 equiv), with the exception that additional HG-II catalyst solution (0.5 mL, 12.6 mg/mL, 0.05 equiv) was added after 6 h. The reaction was stirred for a further 12 h, followed by retro-ene condition A. The residue was purified by C18 reverse column chromatography (0-100% Water-Acetonitrile). Product-containing fractions were lyophilized to give the product as a white powder (39.0 mg, 0.115 mmol, 58%).

^{1}H NMR (400 MHz, Chloroform-*d*) δ 7.88 – 7.81 (m, 4H), 6.38 (s, 1H), 5.91 – 5.78 (m, 1H), 5.24 – 5.09 (m, 2H), 3.62 – 3.50 (m, 2H), 3.10 (t, 4H), 2.47 – 2.37 (m, 2H), 1.56 (h, *J* = 7.5 Hz, 4H), 0.88 (t, *J* = 7.4 Hz, 6H).
^{13}C NMR (101 MHz, Chloroform-*d*) δ 166.2, 142.8, 138.3, 135.1, 127.6, 127.2, 117.6, 50.0, 39.0, 33.6, 21.9, 11.2.
HRMS (ESI+) m/z calculated for $C_{17}H_{27}N_2O_3S$ $[M+H]^+$ 339.17369, found 339.17443 (Δ = 2.2 ppm)
IR (film, cm^{-1}): 3290, 2083, 2964, 2932, 2873, 1632, 1545, 1342, 1147, 603, 557.

hex-5-en-1-yl 2-(4-(2,2-dichlorocyclopropyl)phenoxy)-2-methylpropanoate (4k)

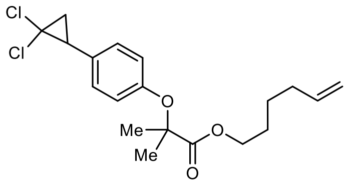

Prepared according to the general procedure for homologation of monosubstituted alkenes (3.2) from pent-4-en-1-yl 2-(4-(2,2-dichlorocyclopropyl)phenoxy)-2-methylpropanoate (71.2 mg, 0.2 mmol, 1.00 equiv), with the exception that 1CTR **1a** (250 mg, 1 mmol, 5.00 equiv) was used. The reaction was stirred for 16 h.
Owing to the incomplete consumption of starting material in the cross-metathesis reaction and the inseparability of the homolog from the starting olefin, the latent homolog was separated from the starting olefin by flash column chromatography on silica gel (10-20% EtOAc in PE). The fractions containing the latent homolog and dimer of the homologating reagent, which co-eluted, were concentrated into a 10 mL microwave vial containing a magnetic stir bar. The vial was sealed using a crimper and evacuate-refilled with N_2 (3 cycles). Dichloromethane (1 mL) was added via a syringe and the vial was heated to 40 °C. Citric acid (576 mg, 3 mmol, 3.00 equiv) and TBAF·3H_2O (394 mg, 1 mmol, 1.25 equiv) were dissolved in 2 mL MeOH. The TBAF-citric acid solution was added through the septum of the microwave vial and the reaction was heated at 40 °C in an oil bath for 16 h, followed by the standard work-up procedure.

The residue was purified by flash column chromatography on silica gel (0-15% EtOAc in 40-60 PE) to give the product as a colourless oil (49.0 mg, 0.132 mmol, 66%).

^{1}H NMR (400 MHz, Chloroform-*d*) δ 7.13 (d, *J* = 8.7 Hz, 2H), 6.83 (d, *J* = 8.7 Hz, 2H), 5.75 (ddt, *J* = 17.0, 6.6 Hz, 1H), 5.05 – 4.92 (m, 2H), 4.18 (t, *J* = 6.6 Hz, 2H), 2.85 (dd, *J* = 10.7, 8.3 Hz, 1H), 2.09 – 2.00 (m, 2H), 1.96 (dd, *J* = 10.7, 7.4 Hz, 1H), 1.79 (dd, *J* = 8.4, 7.3 Hz, 1H), 1.63 (s 6H + m 2H), 1.36 (p, *J* = 7.8 Hz, 2H).

^{13}C NMR (101 MHz, Chloroform-*d*) δ 174.3, 155.0, 138.2, 129.6, 128.0, 118.5, 114.9, 79.2, 65.4, 60.9, 34.8, 33.1, 27.8, 25.8, 25.4, 25.0.

HRMS (ESI+) m/z calculated for $C_{19}H_{25}O_3Cl_2$ $[M+H]^+$ 371.11753, found 371.11721 (Δ = 0.9 ppm)

IR (film, cm^{-1}): 2988, 2938, 2861, 1730, 1511, 1284, 1242, 1175, 1136, 1116, 760.

5-(2-methylpent-4-en-1-yl)benzo[d][1,3]dioxole (4l)

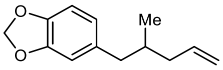

Prepared according to the general procedure for homologation of monosubstituted alkenes (3.2) from 5-(2-methylbut-3-en-1-yl)benzo[d][1,3]dioxole (38.0 mg, 0.2 mmol, 1.00 equiv). The reaction was stirred for 16 h, followed by retro-ene condition B. The residue was purified by flash column chromatography on silica gel (100% pentane) to give the product as a colourless oil (30.5 mg, 0.149 mmol, 75%).

^{1}H NMR (400 MHz, $CDCl_3$) δ 6.72 (d, J = 7.9 Hz, 1H), 6.65 (d, J = 1.8 Hz, 1H), 6.62 – 6.57 (m, 1H), 5.92 (s, 2H), 5.88 – 5.72 (m, 1H), 5.07 – 4.96 (m, 2H), 2.57 (dd, J = 13.5, 6.3 Hz, 1H), 2.30 (dd, J = 13.5, 8.1 Hz, 1H), 2.14 – 2.06 (m, 1H), 1.96 – 1.84 (m, 1H), 1.84 – 1.67 (m, 1H), 0.86 (d, J = 6.6 Hz, 3H).

^{13}C NMR (101 MHz, Chloroform-*d*) 147.4, 145.5, 137.3, 135.1, 121.9, 115.9, 109.5, 107.9, 100.7, 42.8, 40.8, 35.1, 19.1.

HRMS (ESI+) m/z calculated for $C_{13}H_{17}O_2$ $[M+H]^+$ 205.1223, found 205.1222 (Δ = - 0.5 ppm)

IR (film, cm) 3075, 2915, 1502, 1488, 1439, 1246, 1188

methyl (S)-2-((((9H-fluoren-9-yl)methoxy)carbonyl)amino)-2-methyloct-7-enoate (4m)

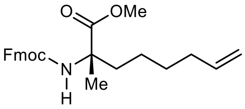

Prepared according to the general procedure for homologation of monosubstituted alkenes (3.2) from methyl (S)-2-((((9H-fluoren-9-yl)methoxy)carbonyl)amino)-2-methylhept-6-enoate (78.7 mg, 0.2 mmol, 1.00 equiv), with the exception that 1CTR **1a** (250 mg, 1 mmol, 5.00 equiv) was used. The reaction was stirred for 16 h, followed by retro-ene condition A. The residue was purified by C18 reverse column chromatography (0-100% Water-Acetonitrile). Product-containing fractions were lyophilized to give the product as a colourless oil (63.5 mg, 0.156 mmol, 78%).

^{1}H NMR (400 MHz, Chloroform-*d*) δ 7.80 (d, J = 7.3 Hz, 2H), 7.63 (d, J = 7.5 Hz, 2H), 7.43 (t, J = 7.3 Hz, 2H), 7.38 – 7.31 (m, 2H), 5.86 – 5.72 (m, 1H), 5.68 (s, 1H), 5.13 – 4.88 (m, 2H), 4.41 (s, 2H), 4.26 (t, J = 6.8 Hz, 1H), 3.79 (s, 3H), 2.20 (s, 1H), 2.10 – 2.00 (m, 2H), 1.82 (s, 1H), 1.62 (s, 3H), 1.47 – 1.24 (m, 3H), 1.11 (s, 1H).

^{13}C NMR (176 MHz, Chloroform-*d*) δ 174.9, 154.4, 144.0, 144.0, 141.4, 138.6, 127.7, 127.1, 127.1, 125.1, 120.0, 120.0, 114.6, 66.4, 60.1, 52.7, 47.3, 36.6, 33.4, 28.6, 23.5, 23.4.

HRMS (ESI+) m/z calculated for $C_{25}H_{30}NO_4$ 408.21693, found 408.21764 (Δ = 1.7 ppm)

IR (film, cm^{-1}) 33354, 2980, 2944, 2859, 1720, 1502, 1449, 1241, 1080, 758, 738

$[\alpha]_D^{20}$ = + 9.0° (c = 0.2, $CDCl_3$)

(*R*)-2-(1-hydroxypent-4-en-2-yl)isoindoline-1,3-dione (4n)

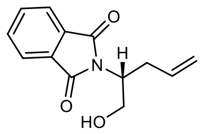

Prepared according to the general procedure for homologation of monosubstituted alkenes (3.2) from (*R*)-2-Phthalimido-3-buten-1-ol (43.4 mg, 0.2 mmol, 1.00 equiv), with the exception that 1CTR **1a** (250 mg, 1 mmol, 5.00 equiv) was used. The reaction was stirred for 16 h.
Owing to the incomplete consumption of starting material in the cross-metathesis reaction and the inseparability of the homolog from the starting olefin, the latent homolog was separated from the starting olefin by flash column chromatography on silica gel (40% EtOAc in PE). The fractions containing the latent homolog were concentrated into a 10 mL microwave vial containing a magnetic stir bar. The vial was sealed using a crimper and evacuate-refilled with N_2 (3 cycles). Dichloromethane (1 mL) was added via a syringe and the vial was heated to 40 °C. Citric acid (115.3 mg, 0.6 mmol, 3.00 equiv) and TBAF·$3H_2O$ (78.9 mg, 0.25 mmol, 1.25 equiv) were dissolved in 1 mL MeOH. The TBAF-citric acid solution was added through the septum of the microwave vial and the reaction was heated at 40 °C in an oil bath for 16 h, followed by the standard work-up procedure.

The residue was purified by flash column chromatography on silica gel (40% EtOAc in 40-60 PE) to give the product as a brown oil (24.4 mg, 0.106 mmol, 53%).

^{1}H NMR (400 MHz, Chloroform-*d*) δ 7.91 – 7.81 (m, 2H), 7.79 – 7.68 (m, 2H), 5.85 – 5.72 (m, 1H), 5.14 – 4.98 (m, 1H), 4.53 – 4.42 (m, 1H), 4.16 – 4.04 (m, 1H), 4.04 – 3.91 (m, 1H), 2.79 – 2.67 (m, 2H), 2.64 – 2.55 (m, 1H).
^{13}C NMR (101 MHz, Chloroform-*d*) δ 169.0, 134.1, 133.8, 131.7, 123.4, 118.3, 63.2, 53.5, 33.2.
HRMS (ESI+) m/z calculated for $C_{13}H_{14}NO_3$ $[M+H]^+$ 232.09682, found 232.09718 (Δ = 1.6 ppm).
IR (film, cm^{-1}): 3461, 3078, 2941, 1771, 1694, 1391, 1366, 717.
$[\alpha]_D^{20}$ = +5.0 (*c* = 0.2, $CDCl_3$)

tert-butyl (*R*)-(1-phenylpent-4-en-2-yl)carbamate (4o)

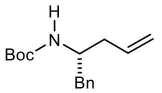

Prepared according to the general procedure for homologation of monosubstituted alkenes (3.2) from tert-butyl (*S*)-(1-phenylbut-3-en-2-yl)carbamate (49.4 mg, 0.2 mmol, 1.00 equiv). The reaction was stirred for 16 h, followed by retro-ene condition B. The residue was purified by flash column chromatography on silica gel (5-10% EtOAc in PE) to give the product as a pale-yellow solid (44.1 mg, 0.168 mmol, 84%).

^{1}H NMR (500 MHz, $CDCl_3$) δ 7.38 – 7.26 (m, 2H), 7.25 – 7.12 (m, 3H), 5.80 (ddt, *J* = 17.5, 10.5, 7.2 Hz, 1H), 5.14 – 5.05 (m, 2H), 4.40 (s, 1H), 3.91 (s, 1H), 2.83 – 2.71 (m, 2H), 2.31 – 2.21 (m, 1H), 2.16 – 2.06 (m, 1H), 1.41 (s, 9H).
^{13}C NMR (126 MHz, Chloroform-*d*) δ 155.3, 138.1, 134.4, 129.5, 128.4, 126.3, 117.9, 79.1, 51.0, 40.5, 38.1, 28.4.
HRMS (ESI+) m/z calculated for $C_{16}H_{23}NO_2Na$ $[M+Na]^+$ 284.1621, found 284.1612 (Δ = -3.0 ppm)
IR (film, cm) 3065, 3028, 2982, 2927, 1681, 1528, 1496

(1S*, 2S*)-1-(4-methoxyphenyl)-2-methylpent-4-en-1-ol (4p)

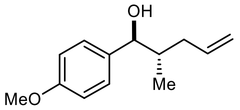

Prepared according to the general procedure for homologation of monosubstituted alkenes (3.2) from (1S*, 2S*)-1-(4-methoxyphenyl)-2-methylbut-3-en-1-ol (38.4 mg, 0.2 mmol, 1.00 equiv), with the exception that 1CTR **1a** (250 mg, 1 mmol, 5.00 equiv) and HG-II stock solution (12.6 mg/mL, 0.10 equiv) were used. The reaction was stirred for 16 h, followed by retro-ene condition B.† The residue was purified by flash column chromatography on silica gel (5-10% EtOAc in PE) to give the product as a colourless oil (32.0 mg, 0.155 mmol, 78%).

† Higher loading of citric acid and TBAF·$3H_2O$ is required to compensate for the higher equivalents of 1CTR **1a**. Citric acid (576 mg, 3 mmol, 3.00 equiv) and TBAF·$3H_2O$ (394 mg, 1 mmol, 1.25 equiv) were dissolved in 2 mL MeOH.

^{1}H NMR (500 MHz, $CDCl_3$) δ 7.26 (d, *J* = 8.9 Hz, 2H), 6.90 (d, *J* = 8.8 Hz, 2H), 5.87 (ddt, *J* = 16.8, 10.1, 6.5 Hz, 1H), 5.13 – 5.01 (m, 2H), 4.41 (d, *J* = 7.4 Hz, 1H), 3.83 (s, 3H), 2.50 – 2.40 (m, 1H), 2.10 – 1.96 (m, 1H), 1.96 – 1.89 (m, 1H), 1.89 – 1.83 (m, 1H), 0.75 (d, *J* = 6.8 Hz, 3H).
^{13}C NMR (126 MHz, Chloroform-*d*) δ 159.0, 137.3, 135.5, 127.9, 116.2, 113.6, 78.3, 55.3, 40.0, 37.2, 15.7.
HRMS (ESI+) m/z calculated for $C_{13}H_{19}O_2$ $[M+H]^+$ 207.1380, found 207.1373 (Δ = -3.2 ppm)
IR (film, cm) 3397, 3074, 2959, 2906, 2836, 1610, 1510, 1457, 1375.

4-allyl-1-(benzyloxy)-2-methoxybenzene (4q)

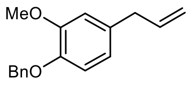

Prepared according to the general procedure for homologation of monosubstituted alkenes (3.2) from 1-(benzyloxy)-2-methoxy-4-vinylbenzene (24.0 mg, 0.1 mmol, 1.00 equiv), with the exception that 1CTR **1a** (125 mg, 0.5 mmol, 5.00 equiv) and HG-II stock solution (0.5 mL, 6.3 mg/mL, 0.05 equiv) were used. The reaction was stirred for 6 h, after which 2-3 drops of butyl vinyl ether were added to quench the catalyst. The residue was concentrated into a 10 mL microwave vial containing a magnetic stirrer bar. The vial was sealed using a crimper and evacuate-refilled with N_2 (3 cycles). A 3:1 mixture of THF:H_2O (800 uL) was added *via* a syringe, and the vial was heated to 90 °C. A 1:1 mixture of TFA:THF (250 uL) was added, and the reaction was stirred for 16 h.

The reaction mixture was transferred to a separatory funnel containing $NaHCO_3$ (approx. 50 mL). The aqueous phase was extracted 3x using Et_2O. The combined organic phases were dried over $MgSO_4$, filtered and concentrated *in vacuo*. The residue was purified by flash column chromatography on silica gel (5-10% Et_2O in PE) to give the product as a colourless oil (16.0 mg, 0.063 mmol, 63%).

^{1}H NMR (400 MHz, $CDCl_3$) δ 7.48 – 7.41 (m, 2H), 7.39 – 7.33 (m, 2H), 7.32 – 7.27 (m, 1H), 6.82 (d, *J* = 8.1 Hz, 1H), 6.74 (d, *J* = 2.0 Hz, 1H), 6.66 (dd, *J* = 8.1, 2.0 Hz, 1H), 6.03 – 5.88 (m, 1H), 5.13 (s, 2H), 5.11 – 5.02 (m, 2H), 3.88 (s, 3H), 3.33 (d, *J* = 6.8 Hz, 2H).

^{13}C NMR (101 MHz, $CDCl_3$) δ 149.6, 146.5, 137.6, 137.4, 133.3, 128.5, 127.7, 127.3, 120.4, 115.6, 114.3, 112.4, 71.2, 56.0, 39.8.

4q is a known compound and its NMR spectra are in accord with published data.[4]

1-allyl-3-bromo-5-(trifluoromethyl)benzene (4r)

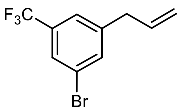

Prepared according to the general procedure for homologation of monosubstituted alkenes (3.2) from 1-bromo-3-(trifluoromethyl)-5-vinylbenzene (25.0 mg, 0.1 mmol, 1.00 equiv), with the exception that 1CTR **1a** (125 mg, 0.5 mmol, 5.00 equiv) and HG-II stock solution (0.5 mL, 6.3 mg/mL, 0.05 equiv) were used. The reaction was stirred for 6 h, after which 2-3 drops of butyl vinyl ether were added to quench the catalyst. The residue was concentrated into a 10 mL microwave vial containing a magnetic stirrer bar. The vial was sealed using a crimper and evacuate-refilled with N_2 (3 cycles). A 3:1 mixture of THF:H_2O (800 uL) was added *via* a syringe, and the vial was heated to 90 °C. A 1:1 mixture of TFA:THF (250 uL) was added, and the reaction was stirred for 16 h.
The reaction mixture was transferred to a separatory funnel containing $NaHCO_3$ (approx. 50 mL). The aqueous phase was extracted 3x using Et_2O. The combined organic phases were dried over $MgSO_4$, filtered and concentrated *in vacuo*.

The product was too volatile to isolate via column chromatography. The assay yield was calculated to be 63% via quantitative ^{1}H NMR relative to methyl benzoate (12.5 μL) a*s an internal standard.*

1-allyl-4-nitrobenzene (4s)

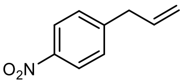

Prepared according to the general procedure for homologation of monosubstituted alkenes (3.2) from 1-nitro-4-vinylbenzene (14.9 mg, 0.1 mmol, 1.00 equiv), with the exception that 1CTR **1a** (125 mg, 0.5 mmol, 5.00 equiv) and HG-II stock solution (0.5 mL, 6.3 mg/mL, 0.05 equiv) were used. The reaction was stirred for 6 h, after which 2-3 drops of butyl vinyl ether were added to quench the catalyst. The residue was concentrated into a 10 mL microwave vial containing a magnetic stirrer bar. The vial was sealed using a crimper and evacuate-refilled with N_2 (3 cycles). A 3:1 mixture of THF:H_2O (800 uL) was added *via* a syringe, and the vial was heated to 90 °C. A 1:1 mixture of TFA:THF (250 uL) was added, and the reaction was stirred for 16 h.
The reaction mixture was transferred to a separatory funnel containing $NaHCO_3$ (approx. 50 mL). The aqueous phase was extracted 3x using Et_2O. The combined organic phases were dried over $MgSO_4$, filtered and concentrated *in vacuo*.

The product was too volatile to isolate via column chromatography. The assay yield was calculated to be 47% via quantitative ^{1}H NMR relative to methyl benzoate (12.5 μL) a*s an internal standard.*

***N*-benzyl-*N*-(but-3-en-1-yl)-4-ethoxy-3-(1-methyl-7-oxo-3-propyl-6,7-dihydro-1H-pyrazolo[4,3-d]pyrimidin-5-yl)benzenesulfonamide (4t)**

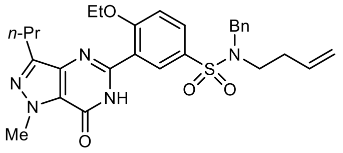

Prepared according to the general procedure for homologation of monosubstituted alkenes (3.2) from *N*-allyl-*N*-benzyl-4-ethoxy-3-(1-methyl-7-oxo-3-propyl-6,7-dihydro-1H-pyrazolo[4,3-d]pyrimidin-5-yl)benzenesulfonamide (52.1 mg, 0.1 mmol, 1.00 equiv), with the exception that 1CTR **1a** (125 mg, 0.5 mmol, 5.00 equiv) and HG-II stock solution (0.5 mL, 6.3 mg/mL, 0.05 equiv) were used. The reaction was stirred for 16 h, followed by retro-ene condition A. The residue was purified by C18 reverse column chromatography (0-100% Water-Acetonitrile). Product-containing fractions were lyophilized to give the product as an off-white white solid (37.0 mg, 0.069 mmol, 69%).

^{1}H NMR (400 MHz, $CDCl_3$) δ 10.86 (s, 1H), 8.95 – 8.89 (m, 1H), 7.91 (dd, *J* = 8.8, 2.5 Hz, 1H), 7.35 – 7.22 (m, 5H), 7.13 (d, *J* = 8.8 Hz, 1H), 5.58 (ddt, *J* = 17.3, 10.6, 6.8 Hz, 1H), 4.97 – 4.86 (m, 2H), 4.44 – 4.33 (m, 4H), 4.27 (s, 3H), 3.28 – 3.19 (m, 2H), 2.96 – 2.87 (m, 2H), 2.20 – 2.09 (m, 2H), 1.85 (h, *J* = 7.4 Hz, 2H), 1.64 (t, *J* = 7.0 Hz, 3H), 0.99 (t, *J* = 7.4 Hz, 3H).
^{13}C NMR (101 MHz, $CDCl_3$) δ 159.0, 153.6, 147.0, 146.5, 138.4, 136.1, 134.5, 133.6, 131.2, 130.5, 128.6, 128.3, 127.9, 124.5, 121.1, 117.1, 113.0, 66.0, 52.0, 47.5, 38.2, 32.7, 27.7, 22.3, 14.5, 14.0.
HRMS (ESI+) m/z calculated for $C_{28}H_{34}N_5O_4S$ $[M+H]^+$ 536.2332, found 536.2350 (Δ = 3.4 ppm)
IR (film, cm) 3304, 3077, 2932, 2871, 1697, 1335, 1153, 1027, 921.

1-cyclopropyl-6-fluoro-8-methoxy-7-((4aS,7aS)-1-(3-methylhex-5-enoyl)octahydro-6H-pyrrolo[3,4-b]pyridin-6-yl)-4-oxo-1,4-dihydroquinoline-3-carboxylic acid (4u)

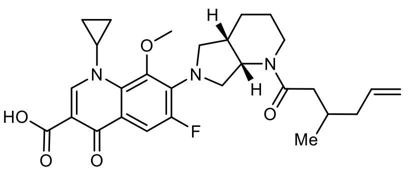

Prepared according to the general procedure for homologation of monosubstituted alkenes (3.2) from 1-cyclopropyl-6-fluoro-8-methoxy-7-((4aS,7aS)-1-(3-methylpent-4-enoyl)octahydro-6H-pyrrolo[3,4-b]pyridin-6-yl)-4-oxo-1,4-dihydroquinoline-3-carboxylic acid (49.7 mg, 0.1 mmol, 1.00 equiv), with the exception that 1CTR **1a** (125 mg, 0.5 mmol, 5.00 equiv) and HG-II stock solution (12.6 mg/mL, 0.10 equiv) were used. The reaction was stirred for 16 h. The residue was concentrated into a 10 mL microwave vial containing a magnetic stirrer bar. The vial was sealed using a crimper and evacuate-refilled with N_2 (3 cycles). A 3:1 mixture of THF:H_2O (800 uL) was added *via* a syringe, and the vial was heated to 70 °C. A 1:1 mixture of TFA:THF (250 uL) was added, and the reaction was stirred for 16 h.

The crude reaction was transferred to a stirred round bottom flask containing EtOAc (10 mL) and H_2O (25 mL). The solution was neutralized to pH 8 using aqueous ammonia. The reaction mixture was transferred to a separatory funnel. The aqueous phase was extracted 3x using EtOAc. The combined organic phases were dried over $MgSO_4$, filtered and concentrated *in vacuo.* The residue was purified by preparative HPLC using a Shimadzu Nexera Prep with a reversed-phase Phenomenex Luna 5 µm C18(2) 100 Å, LC Column (150 x 10 mm), eluting with a gradient of 5-100% solvent A-Solvent B over 20 minutes (solvent A: 0.05% (v/v) TFA in H2O, solvent B: 0.05% (v/v) TFA in MeCN). Product-containing fractions were lyophilized to give the product as a yellow powder (38.1 mg, 74.5 µmol, 75%).

4u exists as a ~7:3 mixture of rotamers in solution. Fatty acid derivatives of Moxifloxacin derivatives are reported to exist as rotameric mixtures.[5]

^{1}H NMR (500 MHz, $CDCl_3$) δ 14.99 (br. s, 1H), 8.76 (s, 1H), 7.77 (d, J = 2.5 Hz, 1H), 5.85 – 5.71 (m, 1H), 5.28 (q, J = 8.4 Hz, 0.7H), 5.07 – 4.96 (m, 2H), 4.72 – 4.62 (m, 0.3H), 4.60 – 4.50 (m, 0.3H), 4.17 – 4.04 (m, 1H), 4.03 – 3.94 (m, 1.3H), 3.87 – 3.75 (m, 1.4H), 3.60 (s, 0.7H), 3.57 (s, 2.3H), 3.54 – 3.45 (m, 0.7H), 3.41 – 3.35 (m, 0.3H), 3.34 – 3.27 (m, 0.3H), 3.24 (d, J = 10.6 Hz, 0.7H), 3.16 (t, J = 12.6 Hz, 0.7H), 2.72 (t, J = 13.2 Hz, 0.3H), 2.54 – 1.96 (m, 6H), 1.92 – 1.82 (m, 2H), 1.60 – 1.44 (m, 2H), 1.37 – 1.22 (m, 1H), 1.17 – 1.01 (m, 2H), 1.00 – 0.95 (m, 3H), 0.87 – 0.77 (m, 1H).
^{13}C NMR (126 MHz, $CDCl_3$) δ 176.7, 172.9, 172.8, 172.2, 167.1, 167.0, 153.8 (d, $^1J_{C-F}$ = 252 Hz), 153.5 (d, $^1J_{C-F}$ = 252 Hz), 149.8, 149.7, 141.2 (d, $^3J_{C-F}$ = 7.0 Hz), 137.2 (d, $^2J_{C-F}$ = 10.9 Hz), 136.7, 136.6, 134.4, 118.8 (d, $^3J_{C-F}$ 8.8 Hz), 116.6, 116.5, 108.0, 107.8, 107.6, 61.2, 56.5 (d, $^4J_{C-F}$ = 6.4 Hz), 54.5, 50.2, 49.2, 48.0 (d, $^4J_{C-F}$ = 6.5 Hz), 41.5, 41.5, 41.3, 41.2, 40.5, 40.3, 40.2, 37.1, 36.2, 35.5, 30.3, 30.0, 25.5, 25.1, 24.8, 24.7, 23.9, 19.9, 19.8, 10.5, 8.5.
^{19}F NMR (471 MHz, $CDCl_3$) δ -120.93 (d, J = 5.5 Hz), -121.24 (d, J = 3.6 Hz).
IR (film, cm) 3303, 3077, 2932, 2863, 1724, 1698, 1617, 1508, 1433, 1029
HRMS (ESI+) m/z calculated for $C_{28}H_{35}N_3O_5F$ $[M+H]^+$ 512.2561, found 512.2565 (Δ = 0.8 ppm)

(1R,2R,4aS,8aS)-1-((R)-3-hydroxy-3-methylhex-5-en-1-yl)-2,5,5,8a-tetramethyldecahydronaphthalen-2-ol (5)

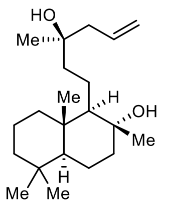

Prepared according to the general procedure for homologation of monosubstituted alkenes (3.2) from sclareol (61.7 mg, 0.2 mmol, 1.00 equiv), with the exception that 1CTR **1a** (250 mg, 1 mmol, 5.00 equiv) and HG-II stock solution (12.6 mg/mL, 0.10 equiv) were used.
Owing to the incomplete consumption of starting material in the cross-metathesis reaction and the inseparability of the homolog from the starting olefin, the latent homolog was separated from the starting olefin by flash column chromatography on silica gel (30-100% EtOAc in PE). The fractions containing the latent homolog were concentrated into a 10 mL microwave vial containing a magnetic stir bar. The vial was sealed using a crimper and evacuate-refilled with N_2 (3 cycles). Dichloromethane (1 mL) was added via a syringe and the vial was heated to 40 °C. Citric acid (115.3 mg, 0.6 mmol, 3.00 equiv) and TBAF·$3H_2O$ (78.9 mg, 0.25 mmol, 1.25 equiv) were dissolved in 1 mL MeOH. The TBAF-citric acid solution was added through the septum of the microwave vial and the reaction was heated at 40 °C in an oil bath for 16 h, followed by the standard work-up procedure.

The residue was purified by silica gel column chromatography (30-70% EtOAc in PE) to give the product as a brown solid (32.6 mg, 0.101 mmol, 51%).

^{1}H NMR (400 MHz, Chloroform-*d*) δ 5.90 (ddt, *J* = 16.9, 10.3 Hz, 1H), 5.18 – 5.07 (m, 2H), 2.24 (d, *J* = 7.5 Hz, 2H, br.s 2H), 1.90 – 1.81 (m, 1H), 1.72 – 1.48 (m, 6H), 1.39 (d, *J* = 13.7 Hz, 4H), 1.29 (d, *J* = 3.2 Hz, 1H), 1.20 (s, 3H), 1.19 (s, 3H), 1.16 (d, *J* = 4.2 Hz, 2H), 1.04 – 0.90 (m, 2H), 0.88 (s, 3H), 0.81 (s, 3H), 0.81 (s, 3H).
^{13}C NMR (126 MHz, Chloroform-*d*) δ 134.4, 118.3, 74.7, 72.8, 61.9, 56.1, 47.4, 44.6, 44.3, 42.0, 39.7, 39.2, 33.4, 33.2, 26.2, 24.3, 21.5, 20.5, 18.9, 18.4, 15.4.
HRMS (ESI+) m/z calculated for $C_{21}H_{38}O_2Na$ $[M+Na]^+$ 345.2770, found 345.2784 (Δ = 4.1 ppm)
IR (film, cm^{-1}): 3307, 2965, 2921, 2865, 2846, 1464, 1389, 1367, 995, 906
$[\alpha]_D^{20}$ = -2.5 (*c* = 0.2, $CDCl_3$)

(8R,9S,10R,13S,14S,17S)-17-(but-3-en-1-yl)-13-methyl-2,3,6,7,8,9,10,11,12,13,14,15,16,17-tetradecahydro-1H-cyclopenta[a]phenanthren-17-ol (6)

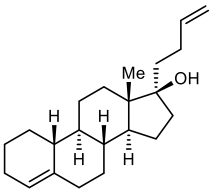

A 5 mL round-bottom flask was charged with a magnetic stirrer bar, 1CTR transfer reagent **1a** (250 mg, 1 mmol, 5.00 equiv) and allylestrenol (60.1 mg, 0.2 mmol, 1.00 equiv). The round-bottom flask was fitted to a reflux condenser (Teflon tape around the ground glass joint, suba-seal on condenser outlet, connected to a N_2 line with a needle through the suba-seal) and evacuate-refilled with N_2 (3 cycles). The flask was then heated to 40 °C in an oil bath and dichloromethane (0.25 mL) was added through the top of the condenser to dissolve the starting materials. A stock solution of HG-II (7.6 mg/mL, 0.06 equiv) was prepared in dichloromethane and 1 mL of the stock solution was added, followed by dichloromethane (0.25 mL). The reaction was heated and stirred for 4 h, after which additional HG-II catalyst (0.5 mL, 7.6 mg/mL, 0.03 equiv) and 1CTR **1a** (125 mg, 0.5 mmol, 2.50 equiv) were added. The reaction was stirred for a further 12 h, followed by retro-ene condition B (3.2).†

† Higher loading of citric acid and TBAF·$3H_2O$ is required to compensate for higher equivalents of 1CTR **1a**. Citric acid (865 mg, 3 mmol, 3.00 equiv) and TBAF·$3H_2O$ (591 mg, 1 mmol, 1.25 equiv) were dissolved in 3 mL MeOH.

The residue was purified by silica gel column chromatography (0-5% EtOAc in 40-60 PE) to give the product as a brown solid (39.4 mg, 0.125 mmol, 63%).

^{1}H NMR (500 MHz, Chloroform-*d*) δ 5.91 (td, *J* = 16.9, 6.7 Hz, 1H), 5.41 (s, 1H), 5.13 – 4.94 (m, 2H), 2.35 – 2.16 (m, 3H), 2.10 – 1.91 (m, 5H), 1.85 (dd, *J* = 13.4, 2.9 Hz, 1H), 1.82 – 1.74 (m, 1H), 1.70 (dd, *J* = 12.6, 2.7 Hz, 2H), 1.66 – 1.47 (m, 5H (residual H_2O)), 1.40 – 1.19 (m, 7H), 1.16 – 1.06 (m, 1H), 0.98 – 0.79 (s, 3H & m, 1H), 0.70 – 0.59 (m, 1H).
^{13}C NMR (101 MHz, Chloroform-*d*) δ 140.4, 139.6, 119.9, 114.3, 83.4, 50.3, 49.5, 46.7, 42.0, 41.9, 35.9, 35.5, 34.4, 31.9, 31.5, 28.8, 28.2, 26.0, 25.5, 23.6, 22.1, 14.4.
HRMS (ESI+) m/z calculated for $C_{22}H_{35}O$ $[M+H]^+$ 315.26824, found 315.26843 (Δ = 0.6 ppm)
IR (film, cm^{-1}): 3345, 2919, 2854, 1640, 1010, 909, 898.
$[\alpha]_D^{20}$ = +28.0 (*c* = 0.2, $CDCl_3$)

(3S,4aR,5S,6S,6aS,10S,10aR,10bS)-3-allyl-6,10,10b-trihydroxy-3,4a,7,7,10a-pentamethyl-1-oxododecahydro-1H-benzo[f]chromen-5-yl acetate (7)

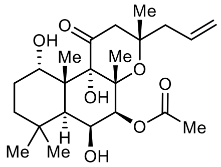

A 10 mL microwave vial was charged with a magnetic stirrer bar, 1CTR **1a** (125 mg, 0.5 mmol, 10.00 equiv) and forskolin (20.5 mg, 0.05 mmol, 1.00 equiv). The vial was sealed using a crimper and back-refilled with N_2 (3-cycles). A stock solution of HG-II catalyst (6.3 mg/mL, 0.20 equiv) was prepared in dichloroethane, 0.5 mL of the stock solution was injected through the septum of the vial using a syringe and the reaction was heated at 100 °C for 6 h. The reaction was concentrated *in vacuo* and the residue was purified by C18 reverse phase chromatography (0-100% Water-Acetonitrile) to remove unreacted starting material. Fractions containing latent homolog and forskolin were concentrated by lyophilisation (9.2 mg of Forskolin recovered).

The latent homolog was transferred to a 10 mL microwave vial fitted with a magnetic stir bar. The vial was sealed using a crimper and back-refilled with N_2 (3-cycles). DCE (0.5 mL) was added via a syringe and the reaction was heated to 40 °C. Citric acid (30 mg, 0.15 mmol) and TBAF·3H_2O (20 mg, 0.0625 mmol) were dissolved in 0.5 mL MeOH. The TBAF-citric acid solution was added through the septum of the microwave vial and the reaction was heated at 40 °C in an oil bath for 16 h, followed by the standard work-up procedure (3.2).

The residue was purified by C18 reverse column chromatography (0-100% Water-Acetonitrile). Homolog containing fractions were lyophilized to give the product as a white solid (5.5 mg, 12.9 µmol, 26%, 47% based on recovered Forskolin).

^{1}H NMR (700 MHz, Chloroform-*d*) δ 6.16 (s, 1H), 5.96 (ddt, *J* = 17.3, 10.2, 7.2 Hz, 1H), 5.46 (d, *J* = 4.2 Hz, 1H), 5.20 – 5.04 (m, 2H), 4.56 – 4.40 (m, 2H), 3.30 (d, *J* = 16.0 Hz, 1H), 2.80 (d, *J* = 2.8 Hz, 1H), 2.38 – 2.31 (m, 2H), 2.27 – 2.21 (m, 3H), 2.19 (s, 3H), 1.81 – 1.74 (m, 2H), 1.67 (s, 3H), 1.49 (s, 3H), 1.47 – 1.41 (m, 1H), 1.29 (s, 3H), 1.28 (s, 3H), 1.16 – 1.11 (m, 1H), 1.06 (s, 3H).
^{13}C NMR (176 MHz, Chloroform-*d*) δ 207.3, 169.6, 134.3, 118.8, 82.7, 81.6, 76.5, 76.0, 74.4, 70.0, 49.8, 48.2, 43.0, 42.9, 36.1, 34.4, 32.9, 30.6, 26.8, 24.3, 23.5, 21.2, 19.8.
HRMS (ESI+) m/z calculated for $C_{23}H_{36}O_7Na$ $[M+Na]^+$ 447.23532, found 447.23600 (Δ = 1.5 ppm)
IR (film, cm^{-1}): 3430, 3291, 3075, 3005, 2951, 2919, 2855, 1715, 1698, 1376, 1264, 1064, 1039
$[\alpha]_D^{20}$ = +39.0 (*c* = 0.2, $CDCl_3$)

Fig. S3. Temporary protection of quinine as its ammonium salt

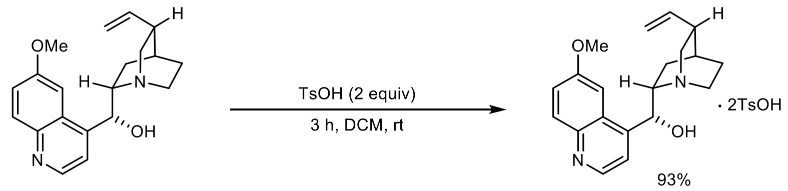

A 50 mL round-bottom flask fitted with magnetic stirrer bar was charged with quinine (1.5 g, 4.62 mmol, 1.00 equiv) and TsOH (1.59 g, 9.24 mmol, 2.00 equiv) which had been dried under high-vacuum at 100 °C for 4 h. Dichloromethane (20 mL) was added to the round-bottom flask and the reaction was stirred at room temperature for 3 h. The resulting white precipitate was filtered and washed with cold dichloromethane (2 x 10 mL). The precipitate was dried under vacuum to give the product as a yellow crystalline solid (2.83 g, 4.31 mmol, 93%).

(*R*)-((1S,2S,4S,5R)-5-allylquinuclidin-2-yl)(6-methoxyquinolin-4-yl)methanol (8)

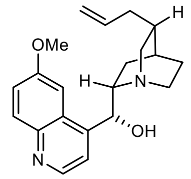

A 5 mL round-bottom flask was charged with a magnetic stirrer bar, 1CTR **1a** (125 mg, 0.5 mmol, 5.00 equiv) and quinine ditosylate (66.8 mg, 0.1 mmol, 1.00 equiv). The round-bottom flask was fitted to a reflux condenser (Teflon tape around the ground glass joint, suba-seal on condenser outlet, connected to a N_2 line with a needle through the suba-seal) and evacuate-refilled with N_2 (3 cycles). The flask was then heated to 40 °C in an oil bath and dichloromethane (0.5 mL) was added through the top of the condenser. A stock solution of HG-II (6.3 mg/mL, 0.05 equiv) was prepared in dichloromethane and 0.5 mL of the stock solution was added, followed by dichloromethane (0.25 mL). The reaction was heated and stirred for 4 h, after which additional HG-II catalyst (0.25 mL, 6.3 mg/mL, 0.025 equiv) and 1CTR reagent (63 mg, 0.25 mmol, 2.50 equiv) were added. The reaction was stirred for a further 12 h, followed by retro-ene condition A (3.2). The residue was purified by column chromatography on silica gel (20-30% {1.75 M NH_3 in MeOH} in DCM) to give the product as a brown solid (25.3 mg, 0.075 mmol, 75%).

^{1}H NMR (500 MHz, Chloroform-*d*) δ 8.73 (d, *J* = 4.4 Hz, 1H), 8.02 (d, *J* = 9.3 Hz, 1H), 7.53 (d, *J* = 4.4 Hz, 1H), 7.36 (dd, *J* = 9.2, 2.7 Hz, 1H), 5.73 – 5.63 (m, 1H), 5.57 (d, *J* = 4.3 Hz, 1H), 5.08 – 4.86 (m, 2H), 3.92 (s, 3H), 3.47 – 3.37 (m, 1H), 3.21 – 3.14 (m, 1H), 3.07 (dd, *J* = 13.7, 10.0 Hz, 1H), 2.72 – 2.64 (m, 1H), 2.48 – 2.41 (m, 1H), 2.08 – 1.97 (m, 2H), 1.82 – 1.69 (m, 5H), 1.69 – 1.61 (m, 1H), 1.59 – 1.51 (m, 1H), 1.50 – 1.41 (m, 1H).
^{13}C NMR (126 MHz, Chloroform-*d*) δ 157.8, 147.6, 147.5, 144.3, 136.9, 131.6, 126.6, 121.5, 118.4, 115.7, 101.3, 72.0, 59.9, 58.1, 55.7, 43.3, 39.0, 35.1, 28.3, 25.6, 21.5.
HRMS (ESI+) m/z calculated for $C_{21}H_{27}N_2O_2$ $[M+H]^+$ 339.20670, found 339.20713 (Δ = 1.3 ppm).
IR (film, cm^{-1}) 3074, 2925, 2865, 1620, 1508, 1240, 1028, 717
$[\alpha]_D^{20}$ = -94.5 (*c* = 0.2, $CDCl_3$)

(33R,35S,91R,92R,5S)-N-((1R,2R)-2-allyl-1-((cyclopropylsulfonyl)carbamoyl)cyclopropyl)-5-(tert-butyl)-17-methoxy-4,7-dioxo-2,8-dioxa-6-aza-1(2,3)-quinoxalina-3(3,1)-pyrrolidina-9(1,2)-cyclopropanacyclotetradecaphane-35-carboxamide (9)

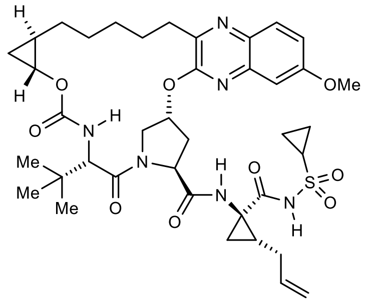

A 2 mL microwave vial was charged with a magnetic stirrer bar, 1CTR **1a** (62.5 mg, 0.25 mmol, 10.00 equiv), HG-II catalyst (3.2 mg, 0.005 mmol, 0.20 equiv) and grazoprevir (19.2 mg, 0.025 mmol, 1.00 equiv). The vial was sealed using a crimper and back-refilled with N_2 (3-cycles). Dichloromethane (0.25 mL) was added via a syringe and the reaction was heated at 40 °C for 8 h. The reaction was concentrated *in vacuo* and the residue was purified by C18 reverse phase chromatography (0-100% Water-Acetonitrile) to remove unreacted starting material. Product-containing fractions were concentrated by lyophilization.

The latent homolog was transferred to a 4 mL vial fitted with a magnetic stir bar. The vial was sealed using a crimper and back-refilled with N_2 (3-cycles). Dichloromethane (0.25 mL) was added via a syringe and the reaction was heated to 40 °C. A solution of 5:1 MeOH:$HCl_{(aq)}$ (0.25 mL) was added via a syringe and the reaction was heated for 16 h, followed by the standard work-up procedure (3.2).

The residue was purified by preparative HPLC using an Agilent 1260 Infinity with a reversed-phase C18 Supelcosil™ ABZ+PLUS column (250 mm × 21.2 mm, 5 µm) eluting with a gradient of 40-100% solvent A-Solvent B over 20 minutes (solvent A: 0.1% (v/v) TFA in H_2O, solvent B: 0.05% (v/v) TFA in MeCN). Product-containing fractions were lyophilized to give the product as an off-white powder (8.4 mg, 0.01076 mmol, 43%).

^{1}H NMR (700 MHz, Chloroform-*d*) δ 10.07 (s, br, 1H), 7.85 (d, *J* = 9.0 Hz, 1H), 7.22 (dd, *J* = 9.0, 2.8 Hz, 1H), 7.15 (d, *J* = 2.8 Hz, 1H), 6.54 (s, 1H), 6.02 (t, *J* = 4.1 Hz, 1H), 5.78 (ddt, *J* = 16.7, 10.2, 6.3 Hz, 1H), 5.33 (d, *J* = 9.8 Hz, 1H), 5.08 – 4.98 (m, 2H), 4.57 – 4.53 (m, 1H), 4.45 (d, *J* = 10.0 Hz, 1H), 4.33 – 4.28 (m, 1H), 4.07 (dd, *J* = 11.6, 4.0 Hz, 1H), 3.96 (s, 3H), 3.79 – 3.75 (m, 1H), 2.98 – 2.91 (m, 1H), 2.91 – 2.84 (m, 1H), 2.81 – 2.74 (m, 1H), 2.58 (dd, *J* = 14.4, 6.1 Hz, 1H), 2.50 – 2.43 (m, 1H), 2.38 (h, *J* = 9.4 Hz, 2H), 1.86 – 1.60 (m, 6H), 1.57 – 1.47 (m, 3H), 1.44 – 1.39 (m, 1H), 1.39 – 1.33 (m, 3H), 1.33 – 1.30 (m, 1H), 1.11 (s, 9H), 1.07 – 1.00 (m, 3H), 0.96 – 0.93 (m, 1H), 0.74 – 0.67 (m, 1H), 0.53 – 0.47 (m, 1H).
^{13}C NMR (176 MHz, Chloroform-*d*) δ 172.3, 172.1, 169.4, 160.4, 157.3, 154.7, 148.2, 140.8, 136.1, 134.4, 129.2, 118.8, 115.9, 105.9, 74.1, 60.0, 59.2, 55.7, 55.5, 54.7, 39.2, 35.1, 34.9, 34.2, 32.9, 31.3, 30.8, 30.1, 29.5, 28.4, 28.1, 26.5, 23.2, 18.6, 11.1, 6.3, 6.0.
HRMS (ESI+) m/z calculated for $C_{39}H_{53}N_6O_9S$ $[M+H]^+$ 781.35892, found 781.35883 (Δ = 0.1 ppm)
IR (film, cm^{-1}): 3299, 3000, 2956, 2929, 2856, 1709, 1621, 1502, 1417, 1345, 1224, 1166, 728
$[\alpha]_D^{20}$ = -41.5 (*c* = 0.2, $CDCl_3$)

(3S,4R,5S,8S,12R,14R,15S,16S,18R,19R,26aR,Z)-8-(but-3-en-1-yl)-5,19-dihydroxy-3-((E)-1-((1R,3R,4R)-4-hydroxy-3-methoxycyclohexyl)prop-1-en-2-yl)-14,16-dimethoxy-4,10,12,18-tetramethyl-5,6,8,11,12,13,14,15,16,17,18,19,24,25,26,26a-hexadecahydro-3H-15,19-epoxypyrido[2,1-c][1]oxa[4]azacyclotricosine-1,7,20,21(4H,23H)-tetraone (10)

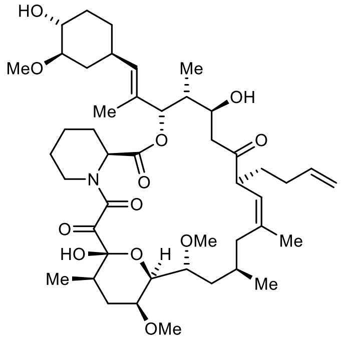

A 5 mL round-bottom flask was charged with a magnetic stirrer bar, 1CTR **1a** (250 mg, 1 mmol, 10.00 equiv) and Tacrolimus (80.4 mg, 0.1 mmol, 1.00 equiv). The round-bottom flask was fitted to a reflux condenser (Teflon tape around the ground glass joint, suba-seal on condenser outlet, connected to a N_2 line with a needle through the suba-seal) and evacuate-refilled with N_2 (3 cycles). The flask was then heated to 40 °C in an oil bath and dichloromethane (0.25 mL) was added through the top of the condenser. A stock solution of HG-II (12.6 mg/mL, 0.10 equiv) was prepared in dichloromethane and 0.5 mL of the stock solution was added, followed by dichloromethane (0.25 mL). The reaction was heated for 16 h, and then concentrated *in vacuo*. The residue was purified by C18 reverse phase chromatography (0-100% Water-Acetonitrile) to remove unreacted starting material. Product-containing fractions were concentrated by lyophilization.

The fractions containing the latent homolog and dimer of the homologating reagent, which co-eluted, were concentrated into a 10 mL microwave vial containing a magnetic stir bar. The vial was sealed using a crimper and back-refilled with N_2 (3-cycles). Dichloromethane (1 mL) was added via a syringe and the reaction was heated to 40 °C. Citric acid (576 mg, 3 mmol, 3.00 equiv) and TBAF·3H_2O (394 mg, 1.25 mmol, 1.25 equiv) were dissolved in 2 mL MeOH. The TBAF-citric acid solution was added through the septum of the microwave vial and the reaction was heated at 40 °C in an oil bath for 16 h, followed by the standard work-up procedure (3.2).

The residue was purified by preparative SFC using a Sepiatec 100 SFC with a Chiralpak IK column (250 mm × 20 mm, 5 µm) eluting with 25% MeOH + 0.1% NH_3 / 75% scCO_2. Product-containing fractions were lyophilized to give the product as an off-white powder (43.6 mg, 0.053 mmol, 53%).

Similarly to FK-506, homo FK-506 exists as a 2:1 mixture of trans:cis rotamers.[6]

^{1}H NMR (500 MHz, Chloroform-*d*) δ 5.82 – 5.68 (m, 1H), 5.30 (trans rotamer, d, *J* = 3.2 Hz, 0.65H), 5.17 (cis rotamer, d, *J* = 3.1 Hz, 0.34H), 5.10 – 4.93 (rotamers, m, 4.38H), 4.75 (cis rotamer, s, 0.33H), 4.59 (trans rotamer, d, *J* = 5.6 Hz, 0.65H), 4.45 – 4.38 (trans rotamer, m, 0.64H), 4.26 (trans rotamer, s, 0.61H), 3.97 – 3.83 (rotamers, m, 1.4H), 3.74 – 3.64 (m, 1H), 3.60 – 3.52 (m, 1H), 3.49 – 3.34 (m, 8H), 3.34 – 3.21 (m, 4H), 3.09 – 2.94 (m, 2H), 2.82 – 2.68 (m, 2H), 2.35 – 2.22 (m, 2H), 2.20 – 2.10 (m, 2H), 2.02 (d, *J* = 25.0 Hz, 6H), 1.92 – 1.83 (m, 2H), 1.83 – 1.70 (m, 3H), 1.67 – 1.54 (m, 9H), 1.59 – 1.29 (m, 6H), 1.10 – 1.01 (m, 2H), 1.01 – 0.80 (m, 11H).
^{13}C NMR (126 MHz, Chloroform-*d*) δ 213.2, 213.1, 196.1, 192.7, 169.0, 168.7, 165.7, 164.7, 139.8, 138.8, 138.0, 137.9, 132.3, 131.7, 129.8, 129.7, 123.2, 122.8, 115.3, 115.2, 98.6, 97.1, 84.1, 77.9, 76.5, 75.2, 73.6, 73.6, 73.5, 73.5, 72.8, 72.2, 70.1, 69.0, 57.6, 57.0, 56.6, 56.6, 56.6, 56.3, 56.1, 52.7, 52.3, 52.2, 48.6, 48.4, 43.9, 43.3, 40.5, 39.8, 39.2, 35.4, 34.9, 34.9, 34.8, 34.7, 34.6, 33.6, 33.1, 32.7, 32.5, 31.2, 31.2, 30.6, 30.2, 30.0, 27.6, 26.2, 26.0, 24.6, 24.5, 21.1, 20.8, 20.4, 19.5, 16.2, 16.1, 16.0, 15.7, 14.2, 14.1, 9.8, 9.5.
HRMS (ESI+) m/z calculated for $C_{45}H_{71}NO_{12}Na$ $[M+Na]^+$ 840.48685, found 840.48772 (Δ = 1.0 ppm)
IR (film, cm^{-1}): 3453, 2930, 2868, 2827, 1739, 1705, 1646, 1449, 1089, 1052, 1035, 988
$[\alpha]_D^{20}$ = -82.5 (*c* = 0.2, $CDCl_3$)

(2R,6aS,12aS)-2-(but-3-en-2-yl)-8,9-dimethoxy-1,2,12,12a-tetrahydrochromeno[3,4-b]furo[2,3-h]chromen-6(6aH)-one (11a)

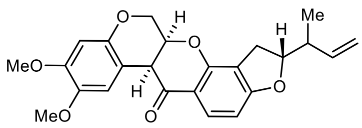

A 10 mL microwave vial was charged with a magnetic stirrer bar, 1CTR **1b** (83 mg, 0.3 mmol, 3.00 equiv), HG-II catalyst (12.5 mg, 0.02 mmol) and rotenone (39.4 mg, 0.1 mmol, 1.00 equiv). The vial was sealed using a crimper and back-refilled with N_2 (3-cycles). DCE (1 mL) was added via a syringe and the reaction was heated at 100 °C for 16 h. Butyl vinyl ether (~0.1 mL) was injected to quench the catalyst and the reaction mixture was concentrated *in vacuo*. The residue was purified by silica gel chromatography (30-50% EA in 40-60 PE) to remove unreacted starting material.

The fractions containing the latent homolog were concentrated *in vacuo* and transferred to a 20 mL microwave vial fitted with a magnetic stir bar. The vial was sealed using a crimper and back-refilled with N_2 (3-cycles). DCE (1.0 mL) was added via a syringe and the reaction was heated to 100 °C. Citric acid (173 mg, 0.9 mmol, 3.00 equiv) and TBAF·$3H_2O$ (118 mg, 1.25 mmol, 1.25 equiv) were dissolved in 1 mL MeOH. The TBAF-citric acid solution was added through the septum of the microwave vial and the reaction was heated at 100 °C for 16 h, followed by the standard work-up procedure (3.4). The residue was purified by C18 reverse column chromatography (0-100% Water-Acetonitrile). Product-containing fractions were lyophilized to give the product as a brown solid (29.8 mg, 0.0729 mmol, 73%) as a 1:1 mixture of diastereomers.

^{1}H NMR (700 MHz, Chloroform-*d*) δ 8.00 – 7.72 (m, 1H), 7.29 (s, 1H), 6.79 (s, 1H), 6.59 – 6.34 (m, 2H), 5.85 – 5.73 (m, 1H), 5.30 – 5.08 (m, 1H), 4.97 – 4.92 (m, 1H), 4.83 – 4.77 (m, 0.44H), 4.76 – 4.70 (m, 0.54H), 4.66 – 4.58 (m, 1H), 4.23 – 4.17 (m, 1H), 3.85 (d, *J* = 4.1 Hz, 1H), 3.83 (s, 3H), 3.78 (s, 3H), 3.21 – 3.11 (m, 1H), 2.94 – 2.87 (m, 1H), 2.64 – 2.54 (m, 1H), 1.16 (d, *J* = 6.8 Hz, 1.6H), 1.13 (d, *J* = 6.9 Hz, 1.34H).
^{13}C NMR (176 MHz, Chloroform-*d*) δ 189.0, 189.0, 167.6, 167.5, 157.9, 157.9, 149.5, 147.4, 147.4, 143.9, 143.9, 138.4, 138.3, 129.8, 116.4, 116.2, 113.3, 113.2, 113.2, 113.1, 110.4, 104.9, 104.9, 104.8, 100.9, 88.7, 88.6, 72.2, 66.3, 66.3, 56.3, 55.9, 44.6, 42.7, 42.6, 29.5, 29.3, 15.4, 15.2.
HRMS (ESI+) m/z calculated for $C_{24}H_{25}O_6$ $[M+H]^+$ 409.16456, found 409.16403 (Δ =1.3 ppm)
IR (film, cm^{-1}): 3073, 2962, 2930, 1668, 1606, 1510, 1455, 1213, 1192, 814

(5*R*)-5-(but-3-en-2-yl)-2-methylcyclohex-2-en-1-one (11b)

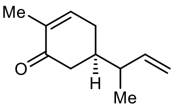

Prepared according to the general procedure for homologation of 1,1-disubstituted alkenes (3.4) from (*R*)-(−)-carvone (31.5 µL, 0.2 mmol, 1.00 equiv) using PhMe (1.5 mL) as the solvent, stirring for 16 h, followed by the retro-ene condition C. (organic phases from work-up were concentrated *in vacuo* with water bath temperature @ 20 °C, and rotary evaporator @ 300 mbar).

The product was too volatile to isolate via column chromatography. The assay yield was calculated to be 50% via quantitative ^{1}H NMR relative to 1,1,2,2-Tetrachloroethane (21 µL) a*s an internal standard. A ~1:1 mixture of diastereomers can be observed by GCMS with the correct corresponding mass for the product.*

(4R,4aS,6R)-6-(but-3-en-2-yl)-4,4a-dimethyl-4,4a,5,6,7,8-hexahydronaphthalen-2(3H)-one (11c)

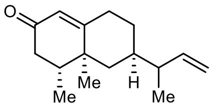

Prepared according to the general procedure for homologation of 1,1-disubstituted alkenes (3.4) from nootkatone (43.7 mg, 0.2 mmol, 1.00 equiv) and PhMe (1.5 mL) as the solvent, stirring for 16 h, followed by the retro-ene condition C. The residue was purified by C18 reverse column chromatography (20-100% Water-Acetonitrile). Product-containing fractions were lyophilized to give the product as a yellow oil (23.1 mg, 0.0994 mmol, 50%) as a 1:1 mixture of diastereomers.

^{1}H NMR (700 MHz, Chloroform-*d*) δ 5.77 (s, 1H), 5.74 – 5.65 (m, 1H), 5.07 – 4.88 (m, 2H), 2.51 – 2.42 (m, 1H), 2.39 – 2.31 (m, 1H), 2.32 – 2.20 (m, 2H), 2.06 – 1.88 (m, 4H), 1.63 (M, 1H), 1.19 – 1.11 (m, 1H), 1.11 – 1.07 (m, 3H), 1.04 (t, *J* = 7.0 Hz, 3H), 1.01 – 0.95 (m, 3H), 0.95 – 0.86 (m, 1H).
^{13}C NMR (176 MHz, Chloroform-*d*) δ 199.8, 199.8, 171.3, 171.3, 142.7, 142.3, 124.4, 124.4, 114.1, 114.0, 43.0, 43.0, 42.7, 42.6, 42.1, 42.1, 40.5, 40.5, 39.3, 39.3, 37.8, 37.7, 33.1, 33.1, 30.1, 30.0, 17.4, 17.2, 17.0, 16.9, 15.0, 15.0.
HRMS (ESI+) m/z calculated for $C_{16}H_{25}O$ $[M+H]^+$ 233.18999, found 233.19043 (Δ = 1.9 ppm)
IR (film, cm^{-1}): 2962, 2932, 1668, 1606, 1511, 1455, 1349, 1212, 1195, 1090, 748

***tert*-butyl 6-vinyl-2-azaspiro[3.3]heptane-2-carboxylate (11d)**

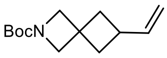

Prepared according to the general procedure for homologation of 1,1-disubstituted alkenes (3.4) from *tert*-butyl 6-methylene-2-azaspiro[3.3]heptane-2-carboxylate (83.7 mg, 0.4 mmol, 1.00 equiv), with the exception that HG-II catalyst (50 mg, 0.08 mmol, 0.20 equiv), 1CTR **1b** **(**334 mg, 1.2 mmol, 3.00 equiv) and DCE (3 mL) were used. The reaction was stirred for 16 h.

Owing to the incomplete consumption of starting material in the cross-metathesis reaction and the inseparability of the homolog from the starting olefin, the latent homolog was separated from the starting olefin by flash column chromatography on silica gel (20-30% EA in PE). The fractions containing the latent homolog, and remaining 1CTR **1b**, which co-eluted, were concentrated *in vacuo* and transferred to a 20 mL microwave vial containing a magnetic stir bar. The vial was sealed using a crimper and back-refilled with N_2 (3-cycles). DCE (2 mL) was added via a syringe and the reaction was heated to 100 °C. Citric acid (692 mg, 3.6 mmol, 3.00 equiv) and TBAF·3H_2O (473 mg, 1.5 mmol, 1.25 equiv) were dissolved in 3 mL MeOH (sonication required). The solution was added through the septum of the microwave vial and the reaction was heated and stirred at 100 °C for 16 h, followed by the standard work-up procedure.

The residue was purified by silica gel column chromatography (0-15% EA in 40-60 PE) to give the product as a colourless oil (34.7 mg, 0.155 mmol, 39%).

^{1}H NMR (400 MHz, Chloroform-*d*) δ 5.87 (ddd, *J* = 17.0, 10.3, 6.5 Hz, 1H), 5.01 – 4.90 (m, 2H), 3.96 (s, 2H), 3.81 (s, 2H), 2.85 – 2.72 (m, 1H), 2.38 – 2.29 (m, 2H), 2.08 – 1.97 (m, 2H), 1.44 (s, 9H).
^{13}C NMR (101 MHz, Chloroform-*d*) δ 156.2, 141.8, 112.9, 79.2, 38.7, 34.3, 32.9, 28.4.
HRMS (ESI+) m/z calculated for $C_9H_{14}NO_2$ $[M+H-^tBu]^+$168.10191, 168.10222 found (Δ = 1.8 ppm)
IR (film, cm^{-1}): 3079, 2975, 2978, 2869, 1699, 1390, 1356, 1166, 1099

hept-5-en-1-yl (((9H-fluoren-9-yl)methoxy)carbonyl)-L-isoleucinate (14a)

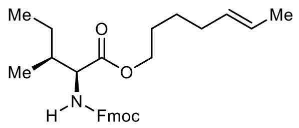

Prepared according to the general procedure for homologation of 1,2-disubstituted alkenes (3.5) from (*E*)-hex-4-en-1-yl ((((3Z,5E)-2-phenylocta-3,5,7-trien-1-yl)oxy)carbonyl)-L-isoleucinate (43.5 mg, 0.1 mmol, 1 equiv). The reaction was heated for 8 h, followed by stirring with ethyl vinyl ether (~0.1 mL) for ~5 minutes at rt. The reaction was concentrated *in vacuo* and the residue was purified by silica gel chromatography (10-20% EA in PE) to remove ruthenium species. Product-containing fractions were concentrated into a 10 mL microwave vial followed by retro-ene condition D.

The residue was purified by flash column chromatography on silica gel (10-20% EA in PE) to give the product as a white solid (39.1 mg, 0.0871 mmol, 87%).

^{1}H NMR (700 MHz, Chloroform-*d*) δ 7.79 (dd, *J* = 7.5, 2.0 Hz, 2H), 7.68 – 7.56 (m, 2H), 7.43 (td, *J* = 7.5, 3.0 Hz, 2H), 7.35 (td, *J* = 7.4, 3.0 Hz, 2H), 5.57 – 5.31 (m, 3H), 4.42 (d, *J* = 7.2 Hz, 2H), 4.40 – 4.35 (m, 1H), 4.26 (t, *J* = 7.2 Hz, 1H), 4.23 – 4.12 (m, 2H), 2.10 (*Z*-isomer, q, *J* = 7.4 Hz, 0.52H), 2.06 – 2.00 (*E*-isomer, m, 1.47H), 1.97 – 1.89 (m, 1H), 1.68 (dd, *J* = 13.9, 5.2 Hz, 5H), 1.51 – 1.40 (m, 3H), 1.27 – 1.18 (m, 1H), 1.01 – 0.93 (m, 5H), 0.93 – 0.85 (m, 1H).
^{13}C NMR *E*-isomer (176 MHz, Chloroform-*d*) δ 172.2, 156.1, 144.0, 143.8, 141.3, 130.7, 127.7, 127.1, 125.5, 125.1, 120.0, 120.0, 67.0, 65.3, 58.4, 47.2, 38.2, 32.0, 28.0, 25.8, 25.1, 17.9, 15.5, 11.7.
^{13}C NMR *Z*-isomer (176 MHz, Chloroform-*d*) δ 172.2, 156.1, 144.0, 143.8, 141.3, 129.8, 127.7, 127.1, 125.1, 124.5, 120.0, 120.0, 67.0, 65.3, 58.4, 47.2, 38.2, 28.1, 26.3, 25.8, 25.1, 15.5, 12.8, 11.7.
IR (film, cm^{-1}): 3412, 2963, 2932, 2876, 2857, 1737, 1721, 1513, 1225, 1194, 1084, 1040, 755, 740.
HRMS (ESI+) m/z calculated for $C_{28}H_{36}NO_4$ $[M+H]^+$ 450.26389, 450.26477 found (Δ = 1.9 ppm)

p-[trans-4-(2E-butenyl)cyclohexyl]benzonitrile (14b)

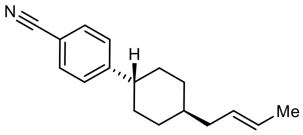

Prepared according to the general procedure for homologation of 1,2-disubstituted alkenes (3.5) from 4-(trans-4-((*E*)-Prop-1-en-1-yl)cyclohexyl)benzonitrile (45.1 mg, 0.2 mmol, 1.00 equiv), with the exception that 1CTR **1c** (278 mg, 1 mmol, 5.00 equiv) and HG-II (12.6 mg/mL, 0.10 equiv) were used. The reaction was heated for 8 h, followed by stirring with ethyl vinyl ether (~0.1 mL) for ~5 minutes at rt. The reaction was concentrated *in vacuo* and the residue purified by silica gel chromatography (10-25% EA in PE) to remove ruthenium species.

Product containing fractions, and dimer of 1CTR **1c**, which co-eluted, were concentrated into a 10 mL microwave vial followed by retro-ene condition D (scaled to 0.2 mmol) - citric acid (576 mg, 3 mmol, 3.00 equiv) and TBAF·3H_2O (394 mg, 1.25 mmol, 1.25 equiv), followed by the standard work-up procedure.

The residue was purified by flash column chromatography on silica gel PE:Et_2O 20:1 to give the product as a colourless oil (23.8 mg, 0.099 mmol, 50%).

^{1}H NMR (700 MHz, Chloroform-*d*) δ 7.59 (dd, *J* = 8.3, 1.6 Hz, 2H), 7.32 (dd, *J* = 8.3, 1.9 Hz, 2H), 5.57 – 5.41 (m, 2H), 2.58 – 2.50 (m, 1H), 2.03 (Z-isomer, t, *J* = 7.1 Hz, 0.58H), 1.99 – 1.94 (*E*-isomer, m, 1.41H), 1.94 – 1.88 (m, 4H), 1.73 – 1.67 (*E*-isomer, m, 2.13H), 1.66 – 1.62 (*Z*-isomer, m, 0.85H), 1.50 – 1.33 (m, 3H), 1.18 – 1.05 (m, 2H).
^{13}C NMR *E*-isomer (176 MHz, Chloroform-*d*) δ 153.3, 132.2, 129.6, 127.7, 126.1, 119.2, 109.6, 44.7, 40.4, 37.6, 33.8, 33.0, 18.0.
^{13}C NMR *Z*-isomer (176 MHz, Chloroform-*d*) δ 153.2, 132.2, 128.8, 127.7, 124.7, 119.2, 109.6, 44.7, 37.8, 34.3, 33.9, 33.0, 12.9.
IR (film, cm^{-1}): 3014, 2918, 2851, 2226, 1607, 1503, 1447, 964, 828, 561
HRMS (ESI+) m/z calculated for $C_{12}H_{22}N$ $[M+H]^+$ 240.17468, 240.17480 found (Δ = 0.5 ppm)

hept-5-en-1-yl 2-(1-(4-chlorobenzoyl)-5-methoxy-2-methyl-1H-indol-3-yl)acetate (14c)

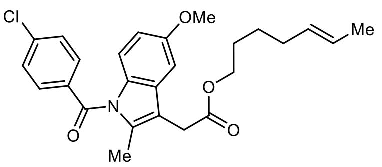

Prepared according to the general procedure for homologation of 1,2-disubstituted alkenes (3.5) from (*E*)-hex-4-en-1-yl-2-(1-(4-chlorobenzoyl)-5-methoxy-2-methyl-1H-indol-3-yl)acetate (43.9 mg, 0.1 mmol, 0.1 equiv). The reaction was heated for 8 h, followed by stirring with ethyl vinyl ether (~0.1 mL) for ~5 minutes at rt. The reaction was concentrated *in vacuo* and the residue was purified by silica gel chromatography (25-30% EA in PE) to remove ruthenium species. Product-containing fractions were concentrated into a 10 mL microwave vial followed by retro-ene condition D.

The residue was purified by flash column chromatography on silica gel (10-20% EA in PE) to give the product as a yellow solid (38.2 mg, 0.0843 mmol, 84%).

^{1}H NMR (400 MHz, Chloroform-*d*) δ 7.68 (d, *J* = 8.4 Hz, 2H), 7.49 (d, *J* = 8.4 Hz, 2H), 6.99 (d, *J* = 2.6 Hz, 1H), 6.90 (d, *J* = 9.0 Hz, 1H), 6.69 (dd, *J* = 8.9, 2.6 Hz, 1H), 5.48 – 5.29 (m, 2H), 4.17 – 4.08 (m, 2H), 3.86 (s, 3H), 3.68 (s, 2H), 2.41 (s, 3H), 2.10 – 1.93 (*Z*-isomer, m, 0.51H; *E*-isomer, m, 1.52H), 1.70 – 1.57 (m, 5H), 1.47 – 1.32 (m, 2H).

^{13}C NMR *E*-isomer (101 MHz, Chloroform-*d*) δ 170.9, 168.3, 156.1, 139.2, 135.9, 134.0, 131.2, 130.8, 130.7, 130.7, 129.1, 125.3, 115.0, 112.8, 111.7, 101.3, 65.0, 55.7, 32.0, 30.4, 28.1, 25.8, 17.9, 13.4.

^{13}C NMR *Z*-isomer (101 MHz, Chloroform-*d*) δ 170.9, 168.3, 156.1, 139.2, 135.9, 134.0, 131.2, 130.8, 130.7, 129.9, 129.1, 124.4, 115.0, 112.8, 111.7, 101.3, 65.0, 55.7, 30.4, 28.2, 26.3, 25.8, 17.9, 12.8.

IR (film, cm^{-1}): 2930, 2855, 1731, 1680, 1477, 1312, 1220, 1163, 1141, 1087, 1066, 753

HRMS (ESI+) m/z calculated for $C_{26}H_{29}NO_4Cl$ $[M+H]^+$ 454.17796, 454.17916 found (Δ = 2.6 ppm)

1-cyclohexylpent-3-en-1-ol (14d)

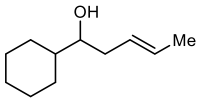

Prepared according to the general procedure for homologation of 1,2-disubstituted alkenes (3.5) from 1-cyclohexyl-2-buten-1-ol (15.4 mg, 0.1 mmol, 1 equiv). The reaction was heated for 8 h, followed by stirring with ethyl vinyl ether (~0.1 mL) for ~5 minutes at rt. The reaction was concentrated *in vacuo* and the residue was purified by silica gel chromatography (5-30% EA in PE) to remove ruthenium species. Product-containing fractions were concentrated into a 10 mL microwave vial followed by the retro-ene condition D.

The product was too volatile to isolate via column chromatography. The assay yield was calculated to be 55% via quantitative ^{1}H NMR relative to methyl benzoate (12.6 µL) as an internal standard. A 70:30 E:Z ratio can be observed by GCMS with the correct corresponding masses for the product.

(*E*)-N-(4-hydroxy-3-methoxybenzyl)-9-methyldec-7-enamide (15)

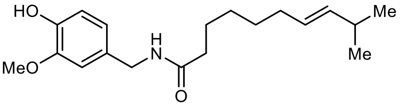

A 4 mL screw-top vial fitted with a magnetic stirrer bar was charged with 1CTR **1d** (141 mg, 0.48 mmol, 5.00 equiv‡), natural capsaicin extract† (50 mg, 58.8% capsaicin, 28.4% dihydrocapsaicin, 9.6% other capsaicinoids) and HG-II (12 mg, 0.019 mmol, 0.20 equiv‡). The vial was fitted with a screwtop septum and heated under an active vacuum (~5 mbar) for 6 h at 100 °C. The crude reaction was then filtered through a pad of activated charcoal with EtOAc (50 mL). The filtrate was concentrated into a 4 mL vial which was evacuate-refilled with N_2 (3 cycles), charged with dichloromethane (0.5 mL) and heated to 40 °C with stirring. A stock solution of MeOH:$HCl_{(aq)}$5:1 (0.5 mL) was then added and the reaction was stirred for 16 h. After the standard work-up procedure (3.5), the extract was taken up in $CDCl_3$ and analysed by ^{1}H NMR to determine the alkene content of the extract.

‡ *Relative to the amount of capsaicin in extract.*

† *Obtained from Doug Discovery (Fluorochem); cat no: M01481; batch no: FCB018447.*

Capsaicin is the only major alkene-containing component in capsaicin extract (homocapsaicin <1%) with the remainder of capsaicinoids being saturated. Less than 5% capsaicin remained after completion of the cross-metathesis reaction. Alkene content of the crude extract after one-carbon homologation was determined to be ~51%.

It was not possible to isolate pure homocapsaicin from the extract (homocapsaicin and dihydrocapsaicin co-elute by HPLC). However, an aliquot of the crude extract was purified by semi-preparative HPLC (5-95% H_2O-MeCN) to give a mixed sample of homocapsaicin and dihydrocapsaicin. ^{1}H NMR of the isolated sample contained 45% homocapsaicin (85:15 *E*:*Z* by relative integrals of the terminal methyl groups) and 55% dihydrocapsaicin.

^{1}H NMR (400 MHz, $CDCl_3$) δ 6.86 (d, *J* = 8.0 Hz, 1H), 6.80 (s, 1H), 6.77 – 6.73 (m, 1H), 5.69 (s, 2H), 5.46 – 5.09 (m, 0.96H), 4.35 (d, *J* = 5.6 Hz, 2H), 3.87 (s, 3H), 2.27 – 2.12 (m, 2.67H), 2.00 – 1.86 (m, 0.8H), 1.71 – 1.58 (m, 2.46H), 1.49 (m, 0.76H), 1.40 – 1.20 (m, 6.64H), 1.19 – 1.08 (m, 1.24H), 0.94 (**homocapsaicin**, d, *J* = 6.7 Hz, 2.68H), 0.85 (**dihydrocapsaicin**, d, *J* = 6.6 Hz, 3.33H).
^{13}C NMR (101 MHz, Chloroform-*d*) δ 172.9, 172.9, 146.7, 145.1, 145.1, 137.8, 130.4, 130.4, 127.0, 126.8, 120.8, 114.4, 114.4, 110.7, 110.7, 55.9, 55.9, 43.5, 43.5, 38.9, 36.9, 36.8, 32.3, 31.0, 29.6, 29.4, 29.3, 28.8, 27.9, 27.2, 25.8, 25.6, 23.2, 22.7, 22.6.
HRMS m/z calculated for $C_{19}H_{29}NO_3$ $[M+H]^+$ 320.22202, found 320.22208 (Δ = 0.6 ppm)

15 is a known compound and its NMR spectra are in accord with published data.[7]

tert-butyl (R)-(1-phenylhex-4-en-2-yl)carbamate (16)

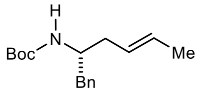

A 5 mL round-bottom flask was charged with a magnetic stirrer bar, 1CTR **1c** (106 mg, 0.4 mmol, 4.00 equiv) and *tert*-butyl (*S*)-(1-phenylbut-3-en-2-yl)carbamate (24.7 mg, 0.1 mmol, 1.00 equiv). The round-bottom flask was fitted to a reflux condenser (Teflon tape around the ground glass joint, suba-seal on condenser outlet, connected to a N_2 line with a needle through the suba-seal) and evacuate-refilled with N_2 (3 cycles). The flask was then heated to 40 °C in an oil bath and dichloromethane (0.25 mL) was added through the top of the condenser to dissolve the starting materials. A stock solution of HG-II (6.3 mg/mL, 0.05 equiv) was prepared in dichloromethane and 0.5 mL of the stock solution was added, followed by dichloromethane (0.25 mL). An outlet needle was pierced through the suba-seal to help purge the reaction vessel of ethylene, and the reaction was heated for 16 h with stirring. After reaction completion, the catalyst was quenched using ethyl vinyl ether (~0.1 mL).

The latent homolog was purified by flash column chromatography on silica gel (10-30% EA in PE). After chromatographic purification, the latent homolog, and excess 1CTR 1c, which co-eluted, was concentrated into a 10 mL microwave vial. The vial was sealed using a crimper, back-refilled with N_2 (3-cycles), charged with dichloromethane (0.5 mL) and heated to 40 °C. Citric acid (231 mg, 1.2 mmol, 3.00 equiv) and TBAF·$3H_2O$ (158 mg, 0.5 mmol, 1.25 equiv) were dissolved in 2 mL MeOH (sonication required). The solution was added through the septum of the microwave vial and the reaction was heated and stirred at 40 °C in an oil bath for 16 h.

The reaction mixture was transferred to a separatory funnel containing $NaHCO_3$ (approx. 50 mL). The aqueous phase was extracted 3x using Et_2O. The combined organic phases were dried over $MgSO_4$, filtered and concentrated *in vacuo*. The residue was purified by flash column chromatography on silica gel (5-10% EtOAc in PE) to give the product as a white solid (16.4 mg, 0.06 mmol, 60%).

^{1}H NMR (400 MHz, $CDCl_3$) δ 7.34 – 7.29 (m, 2H), 7.26 – 7.18 (m, 3H), 5.66 – 5.39 (m, 2H), 4.40 (s, 1H), 3.87 (s, 1H), 2.87 – 2.70 (m, 2H), 2.32 – 2.00 (m, 2H), 1.73 – 1.67 (*E*-isomer, m, 2.11H), 1.64 – 1.59 (*Z*-isomer, m, 0.79H), 1.43 (s, 9H).
^{13}C NMR (126 MHz, $CDCl_3$) δ 155.4, 138.3, 129.5, 129.4, 128.6, 128.3, 126.9, 126.7, 126.3, 126.2, 125.8, 79.0, 51.3, 40.4, 36.8, 28.4, 18.1 (*E*-isomer), 13.1 (*Z*-isomer).
IR (film, cm^{-1}): 3351, 3026, 2976, 2929, 1692, 1496, 1453, 1390, 1365, 1249, 1169
HRMS (ESI+) m/z calculated for $C_{17}H_{26}O_2N$ $[M+H]^+$ 276.1958, 276.1948 found (Δ = - 3.8 ppm)

non-8-en-1-yl undec-10-enoate (19)

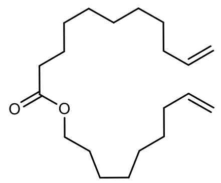

Prepared according to the general procedure for homologation of monosubstituted alkenes (3.2) from ambrettolide (50.5 mg, 0.2 mmol, 1.00 equiv), with the exception that 1CTR **1a** (400 mg, 1.6 mmol, 8.00 equiv) and HG-II stock solution (12.6 mg/mL, 0.10 equiv) were used. The reaction was stirred for 16 h, followed by retro-ene condition B†. The residue was purified by C18 reverse column chromatography (40-100% Water-Acetonitrile). Product-containing fractions were extracted into pentane, and concentrated in vacuo to give the product as a colourless oil (50.1 mg, 0.163 mmol, 81%).

† Higher loading of citric acid and TBAF·3H_2O is required to compensate for the higher equivalents of 1CTR **1a**. Citric acid (922 mg, 4.8 mmol, 3.00 equiv) and TBAF·3H_2O (631 mg, 2 mmol, 1.25 equiv) were dissolved in 3 mL MeOH.

^{1}H NMR (400 MHz, $CDCl_3$) δ 5.90 – 5.76 (m, 2H), 5.13 – 4.86 (m, 4H), 4.08 (t, *J* = 6.7 Hz, 2H), 2.31 (t, *J* = 7.5 Hz, 2H), 2.11 – 2.01 (m, 4H), 1.69 – 1.57 (m, 4H), 1.46 – 1.11 (m, 18H).
^{13}C NMR (101 MHz, $CDCl_3$) δ 174.0, 139.2, 139.1, 114.2, 114.1, 64.4, 34.4, 33.8, 33.8, 29.3, 29.2, 29.1, 29.1, 29.1, 29.0, 28.9, 28.8, 28.6, 25.9, 25.0.
IR (film, cm^{-1}): 3078, 2924, 2854, 1735, 1640, 1168, 907
HRMS m/z calculated for $C_{20}H_{37}O_2$ $[M+H]^+$ 309.27881, found 309.27874 (Δ = -0.2 ppm)

oxacyclononadec-11-en-2-one *E*/*Z*-(20)

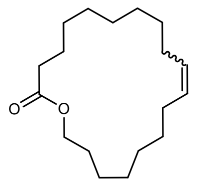

Condition A: *E*-selective RCM using UltraNitroCat.[8]

Non-8-en-1-yl undec-10-enoate (**19**) (25 mg, 0.08 mmol, 1 equiv) and UltraNitroCat (CAS: 2106819-64-9) (0.9 mg, 1.3 μmol, 0.016 equiv)) were weighed into separate 4 mL vials fitted with screw-top septa and back-refilled with argon (3-cycles). PhMe (1 mL) was added to each of the 4 mL vials to dissolve the reagents. A 100 mL 3-neck round bottom flask fitted was fitted with a reflux condenser and back-refilled with argon (3-cycles). The round-bottom flask was heated to 70 °C, charged with PhMe (17 mL), followed by the 1 mL stock solution of **19**. An additional 1 mL of PhMe was added to the vial containing **19**, and washings were transferred to the 100 mL round-bottom-flask. Two long needles connected an argon line were pierced through suba-seals in the left and right neck of the round-bottom flask. An outlet needle was pierced through the suba-seal on top of the condenser. The solution was vigorously sparged with argon. The UltraNitroCat stock solution (1 mL) was injected into the reaction. The reaction was stirred for 25 minutes with vigorous sparging, at which point full consumption of **19** was indicated by TLC. The reaction was then quenched using SnatchCat (4.4 eq in relation to catalyst) and stirred for 5 minutes at room temperature. Volatiles were then concentrated *in vacuo.*

The residue was purified by C18 reverse column chromatography (40-100% Water-Acetonitrile). Product-containing fractions were extracted into pentane, and concentrated *in vacuo* to give the product as a colourless oil (20.5 mg, 0.073 mmol, 89%).

Note: the use of an efficient reflux condenser is required to prevent the evaporation of toluene and concentration of the reaction mixture during RCM.

^{1}H NMR (500 MHz, $CDCl_3$) δ 5.39 – 5.24 (m, 2H), 4.15 – 4.08 (m, 2H), 2.35 – 2.27 (m, 2H), 2.09 – 1.96 (m, 4H), 1.70 – 1.57 (m, 4H), 1.40 – 1.21 (m, 18H).
^{13}C NMR (126 MHz, $CDCl_3$) δ 174.0, 174.0, 130.9, 130.8, 130.4, 130.1, 64.3, 63.9, 34.6, 34.4, 32.1, 32.0, 29.6, 29.5, 29.2, 29.1, 29.1, 29.0, 29.0, 28.9, 28.9, 28.9, 28.7, 28.7, 28.7, 28.6, 28.1, 28.0, 27.8, 27.1, 26.5, 26.2, 26.0, 25.2.
IR (film, cm^{-1}): 2923, 2853, 1732, 1462. 1237, 911, 731
HRMS m/z calculated for $C_{18}H_{33}O_2$ $[M+H]^+$ 281.24751, found 281.24748 (Δ = - 0.1 ppm)
***E*:*Z* ratio (3:1)** as determined by quantitative ^{13}C NMR acquired at 176 MHz (interpulse delay was set to be 5x the longest value measured, and quantitative ^{13}C acquisition parameter set was used to mitigate ^{13}C-^{1}H NoE's affecting the result).

Condition B: *Z*-selective RCM using M2001.[9]

A 20 mL microwave vial was charged with a solution of non-8-en-1-yl undec-10-enoate (**19**) (18 mg, 58.4 µmol) in dichloroethane (10.6 mL). A solution of M2001 catalyst (7.5 mol%) dissolved in dichloroethane (1.0 mL) was added via a syringe. The microwave vial was subjected to a single freeze-pump-thaw cycle. The vial was kept under a static vacuum (~20 mbar) and heated at 60 °C. After 24 hours, the mixture was allowed to warm to room temperature. The catalyst was quenched with ethyl vinyl ether (2-3 drops), and concentrated in vacuo.

The assay yield was determined to be 74% by ^{1}H NMR relative to methyl benzoate as an internal standard.

*It was not possible to isolate pure oxacyclononadec-11-en-2-one from the crude reaction due to co-elution of uncyclized non-8-en-1-yl undec-10-enoate (**19**). ^{1}H and ^{13}C NMR of mixed Z-***20** and **19** are provided.

***Z*:*E* ratio (9:1)** as determined by quantitative ^{13}C NMR acquired at 176 MHz (interpulse delay was set to be 5x the longest value measured, and quantitative ^{13}C acquisition parameter set was used to mitigate ^{13}C-^{1}H NoE's affecting the result).

(3S,6S,9S,12R,15S,18S,21R,24S,30S,33S)-30-ethyl-33-((1R,2R,E)-1-hydroxy-2-methylhept-5-en-1-yl)-6,9,18,24-tetraisobutyl-3,21-diisopropyl-1,4,7,10,12,15,19,25,28-nonamethyl-1,4,7,10,13,16,19,22,25,28,31-undecaazacyclotritriacontan-2,5,8,11,14,17,20,23,26,29,32-undecaone (*homo* Cyclosporine A – 21)

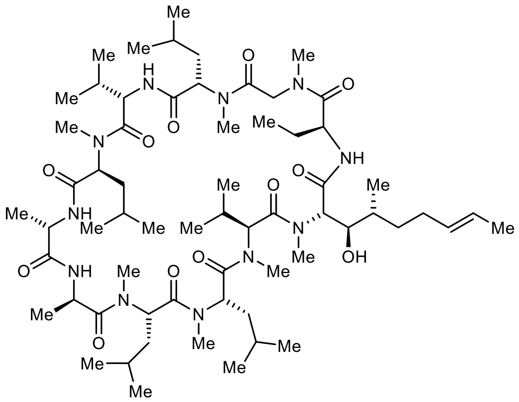

A 10 mL microwave vial was charged with a magnetic stirrer bar, 1CTR **1c** (132 mg, 0.5 mmol, 5.00 equiv), Cyclosporine A (120 mg, 0.1 mmol, 1.00 equiv) and nitro-Grela-I_2 SIPr (CAS: 1874265-00-5) catalyst (18.8 mg, 0.02 mmol, 0.20 equiv.) The vial was sealed using a crimper and back-refilled with N_2 (3-cycles). Dichloroethane (1 mL) was added via a syringe and the reaction was heated to 100 °C in an oil bath for 6 h. The reaction mixture was concentrated *in vacuo* and the residue was then purified by C18 reverse phase chromatography (0-100% Water-Acetonitrile) to remove unreacted starting material. The product-containing fractions were concentrated by lyophilization.

The fractions containing the latent homolog and dimer of the 1CTR **1c**, which co-eluted, were transferred to a 10 mL microwave vial containing a magnetic stir bar. The vial was sealed using a crimper and back-refilled with N_2 (3-cycles). Dichloromethane (1 mL) was added via a syringe and the reaction was heated to 40 °C. Citric acid (288 mg, 1.5 mmol, 3.00 equiv) and TBAF·3H_2O (197 mg, 0.63 mmol, 1.25 equiv) were dissolved in 2 mL MeOH. The solution was added through the septum of the microwave vial and the reaction was heated for 16 h, followed by the standard work-up procedure (3.5). The residue was purified by C18 reverse column chromatography (0-100% Water-Acetonitrile). Product-containing fractions were lyophilized to give the product as an off-white powder white solid (59.7 mg, 0.049 mmol, 49%).

^{1}H NMR (700 MHz, Chloroform-*d*) δ 8.00 (d, *J* = 10.1 Hz, 1H), 7.68 (d, *J* = 7.3 Hz, 1H), 7.49 (d, *J* = 8.5 Hz, 1H), 7.20 (d, *J* = 8.0 Hz, 1H), 5.73 (dd, *J* = 10.7, 4.4 Hz, 1H), 5.50 (d, *J* = 6.3 Hz, 1H), 5.42 – 5.36 (m, 1H), 5.35 – 5.28 (m, 2H), 5.15 (d, *J* = 10.9 Hz, 1H), 5.10 – 5.03 (m, 2H), 4.99 (dd, *J* = 9.8, 5.8 Hz, 1H), 4.89 – 4.83 (m, 1H), 4.75 (d, *J* = 14.0 Hz, 1H), 4.68 (t, *J* = 9.0 Hz, 1H), 4.55 (p, *J* = 6.9 Hz, 1H), 3.77 (t, *J* = 6.9 Hz, 1H), 3.53 (s, 3H), 3.41 (s, 3H), 3.29 (s, 3H), 3.12 (s, 6H), 2.73 (s, 3H), 2.71 (s, 3H), 2.65 (s, 2H), 2.49 – 2.43 (m, 1H), 2.19 – 1.95 (m, 7H), 1.95 – 1.86 (m, 1H), 1.83 – 1.76 (m, 2H), 1.76 – 1.70 (m, 1H), 1.67 – 1.58 (m, 5H), 1.53 – 1.48 (m, 1H), 1.47 – 1.39 (m, 2H), 1.37 (d, *J* = 7.2 Hz, 3H), 1.34 – 1.22 (m, 6H), 1.10 (d, *J* = 6.7 Hz, 3H), 1.06 – 1.00 (m, 9H), 0.97 (dd, *J* = 14.7, 6.7 Hz, 10H), 0.93 – 0.83 (m, 18H), 0.76 (d, *J* = 6.7 Hz, 3H).

^{13}**C NMR** (126 MHz, Chloroform-*d*) δ 173.9, 173.8, 173.7, 173.4, 171.6, 171.3, 171.1, 170.5, 170.3, 170.2, 170.1, 131.8 (*E*-isomer), 130.9 (*Z*-isomer), 124.3 (*E*-isomer), 123.4 (*Z*-isomer), 75.1, 59.0, 57.9, 57.6, 55.5, 55.4, 55.3, 50.3, 48.7, 48.5, 48.1, 45.1, 41.0, 40.5, 39.5, 39.0, 37.5, 36.0, 36.0, 34.0, 32.6, 31.6, 31.3, 31.1, 30.2, 29.8, 29.8, 29.7, 29.5, 29.2, 25.3, 25.3, 24.9, 24.9, 24.6, 24.4, 23.9, 23.8, 23.7, 23.6, 23.4, 21.8, 21.8, 21.2, 21.1, 20.4, 20.0, 18.7, 18.3, 18.2, 17.9, 17.1, 17.0, 15.9, 12.8, 9.9.
HRMS (ESI+) m/z calculated for $C_{63}H_{114}N_{11}O_{12}$ $[M+H]^+$ 1216.86429, found 1216.86379 (Δ = -0.4 ppm)
***Z*:*E* ratio 23:100 (~1:5)** as determined by quantitative ^{13}C NMR acquired at 176 MHz (interpulse delay was set to be 5x the longest value measured, and quantitative ^{13}C acquisition parameter set was used to mitigate ^{13}C-^{1}H NoE's affecting the result).

(3S,6S,9S,12R,15S,18S,21R,24S,30S,33S)-30-ethyl-33-((1R,2R,E)-1-hydroxy-2-methyloct-6-en-1-yl)-6,9,18,24-tetraisobutyl-3,21-diisopropyl-1,4,7,10,12,15,19,25,28-nonamethyl-1,4,7,10,13,16,19,22,25,28,31-undecaazacyclotritriacontan-2,5,8,11,14,17,20,23,26,29,32-undecaone (*bishomo* Cyclosporine A – 22)

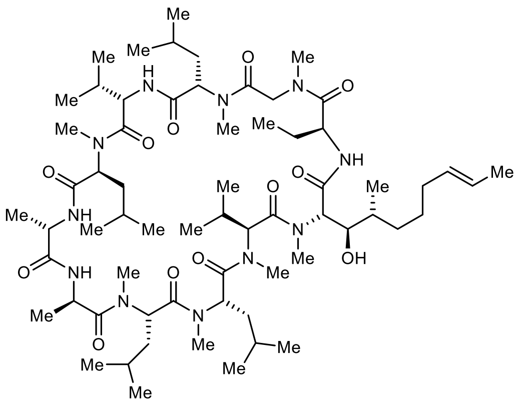

A 10 mL microwave vial was charged with a magnetic stirrer bar, 1CTR **1c** (65.1 mg, 0.247 mmol, 5.00 equiv), *homo* Cyclosporine A (**21**) (60 mg, 0.049 mmol, 1 equiv) and nitro-Grela-I_2 SIPr (CAS: 1874265-00-5) catalyst (9.3 mg, 9.87 μmol, 0.20 equiv.) The vial was sealed using a crimper and back-refilled with N_2 (3-cycles). Dichloroethane (0.5 mL) was added via a syringe and the reaction was heated to 100 °C in an oil bath for 6 h. The reaction mixture was concentrated *in vacuo* and the residue was then purified by C18 reverse phase chromatography (0-100% Water-Acetonitrile) to remove unreacted starting material. Product-containing fractions were concentrated by lyophilization.

The fractions containing the latent homolog and dimer of the 1CTR **1c**, which co-eluted, were transferred to a 10 mL microwave vial containing a magnetic stir bar. The vial was sealed using a crimper and back-refilled with N_2 (3-cycles). Dichloromethane (1 mL) was added via a syringe and the reaction was heated to 40 °C. Citric acid (144 mg, 0.75 mmol, 3.00 equiv) and TBAF·$3H_2O$ (99 mg, 0.313 mmol, 1.25 equiv) were dissolved in 1 mL MeOH. The solution was added through the septum of the microwave vial and the reaction was heated for 16 h, followed by the standard work-up procedure (3.5). The residue was purified by C18 reverse column chromatography (0-100% Water-Acetonitrile). Product-containing fractions were lyophilized to give the product as an off-white powder white solid (25.0 mg, 0.0203 mmol, 41%).

^{1}H NMR (700 MHz, Chloroform-*d*) δ 7.98 (d, *J* = 10.2 Hz, 1H), 7.69 (d, *J* = 7.4 Hz, 1H), 7.48 (d, *J* = 8.3 Hz, 1H), 7.19 (d, *J* = 8.1 Hz, 1H), 5.73 (dd, *J* = 11.1, 4.5 Hz, 1H), 5.50 (d, *J* = 5.8 Hz, 1H), 5.42 (h, *J* = 5.2 Hz, 2H), 5.31 (dd, *J* = 11.9, 4.1 Hz, 1H), 5.16 (d, *J* = 11.0 Hz, 1H), 5.06 (dt, *J* = 18.5, 7.2 Hz, 2H), 4.97 (dd, *J* = 10.0, 5.8 Hz, 1H), 4.85 (p, *J* = 6.9 Hz, 1H), 4.75 (d, *J* = 14.1 Hz, 1H), 4.67 (t, *J* = 8.9 Hz, 1H), 4.55 (p, *J* = 7.4 Hz, 1H), 3.77 – 3.73 (m, 1H), 3.53 (s, 3H), 3.41 (s, 3H), 3.29 (s, 3H), 3.13 (s, 3H), 3.12 (s, 3H), 2.73 (s, 3H), 2.71 (s, 3H), 2.65 (s, 2H), 2.49 – 2.41 (m, 1H), 2.19 – 2.11 (m, 2H), 2.13 – 2.04 (m, 2H), 2.00 (ddd, *J* = 14.3, 10.4, 5.1 Hz, 1H), 1.96 – 1.89 (m, 1H), 1.90 – 1.82 (m, 1H), 1.83 – 1.76 (m, 1H), 1.76 – 1.68 (m, 2H), 1.66 – 1.63 (m, 3H), 1.63 – 1.55 (m, 2H), 1.54 – 1.46 (m, 1H), 1.46 – 1.39 (m, 3H), 1.37 (d, *J* = 7.2 Hz, 3H), 1.36 – 1.31 (m, 2H), 1.31 – 1.23 (m, 6H), 1.10 (d, *J* = 6.6 Hz, 3H), 1.07 – 1.01 (m, 9H), 1.01 – 0.93 (m, 10H), 0.92 – 0.84 (m, 18H), 0.74 (d, *J* = 6.7 Hz, 3H).

^{13}C NMR (101 MHz, Chloroform-*d*) δ 173.8, 173.7, 173.6, 173.4, 171.5, 171.2, 171.1, 171.1, 170.5, 170.4, 170.1, 170.1, 131.8 (*E*-isomer), 130.8 (*Z*-isomer), 124.3 (*E*-isomer), 123.4 (*Z*-isomer), 75.0, 58.9, 57.8, 57.5, 55.4, 55.4, 55.2, 50.3, 48.7, 48.5, 48.1, 45.1, 40.5, 39.4, 39.0, 37.4, 36.0, 35.9, 33.9, 32.5, 31.5, 31.3, 31.1, 30.1, 29.8, 29.8, 29.5, 29.1, 25.3, 25.0, 24.8, 24.6, 24.4, 23.8, 23.7, 23.7, 23.4, 23.4, 21.8, 21.8, 21.2, 21.1, 20.3, 19.8, 18.6, 18.3, 18.1, 17.9, 17.0, 17.0, 15.9, 12.7, 9.9.
HRMS (ESI+) m/z calculated for $C_{64}H_{116}N_{11}O_{12}$ $[M+H]^+$ 1230.87994, found 1230.87887 (Δ = -0.8 ppm)
***Z*:*E* ratio 27:100 (~1:4)** as determined by quantitative ^{13}C NMR acquired at 176 MHz (interpulse delay was set to be 5x the longest value measured, and quantitative ^{13}C acquisition parameter set was used to mitigate ^{13}C-^{1}H NoE's affecting the result).

(3S,6S,9S,12R,15S,18S,21R,24S,30S,33S)-30-ethyl-33-((1R,2R,*E*)-1-hydroxy-2-methylhept-5-en-1-yl-7,7,7-d_3)-6,9,18,24-tetraisobutyl-3,21-diisopropyl-1,4,7,10,12,15,19,25,28-nonamethyl-1,4,7,10,13,16,19,22,25,28,31-undecaazacyclotritriacontan-2,5,8,11,14,17,20,23,26,29,32-undecaone (*homo* d_3-Cyclosporine A – 23)

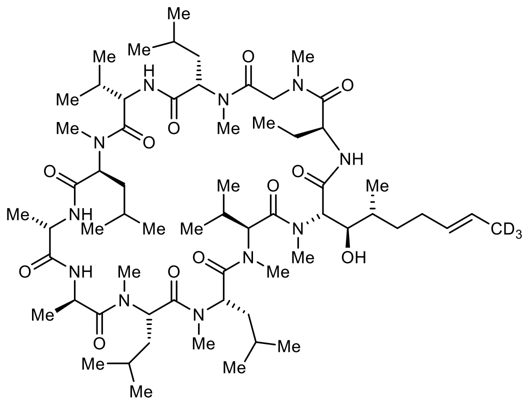

A 10 mL microwave vial was charged with a magnetic stirrer bar, 1CTR **1e** (134 mg, 0.5 mmol, 5.00 equiv), Cyclosporine A (120 mg, 0.1 mmol, 1.00 equiv) and nitro-Grela-I_2 SIPr (CAS: 1874265-00-5) catalyst (18.8 mg, 0.02 mmol, 0.20 equiv.) The vial was sealed using a crimper and back-refilled with N_2 (3-cycles). Dichloroethane (1 mL) was added via a syringe and the reaction was heated to 100 °C in an oil bath for 6 h. The reaction mixture was concentrated *in vacuo* and the residue was then purified by C18 reverse phase chromatography (0-100% Water-Acetonitrile) to remove unreacted starting material and product-containing fractions were concentrated by lyophilization.

The fractions containing the latent homolog and dimer of the 1CTR **1e**, which co-eluted, were transferred to a 10 mL microwave vial containing a magnetic stir bar. The vial was sealed using a crimper and back-refilled with N_2 (3-cycles). Dichloromethane (1 mL) was added via a syringe and the reaction was heated to 40 °C. Citric acid (288 mg, 1.5 mmol, 3.00 equiv) and TBAF·$3H_2O$ (197 mg, 0.63 mmol, 1.25 equiv) were dissolved in 2 mL MeOH. The solution was added through the septum of the microwave vial and the reaction was heated for 16 h, followed by the standard work-up procedure (3.5). The residue was purified by C18 reverse column chromatography (0-100% Water-Acetonitrile). Product-containing fractions were lyophilized to give the product as an off-white powder white solid (54.8 mg, 0.045 mmol, 45%).

^{1}H NMR (700 MHz, $CDCl_3$) δ 7.98 (d, *J* = 9.9 Hz, 1H), 7.68 (d, *J* = 7.3 Hz, 1H), 7.47 (d, *J* = 8.3 Hz, 1H), 7.18 (d, *J* = 7.9 Hz, 1H), 5.71 (dd, *J* = 10.9, 4.3 Hz, 1H), 5.51 – 5.46 (m, 1H), 5.36 (d, *J* = 15.2 Hz, 1H), 5.34 – 5.26 (m, 2H), 5.13 (d, *J* = 10.9 Hz, 1H), 5.08 – 5.01 (m, 2H), 5.00 – 4.95 (m, 1H), 4.84 (p, *J* = 7.1 Hz, 1H), 4.73 (d, *J* = 13.8 Hz, 1H), 4.69 – 4.63 (m, 1H), 4.53 (p, *J* = 7.4 Hz, 1H), 3.91 – 3.81 (m, 1H), 3.75 (q, *J* = 6.7 Hz, 1H), 3.51 (s, 2H), 3.40 (s, 3H), 3.28 (s, 3H), 3.11 (s, 6H), 2.71 (s, 3H), 2.69 (s, 3H), 2.50 – 2.38 (m, 1H), 2.21 – 2.00 (m, 7H), 1.97 (td, *J* = 10.6, 5.3 Hz, 1H), 1.89 (dp, *J* = 15.9, 5.8 Hz, 1H), 1.51 – 1.45 (m, 1H), 1.43 – 1.37 (m, 2H), 1.35 (d, *J* = 7.3 Hz, 3H), 1.29 – 1.23 (m, 5H), 1.08 (d, *J* = 6.5 Hz, 3H), 1.05 – 0.98 (m, 11H), 0.98 – 0.92 (m, 15H), 0.92 – 0.82 (m, 21H), 0.78 – 0.72 (m, 3H).

^{13}C NMR (176 MHz, $CDCl_3$) δ 173.9, 173.8, 173.7, 173.7, 173.5, 173.5, 171.6, 171.3, 171.3, 171.1, 170.5, 170.4, 170.4, 170.2, 170.1, 170.1, 131.9 (*E*-isomer), 130.9 (*Z*-isomer), 124.2 (*E*-

isomer), 123.3 (*Z*-isomer), 75.1, 59.0, 57.9, 57.6, 55.5, 55.4, 55.4, 55.2, 50.3, 48.7, 48.7, 48.5, 48.5, 48.2, 45.1, 40.5, 39.5, 39.0, 37.4, 36.0, 34.0, 34.0, 32.6, 31.6, 31.3, 31.1, 30.2, 29.8, 29.8, 29.7, 29.5, 29.2, 25.3, 25.3, 25.0, 24.9, 24.9, 24.6, 24.5, 24.4, 23.9, 23.8, 23.7, 23.5, 23.5, 23.5, 23.4, 21.9, 21.8, 21.2, 21.2, 20.4, 20.4, 19.9, 18.7, 18.4, 18.3, 18.2, 18.2, 17.1, 17.0, 15.9, 9.9.

HRMS (ESI+) m/z calculated for $C_{63}H_{111}D_3N_{11}O_{12}$ $[M+H]^+$ 1219.88312, found 1219.88244 (Δ = -0.6 ppm)

***Z*:*E* ratio 22:100 (~1:5)** as determined by quantitative ^{13}C NMR acquired at 176 MHz (interpulse delay was set to be 5x the longest value measured, and quantitative ^{13}C acquisition parameter set was used to mitigate ^{13}C-^{1}H NoE's affecting the result).

(3S,6S,9S,12R,15S,18S,21R,24S,30S,33S)-30-ethyl-33-((1R,2R,*Z*)-6-fluoro-1-hydroxy-2-methylhex-5-en-1-yl)-6,9,18,24-tetraisobutyl-3,21-diisopropyl-1,4,7,10,12,15,19,25,28-nonamethyl-1,4,7,10,13,16,19,22,25,28,31-undecaazacyclotritriacontan-2,5,8,11,14,17,20,23,26,29,32-undecaone (*fluoro* Cyclosporine A – 24)

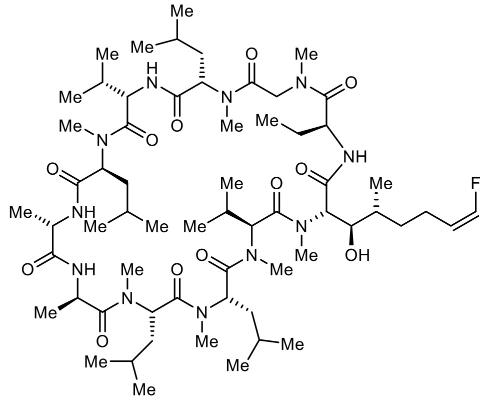

A 5 mL round-bottom flask was charged with a magnetic stirrer bar, 1CTR **1f** (67 mg, 0.25 mmol, 5.00 equiv) and CSA (60 mg, 0.05 mmol, 1.00 equiv). The round-bottom flask was fitted to a reflux condenser (Teflon tape around the ground glass joint, suba-seal on condenser outlet connected via a needle to an N_2 line) and evacuate-refilled with N_2 (3 cycles). The flask was then heated to 40 °C in an oil bath and dichloromethane (0.25 mL) was added through the top of the condenser. A stock solution of HG-II (12.6 mg/mL, 0.10 equiv) was prepared in dichloromethane and 0.25 mL of the stock solution was added, followed by dichloromethane (0.25 mL). The reaction was heated for 16 h and then concentrated *in vacuo*. The residue was purified by C18 reverse phase chromatography (0-100% Water-Acetonitrile) to remove unreacted starting material.

The fractions containing the latent homolog and dimer of the 1CTR **1f**, which co-eluted, were concentrated into a 10 mL microwave vial containing a magnetic stir bar. The vial was sealed using a crimper and back-refilled with N_2 (3-cycles). Dichloromethane (1 mL) was added via a syringe and the reaction was heated to 40 °C. Citric acid (144 mg, 0.75 mmol, 3.00 equiv) and TBAF·3H_2O (98 mg, 0.31 mmol, 1.25 equiv) were dissolved in 1 mL MeOH. The solution was added through the septum of the microwave vial and the reaction was heated for 16 h, followed by the standard work-up procedure (3.2).

It was not possible to purify *fluoro* cyclosporine A (**24**) by flash column chromatography (normal or C18 reverse phase). A 10 mg sample (16% isolated yield) of **24** was obtained from the crude by HPLC chromatography, under the following conditions:

The crude residue was purified by preparative HPLC using a Shimadzu Nexera Prep with a reversed-phase Phenomenex Luna 5 µm C18(2) 100 Å, LC Column (150 x 10 mm), at 75 °C, eluting with a gradient of 40-100% solvent A-Solvent B over 20 minutes (solvent A: 0.1% (v/v) TFA in H_2O, solvent B: 0.05% (v/v) TFA in MeCN). Product-containing fractions were lyophilized to give the product as an off-white powder (10 mg, 8.2 µmol, 16%).

^{1}H NMR (700 MHz, $CDCl_3$) δ 8.00 (d, *J* = 9.7 Hz, 1H), 7.72 (d, *J* = 7.5 Hz, 1H), 7.50 (d, *J* = 8.1 Hz, 1H), 7.20 (d, *J* = 8.4 Hz, 1H), 6.57 – 6.32 (m, 1H), 5.73 (dd, *J* = 10.9, 4.4 Hz, 1H), 5.49 (d, *J* = 6.2 Hz, 1H), 5.36 – 5.27 (m, 1H), 5.18 – 4.95 (m, 5H), 4.90 – 4.82 (m, 1H), 4.75 (d, *J* = 14.0 Hz, 1H), 4.70 – 4.51 (m, 3H), 3.82 – 3.77 (m, 1H), 3.53 (d, *J* = 1.5 Hz, 3H), 3.41 (s, 3H), 3.31 – 3.28 (m, 3H), 3.13 (d, *J* = 2.0 Hz, 3H), 3.12 (s, 3H), 2.73 (s, 3H), 2.71 (d, *J* = 1.9 Hz, 3H), 2.49 – 2.41 (m, 1H), 2.29 – 2.19 (m, 1H), 2.19 – 1.96 (m, 8H), 1.76 – 1.68 (m, 3H), 1.66 – 1.59 (m, 4H), 1.53 – 1.47 (m, 1H), 1.39 – 1.34 (m, 4H), 1.28 (d, *J* = 6.8 Hz, 3H), 1.09 (dd, *J* = 6.6, 2.9 Hz, 3H), 1.07 – 1.01 (m, 10H), 1.00 – 0.94 (m, 10H), 0.93 – 0.86 (m, 16H), 0.86 – 0.83 (m, 3H), 0.82 – 0.74 (m, 3H).

^{13}C NMR (176 MHz, $CDCl_3$) δ 173.8, 173.7, 173.5, 171.6, 171.2, 171.2, 170.5, 170.4, 170.3, 170.2, 149.3, 148.1, 147.8, 146.6, 111.9, 111.9, 111.3, 111.2, 75.0, 58.8, 58.0, 57.6, 57.6, 55.5, 55.4, 55.2, 55.2, 50.3, 48.8, 48.6, 48.5, 48.2, 45.1, 40.9, 40.5, 39.5, 39.0, 37.6, 36.0, 36.0, 35.9, 35.8, 34.0, 33.9, 31.9, 31.6, 31.3, 31.2, 31.2, 30.9, 29.9, 29.8, 29.6, 29.6, 29.2, 25.3, 25.3, 25.0, 24.9, 24.9, 24.7, 24.5, 24.4, 23.9, 23.9, 23.8, 23.7, 23.6, 23.5, 23.4, 23.4, 21.8, 21.8, 21.2, 21.1, 20.5, 20.4, 20.4, 20.3, 19.9, 18.7, 18.4, 18.4, 18.2, 17.1, 17.0, 15.9, 10.0, 9.9.

^{19}F NMR (376 MHz, $CDCl_3$) δ -131.7 (*Z*-isomer), -132.0 (*E*-isomer).

HRMS (ESI+) m/z calculated for $C_{62}H_{111}O_{12}N_{11}F$ $[M+H]^+$ 1220.8392, found 1220.8402 (Δ = 0.8 ppm)

(3S,6S,9S,12R,15S,18S,21R,24S,30S,33S)-30-ethyl-33-((1R,2R)-1-hydroxy-2-methylhex-5-en-1-yl)-6,9,18,24-tetraisobutyl-3,21-diisopropyl-1,4,7,10,12,15,19,25,28-nonamethyl-1,4,7,10,13,16,19,22,25,28,31-undecaazacyclotritriacontan-2,5,8,11,14,17,20,23,26,29,32-undecaone (*iso* Cyclosporine A – 25)

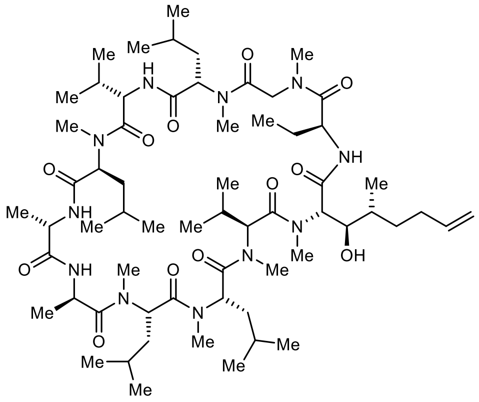

A 5 mL round-bottom flask was charged with a magnetic stirrer bar, 1CTR **1a** (250 mg, 1 mmol, 10.00 equiv) and CSA (120 mg, 0.1 mmol, 1.00 equiv). The round-bottom flask was fitted to a reflux condenser (Teflon tape around the ground glass joint, suba-seal on condenser outlet, connected via a needle to an N_2 line) and evacuate-refilled with N_2 (3 cycles). The flask was then heated to 40 °C in an oil bath and dichloromethane (0.25 mL) was added through the top of the condenser. A stock solution of HG-II (12.6 mg/mL, 0.10 equiv) was prepared in dichloromethane and 0.5 mL of the stock solution was added, followed by dichloromethane (0.25 mL). The reaction was heated for 16 h and then concentrated *in vacuo*. The residue was purified by C18 reverse phase chromatography (0-100% Water-Acetonitrile) to remove unreacted starting material.

The fractions containing the latent homolog and dimer of the 1CTR **1a**, which co-eluted, were concentrated into a 10 mL microwave vial containing a magnetic stir bar. The vial was sealed using a crimper and back-refilled with N_2 (3-cycles). Dichloromethane (1 mL) was added via a syringe and the reaction was heated to 40 °C. Citric acid (576 mg, 3 mmol, 3.00 equiv) and TBAF·$3H_2O$ (394 mg, 1.25 mmol, 1.25 equiv) were dissolved in 2 mL MeOH. The solution was added through the septum of the microwave vial and the reaction was heated for 16 h, followed by the standard work-up procedure (3.2). The residue was purified by C18 reverse column chromatography (0-100% Water-Acetonitrile). Product-containing fractions were lyophilized to give the product as an off-white powder white solid (71.8 mg, 0.060 mmol, 60%).

^{1}H NMR (500 MHz, $CDCl_3$) δ 7.98 (d, *J* = 9.9 Hz, 1H), 7.66 (d, *J* = 7.6 Hz, 1H), 7.46 (d, *J* = 8.2 Hz, 1H), 7.17 (d, *J* = 8.1 Hz, 1H), 5.75 – 5.63 (m, 2H), 5.47 (d, *J* = 5.8 Hz, 1H), 5.31 – 5.24 (m, 1H), 5.11 (d, *J* = 10.8 Hz, 1H), 5.07 – 4.99 (m, 2H), 4.99 – 4.91 (m, 2H), 4.90 – 4.78 (m, 2H), 4.71 (d, *J* = 13.9 Hz, 1H), 4.64 (t, *J* = 8.2 Hz, 1H), 4.52 (p, *J* = 7.3 Hz, 1H), 3.80 – 3.73 (m, 2H), 3.50 (s, 3H), 3.39 (s, 3H), 3.26 (s, 3H), 3.09 (s, 6H), 2.70 (s, 3H), 2.68 (s, 4H), 2.50 – 2.37 (m, 1H), 2.18 – 1.93 (m, 7H), 1.84 – 1.65 (m, 2H), 1.64 – 1.55 (m, 3H), 1.34 (d, *J* = 7.2 Hz, 4H), 1.27 – 1.23 (m, 6H), 1.07 (d, *J* = 6.6 Hz, 3H), 1.04 – 0.97 (m, 10H), 0.97 – 0.90 (m, 11H), 0.89 – 0.80 (m, 19H), 0.75 (d, *J* = 6.7 Hz, 3H).

^{13}C NMR (126 MHz, $CDCl_3$) δ 173.8, 173.8, 173.7, 173.5, 171.6, 171.3, 171.1, 170.5, 170.4, 170.2, 170.1, 139.2, 114.1, 75.0, 58.9, 57.9, 57.6, 55.5, 55.4, 55.2, 50.3, 48.7, 48.5, 48.1, 45.1, 40.5, 39.5, 39.0, 37.5, 36.0, 35.9, 34.0, 31.8, 31.6, 31.3, 31.3, 31.2, 29.8, 29.8, 29.7, 29.5, 29.2, 25.3, 24.9, 24.9, 24.6, 24.4, 23.9, 23.8, 23.7, 23.5, 23.4, 21.8, 21.2, 20.4, 19.9, 18.7, 18.3, 18.2, 17.1, 15.9, 9.9.

HRMS (ESI+) m/z calculated for $C_{62}H_{112}N_{11}O_{12}$ $[M+H]^+$ 1202.84864, found 1202.84778 (Δ = – 0.7 ppm)

(3S,6S,9S,12R,15S,18S,21R,24S,30S,33S)-30-ethyl-33-((1R,2R)-1-hydroxy-2-methylhept-6-en-1-yl)-6,9,18,24-tetraisobutyl-3,21-diisopropyl-1,4,7,10,12,15,19,25,28-nonamethyl-1,4,7,10,13,16,19,22,25,28,31-undecaazacyclotritriacontan-2,5,8,11,14,17,20,23,26,29,32-undecaone (*homoiso* Cyclosporin A – 26)

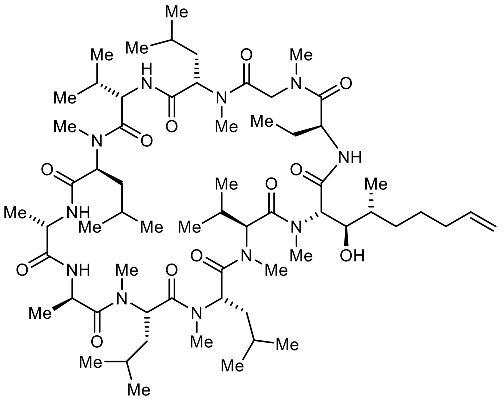

A 5 mL round-bottom flask was charged with a magnetic stirrer bar, 1CTR **1a** (63 mg, 0.25 mmol, 5.00 equiv) and *iso* CSA (**25**) (60 mg, 0.05 mmol, 1.00 equiv). The round-bottom flask was fitted to a reflux condenser (Teflon tape around the ground glass joint, suba-seal on condenser outlet connected via a needle to an N_2 line) and evacuate-refilled with N_2 (3 cycles). The flask was then heated to 40 °C in an oil bath and dichloromethane (0.25 mL) was added through the top of the condenser. A stock solution of HG-II (12.6 mg/mL, 0.10 equiv) was prepared in dichloromethane and 0.25 mL of the stock solution was added, followed by dichloromethane (0.25 mL). The reaction was heated for 16 h and then concentrated *in vacuo*. The residue was purified by C18 reverse phase chromatography (0-100% Water-Acetonitrile) to remove unreacted starting material.

The fractions containing the latent homolog and dimer of the 1CTR **1a**, which co-eluted, were concentrated into a 10 mL microwave vial containing a magnetic stir bar. The vial was sealed using a crimper and back-refilled with N_2 (3-cycles). Dichloromethane (1 mL) was added via a syringe and the reaction was heated to 40 °C. Citric acid (144 mg, 0.75 mmol, 3.00 equiv) and TBAF·$3H_2O$ (98 mg, 0.31 mmol, 1.25 equiv) were dissolved in 2 mL MeOH. The solution was added through the septum of the microwave vial and the reaction was heated for 16 h, followed by the standard work-up procedure (3.2). The residue was purified by C18 reverse column chromatography (0-100% Water-Acetonitrile). Product-containing fractions were lyophilized to give the product as an off-white powder white solid (34.9 mg, 0.029 mmol, 57%).

^{1}H NMR (700 MHz, $CDCl_3$) δ 7.97 (d, *J* = 9.9 Hz, 1H), 7.69 (d, *J* = 7.5 Hz, 1H), 7.48 (d, *J* = 8.2 Hz, 1H), 7.17 (d, *J* = 8.0 Hz, 1H), 5.82 (ddt, *J* = 16.8, 10.2, 6.6 Hz, 1H), 5.73 (dd, *J* = 11.0, 4.4 Hz, 1H), 5.50 (d, *J* = 5.8 Hz, 1H), 5.31 (dd, *J* = 11.7, 4.0 Hz, 1H), 5.15 (d, *J* = 10.9 Hz, 1H), 5.10 – 5.03 (m, 2H), 5.02 – 4.96 (m, 2H), 4.95 – 4.92 (m, 1H), 4.87 – 4.82 (m, 1H), 4.75 (d, *J* = 13.9 Hz, 1H), 4.67 (dd, *J* = 9.8, 8.2 Hz, 1H), 4.55 (p, *J* = 7.3 Hz, 1H), 3.75 (dd, *J* = 8.2, 5.8 Hz, 1H), 3.54 (s, 3H), 3.41 (s, 3H), 3.29 (s, 3H), 3.13 (s, 3H), 3.12 (s, 3H), 2.73 (s, 3H), 2.71 (s, 3H), 2.49 – 2.41 (m, 1H), 2.19 – 2.04 (m, 4H), 2.03 – 1.91 (m, 3H), 1.83 – 1.68 (m, 4H), 1.67 – 1.58 (m, 3H), 1.53 – 1.40 (m, 2H), 1.39 – 1.22 (m, 11H), 1.10 (d, *J* = 6.5 Hz, 3H), 1.04 (q, *J* = 6.6 Hz, 9H), 0.99 – 0.94 (m, 11H), 0.93 – 0.83 (m, 19H), 0.74 (d, *J* = 6.7 Hz, 3H).

^{13}C NMR (176 MHz, $CDCl_3$) δ 173.9, 173.8, 173.7, 173.5, 171.6, 171.3, 171.1, 170.6, 170.4, 170.2, 170.1, 139.1, 114.2, 75.3, 58.9, 57.8, 57.6, 55.5, 55.4, 55.3, 50.3, 48.7, 48.6, 48.2, 45.1, 40.5, 39.5, 39.0, 37.7, 36.3, 36.0, 34.3, 34.2, 32.2, 31.6, 31.3, 31.2, 29.8, 29.5, 29.2, 26.6, 25.3, 24.9, 24.9, 24.6, 24.4, 23.9, 23.8, 23.7, 23.6, 23.5, 21.9, 21.8, 21.2, 20.4, 19.9, 18.7, 18.4, 18.2, 17.1, 16.0, 10.0.

HRMS (ESI+) m/z calculated for $C_{63}H_{114}N_{11}O_{12}$ $[M+H]^+$ 1216.8643, found 1216.8661 (Δ = –1.5 ppm)

6. Biochemical assays

HPLC purification

Prior to submitting Cyclosporine-A homologs (**21**, **22, 23**) to biochemical assays (*vide infra*), samples of the *E*-isomers were obtained using the following HPLC conditions:

Preparative HPLC was performed using a Shimadzu Nexera Prep with a reversed-phase Phenomenex Luna 5 µm C18(2) 100 Å, LC Column (150 x 10 mm), at 75 °C, eluting with an isocratic gradient of 70% Solvent B over 20 minutes (solvent A: 0.1% (v/v) TFA in H_2O, solvent B: 0.05% (v/v) TFA in MeCN). Product-containing fractions were lyophilized to give the product as an off-white powder.

In both cases, the *E*-isomer was found to be separable (<5% *Z*-isomer by HPLC) and no detectable quantities of the Z-isomer were observable by ^{13}C NMR analysis of the purified homologs.

Fig. S4. (**a**) HPLC trace for *E*-homo Cyclosporine-A (**21**); (**b**) superimposed HPLC traces for Cyclosporine-A, *E-homo* Cyclosporine-A (**21**), *E-bishomo* Cyclosporine-A (**22**).

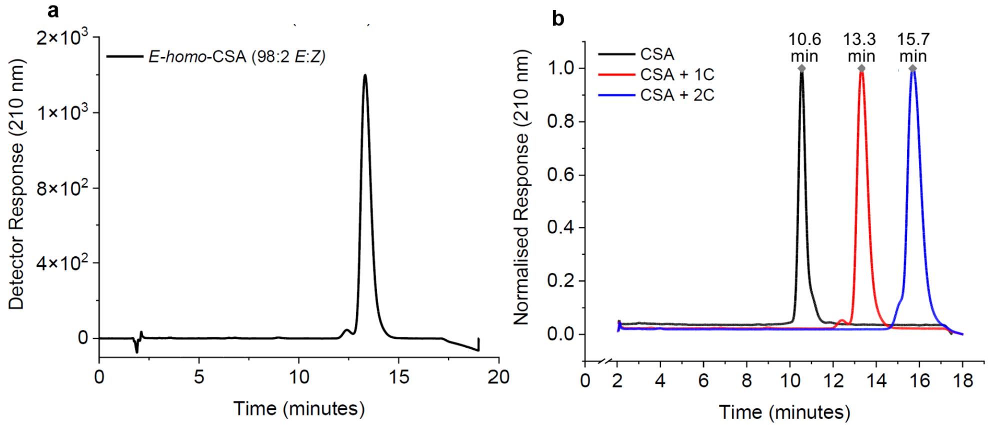

Lipophilicity (cLogP) and Intrinsic Solubility (LogS)

Table S8. cLogP and logS values calculated using ChemAxon cheminformatics software (Chemicalize).[10]

Compound	cLogP	LogS
Cyclosporine A	3.6	– 6.5
+ 1C (**21**)	4.1	– 6.8
+ 2C (**22**)	4.5	– 7.1

The observed increase in calculated lipophilicity (Table S8) upon one-carbon homologation corroborates the HPLC data for homologs **21** and **22** (**Fig. S4-b**). Retention time was found to increase by ~ 2.5 minutes upon each homologation event.[11]

IL-2 release assay

Interleukin-2 (IL-2) release assays were conducted by Eurofins Pan Labs to determine ability for the analogues to inhibit IL-2 secretion from Jurkat cells following stimulation with anti-CD3 and anti-CD28 for a period of 24 hours. Data shown are means of three replicates normalized to the vehicle control

Experimental Protocol:

1) Cryopreserved Jurkat cells were thawed and cultured according to established protocols for four days at 37°C, 5% CO2 prior to assay.
2) Jurkat cells were harvested and seeded into 96-well plates at a density of 2x105 cells/well with 150 μL/well of culture medium (RPMI 1640 containing 10% heat-inactivated FBS, 1% penicillin/streptomycin, and 2 mM L-glutamine).
3) Cells were incubated at 37°C, 5% CO2 for 1 hour prior to the addition of test agents.
4) The test agents were serially diluted in DMSO to 1000X assay concentrations, then further diluted to 20X assay concentrations in culture medium. The reference inhibitor Cyclosporin A (Sigma Aldrich catalog #C1832) was diluted to 1000X assay concentrations in DMSO before dilution to 20X assay concentration in culture medium.
5) The test agents, reference inhibitor, and diluted vehicle controls were added to the cells in volumes of 10 μL in triplicate and incubated for 1 hour at 37°C, 5% CO2.
6) The agonists anti-CD3 (clone OKT3) and anti-CD28 superagonist were diluted to 5X assay concentrations in culture medium. The corresponding isotype controls were diluted to 5X assay concentrations in culture medium.
7) Diluted anti-CD3/anti-CD28 was added to test agent and positive control wells in volumes of 40 μL according to the plate map. Isotype controls were added to the isotype control wells. Culture medium was added to vehicle and unstimulated control wells. This resulted in final assay volumes of 200 μL and control concentrations of 50 ng/mL cyclosporin A (reference inhibitor), 0.1% DMSO (vehicle), 15 μg/mL anti-CD3, and 15 μg/mL anti-CD28.
8) Cells were incubated for 24 hours at 37°C, 5% CO2.
9) At the end of the incubation period, plates were centrifuged at 200g for 10 minutes. Cell culture supernatants were collected and stored at -80°C until needed for analysis.

Data Analysis and Analyte Limits of Detection

IL-2 cytokine levels in cell culture supernatants were determined using Luminex methodology per the manufacturer's protocol using the Human Cytokine/Chemokine/Growth Factor Panel A Kit from Millipore Sigma (catalog #HCYTA-60K) with standard ranges and limits of detection in Table S9. Levels of each cytokine were interpolated from standard curves using non-linear regression analysis in Belysa Software (Millipore Sigma). The raw data in the form of median fluorescence unit was interpolated into test concentrations (pg/mL). The interpolated data were then normalized to the vehicle control

Table S9. Analyte limit of detection

Analyte	Lowest standard (pg/mL)	Highest standard (pg/mL)	Sensitivity (pg/mL)
IL-2	0.64	10,000	0.28

Results – Normalized Cytokine Secretion Summary

Reported mean values (pg/mL) are from three biological replicates normalized to the vehicle control.

Fig. S5. IL-2 release by Jurkat cells following stimulation with anti-CD3/anti-CD28 and treatment with the indicated test agent for 24 hours. Error bars indicate the standard error of the mean.

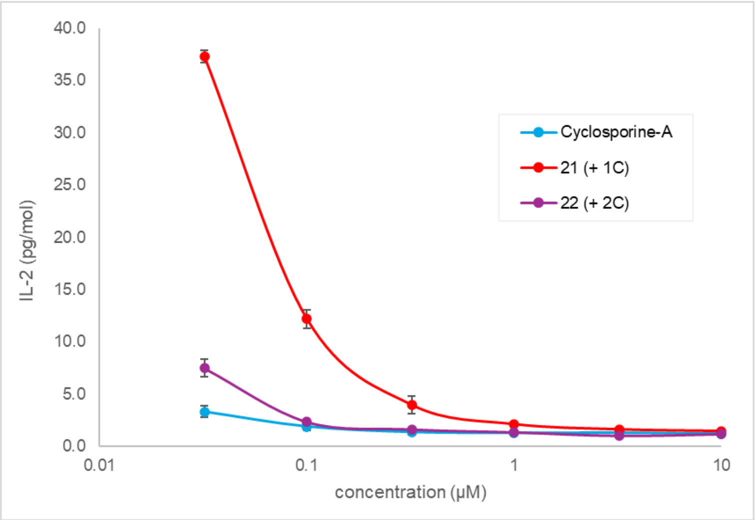

Table S10. IL-2 levels, pg/mL

Note: Replicate values listed as 0 were below the level of quantification (BLQ). Mean values in blue are approximations due to one or more replicates BLQ. † Standard Error of Mean (SEM) calculated from three independent values for each concentration.

Test Agent	Concentration	Rep 1	Rep 2	Rep 3	Mean	SEM†
CSA	10 µM	1.1	1.3	1.2	0.7	0.0580
	3.2 µM	1.0	1.2	1.7	0.8	0.2132
	1.0 µM	1.1	1.5	1.3	0.8	0.1267
	0.32 µM	0.9	1.6	1.5	0.9	0.2273
	0.10 µM	1.2	2.4	2.1	1.4	0.3434
	0.032 µM	2.2	3.5	4.2	2.8	0.5649
+1C (21)	10 µM	1.1	1.4	1.8	1.0	0.2180
	3.2 µM	1.7	1.7	1.5	1.1	0.0394
	1.0 µM	2.2	2.2	1.9	1.6	0.0937
	0.32 µM	3.5	2.8	5.5	3.5	0.8245
	0.10 µM	10.6	12.1	13.7	11.7	0.8944
	0.032 µM	38.2	36.2	37.5	36.8	0.5891
+2C (22)	10 µM	1.3	1.2	1.0	0.7	0.0811
	3.2 µM	0.8	1.2	1.0	0.5	0.1282
	1.0 µM	1.4	1.2	1.5	0.9	0.0828
	0.32 µM	1.5	1.9	1.5	1.1	0.1382
	0.10 µM	2.0	2.4	2.6	1.9	0.1933
	0.032 µM	8.5	5.8	8.1	7.0	0.8306
Controls	anti-CD3+anti-CD28 (15 µg/mL)	109.7	105.0	114.5	109.3	2.7409
	anti-CD3/CD28 + Cyclosporin (50 ng/mL)	2.4	2.8	2.6	2.1	0.1228
	mIgG1 Isotype Control	0.0	0.4	0.7	0.0	N/A
	Vehicle (0.1% DMSO)	0.4	0.4	0.6	0.0	0.0908
	Unstimulated	0.6	0.6	1.1	0.3	0.1509

Metabolic stability assay

Hepatic microsomal stability assays were conducted by Charles River Laboratories. Assays were performed in duplicate.

The stability of the test compound (1 µM) was measured following incubation at 37 °C with human cytochrome P450 (0.5 mg protein/mL) in the presence of the cofactor, NADPH. Incubates were prepared in duplicate, with aliquots removed at 0, 5, 10, 20 and 40 minutes and reactions terminated, and the compound was extracted by the addition of solvent. The disappearance of the parent compound was be monitored by LC-MS/MS and the half-life determined over the time-course of incubation. The half-life value was used to calculate the *in vitro* intrinsic clearance expressed as µL/min/mg protein.

Table S11. Metabolic Stability Data

Compound	Cofactor	Half-life (min) n=1	Half-life (min) n=2	Half-Life (min) (Mean)	Clint (uL/min/mg protn) n=1	Clint (uL/min/mg protn) n=2	Mean Clint (uL/min/mg protn)	Species (results)
Midazolam	+NADPH	5	5	5	277	277	277	Human (Mixed Gender)
Dextromethorphan	+NADPH	49	47	48	29	29	29	Human (Mixed Gender)
CSA	+NADPH	24	23	24	57	61	59	Human (Mixed Gender)
homo CSA (**21**)	+NADPH	35	30	32	40	46	43	Human (Mixed Gender)
bishomo CSA (**22**)	+NADPH	17	18	18	81	78	79	Human (Mixed Gender)
1C d_3-CSA (**23**)	+NADPH	32	31	31	44	45	44	Human (Mixed Gender)
F-CSA (**24**)	+NADPH	29	26	28	48	53	51	Human (Mixed Gender)
iso CSA (**25**)	+NADPH	24	25	25	58	55	56	Human (Mixed Gender)
homo-iso CSA (**26**)	+NADPH	20	21	21	69	65	67	Human (Mixed Gender)

PPIase TR-FRET competitive binding assay

Cyclophilin A assays were conducted by Eurofins Integrated Discovery UK Ltd (EID) to determine the inhibitory activity of the analogues against the Human Cyclophilin A in a TR-FRET Competitive Binding assay. Assays were performed in duplicate, with K_d values calculated from mean averages.

Experimental Protocol:

The 384 well TR-FRET assay determines the ability of the inhibitors to compete for CsA binding to the CypA enzyme: the CypA enzyme, tagged with a polyhistidine sequence, forms a complex with an anti-6xHis antibody labelled with a fluorescent donor, F(d), and the enzyme ligand CsA tagged with a fluorescent acceptor, F(a). Compounds are diluted from DMSO stocks to 50-fold the final concentration and then in assay buffer to 2-fold the final assay plate concentration. An equal volume of compound is added to the master mix in the assay plate containing the enzyme/antibody/ligand complex, with a final concentration of detergent of 0.01%. This is incubated for 30 mins at room temperature and the plate read at 2 wavelengths (A and B) to detect emission from F(d) and F(a), respectively. The ratio B/A is calculated, blank subtracted and either expressed as a percentage of the 'no inhibitor' control or the blank subtracted values are plotted against the inhibitor concentration in Log_{10} Molar and fitted using one site K_i nonlinear regression to determine the K_d. Where possible, compound K_ds are determined from duplicate data points. Each assay plate is set up with 'no enzyme' blanks and 'no inhibitor' controls, which are used to determine the Z' value for each plate. A CsA control curve is also included.

Fig. S6. Cyclophilin A inhibition TR-FRET K_d graphs

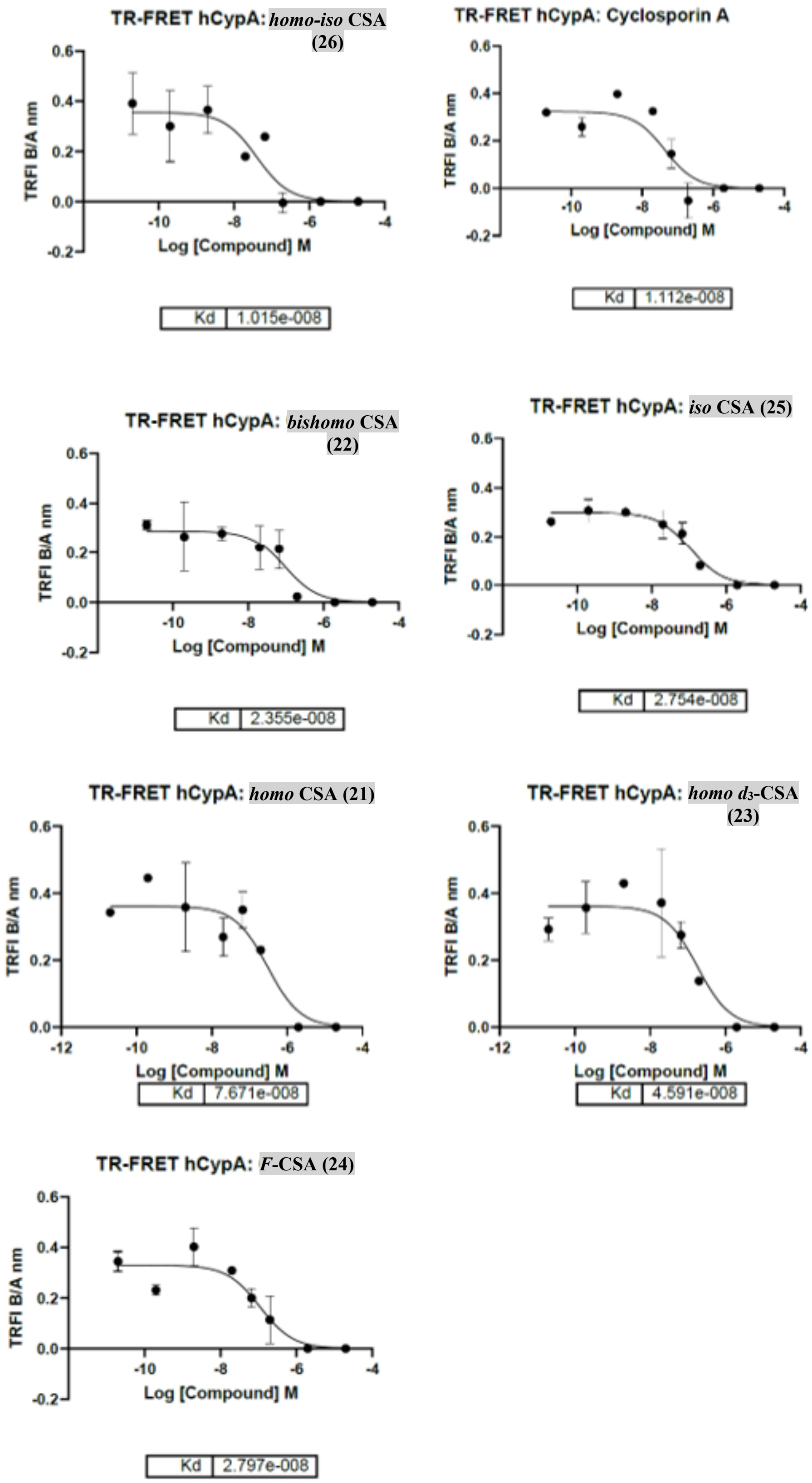

Calcineurin inhibition assay

Calcineurin inhibition assays were conducted by Eurofins Integrated Discovery UK Ltd (EID) to determine the effect of compounds on the activity of calcineurin in the presence of Cyclophilin A. Assays were performed in duplicate, with IC_{50} values calculated from mean averages.

Experimental Protocol:

Compounds were diluted with and without immunophilin (CypA) in a 96-well plate. After addition of calcineurin/calmodulin complex, the plate was incubated at 30 °C for 10 minutes. The addition of the RII phosphopeptide substrate initiated the reaction, and the plate was incubated at 30 °C for 45 minutes. The reaction was stopped by the addition of Malachite green reagent. The plate was incubated at room temperature to allow colour development (5 – 20 mins). After sufficient colour has developed, the absorbance is read at 620 nm. Blank subtracted absorbance readings are plotted against compound concentration to determine IC_{50} values or % inhibition at the top concentration.

Fig. S7. Calcineurin inhibition IC_{50} curves

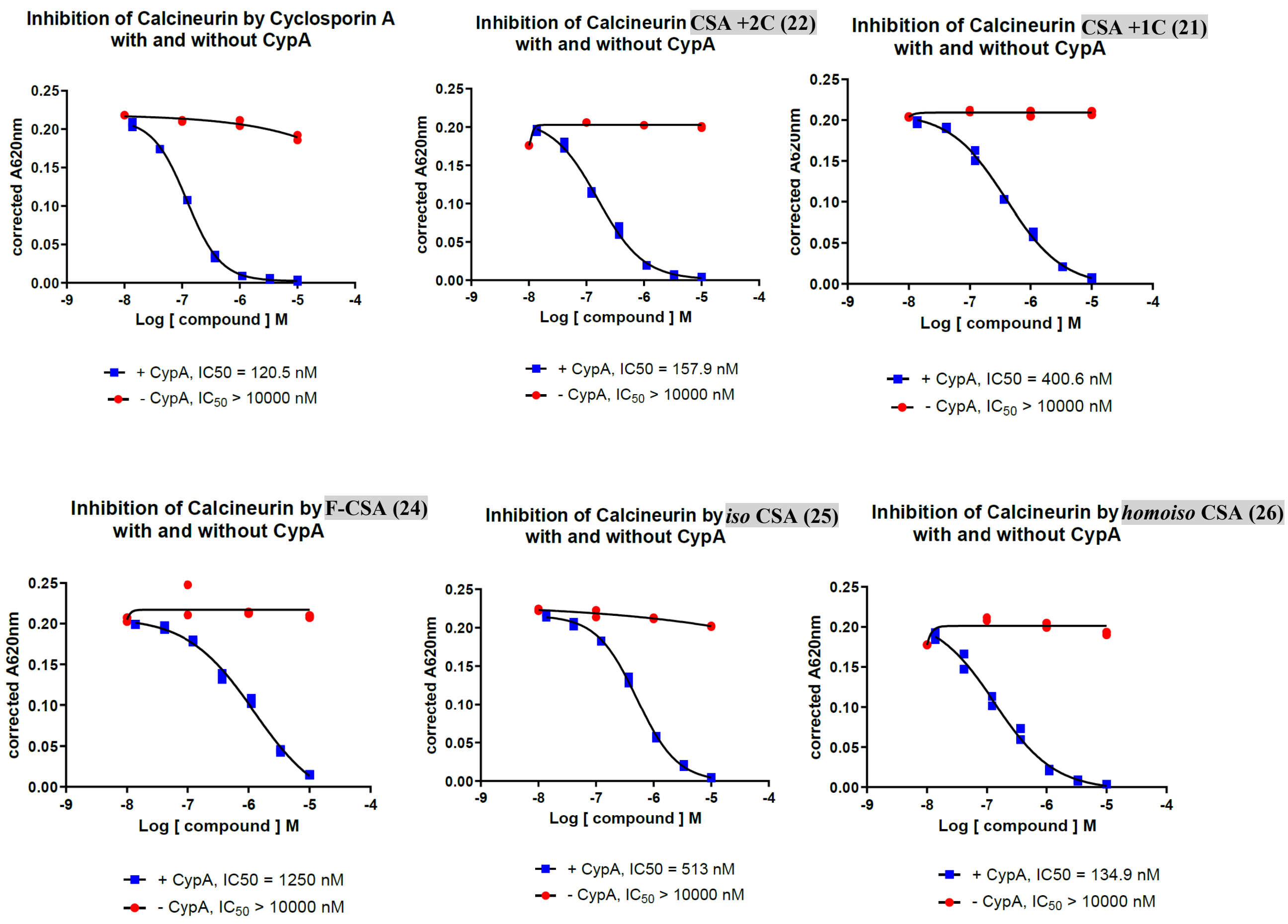

7. NMR Spectra

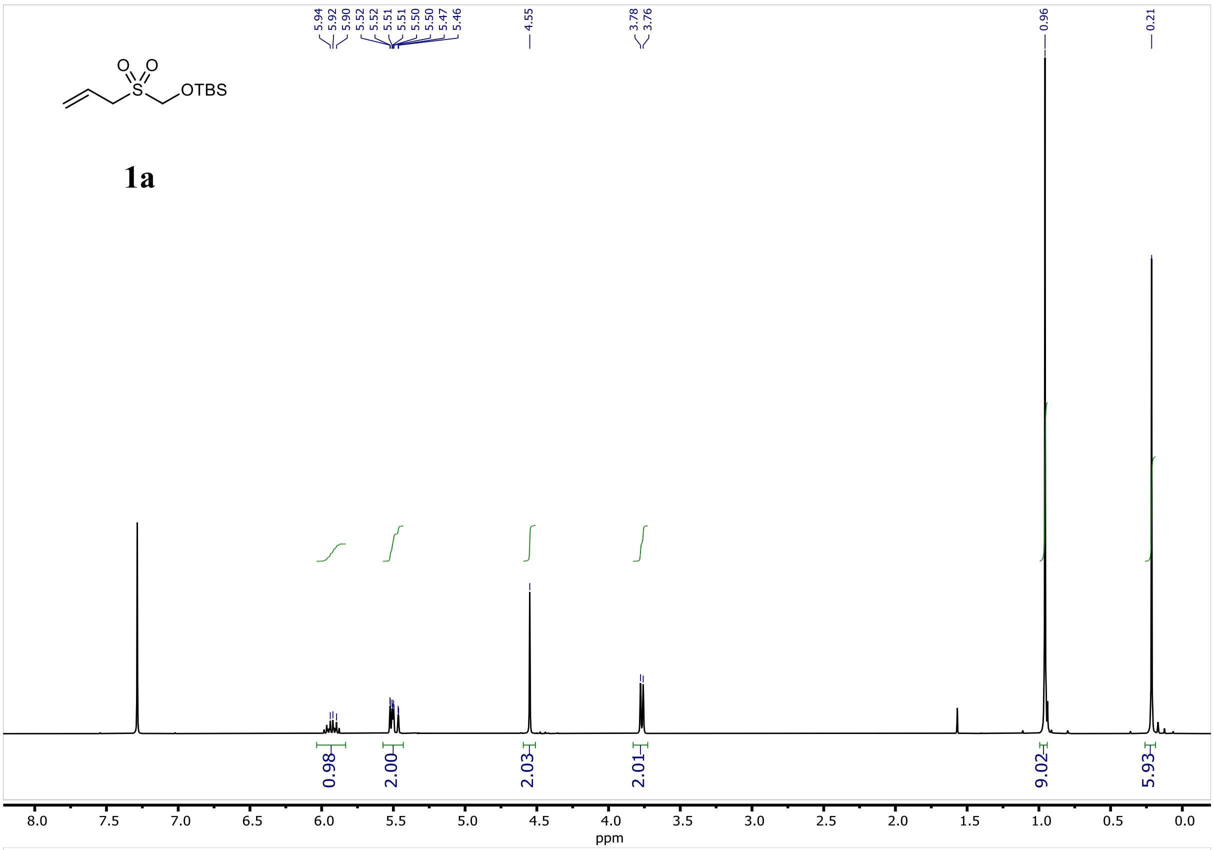

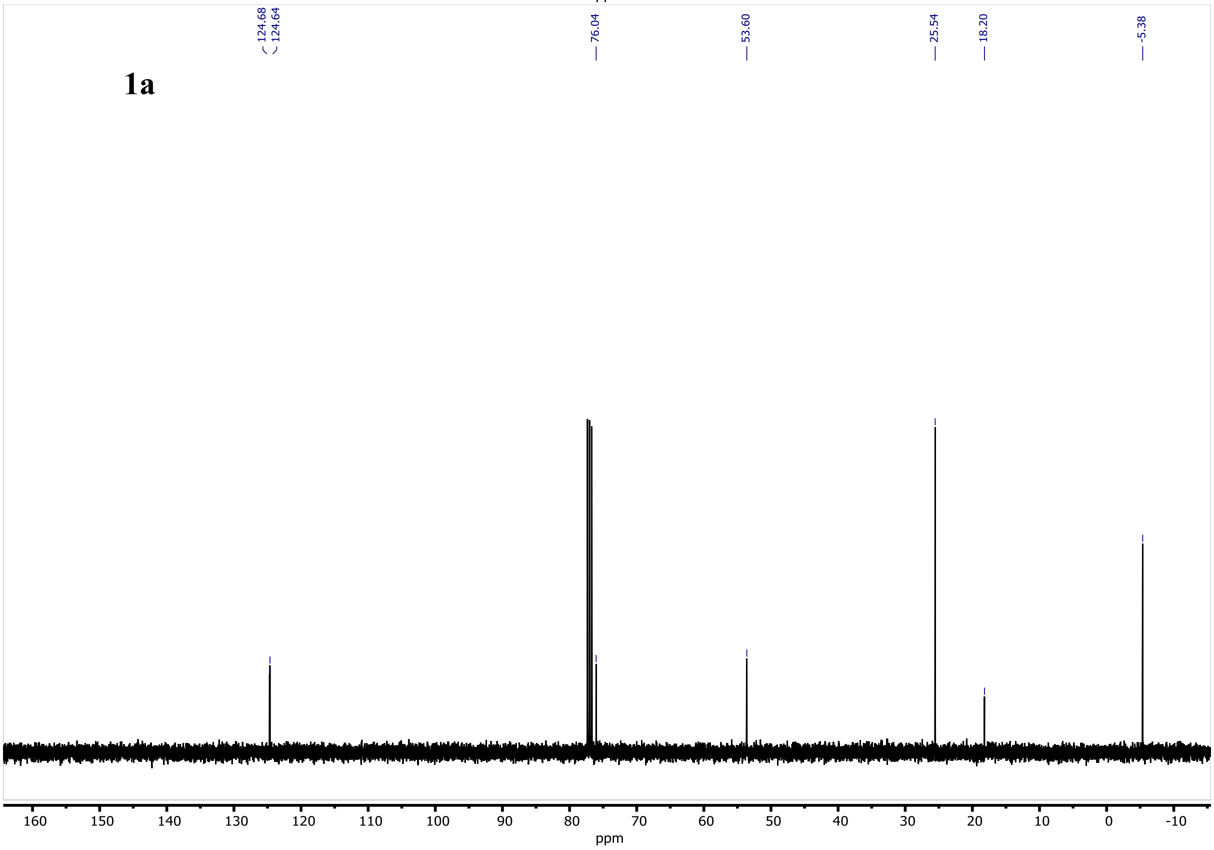

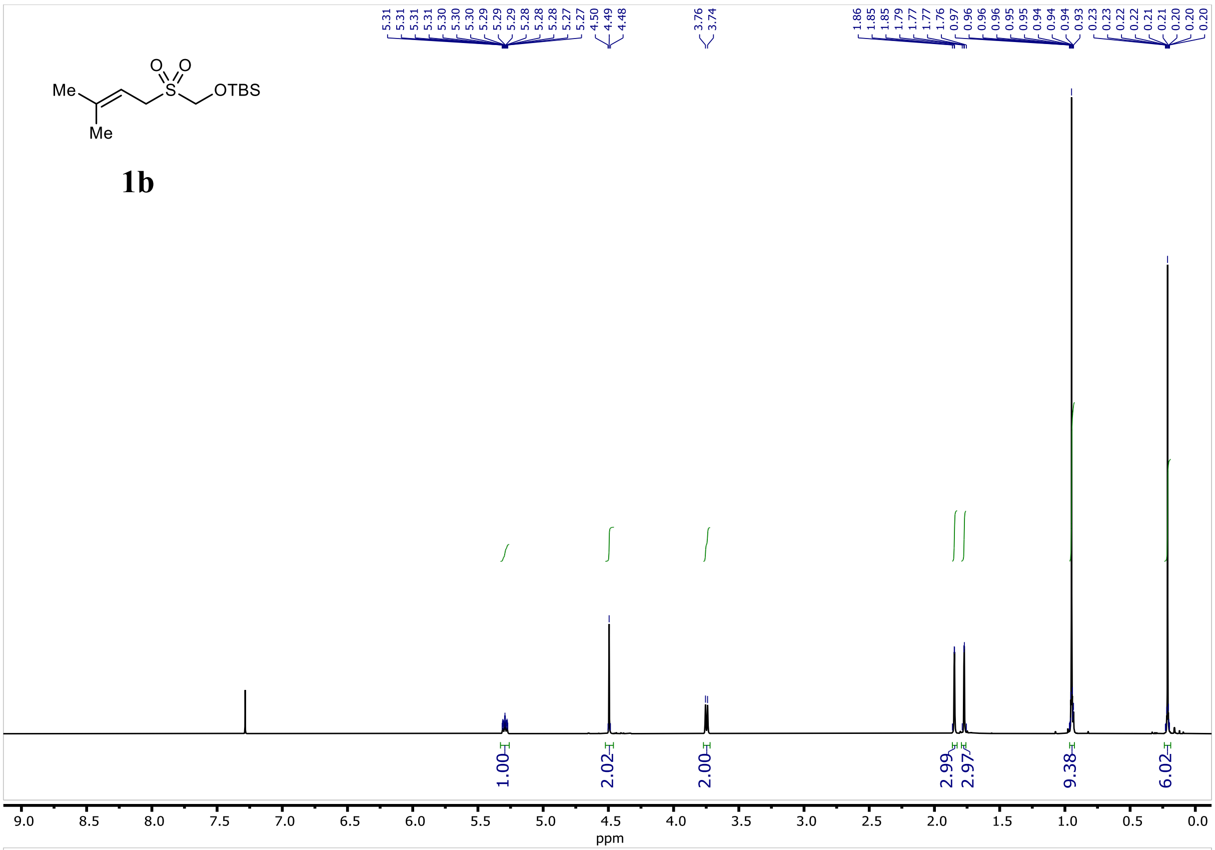
Me
Me
OTBS
1b
9.0
8.5
8.0
7.5
7.0
6.5
6.0
5.5
5.0
4.5
4.0
3.5
3.0
2.5
2.0
1.5
1.0
0.5
0.0
ppm
1.00
2.02
2.00
2.99
2.97
9.38
6.02

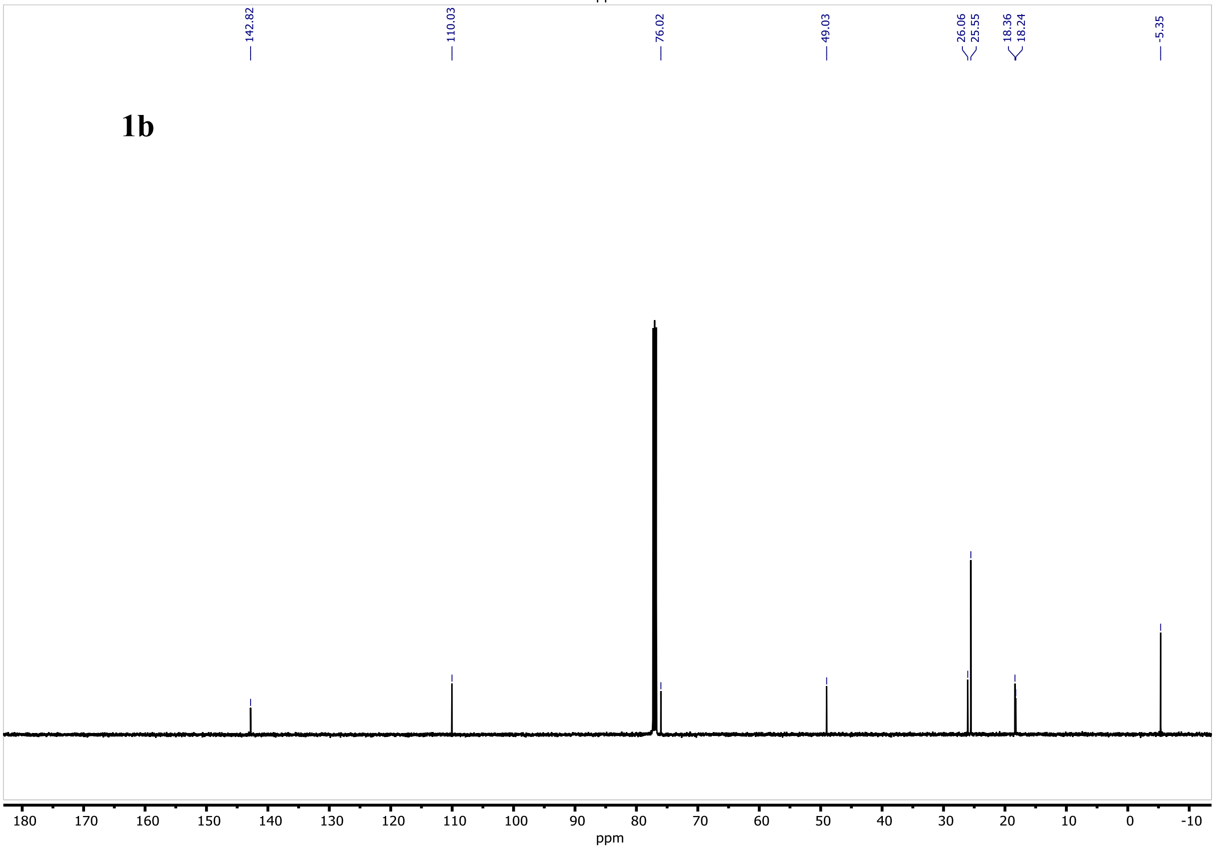
142.82
110.03
76.02
49.03
26.06
25.55
18.36
18.24
-5.35
1b
ppm

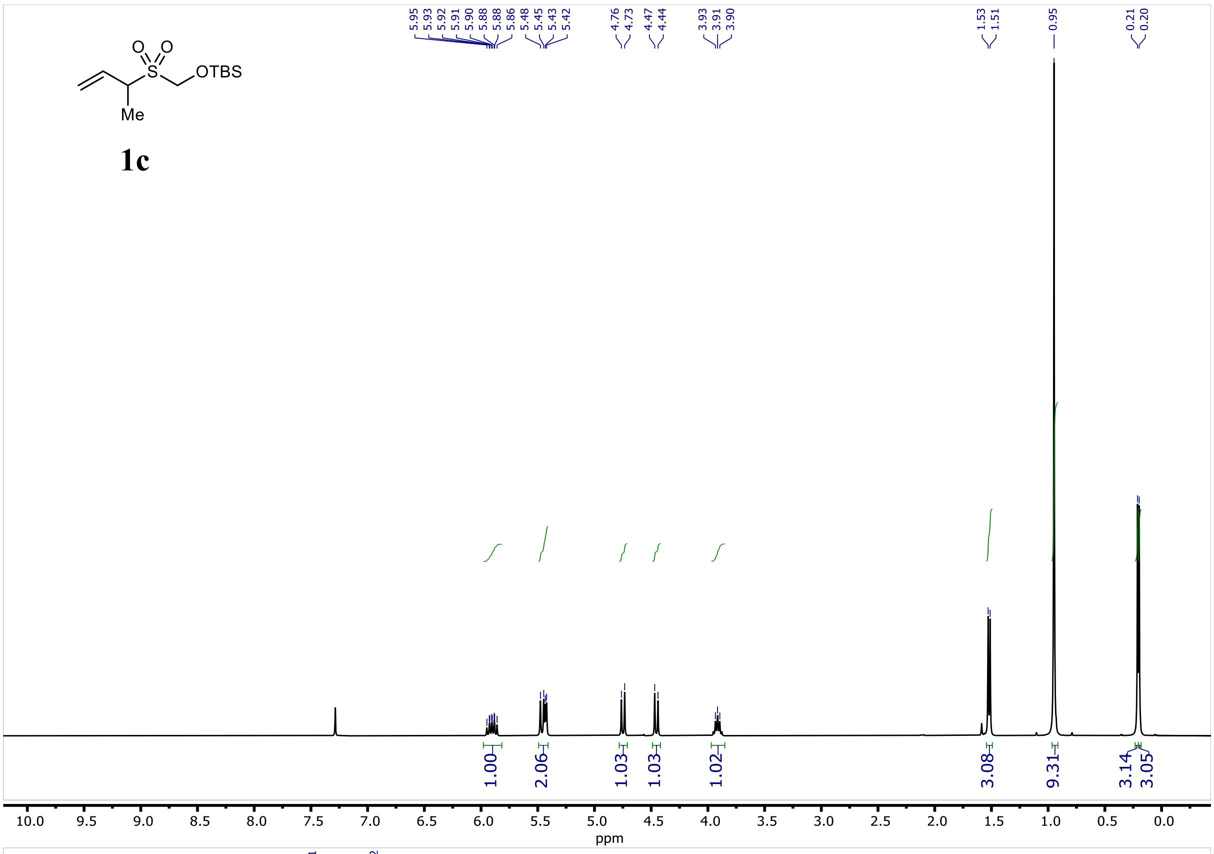
OTBS
Me
1c
5.95 5.93 5.92 5.91 5.90 5.88 5.88 5.86 5.48 5.45 5.43 5.42
4.76 4.73 4.47 4.44
3.93 3.91 3.90
1.53 1.51
0.95
0.21 0.20
1.00 2.06 1.03 1.03 1.02 3.08 9.31 3.14 3.05
ppm

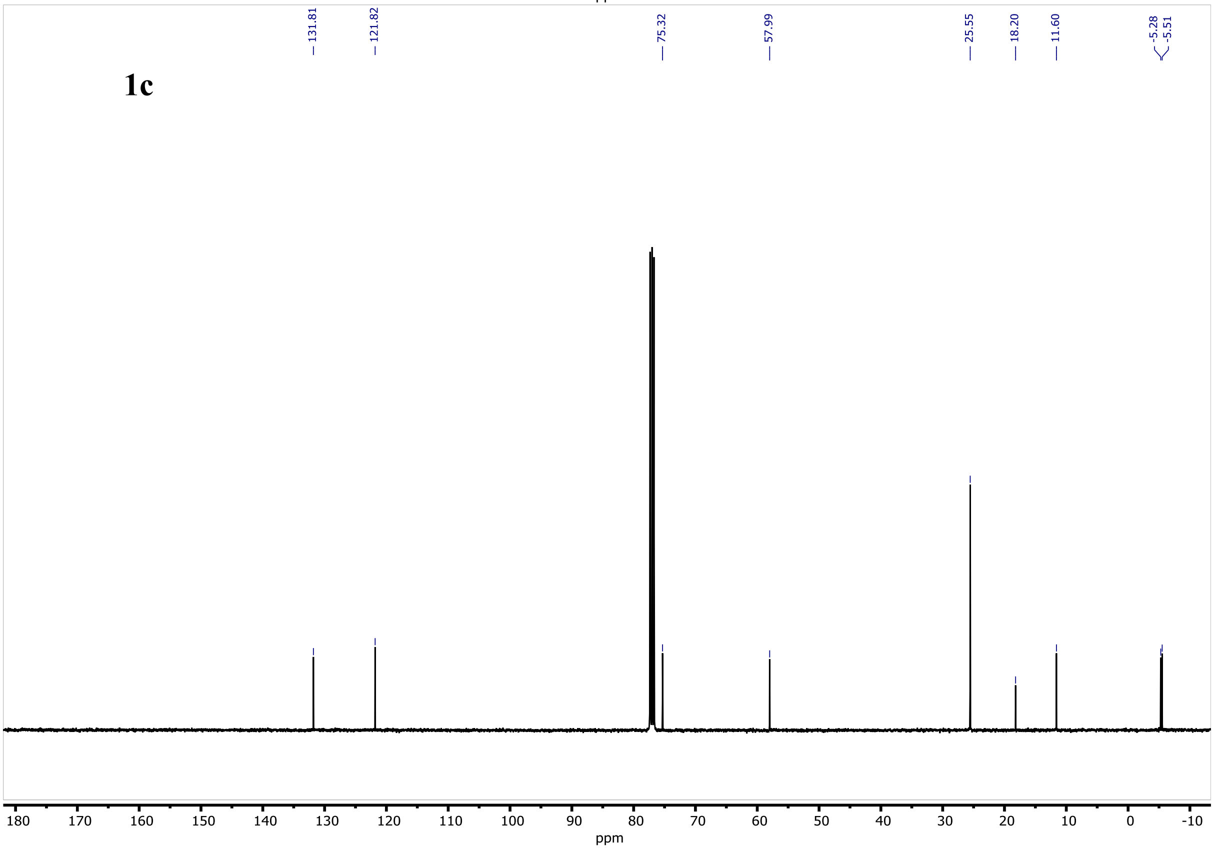
1c
131.81 121.82 75.32 57.99 25.55 18.20 11.60 -5.28 -5.51
ppm

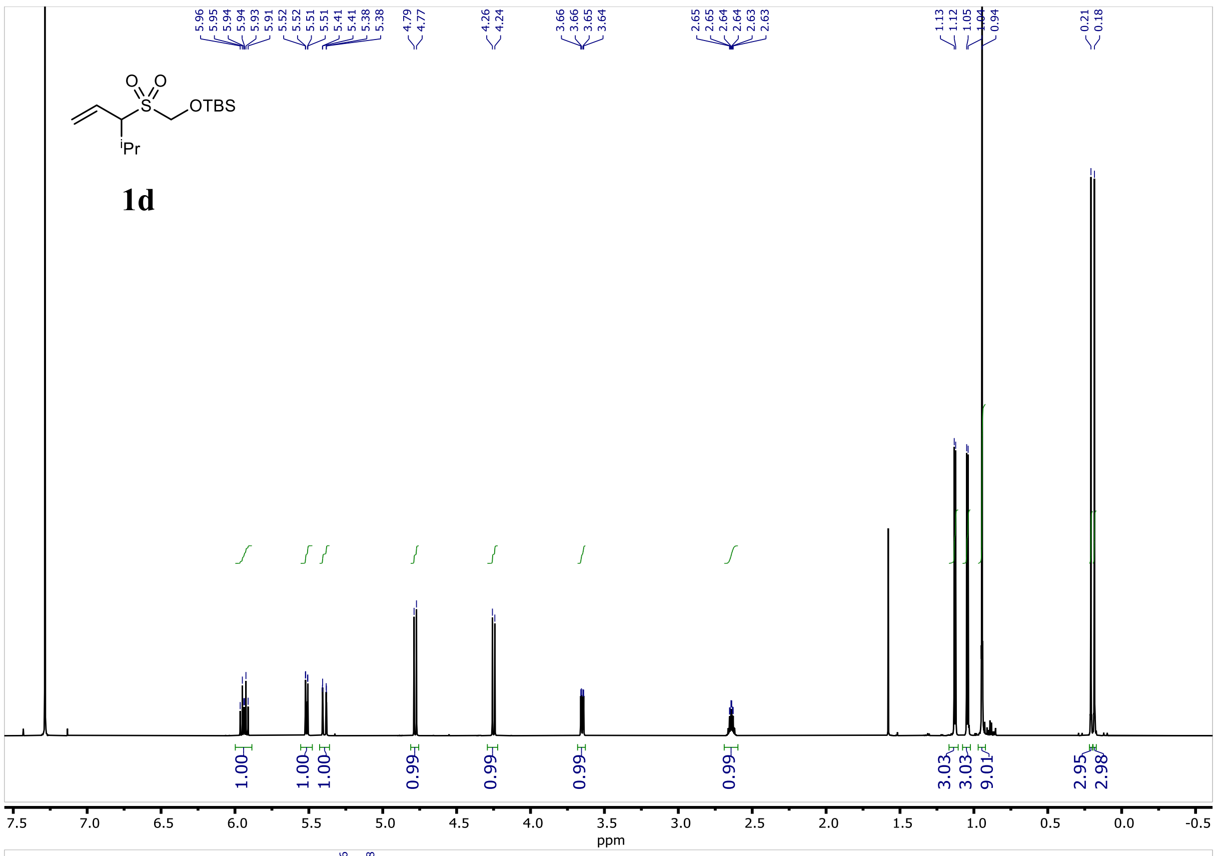

1d

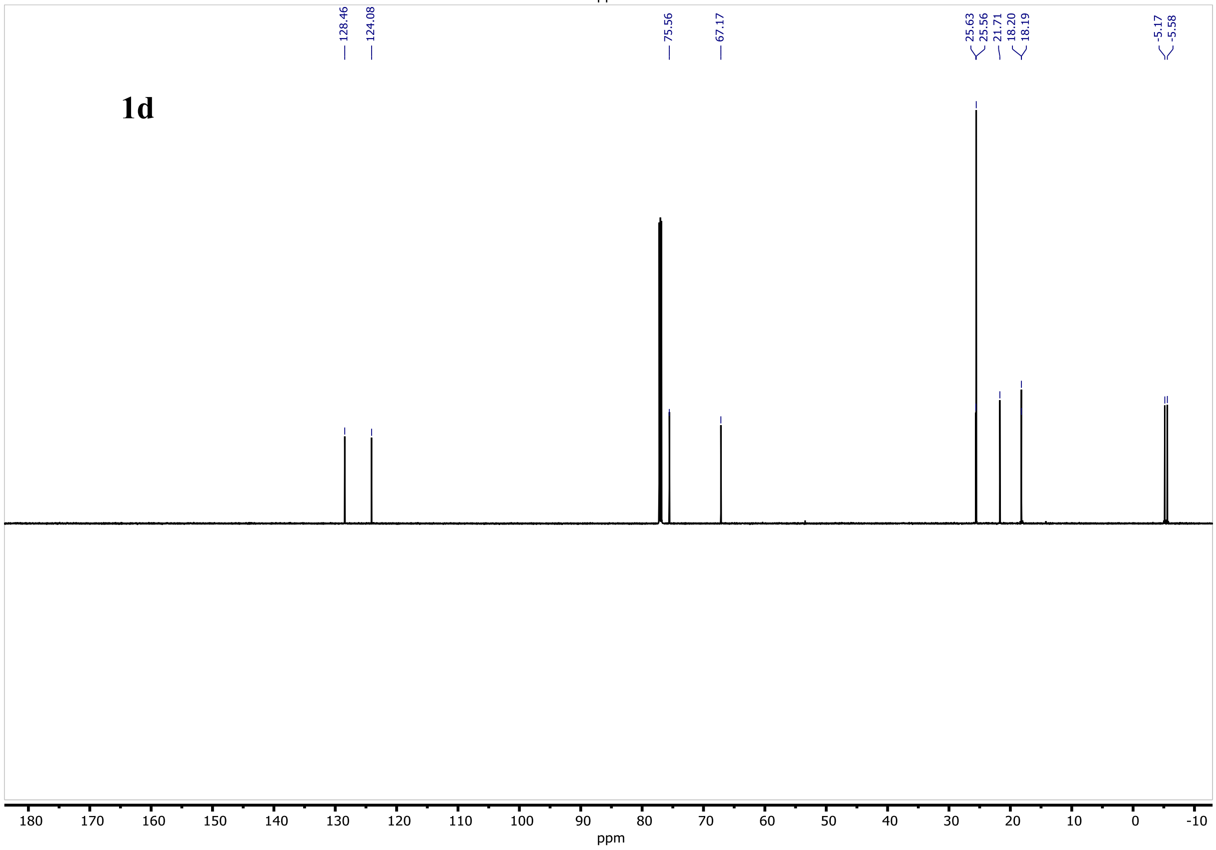

1d

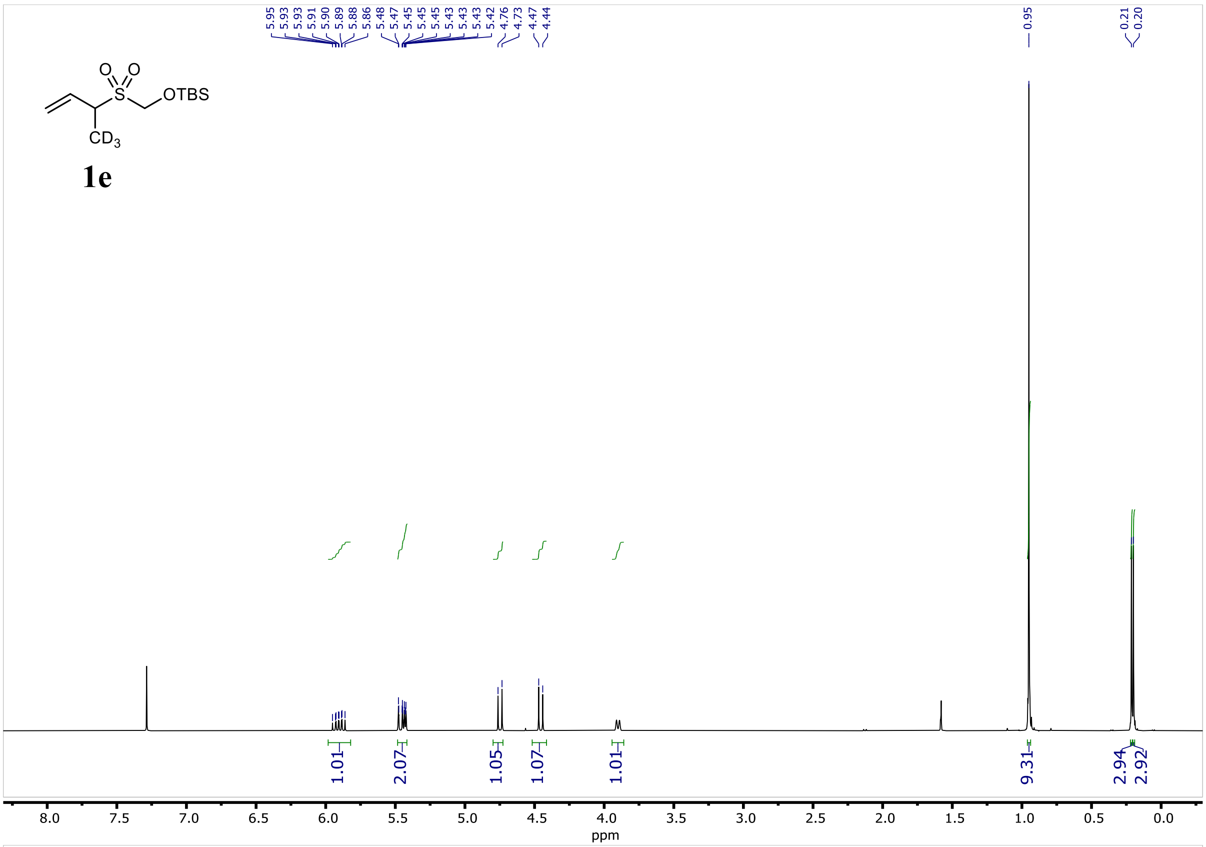
OTBS
CD_3
1e

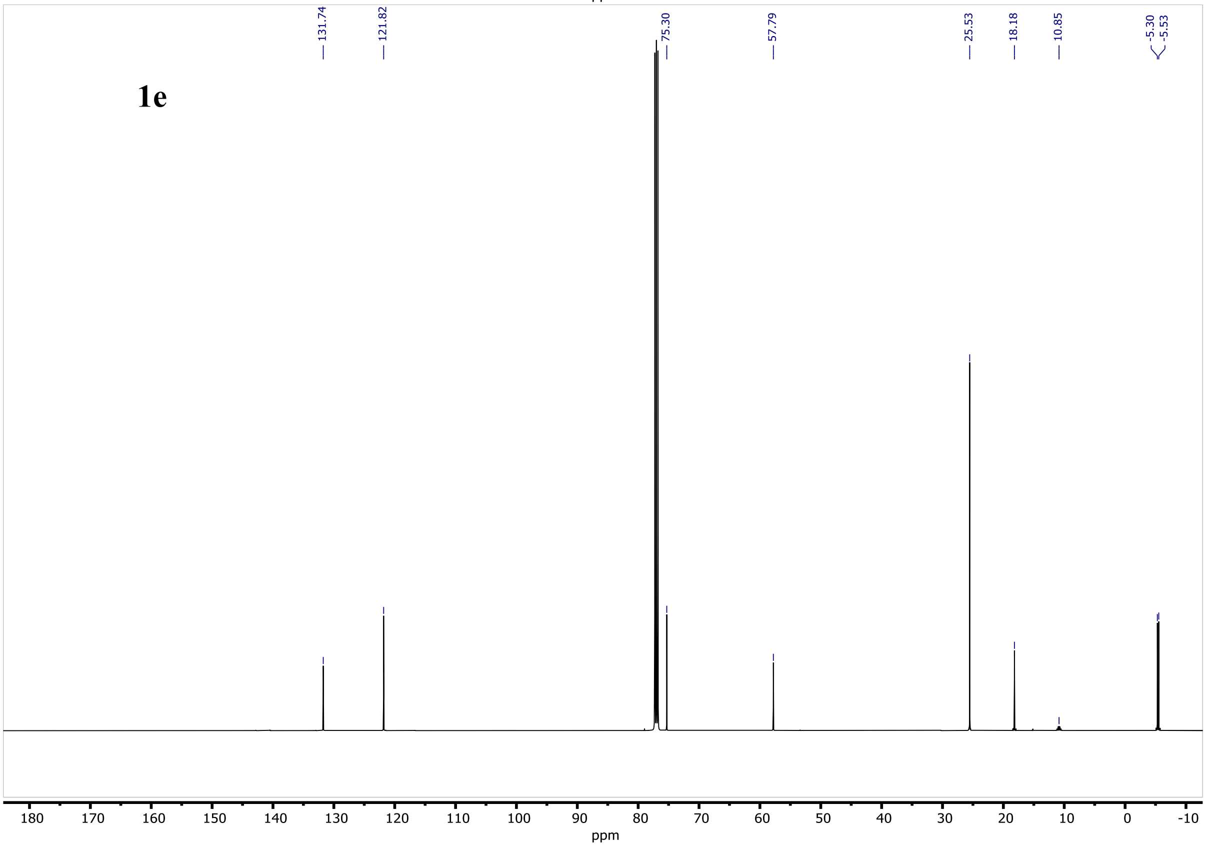
1e

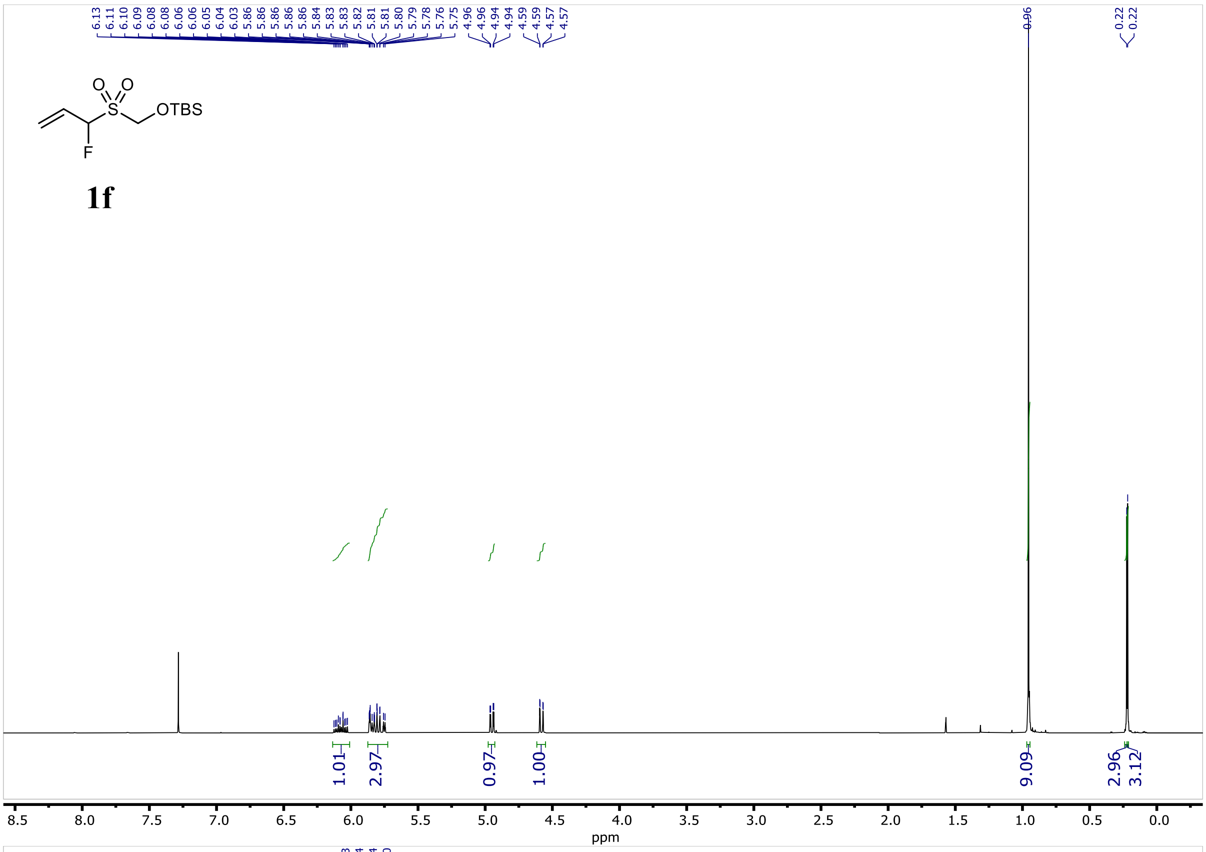
6.13 6.11 6.10 6.09 6.08 6.08 6.06 6.06 6.05 6.04 6.03 5.86 5.86 5.86 5.86 5.86 5.84 5.83 5.83 5.82 5.81 5.81 5.80 5.79 5.78 5.76 5.75 4.96 4.96 4.94 4.94 4.59 4.59 4.57 4.57
0.96
0.22 0.22
OTBS
F
1f
1.01 2.97 0.97 1.00 9.09 2.96 3.12
8.5 8.0 7.5 7.0 6.5 6.0 5.5 5.0 4.5 4.0 3.5 3.0 2.5 2.0 1.5 1.0 0.5 0.0
ppm

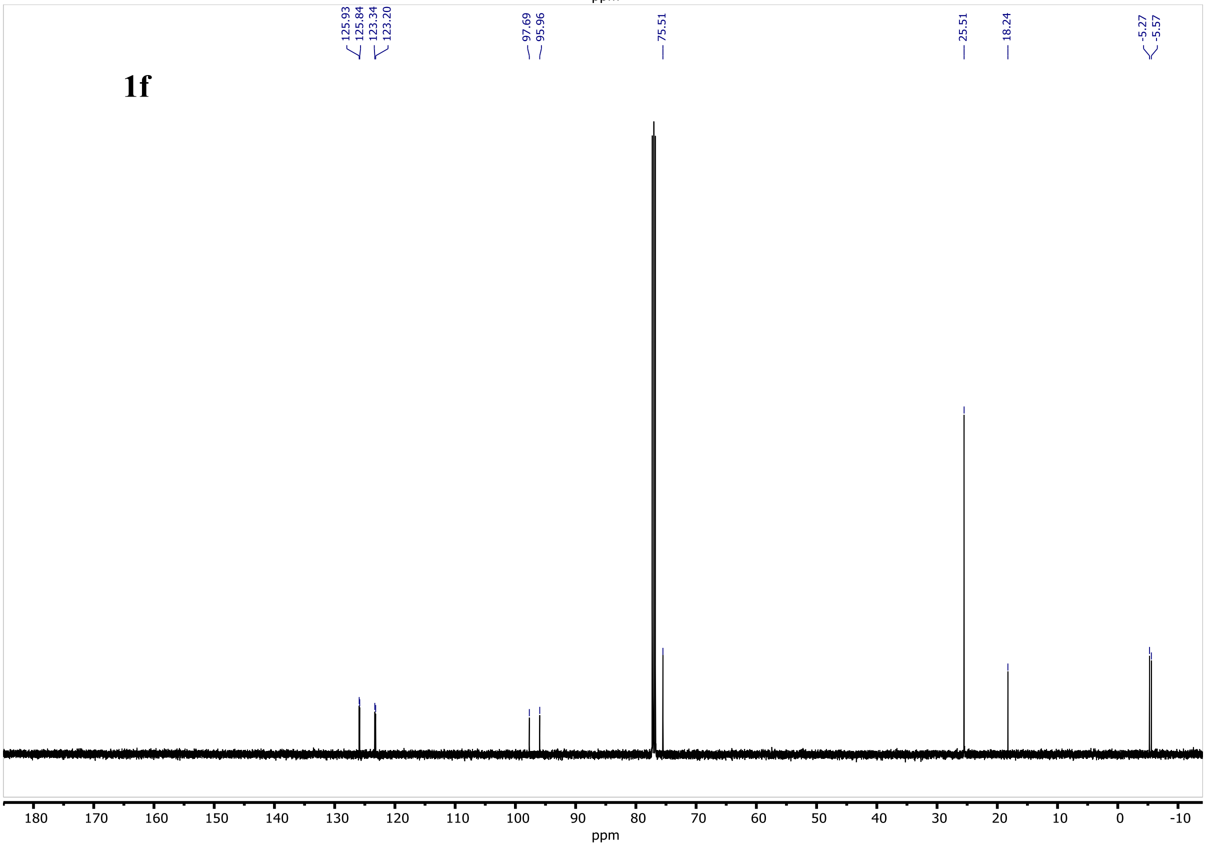
125.93 125.84 123.34 123.20
97.69 95.96
75.51
25.51
18.24
-5.27 -5.57
1f
180 170 160 150 140 130 120 110 100 90 80 70 60 50 40 30 20 10 0 -10
ppm

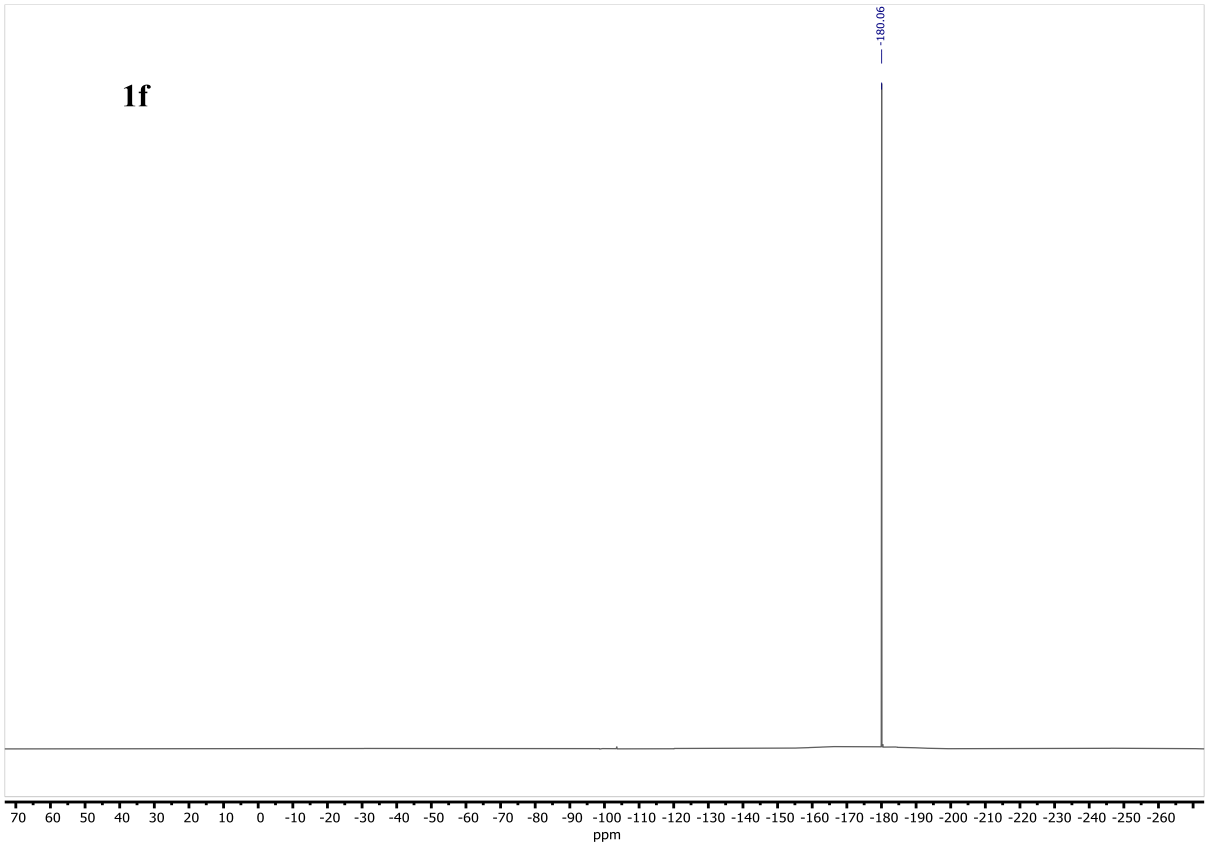

1f
-180.06
70 60 50 40 30 20 10 0 -10 -20 -30 -40 -50 -60 -70 -80 -90 -100 -110 -120 -130 -140 -150 -160 -170 -180 -190 -200 -210 -220 -230 -240 -250 -260
ppm

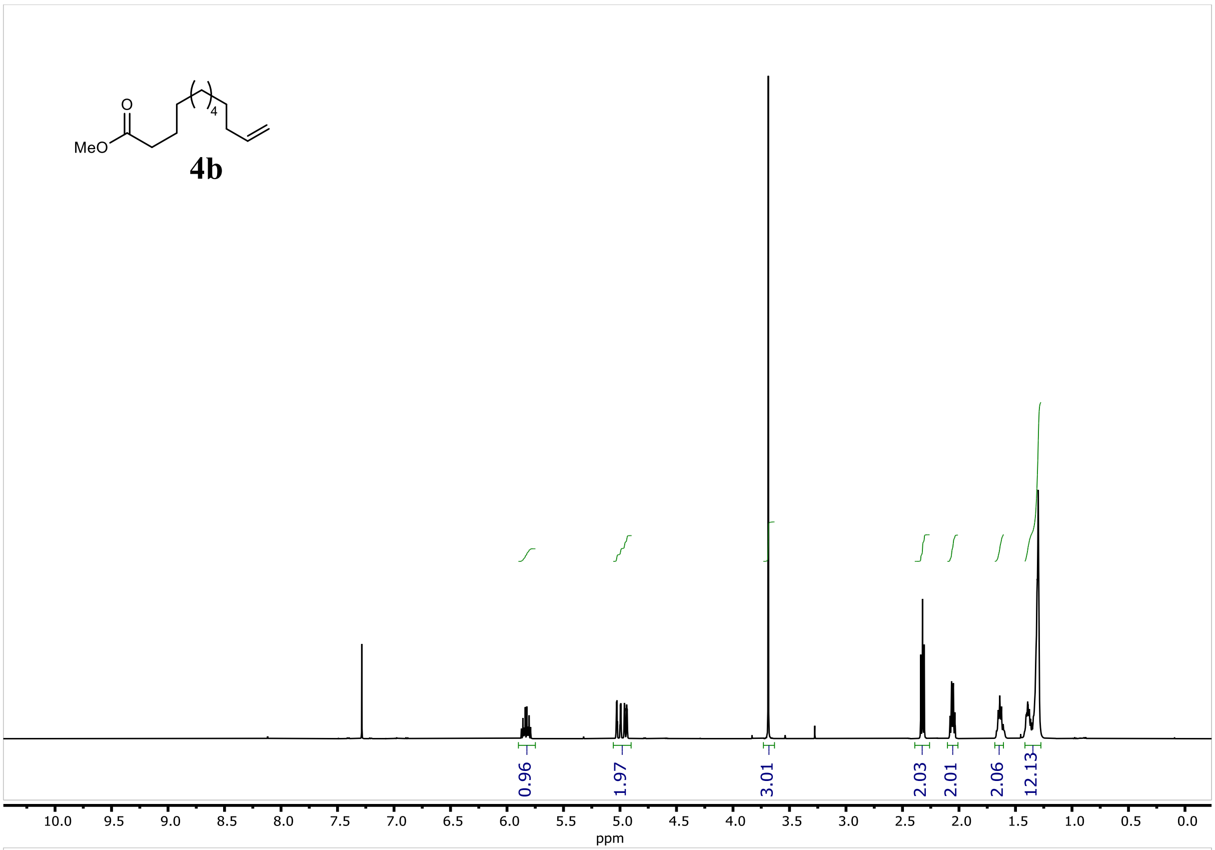
MeO
O
4
4b
0.96
1.97
3.01
2.03
2.01
2.06
12.13
10.0
9.5
9.0
8.5
8.0
7.5
7.0
6.5
6.0
5.5
5.0
4.5
4.0
3.5
3.0
2.5
2.0
1.5
1.0
0.5
0.0
ppm

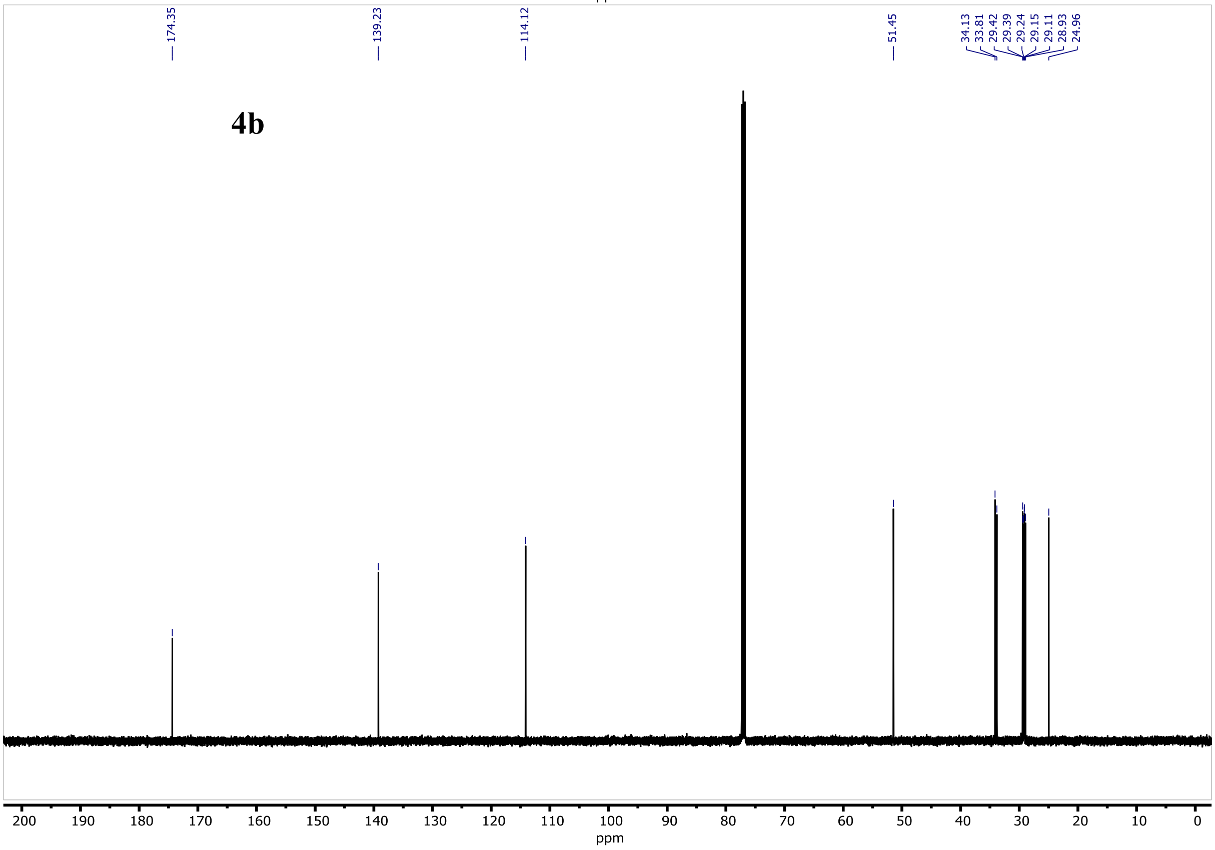
174.35
139.23
114.12
51.45
34.13
33.81
29.42
29.39
29.24
29.15
29.11
28.93
24.96
4b
200
190
180
170
160
150
140
130
120
110
100
90
80
70
60
50
40
30
20
10
0
ppm

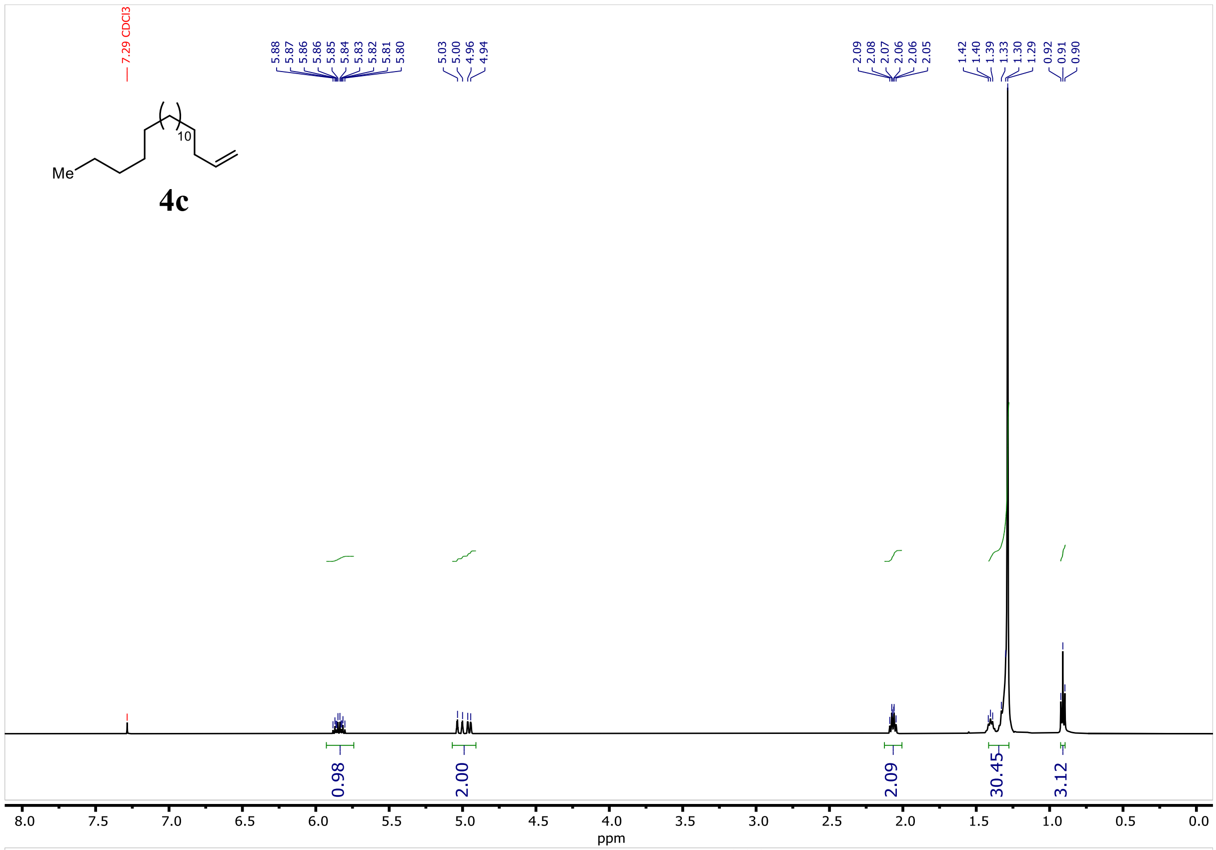
7.29 CDCl3
5.88
5.87
5.86
5.86
5.85
5.84
5.83
5.82
5.81
5.80
5.03
5.00
4.96
4.94
2.09
2.08
2.07
2.06
2.06
2.05
1.42
1.40
1.39
1.33
1.30
1.29
0.92
0.91
0.90
Me
10
4c
0.98
2.00
2.09
30.45
3.12
8.0
7.5
7.0
6.5
6.0
5.5
5.0
4.5
4.0
3.5
3.0
2.5
2.0
1.5
1.0
0.5
0.0
ppm

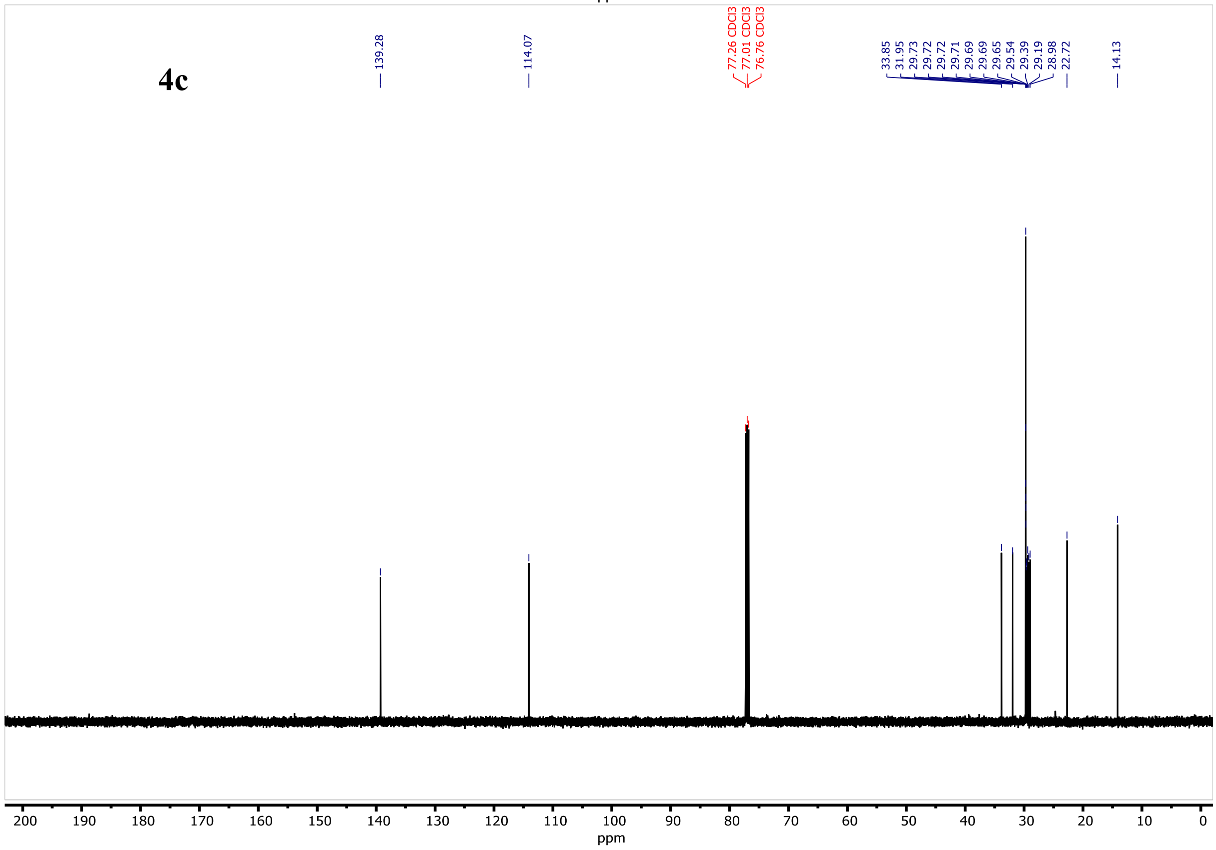
4c
139.28
114.07
77.26 CDCl3
77.01 CDCl3
76.76 CDCl3
33.85
31.95
29.73
29.72
29.72
29.71
29.69
29.69
29.65
29.54
29.39
29.19
28.98
22.72
14.13
200
190
180
170
160
150
140
130
120
110
100
90
80
70
60
50
40
30
20
10
0
ppm

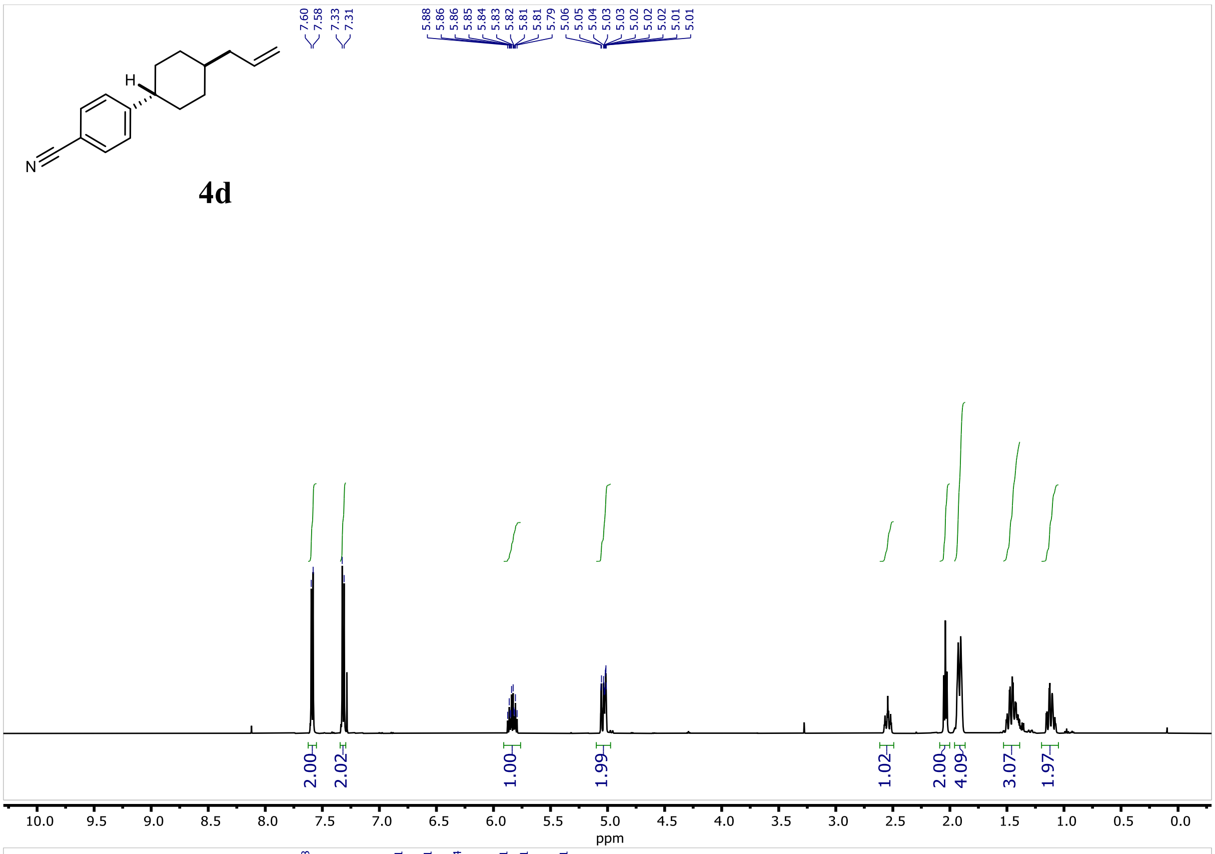
4d
7.60
7.58
7.33
7.31
5.88
5.86
5.86
5.85
5.84
5.83
5.82
5.81
5.81
5.79
5.06
5.05
5.04
5.03
5.03
5.02
5.02
5.02
5.01
5.01
2.00
2.02
1.00
1.99
1.02
2.00
4.09
3.07
1.97
ppm

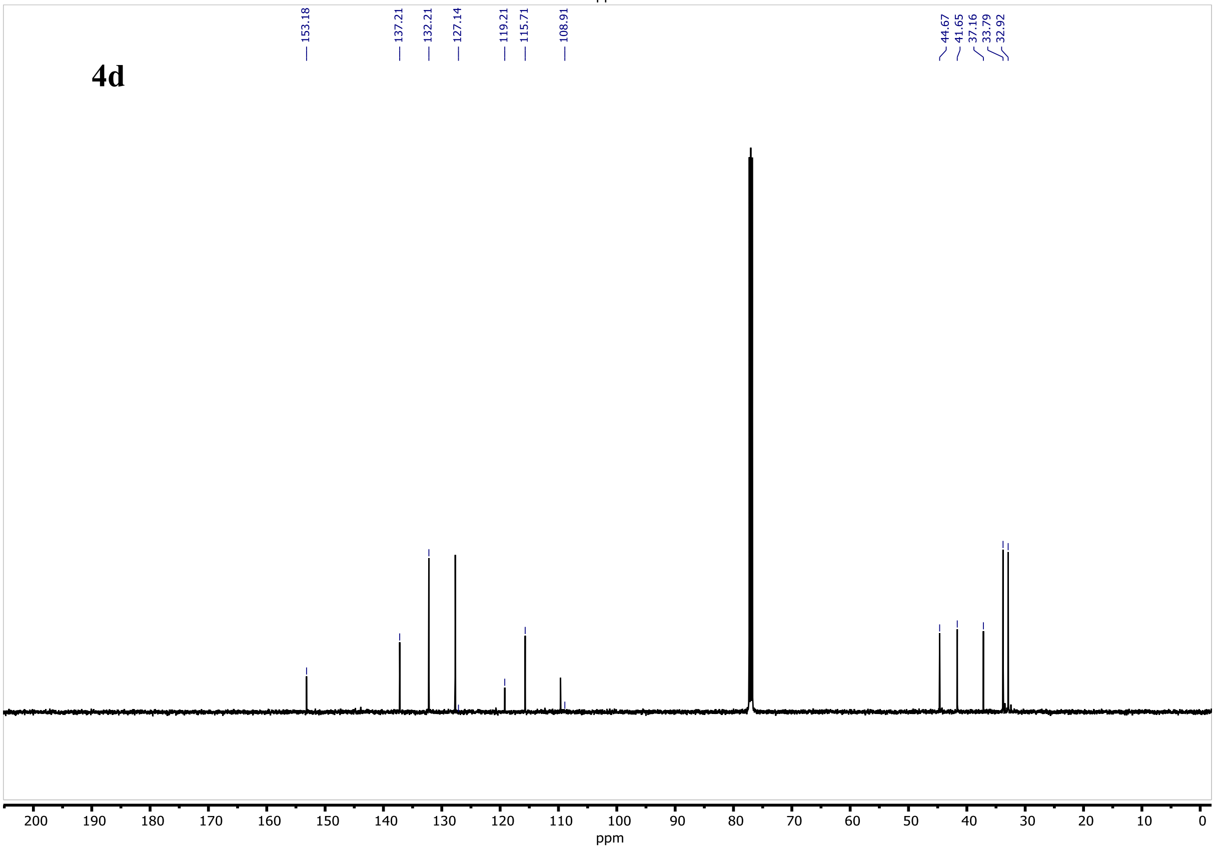
4d
153.18
137.21
132.21
127.14
119.21
115.71
108.91
44.67
41.65
37.16
33.79
32.92
ppm

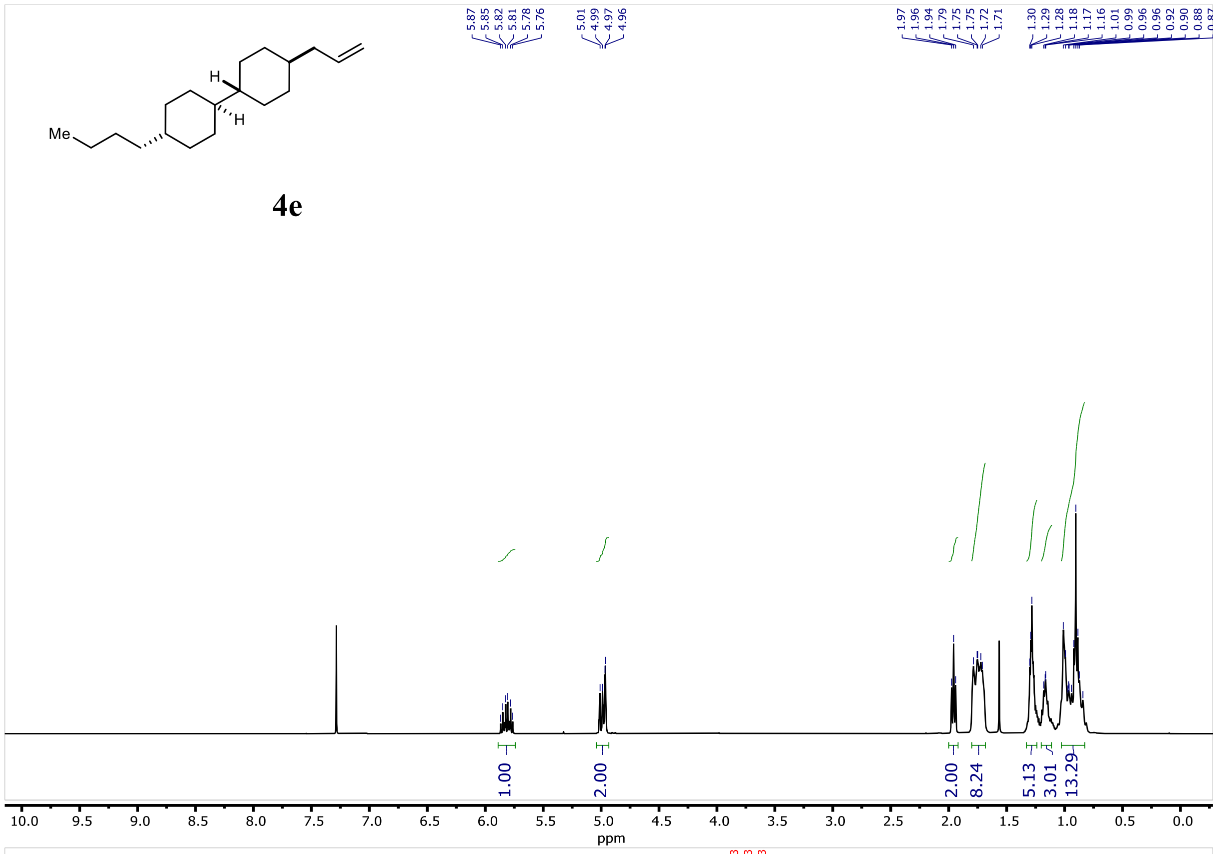
Me
H
H
5.87
5.85
5.82
5.81
5.78
5.76
5.01
4.99
4.97
4.96
1.97
1.96
1.94
1.79
1.75
1.75
1.72
1.71
1.30
1.29
1.28
1.18
1.17
1.16
1.01
0.99
0.96
0.96
0.92
0.90
0.88
1.00
2.00
2.00
8.24
5.13
3.01
13.29
ppm

4e

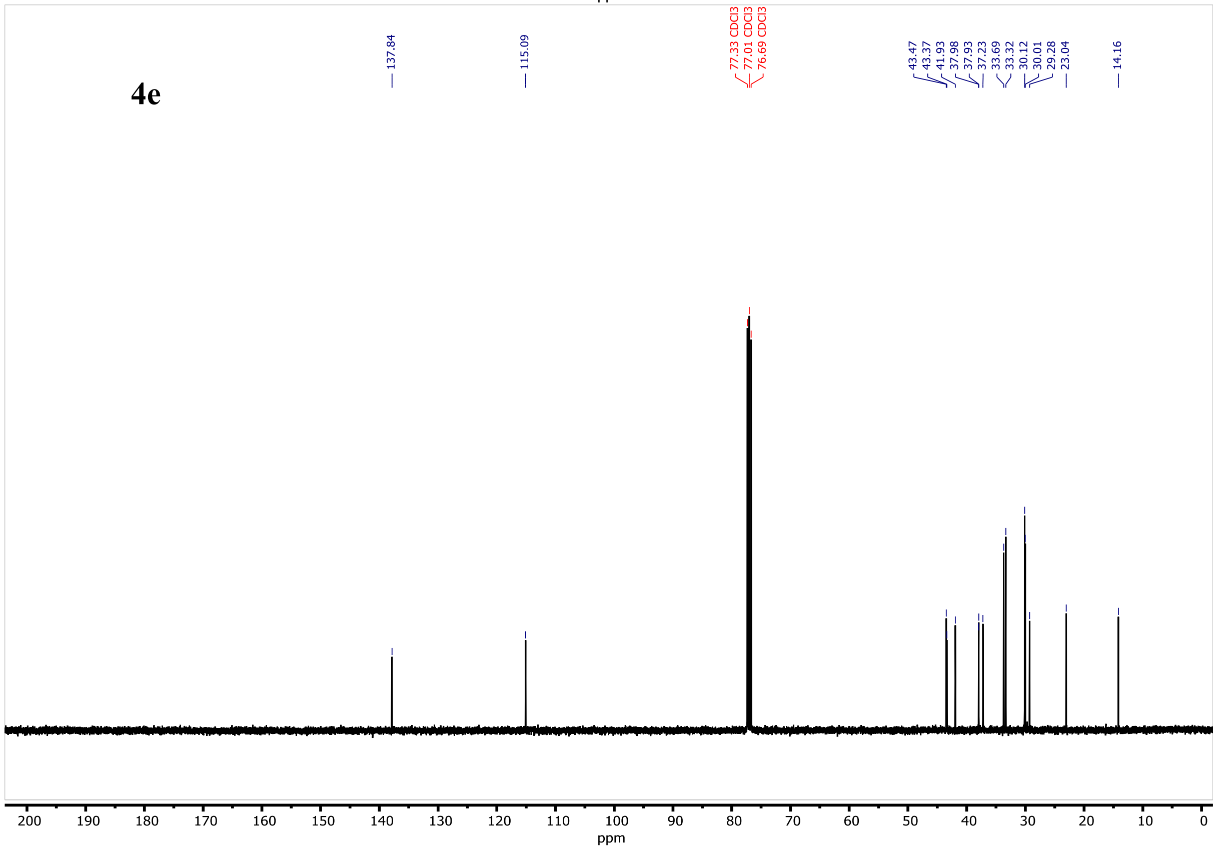
137.84
115.09
77.33 CDCl3
77.01 CDCl3
76.69 CDCl3
43.47
43.37
41.93
37.98
37.93
37.23
33.69
33.32
30.12
30.01
29.28
23.04
14.16
ppm

4e

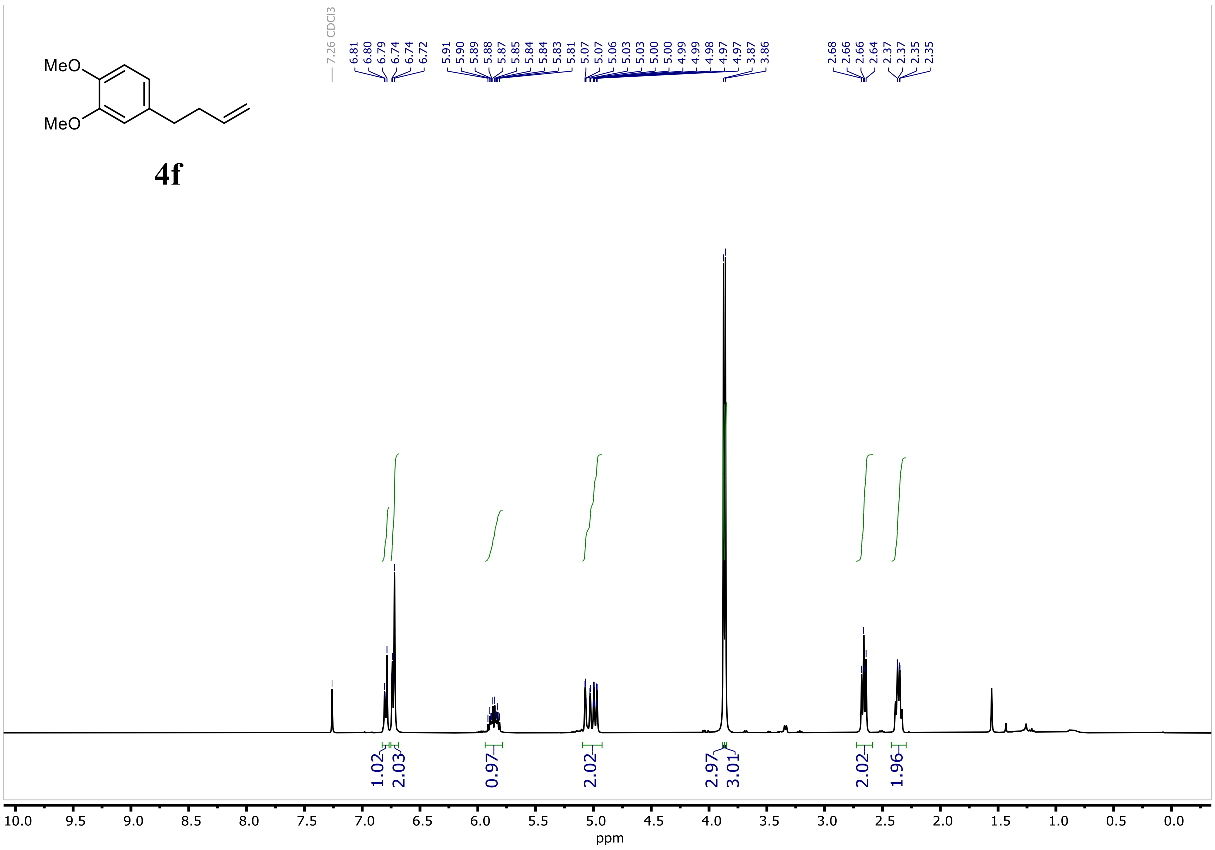

MeO
MeO
4f
7.26 CDCl3
6.81 6.80 6.79 6.74 6.74 6.72
5.91 5.90 5.89 5.88 5.87 5.85 5.84 5.84 5.83 5.81
5.07 5.07 5.06 5.03 5.03 5.00 5.00 4.99 4.99 4.98 4.97 4.97
3.87 3.86
2.68 2.66 2.66 2.64 2.37 2.37 2.35 2.35
1.02 2.03 0.97 2.02 2.97 3.01 2.02 1.96
ppm

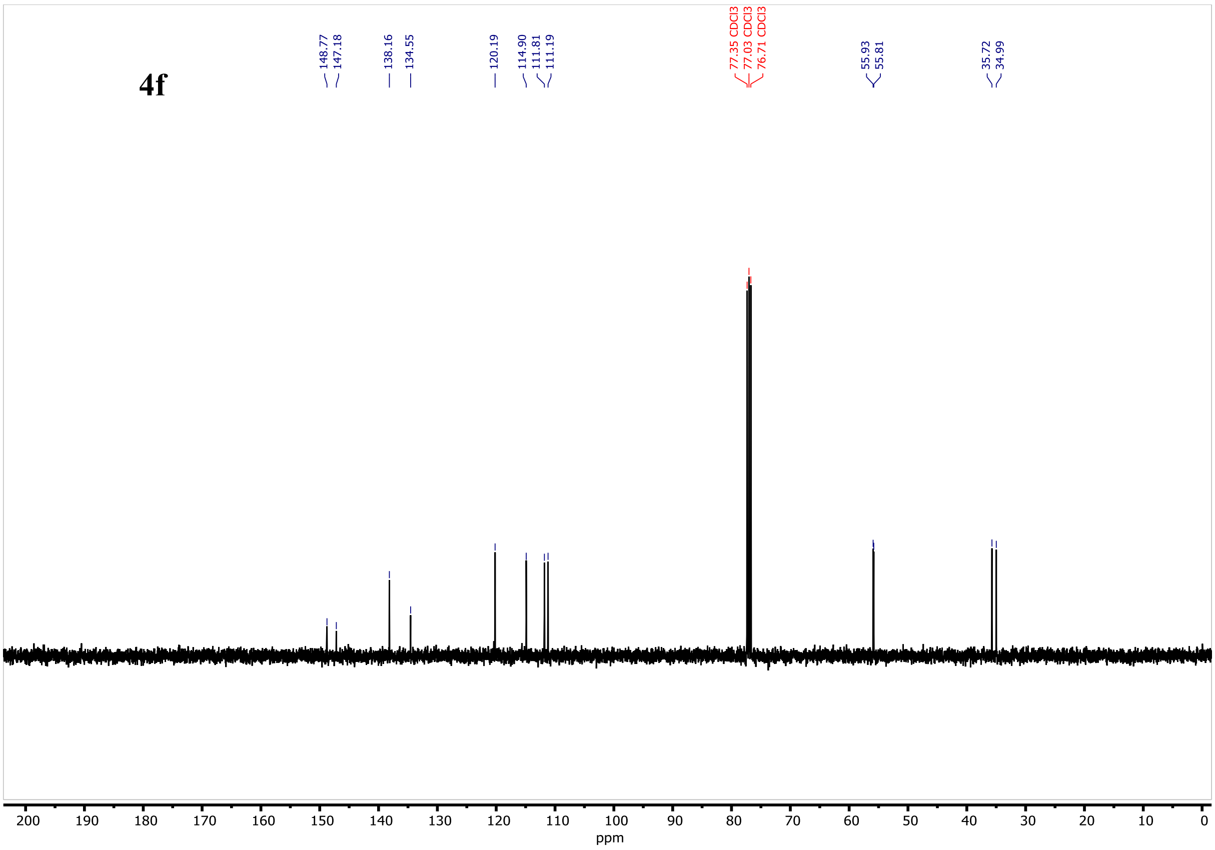

4f
148.77 147.18 138.16 134.55 120.19 114.90 111.81 111.19
77.35 CDCl3 77.03 CDCl3 76.71 CDCl3
55.93 55.81 35.72 34.99
ppm

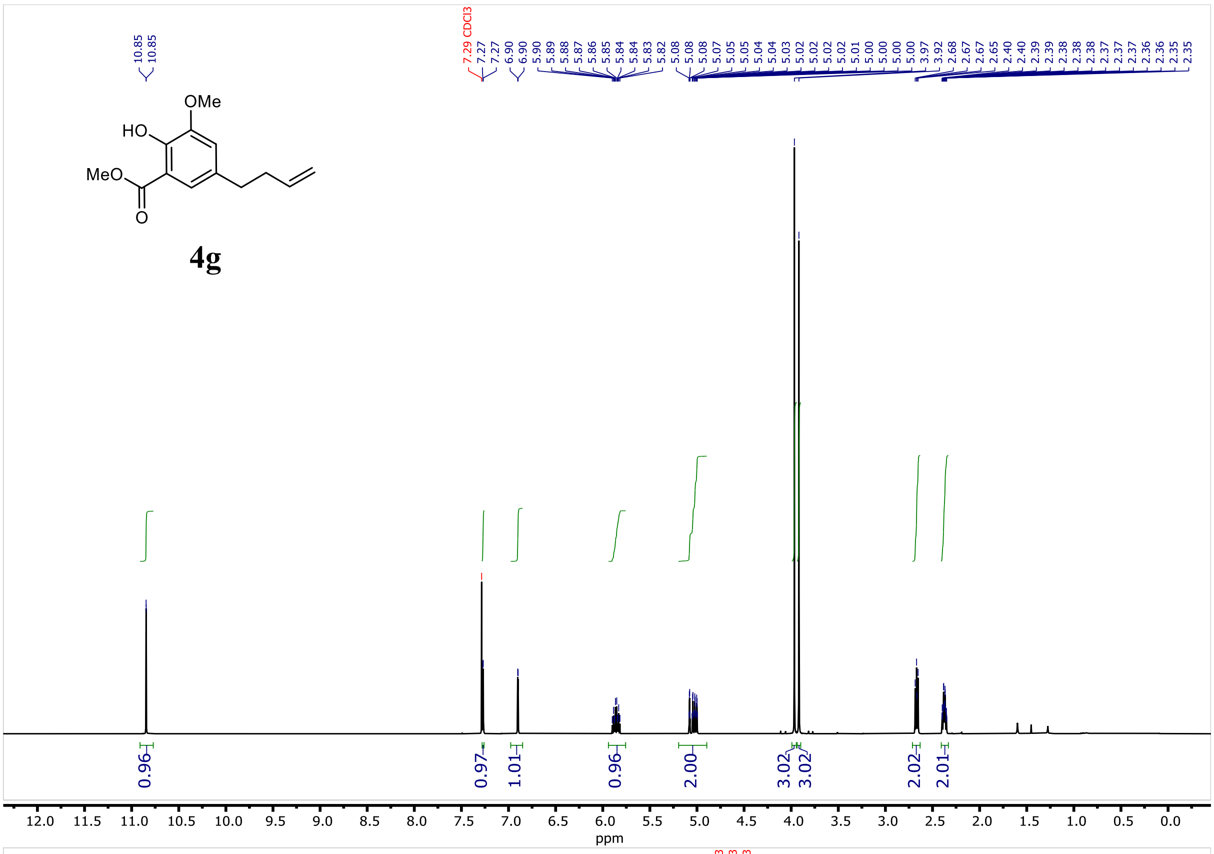
OMe
HO
MeO
O
4g
10.85
10.85
7.29 CDCl3
7.27
7.27
6.90
6.90
0.96
0.97
1.01
0.96
2.00
3.02
3.02
2.02
2.01
ppm

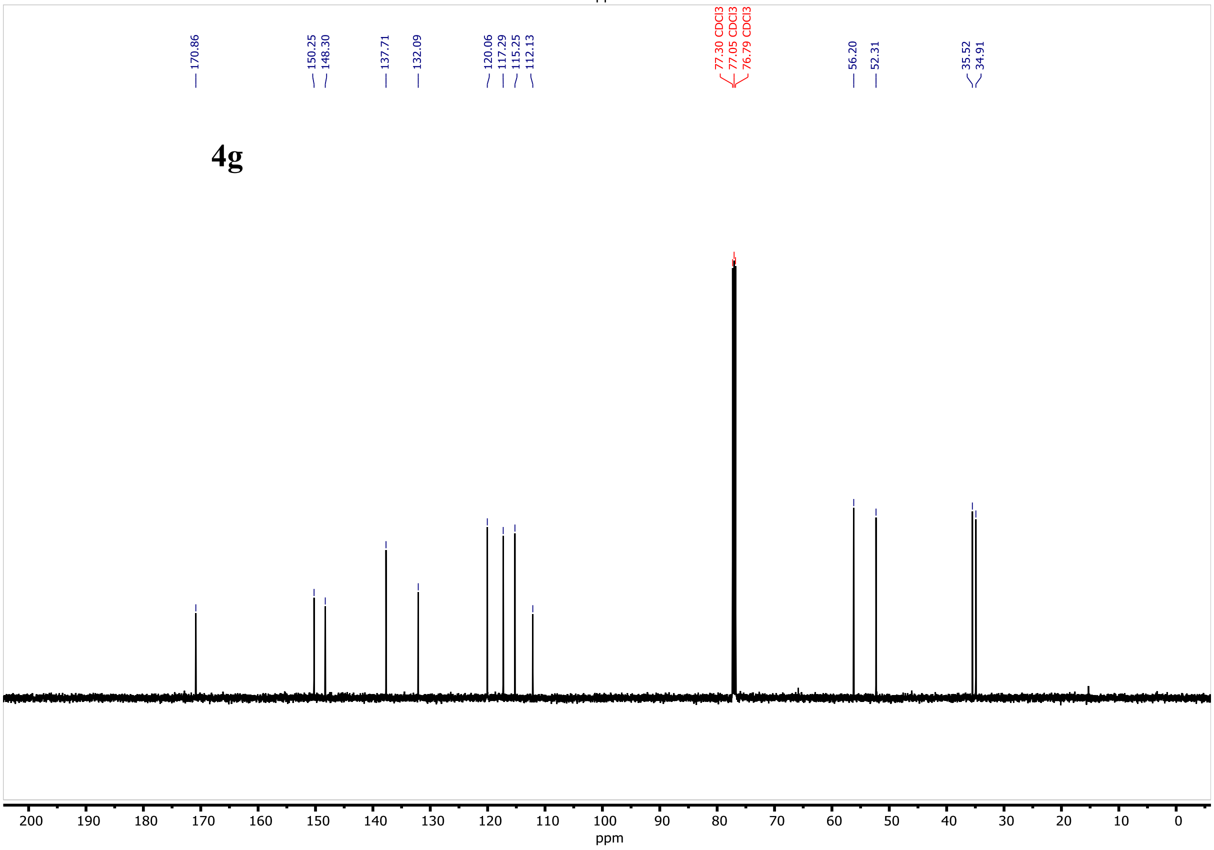
4g
170.86
150.25
148.30
137.71
132.09
120.06
117.29
115.25
112.13
77.30 CDCl3
77.05 CDCl3
76.79 CDCl3
56.20
52.31
35.52
34.91
ppm

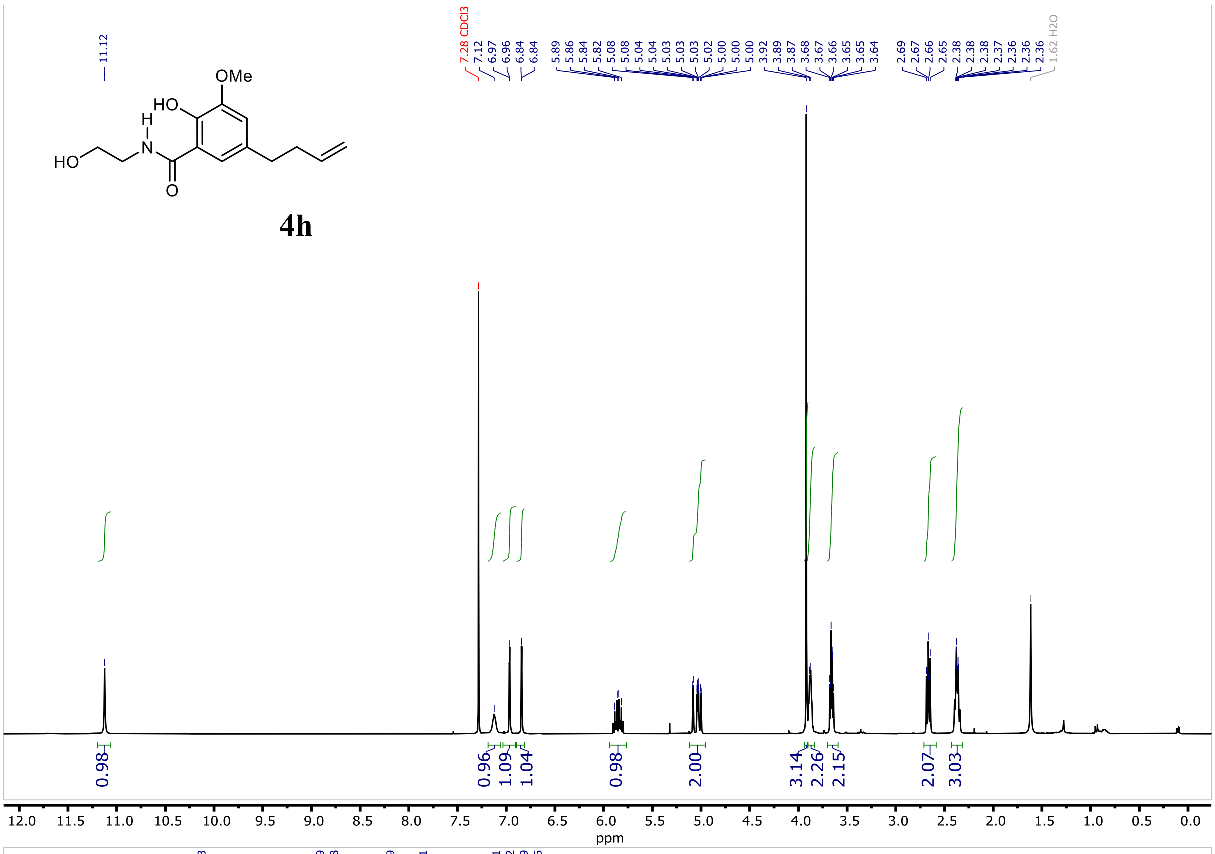

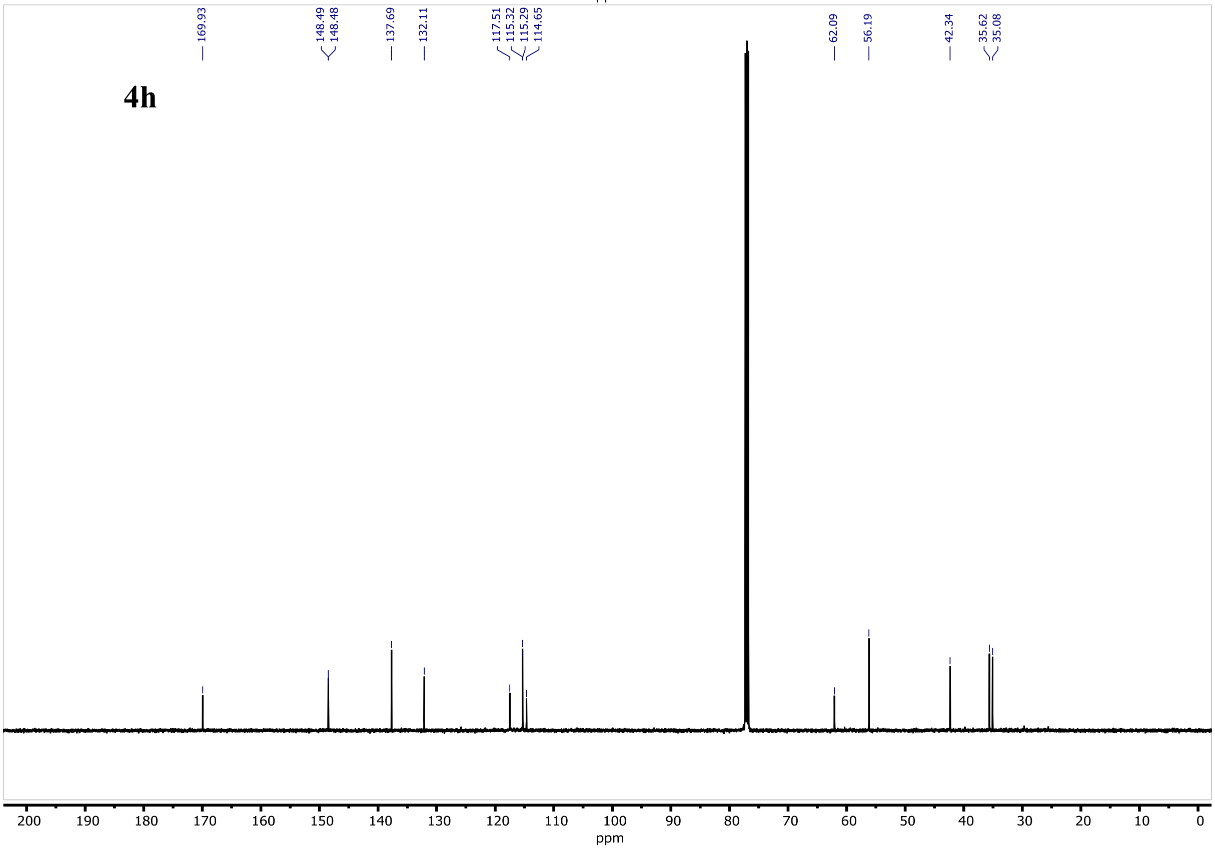

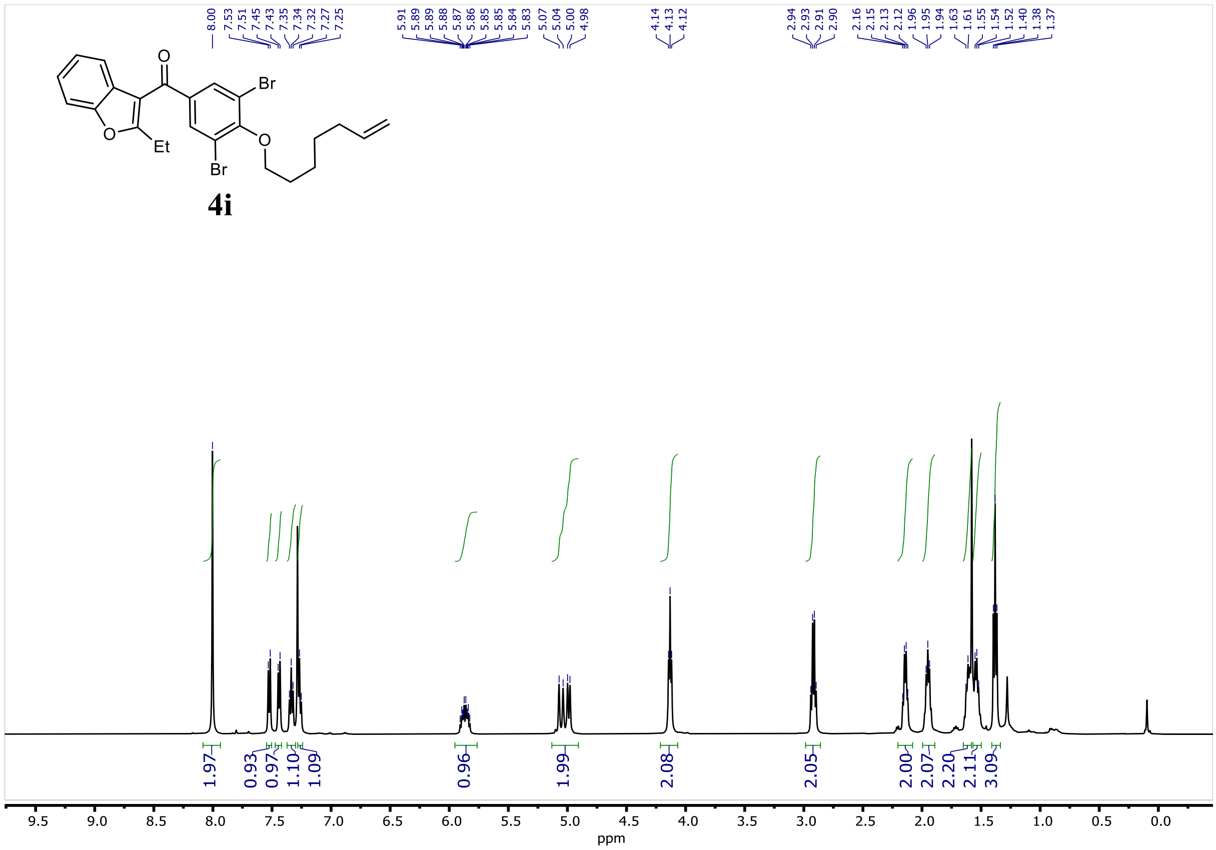
4i
8.00
7.53
7.51
7.45
7.43
7.35
7.34
7.32
7.27
7.25
5.91
5.89
5.89
5.88
5.87
5.86
5.85
5.85
5.84
5.83
5.07
5.04
5.00
4.98
4.14
4.13
4.12
2.94
2.93
2.91
2.90
2.16
2.15
2.13
2.12
1.96
1.95
1.94
1.63
1.61
1.55
1.54
1.52
1.40
1.38
1.37
1.97
0.93
0.97
1.10
1.09
0.96
1.99
2.08
2.05
2.00
2.07
2.20
2.11
3.09
ppm

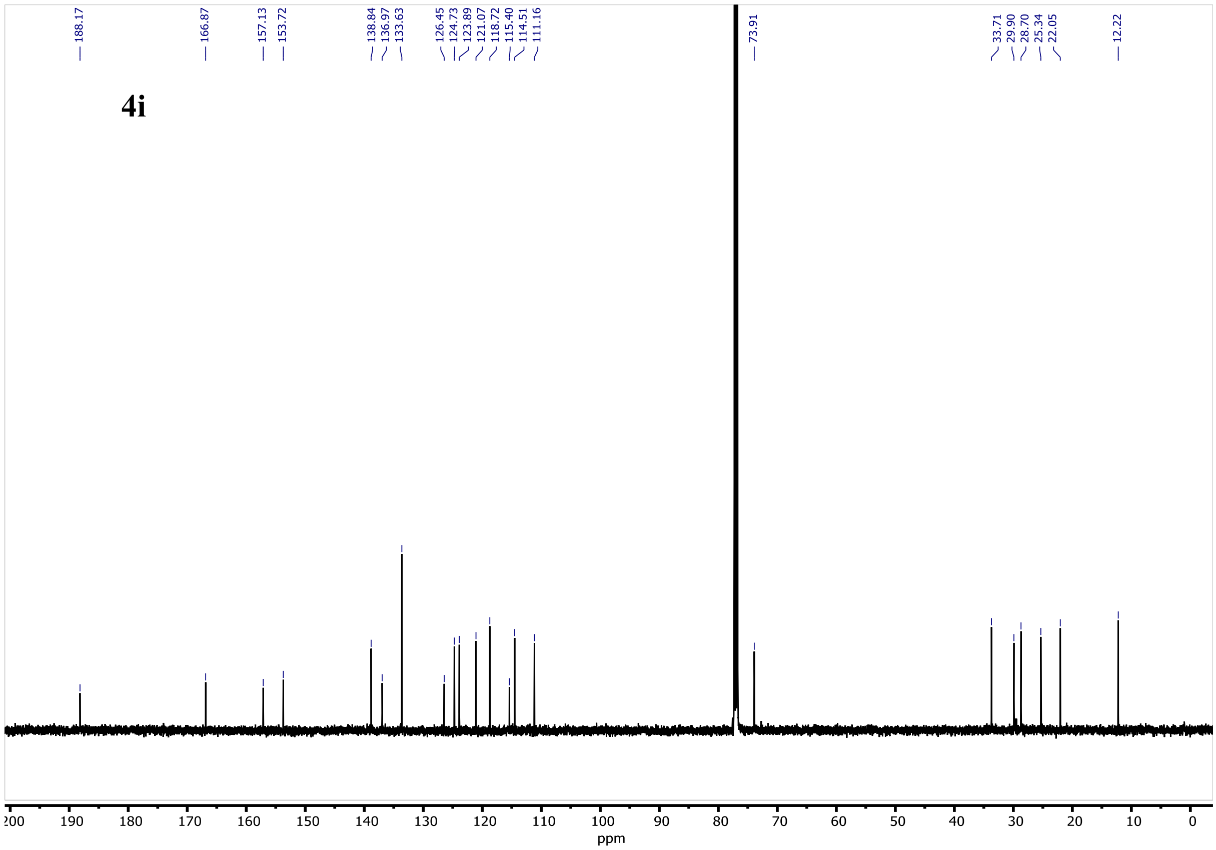
4i
188.17
166.87
157.13
153.72
138.84
136.97
133.63
126.45
124.73
123.89
121.07
118.72
115.40
114.51
111.16
73.91
33.71
29.90
28.70
25.34
22.05
12.22
ppm

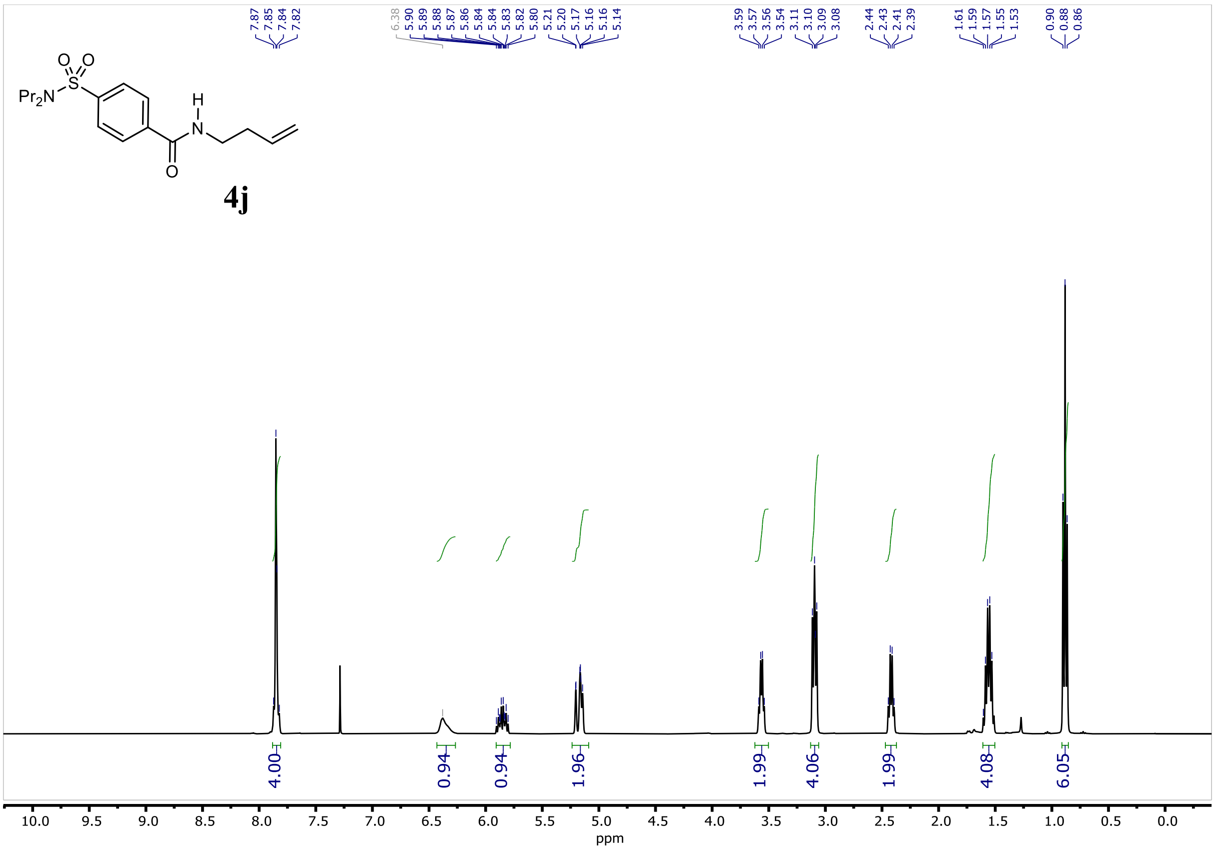
4j
4.00
0.94
0.94
1.96
1.99
4.06
1.99
4.08
6.05
ppm

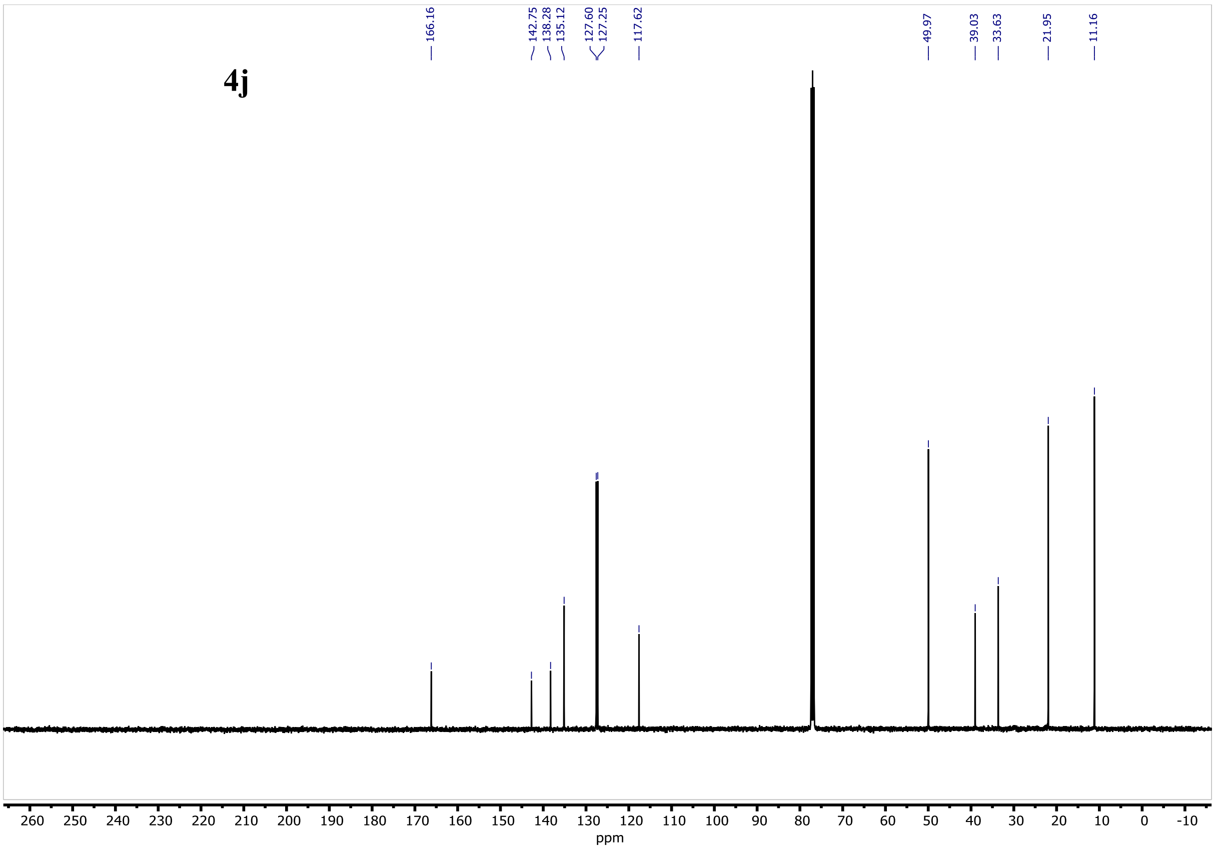
4j
166.16
142.75
138.28
135.12
127.60
127.25
117.62
49.97
39.03
33.63
21.95
11.16
ppm

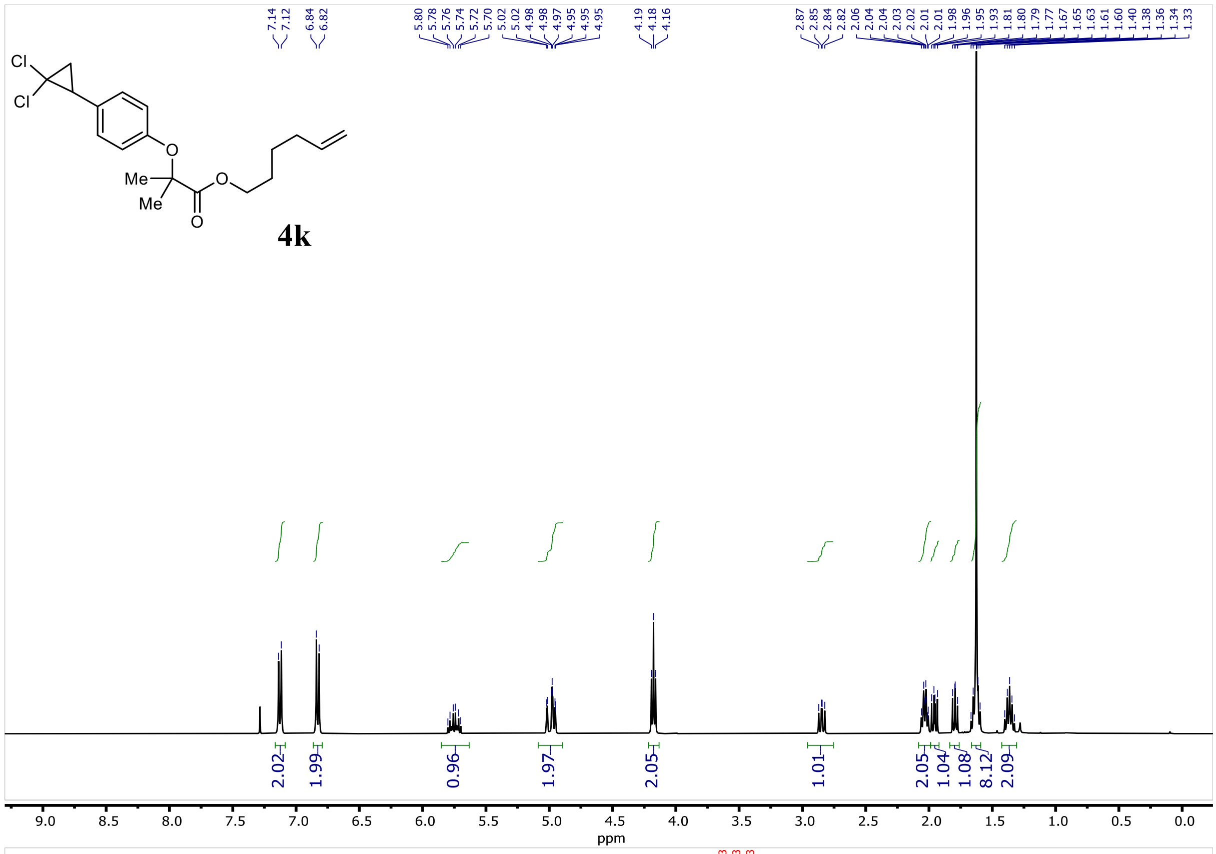

4k

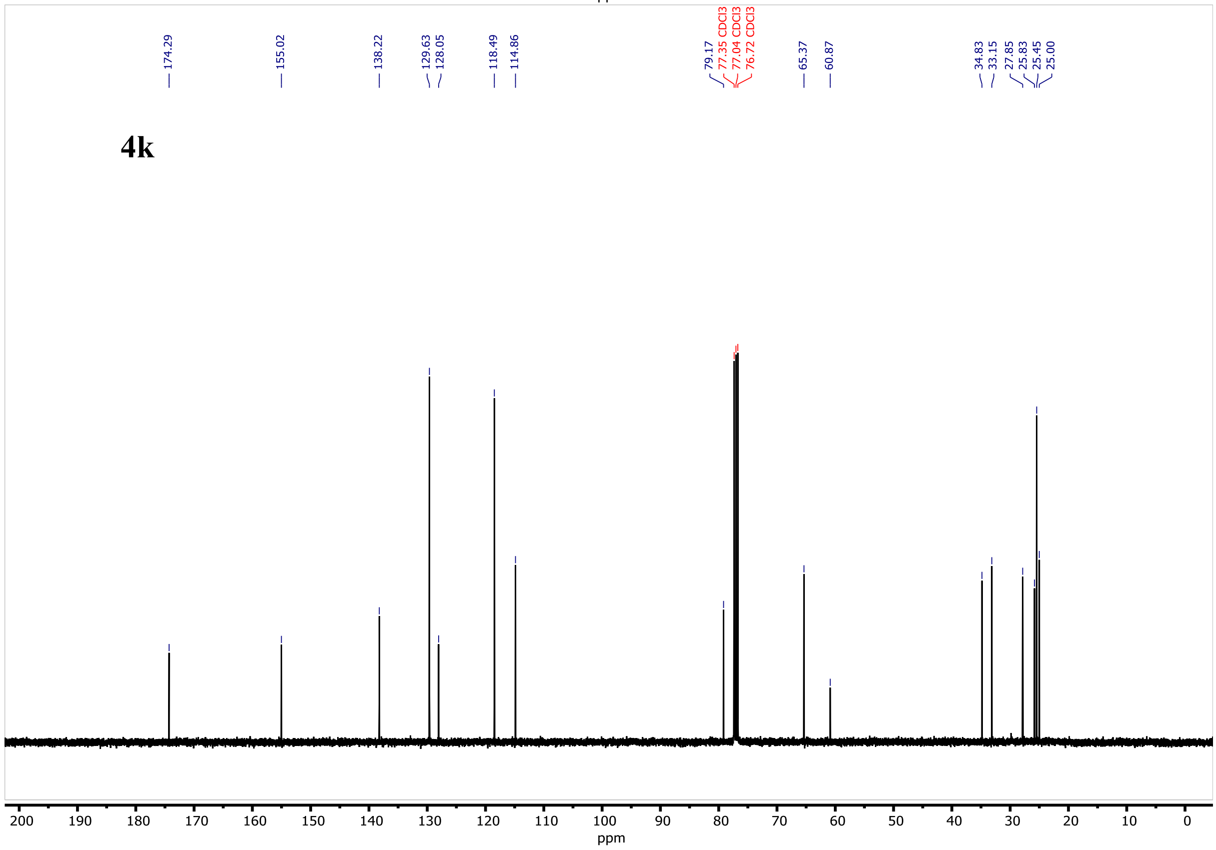

4k

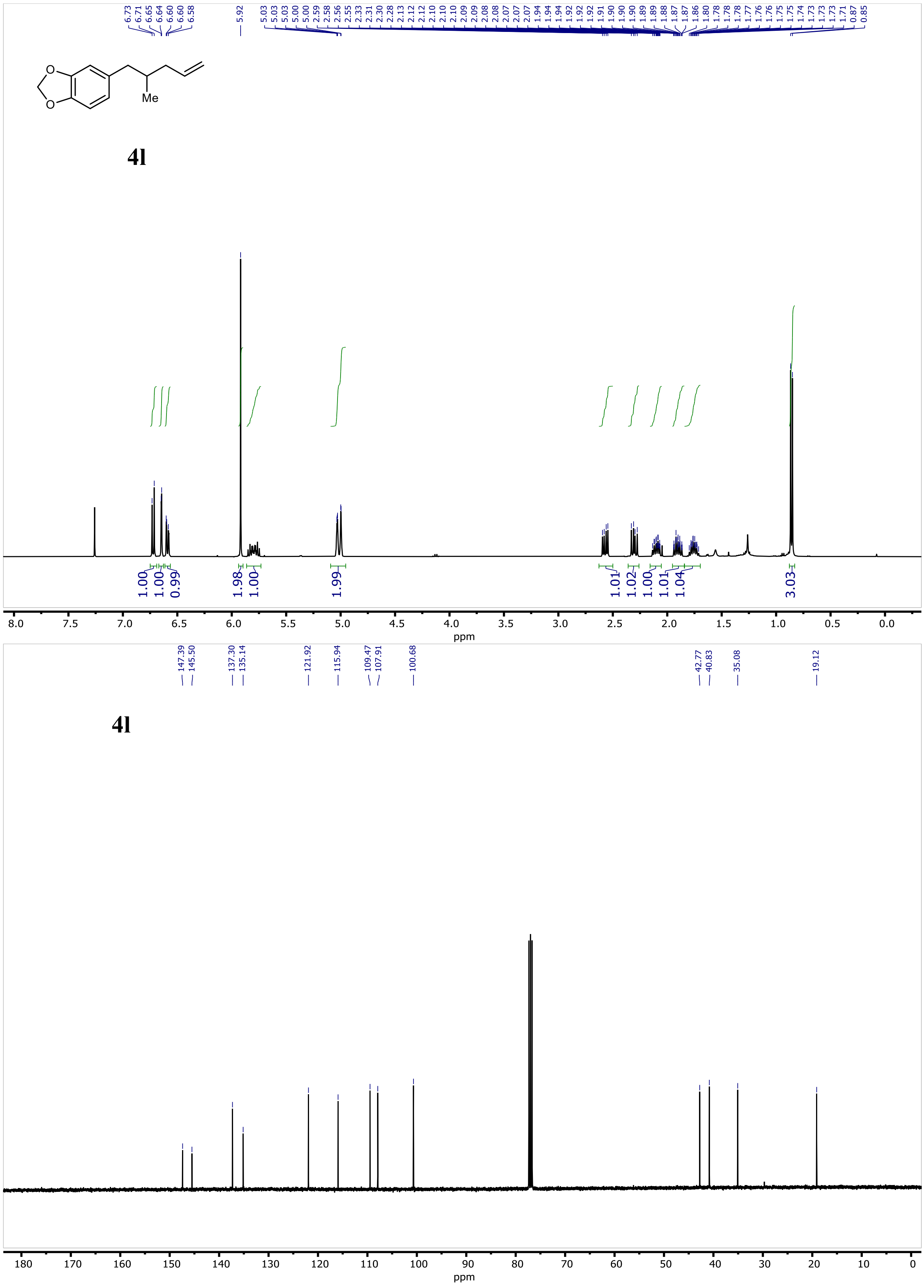
4l
4l
ppm
ppm

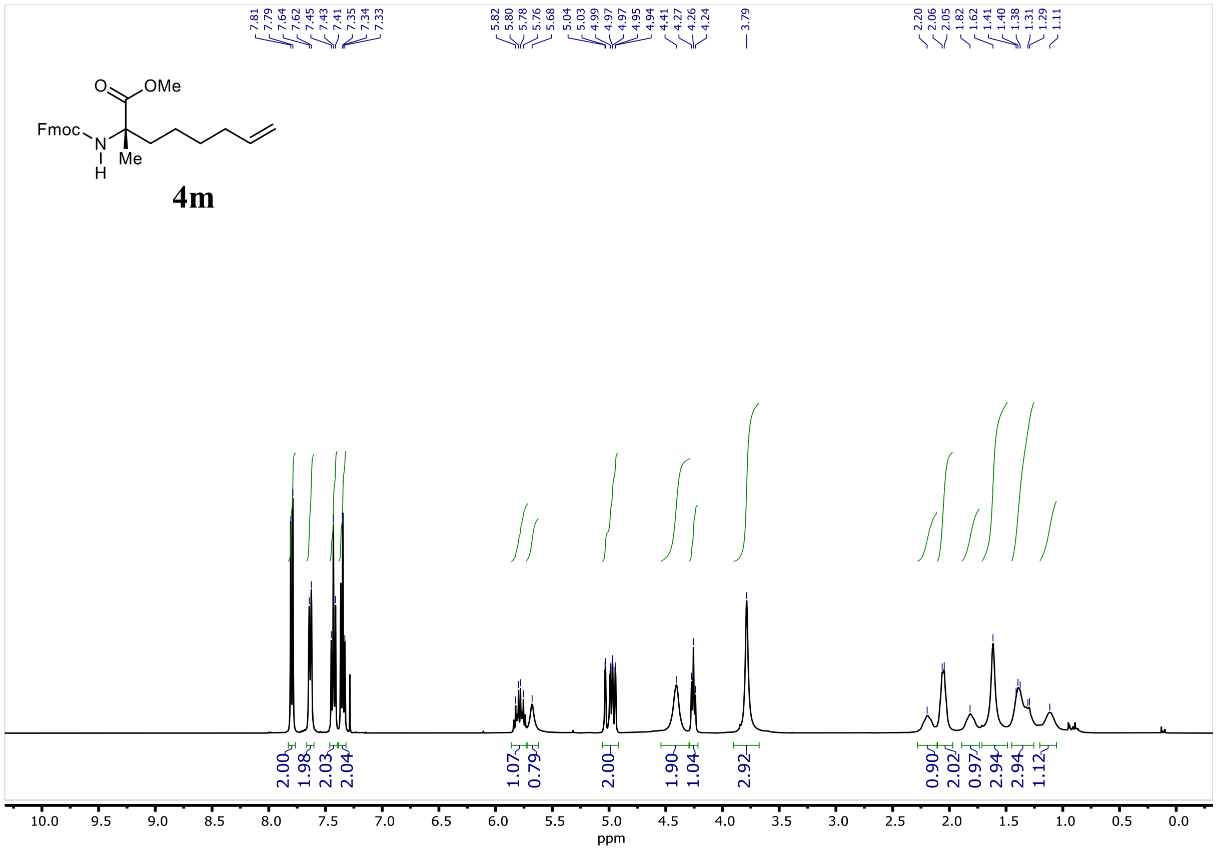
7.81 7.79 7.64 7.62 7.45 7.43 7.41 7.35 7.34 7.33
5.82 5.80 5.78 5.76 5.68 5.04 5.03 4.99 4.97 4.97 4.95 4.94 4.41 4.27 4.26 4.24
3.79
2.20 2.06 2.05 1.82 1.62 1.41 1.40 1.38 1.31 1.29 1.11
O
OMe
Fmoc
N
H
Me
4m
2.00 1.98 2.03 2.04 1.07 0.79 2.00 1.90 1.04 2.92 0.90 2.02 0.97 2.94 2.94 1.12
10.0 9.5 9.0 8.5 8.0 7.5 7.0 6.5 6.0 5.5 5.0 4.5 4.0 3.5 3.0 2.5 2.0 1.5 1.0 0.5 0.0
ppm

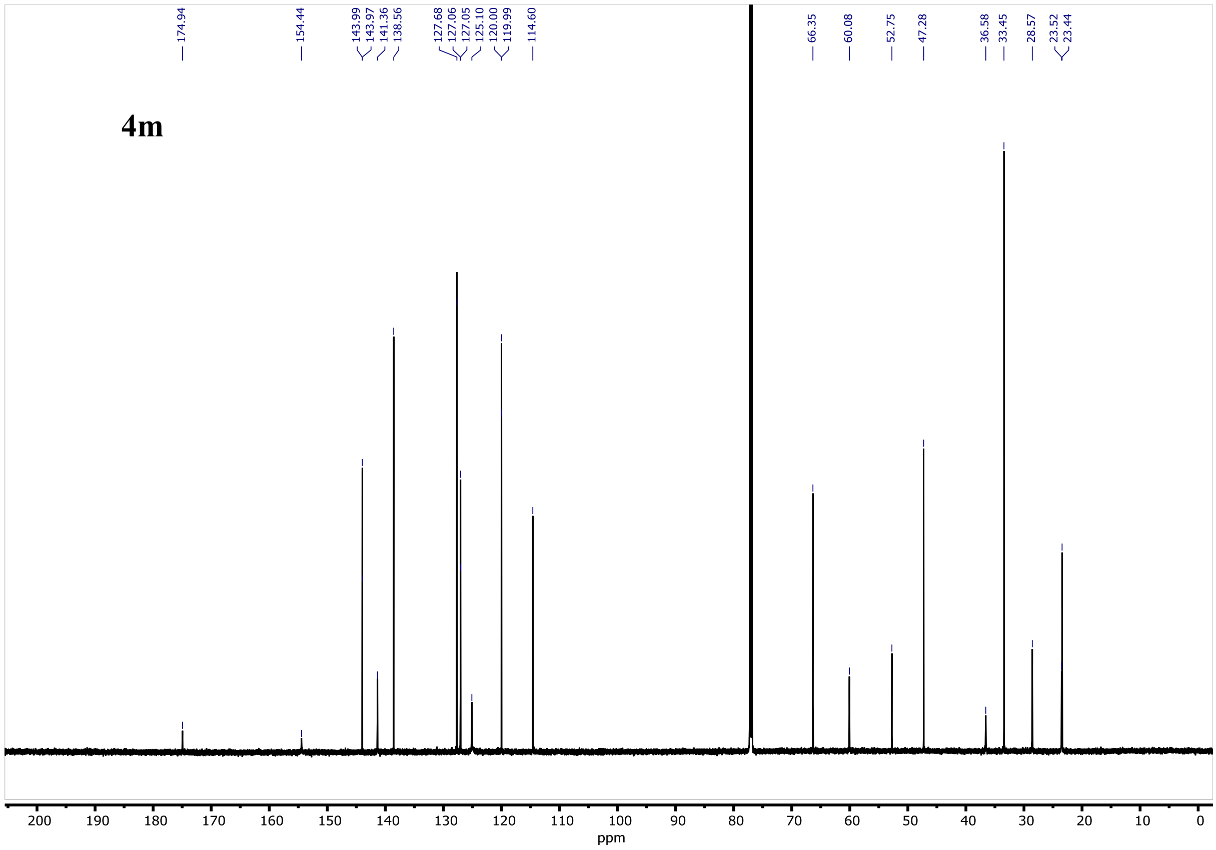
174.94
154.44
143.99 143.97 141.36 138.56
127.68 127.06 127.05 125.10 120.00 119.99
114.60
66.35
60.08
52.75
47.28
36.58
33.45
28.57
23.52 23.44
4m
200 190 180 170 160 150 140 130 120 110 100 90 80 70 60 50 40 30 20 10 0
ppm

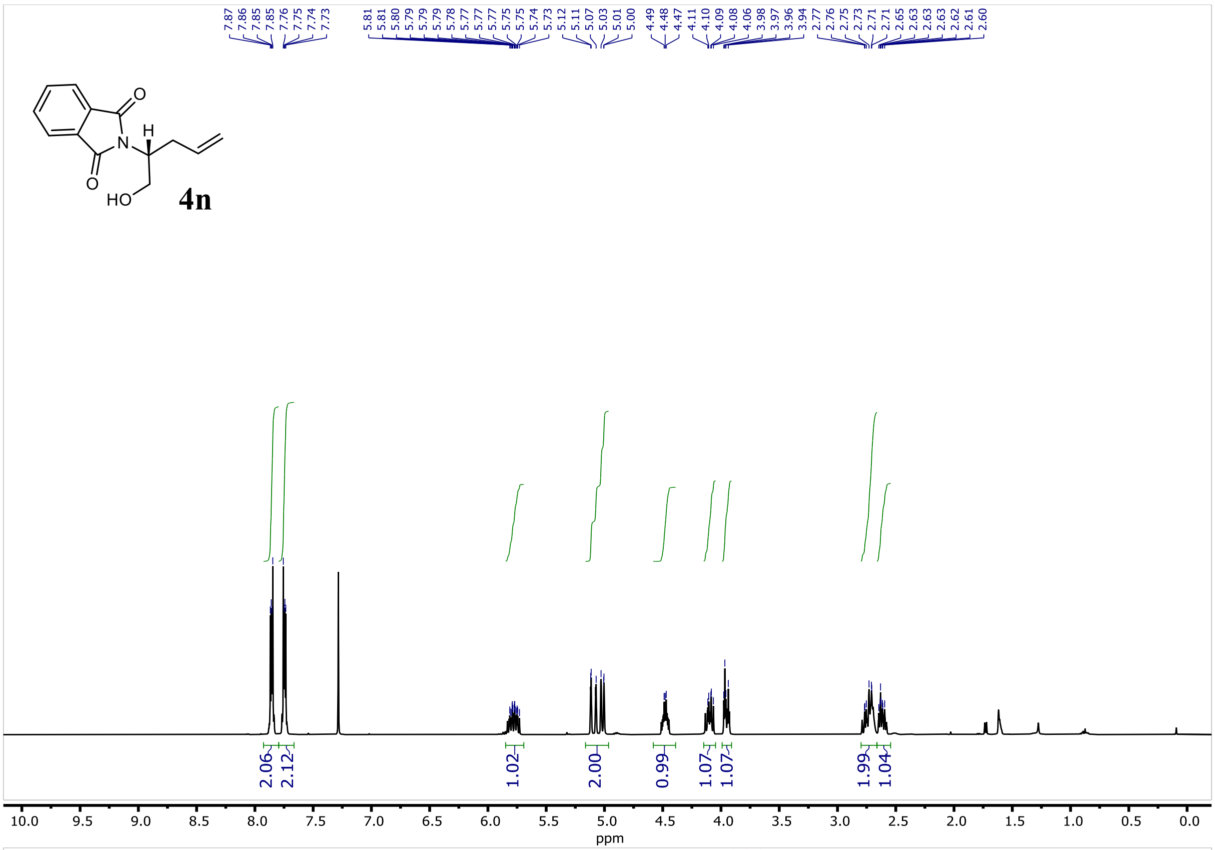
4n
7.87
7.86
7.85
7.85
7.76
7.75
7.74
7.73
5.81
5.81
5.80
5.79
5.79
5.79
5.78
5.77
5.77
5.77
5.75
5.75
5.74
5.73
5.12
5.11
5.07
5.03
5.01
5.00
4.49
4.48
4.47
4.11
4.10
4.09
4.08
4.06
3.98
3.97
3.96
3.94
2.77
2.76
2.75
2.73
2.71
2.71
2.65
2.63
2.63
2.63
2.62
2.61
2.60
2.06
2.12
1.02
2.00
0.99
1.07
1.07
1.99
1.04
ppm

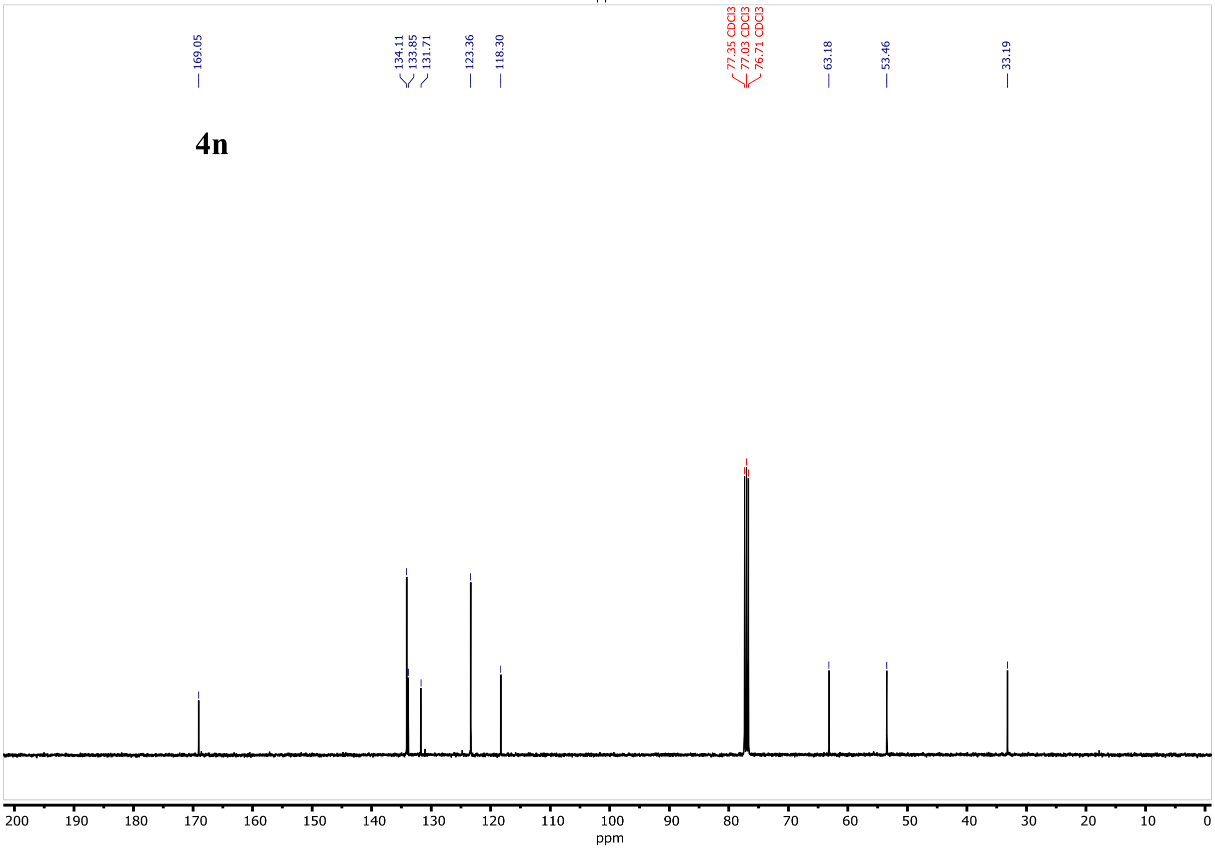
4n
169.05
134.11
133.85
131.71
123.36
118.30
77.35 CDCl3
77.03 CDCl3
76.71 CDCl3
63.18
53.46
33.19
ppm

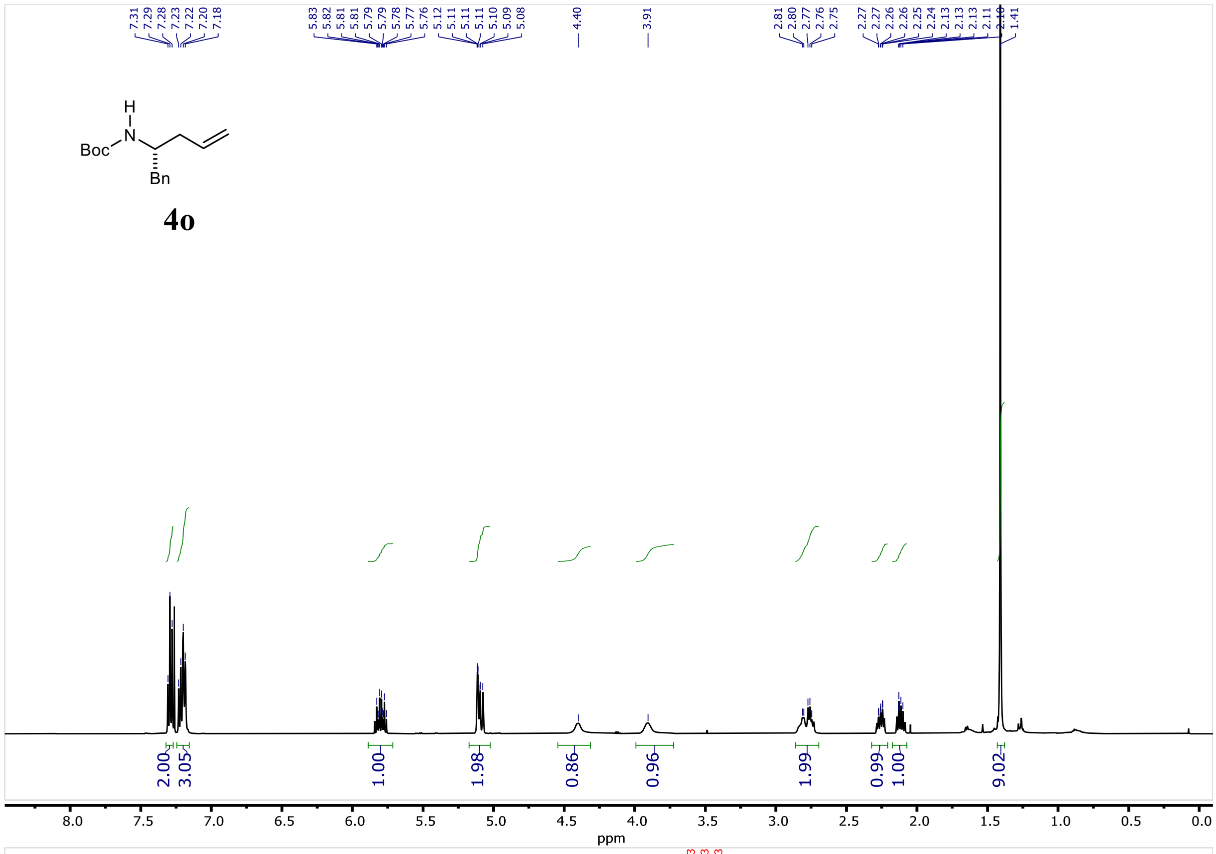

7.31
7.29
7.28
7.23
7.22
7.20
7.18
5.83
5.82
5.81
5.81
5.79
5.79
5.78
5.77
5.76
5.12
5.11
5.11
5.11
5.10
5.09
5.08
4.40
3.91
2.81
2.80
2.77
2.76
2.75
2.27
2.27
2.26
2.26
2.25
2.24
2.13
2.13
2.13
2.11
1.41
H
N
Boc
Bn
4o
2.00
3.05
1.00
1.98
0.86
0.96
1.99
0.99
1.00
9.02
ppm

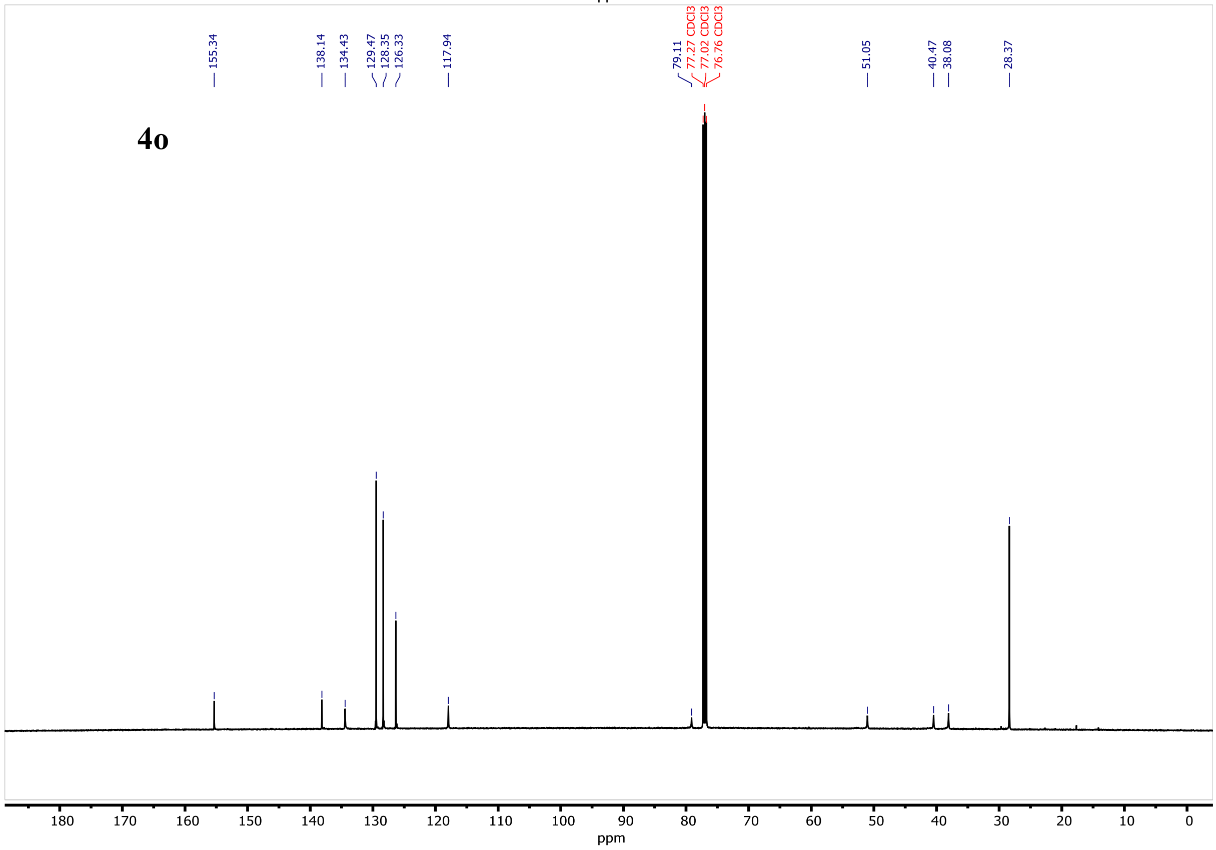

155.34
138.14
134.43
129.47
128.35
126.33
117.94
79.11
77.27 CDCl3
77.02 CDCl3
76.76 CDCl3
51.05
40.47
38.08
28.37
4o
ppm

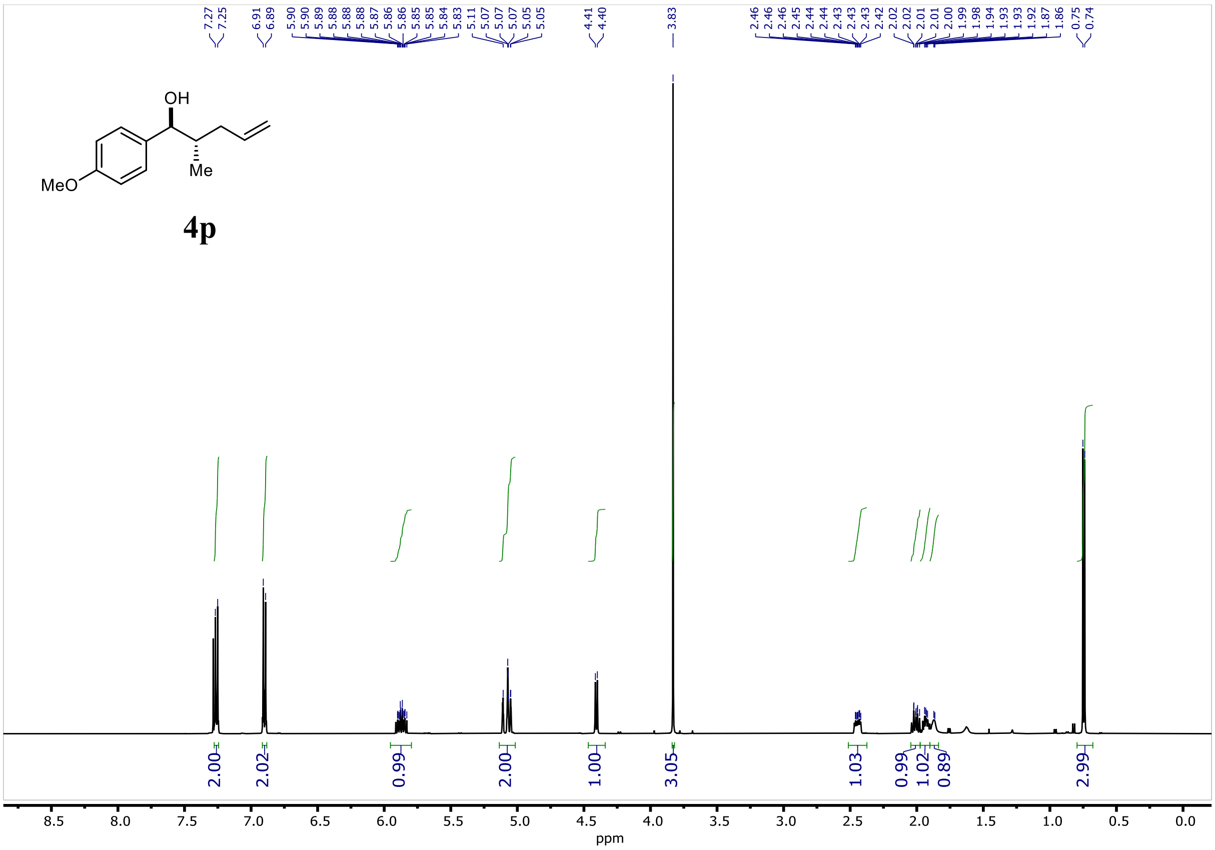

4p

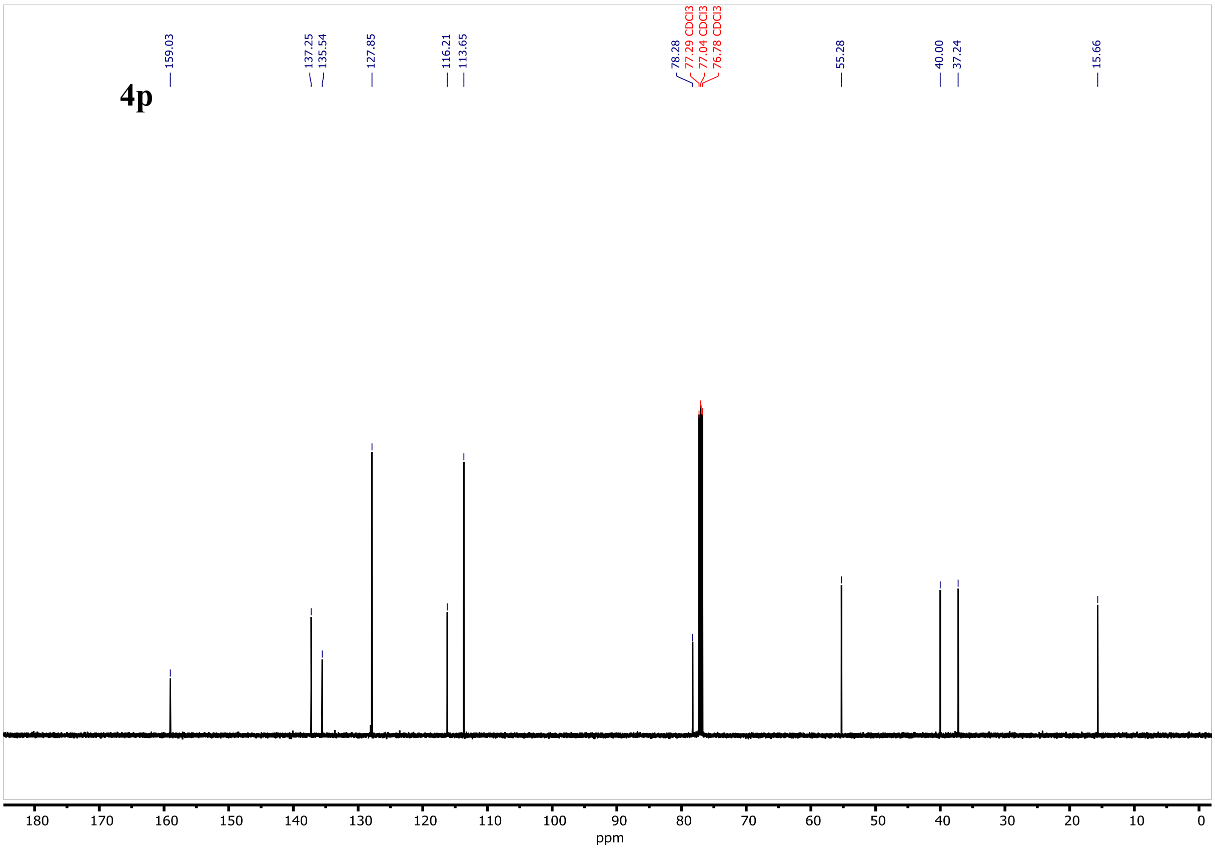

4p

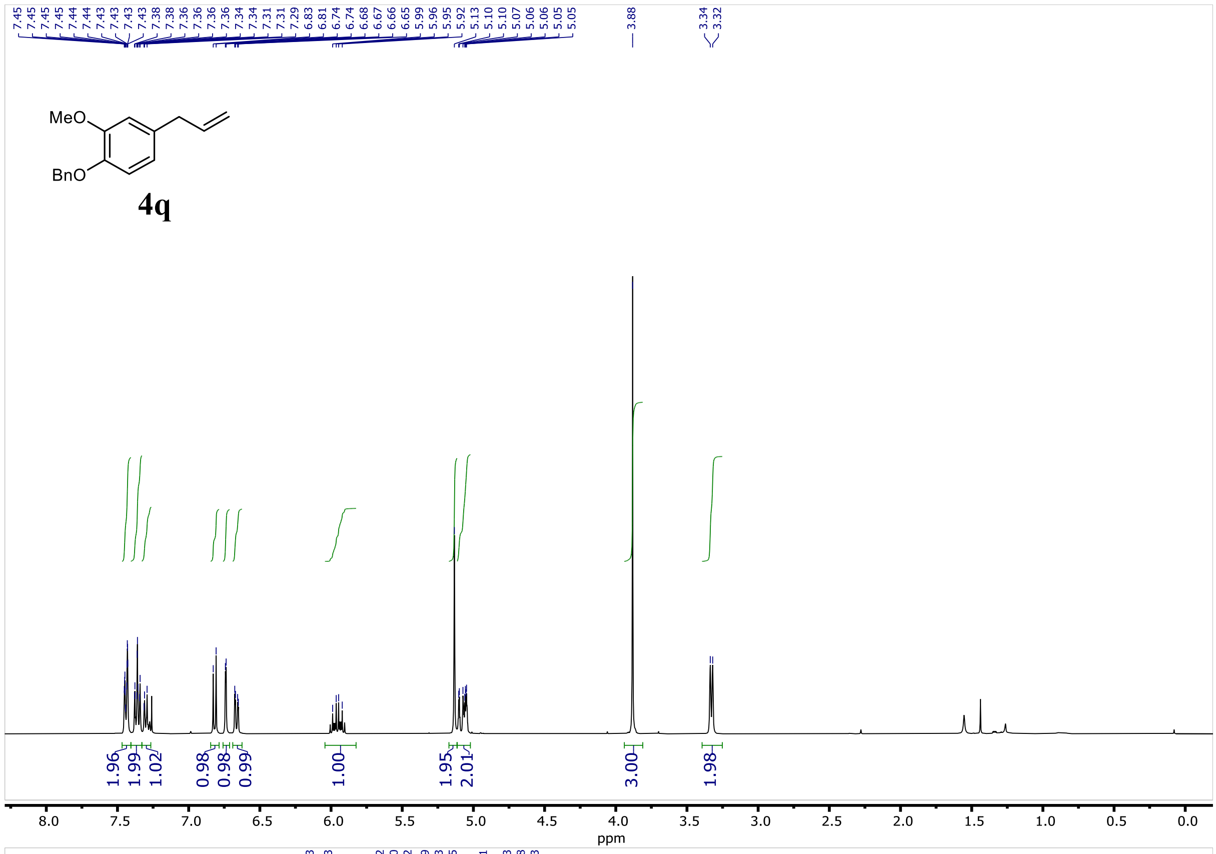

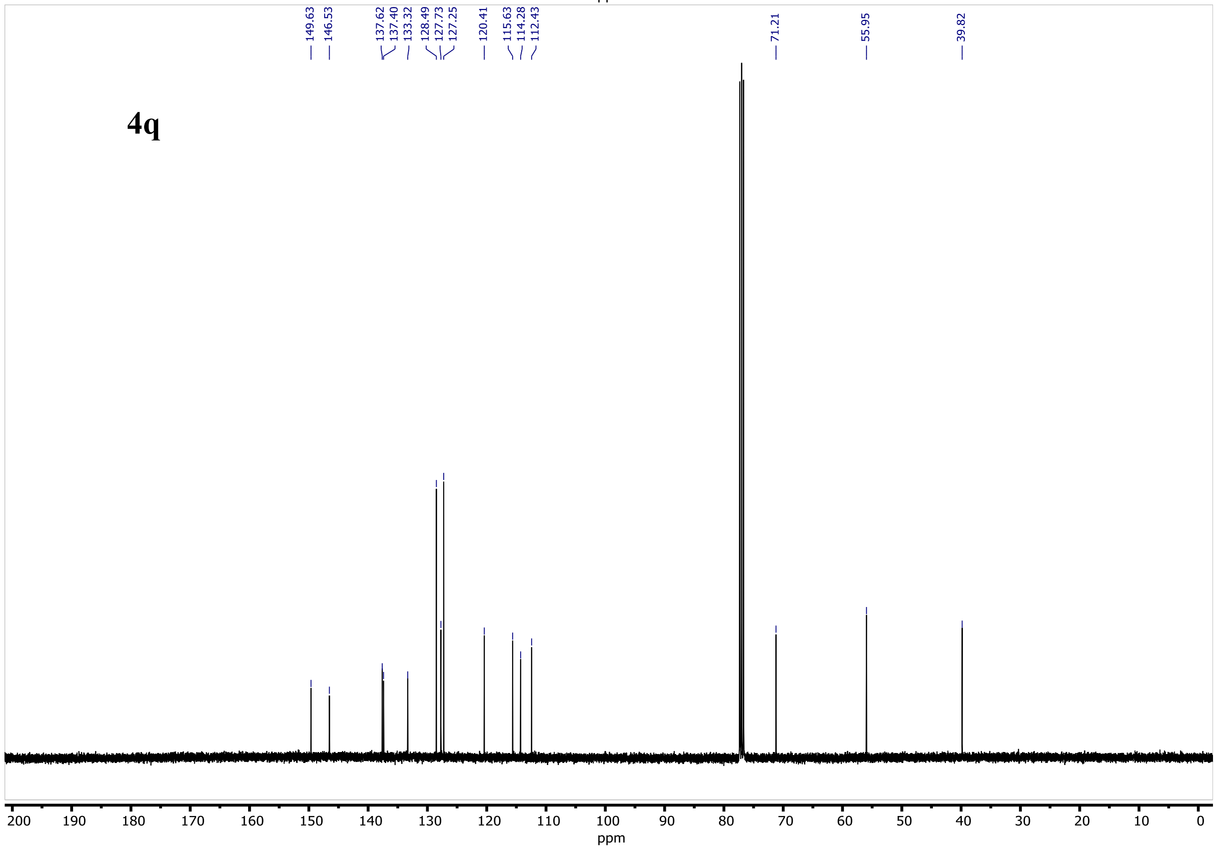

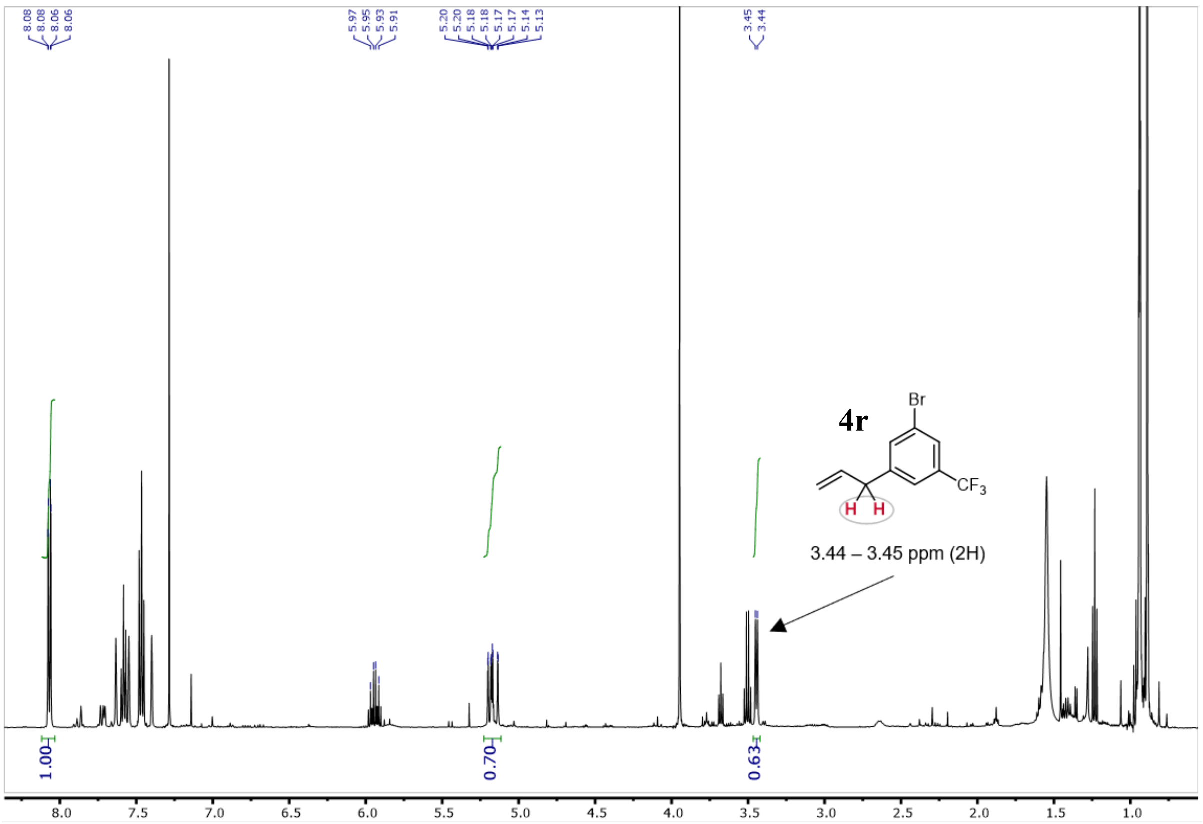
4r
Br
CF3
H H
3.44 – 3.45 ppm (2H)

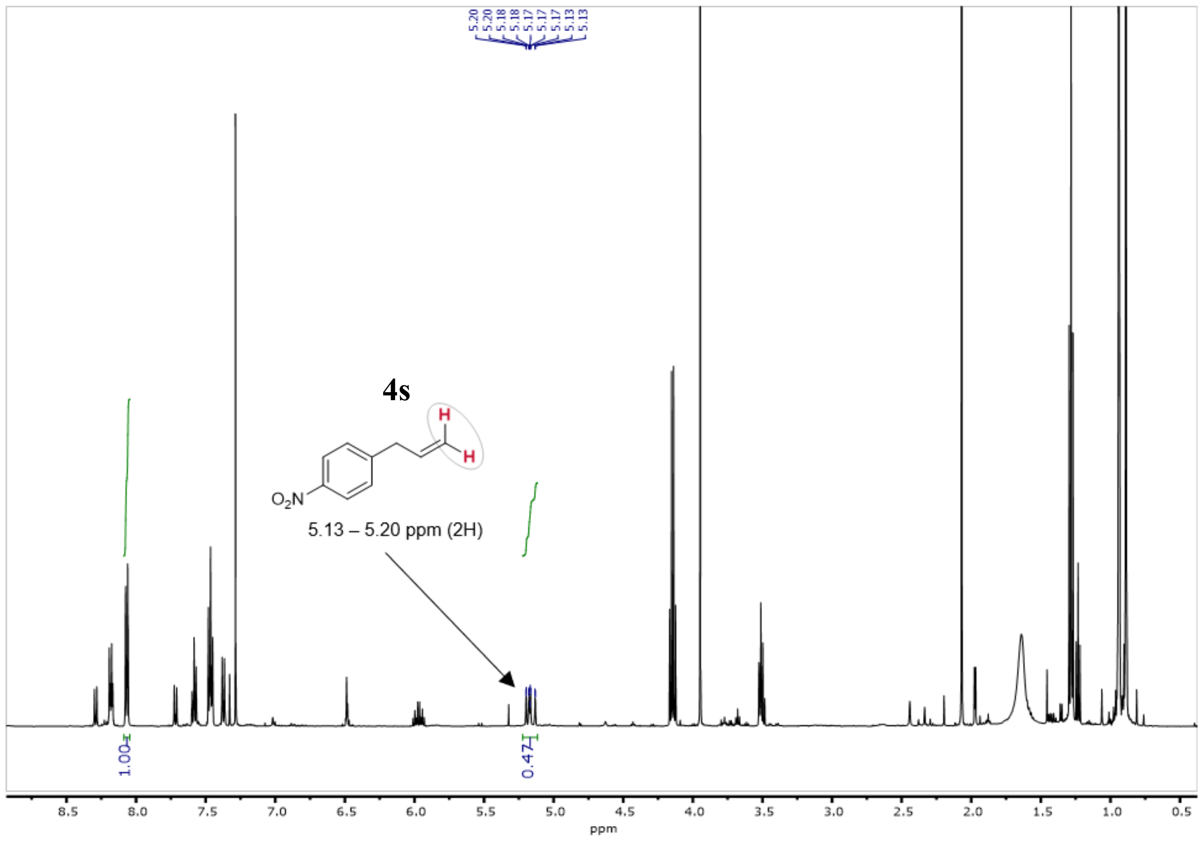
4s
H
H
O2N
5.13 – 5.20 ppm (2H)
ppm

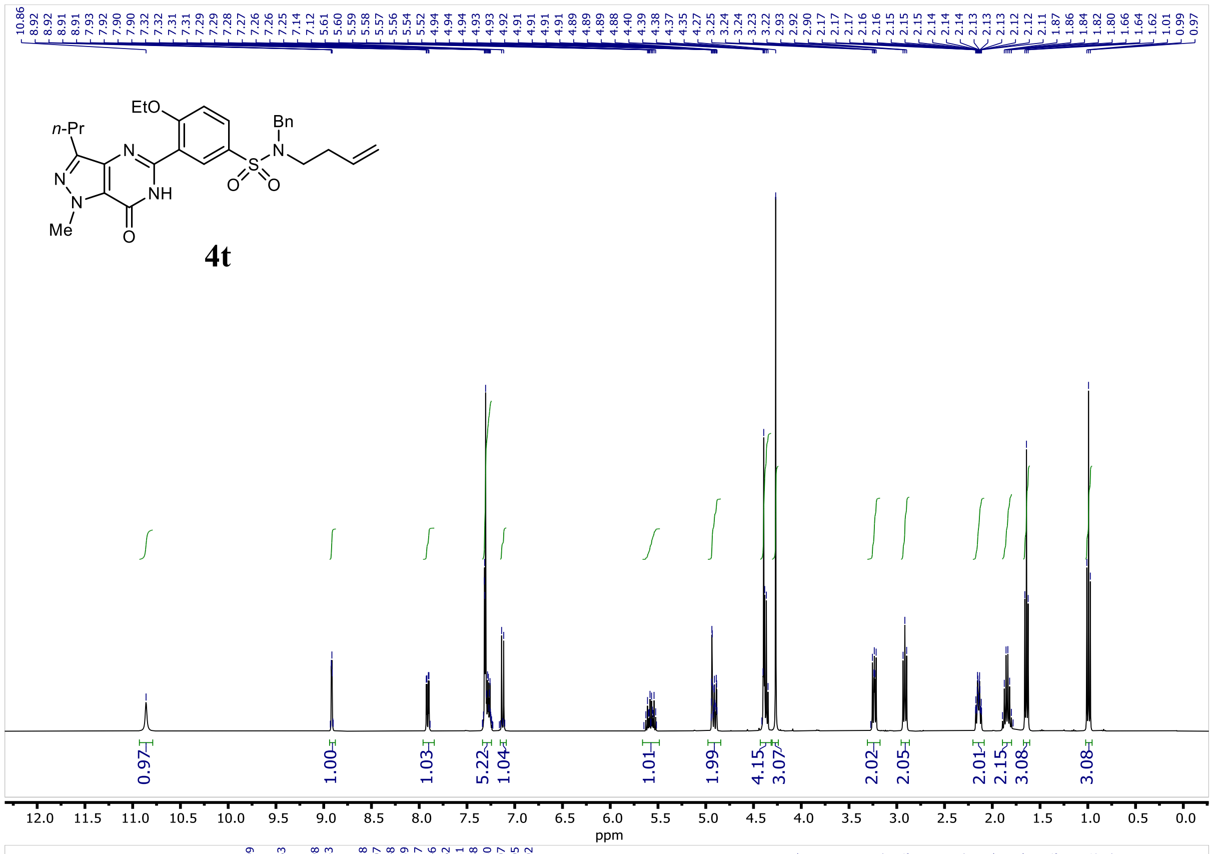

4t

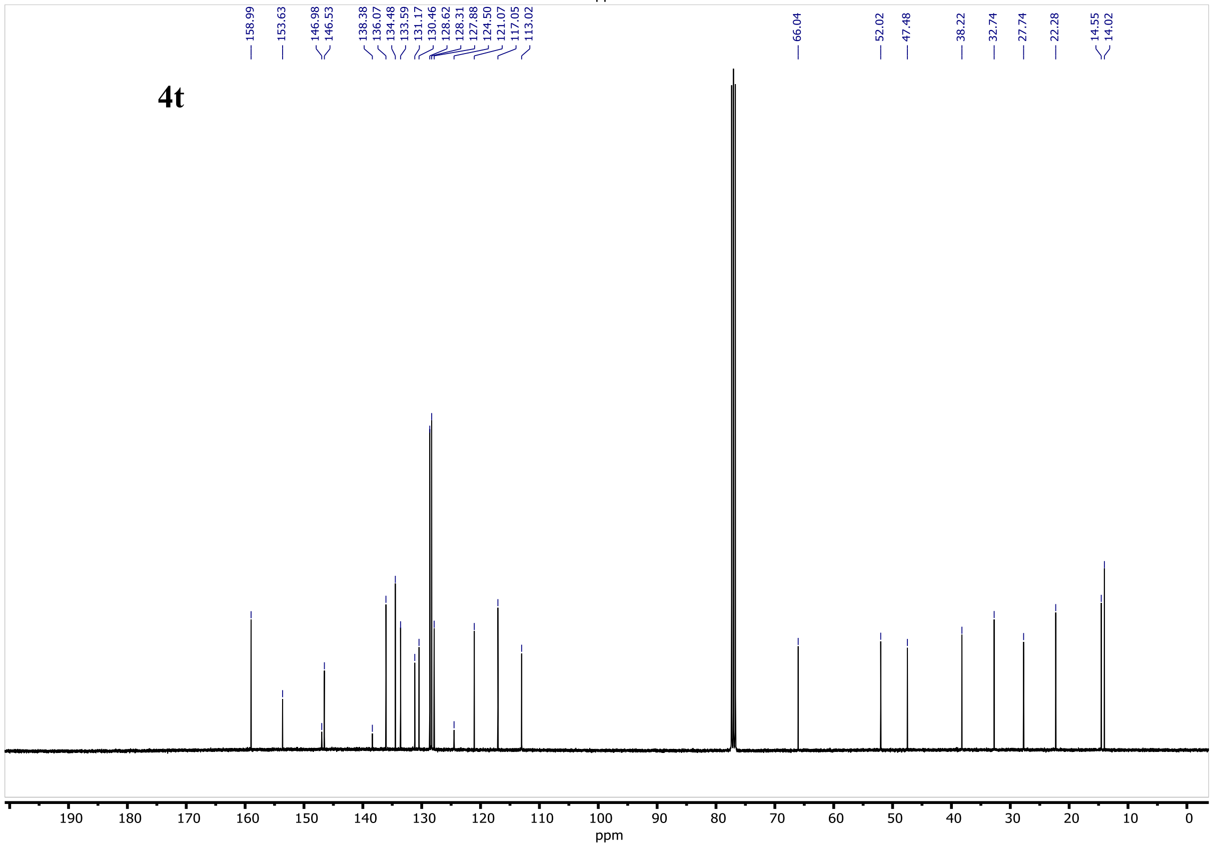

4t

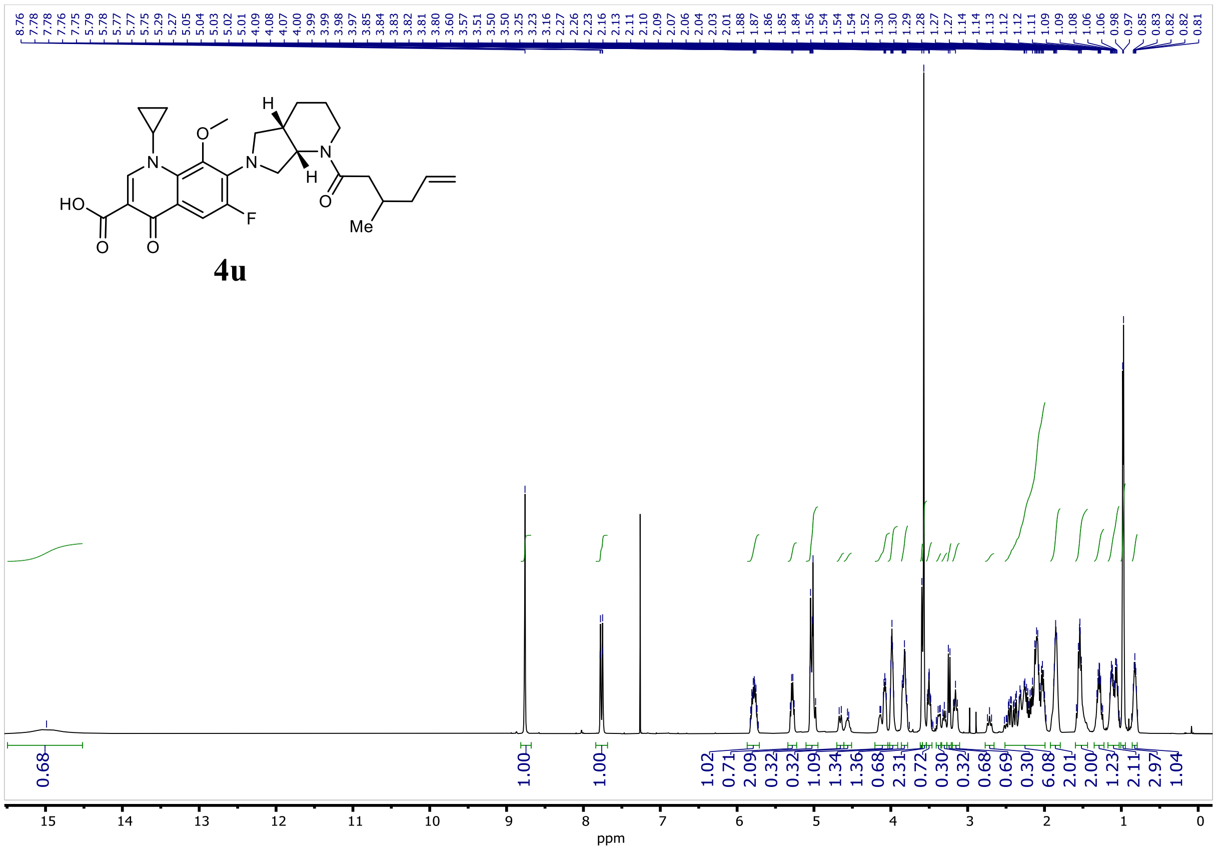

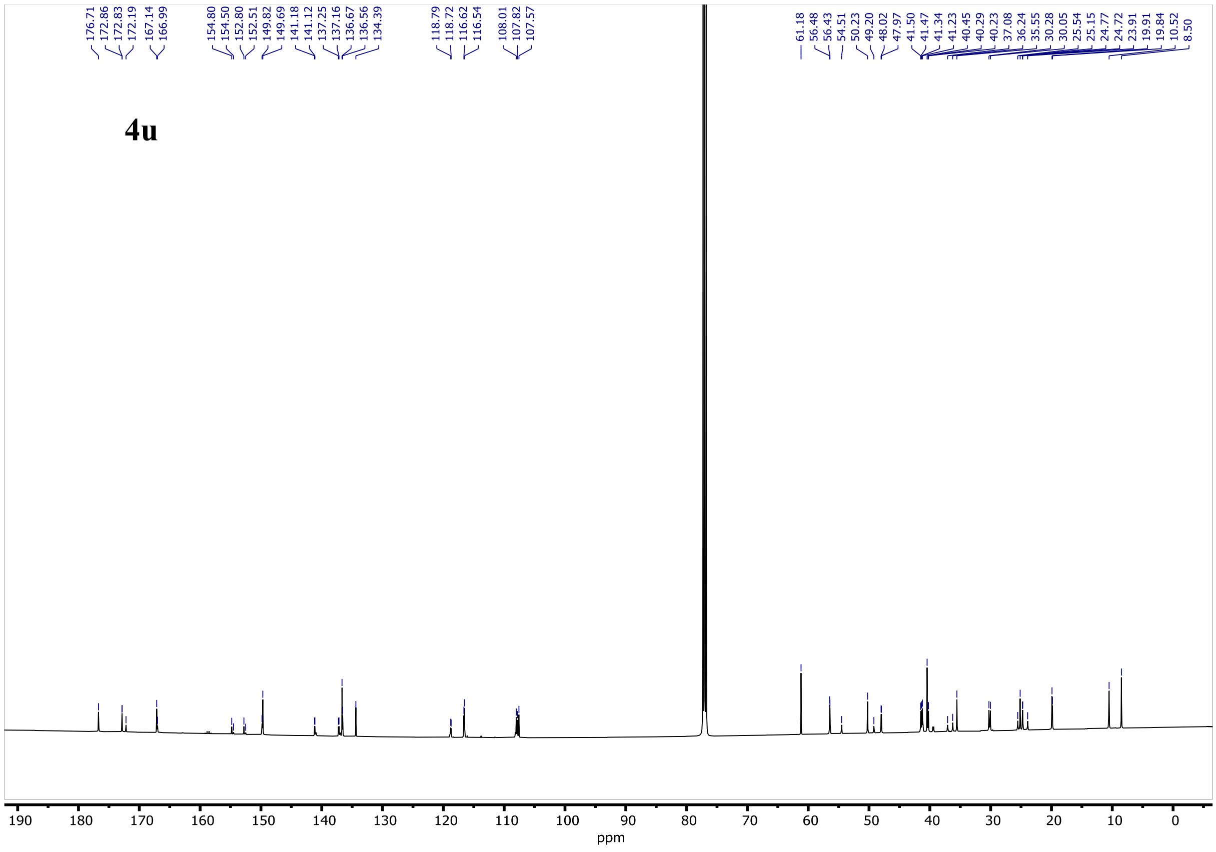

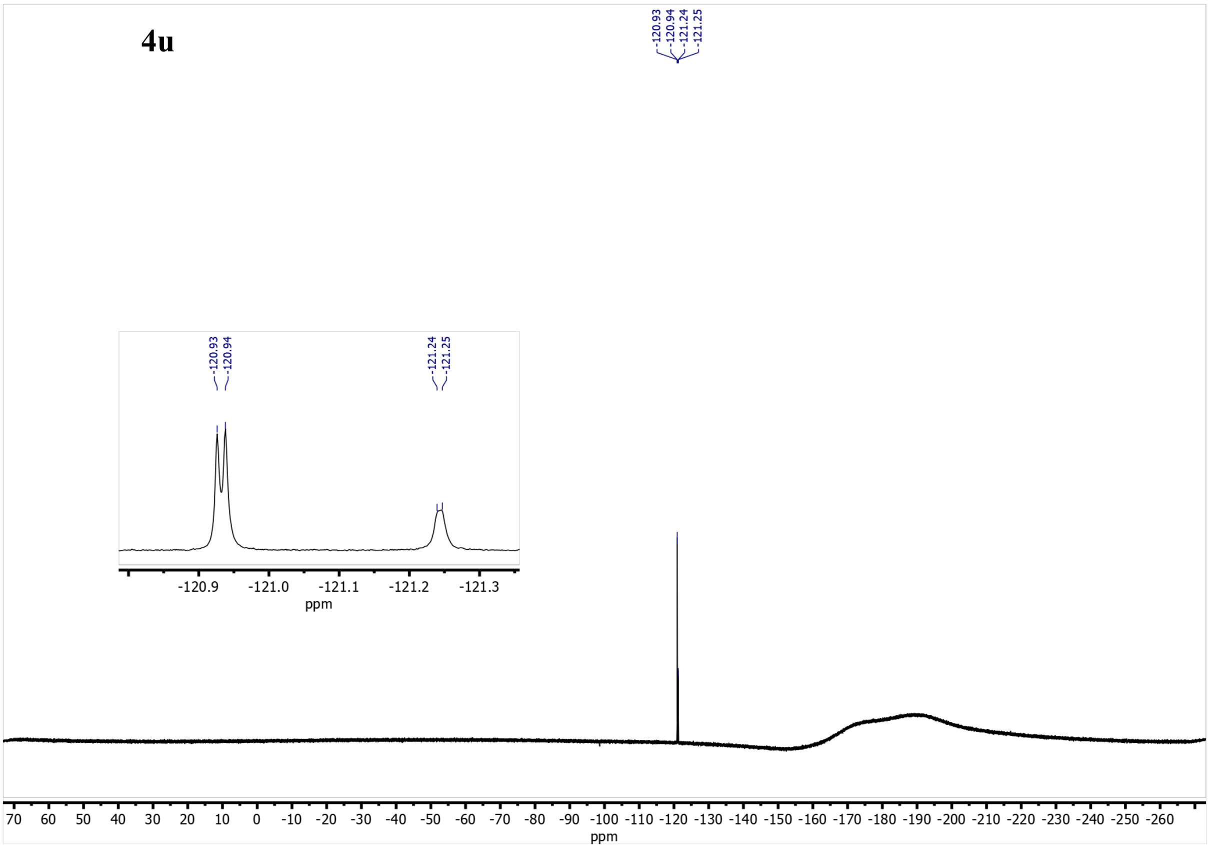
4u
-120.93
-120.94
-121.24
-121.25
-120.9 -121.0 -121.1 -121.2 -121.3
ppm
70 60 50 40 30 20 10 0 -10 -20 -30 -40 -50 -60 -70 -80 -90 -100 -110 -120 -130 -140 -150 -160 -170 -180 -190 -200 -210 -220 -230 -240 -250 -260
ppm

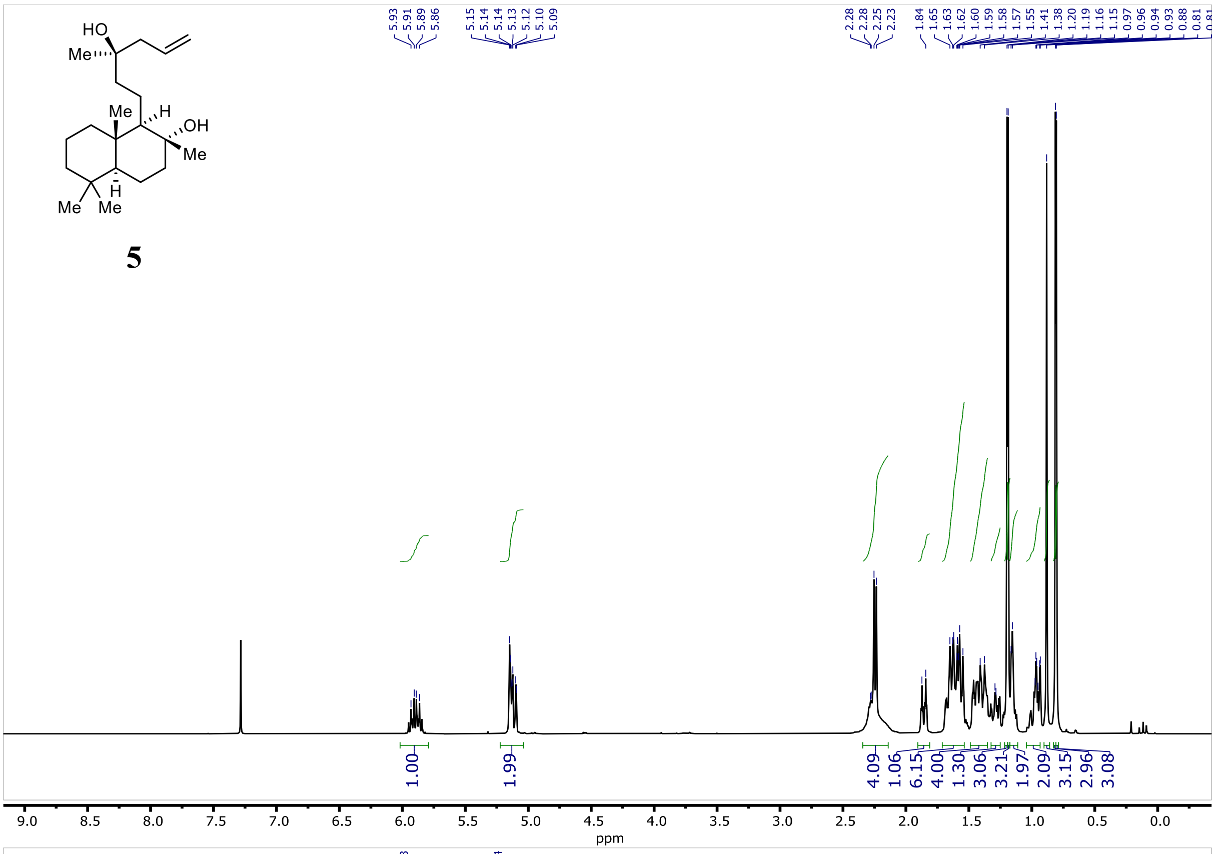
HO
Me
Me
H
OH
Me
H
Me
Me
5
5.93
5.91
5.89
5.86
5.15
5.14
5.14
5.13
5.12
5.10
5.09
2.28
2.28
2.25
2.23
1.84
1.65
1.63
1.62
1.60
1.59
1.58
1.57
1.55
1.41
1.38
1.20
1.19
1.16
1.15
0.97
0.96
0.94
0.93
0.88
0.81
1.00
1.99
4.09
1.06
6.15
4.00
1.30
3.06
3.21
1.97
2.09
3.15
2.96
3.08
ppm

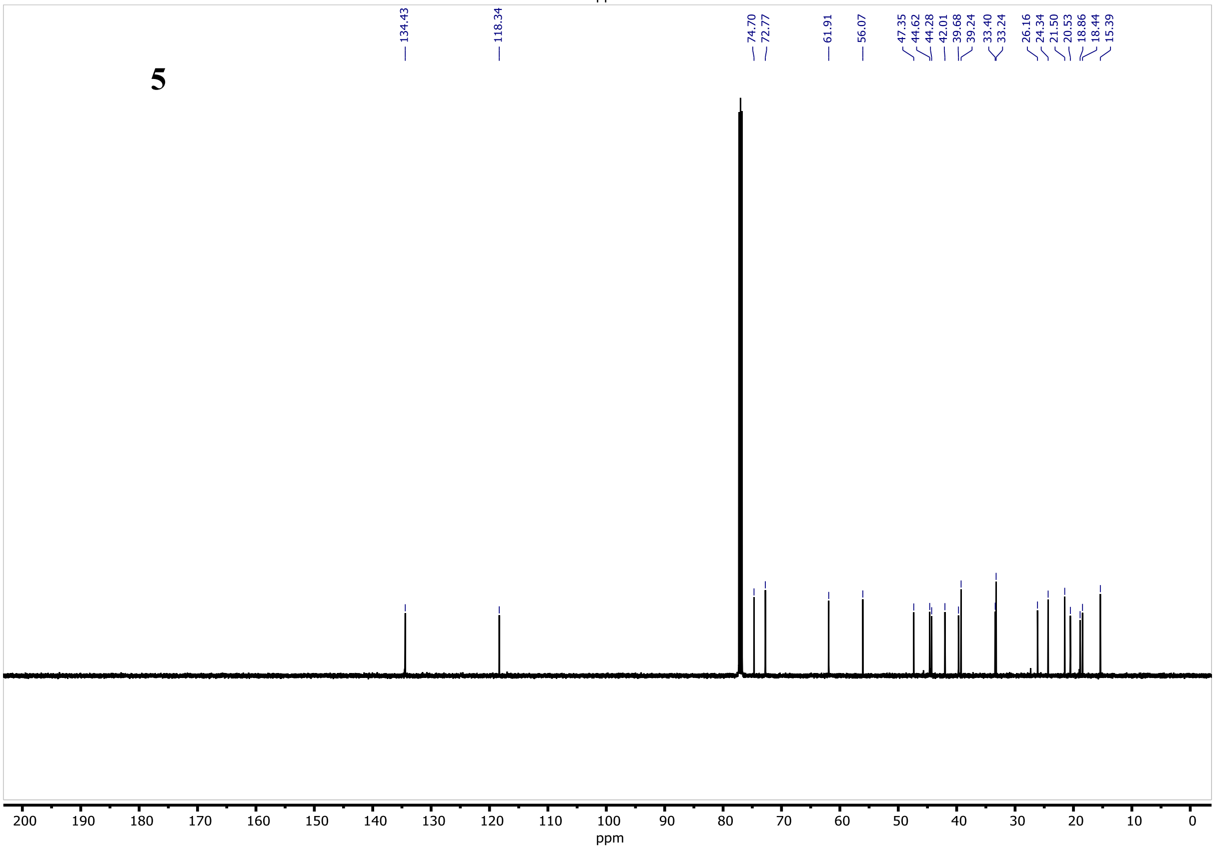
5
134.43
118.34
74.70
72.77
61.91
56.07
47.35
44.62
44.28
42.01
39.68
39.24
33.40
33.24
26.16
24.34
21.50
20.53
18.86
18.44
15.39
ppm

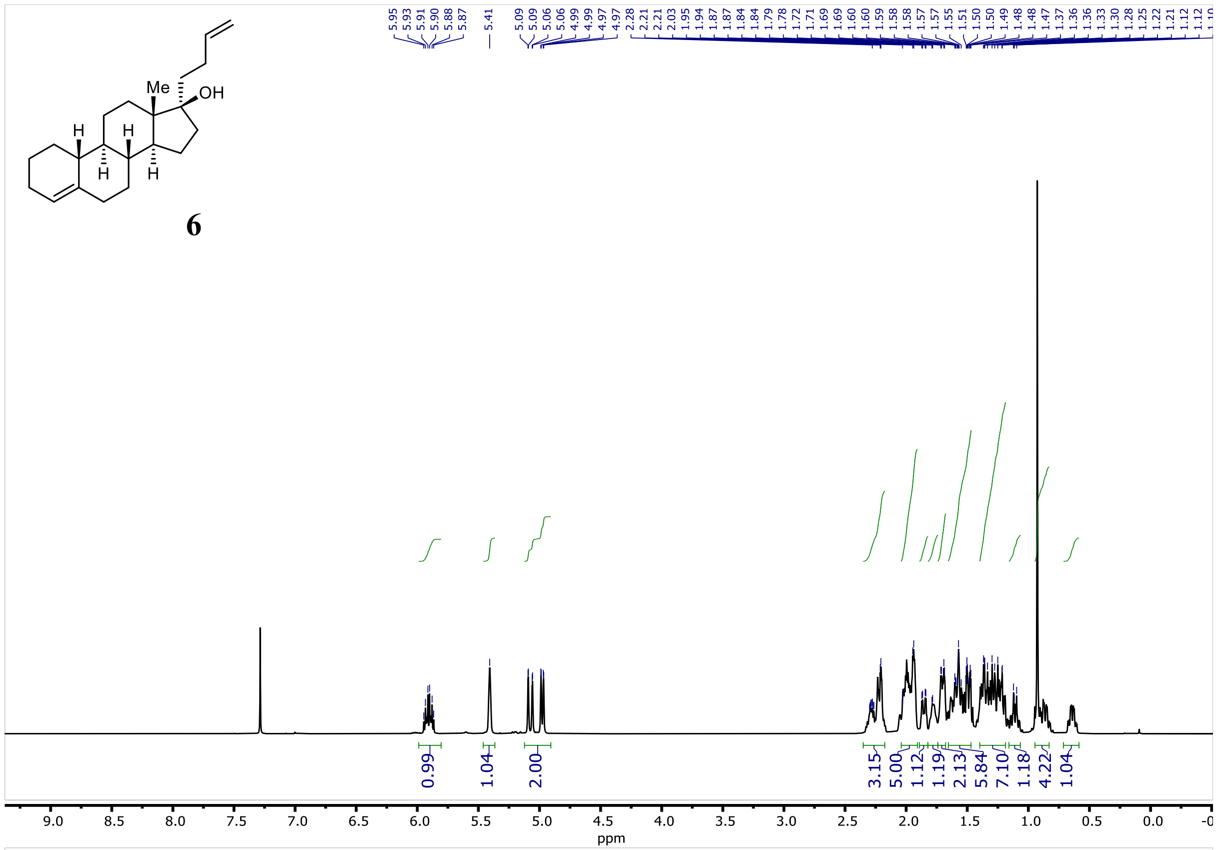
Me
OH
H
H
H
H
6
5.95
5.93
5.91
5.90
5.88
5.87
5.41
5.09
5.09
5.06
5.06
4.99
4.99
4.97
2.28
2.21
2.21
2.03
1.95
1.94
1.87
1.87
1.84
1.84
1.79
1.78
1.72
1.71
1.69
1.69
1.60
1.60
1.59
1.58
1.58
1.57
1.57
1.55
1.51
1.50
1.50
1.49
1.48
1.48
1.47
1.37
1.36
1.36
1.33
1.30
1.28
1.25
1.22
1.21
1.12
1.12
0.99
1.04
2.00
3.15
5.00
1.12
1.19
2.13
5.84
7.10
1.18
4.22
1.04
ppm

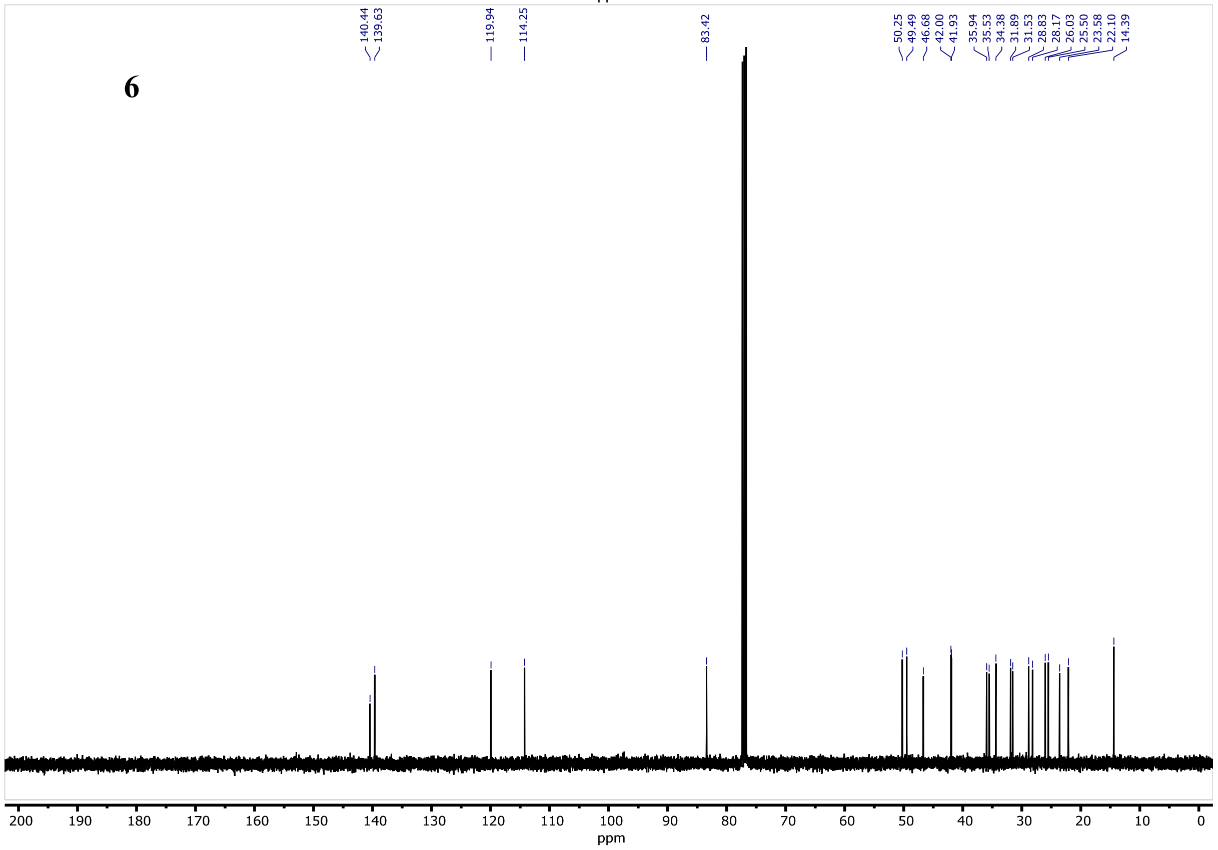
6
140.44
139.63
119.94
114.25
83.42
50.25
49.49
46.68
42.00
41.93
35.94
35.53
34.38
31.89
31.53
28.83
28.17
26.03
25.50
23.58
22.10
14.39
ppm

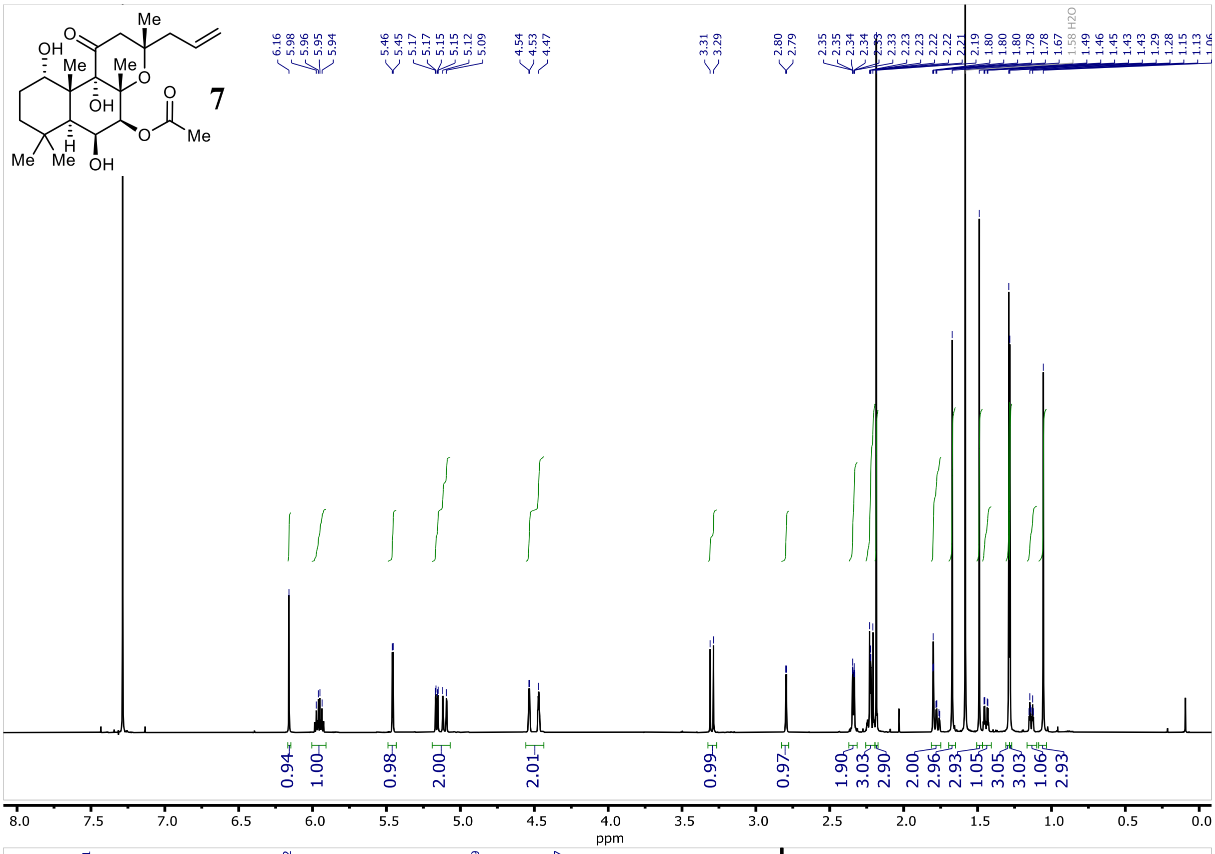
7
ppm
8.0 7.5 7.0 6.5 6.0 5.5 5.0 4.5 4.0 3.5 3.0 2.5 2.0 1.5 1.0 0.5 0.0
0.94 1.00 0.98 2.00 2.01 0.99 0.97 1.90 3.03 2.90 2.00 2.96 2.93 1.05 3.05 3.03 1.06 2.93

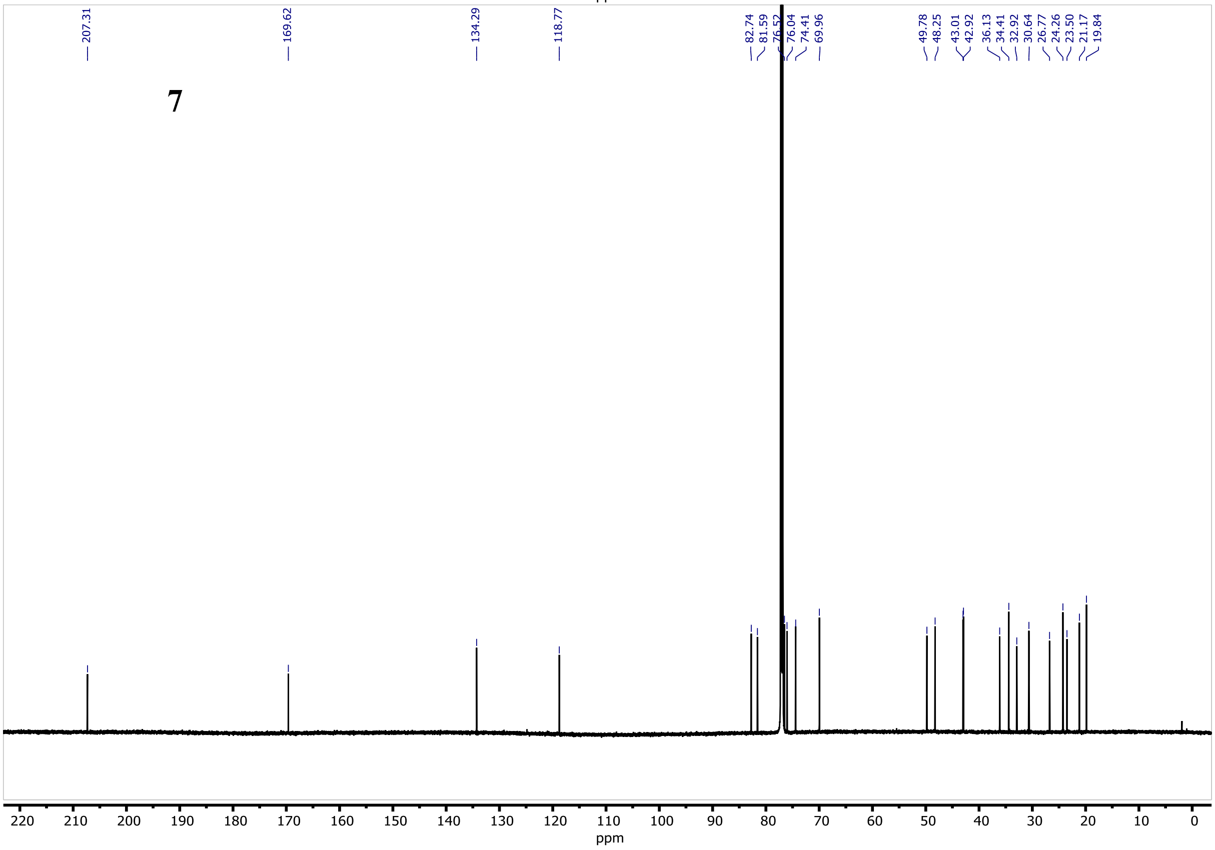
7
207.31 169.62 134.29 118.77 82.74 81.59 76.52 76.04 74.41 69.96 49.78 48.25 43.01 42.92 36.13 34.41 32.92 30.64 26.77 24.26 23.50 21.17 19.84
ppm
220 210 200 190 180 170 160 150 140 130 120 110 100 90 80 70 60 50 40 30 20 10 0

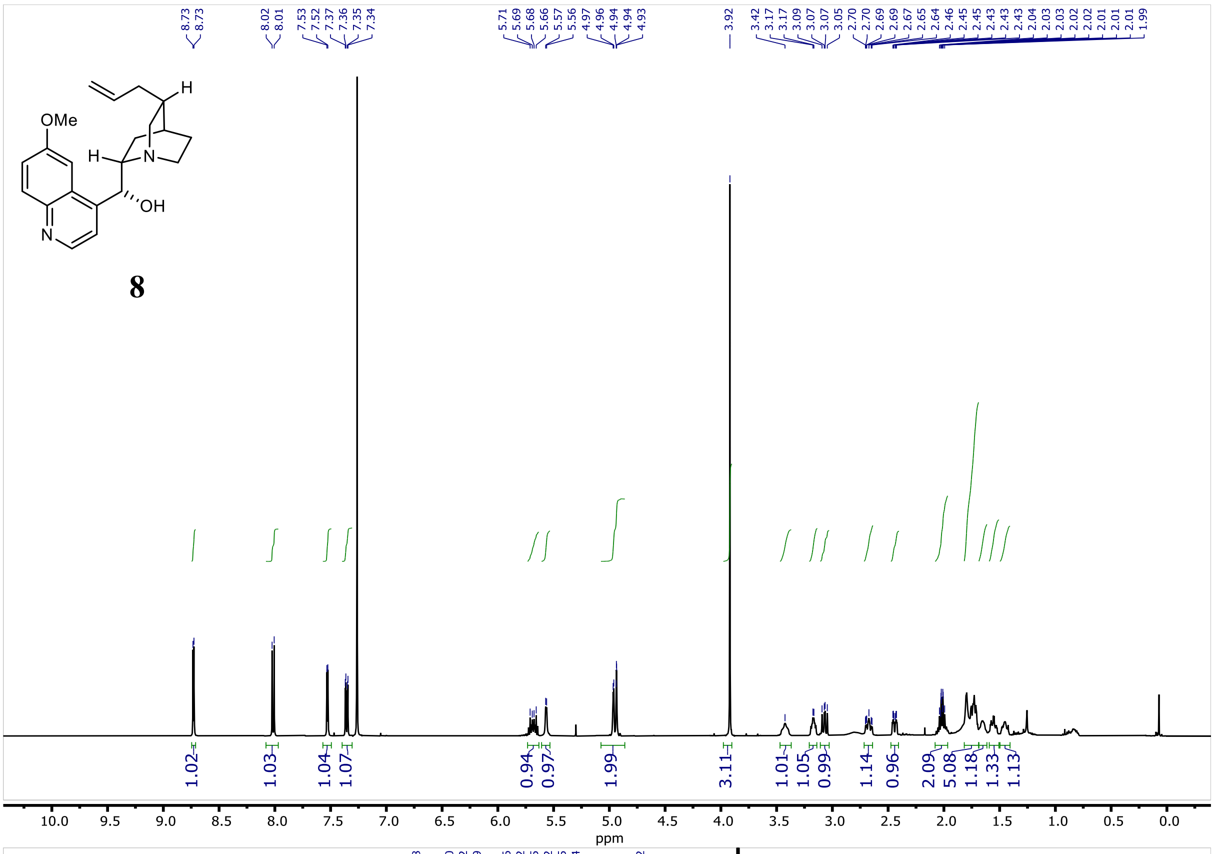
OMe
H
N
OH
N
8.73
8.02
8.01
7.53
7.52
7.37
7.36
7.35
7.34
5.71
5.69
5.68
5.66
5.57
5.56
4.97
4.96
4.94
4.94
4.93
3.92
3.42
3.17
3.17
3.09
3.07
3.07
3.05
1.02
1.03
1.04
1.07
0.94
0.97
1.99
3.11
1.01
1.05
0.99
1.14
0.96
2.09
5.08
1.18
1.33
1.13
ppm

8

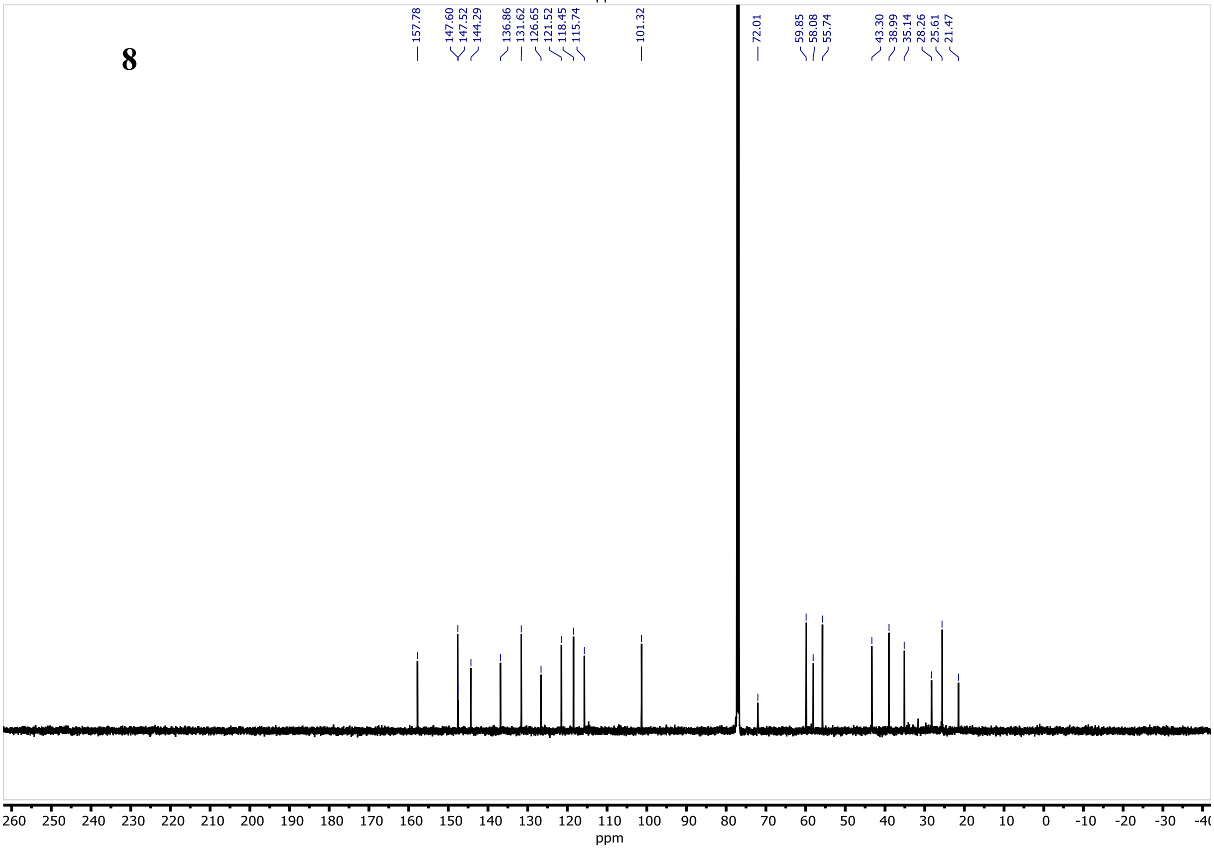
157.78
147.60
147.52
144.29
136.86
131.62
126.65
121.52
118.45
115.74
101.32
72.01
59.85
58.08
55.74
43.30
38.99
35.14
28.26
25.61
21.47
ppm

8

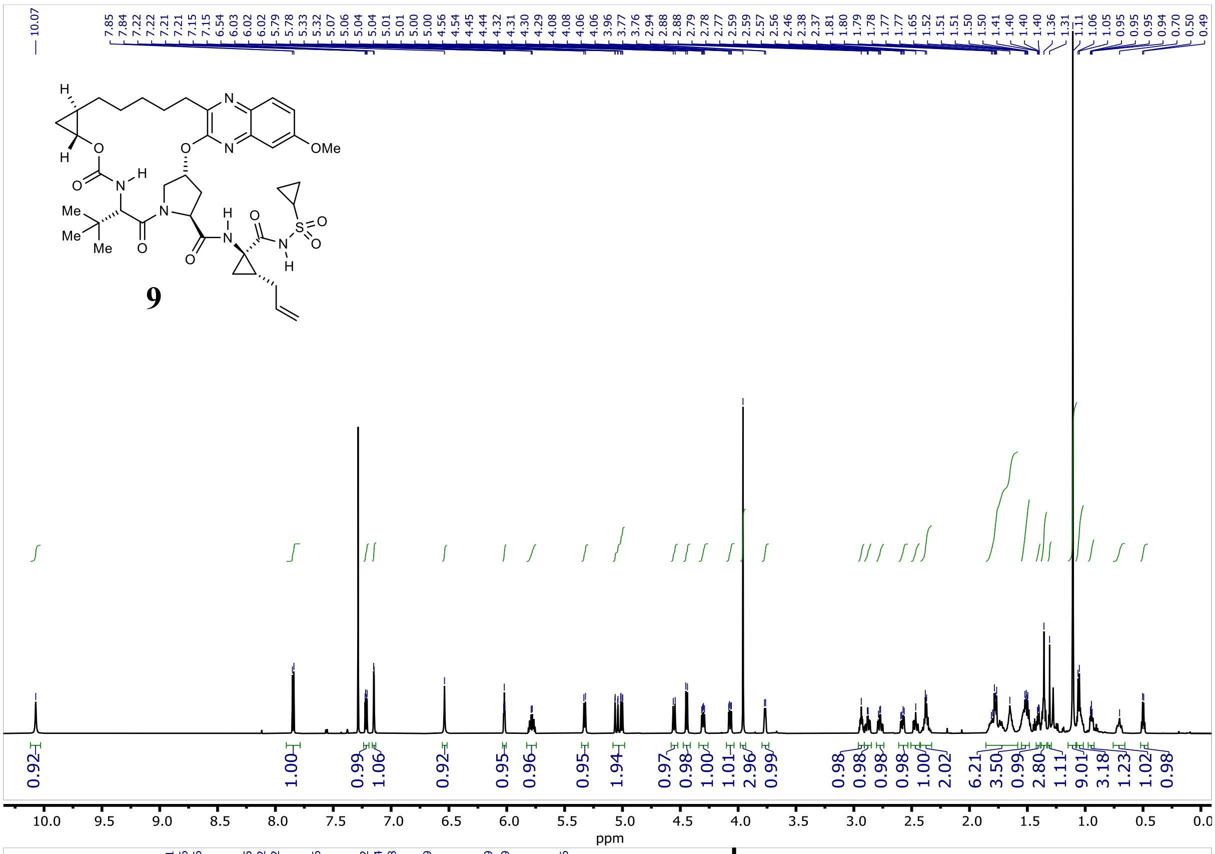

9

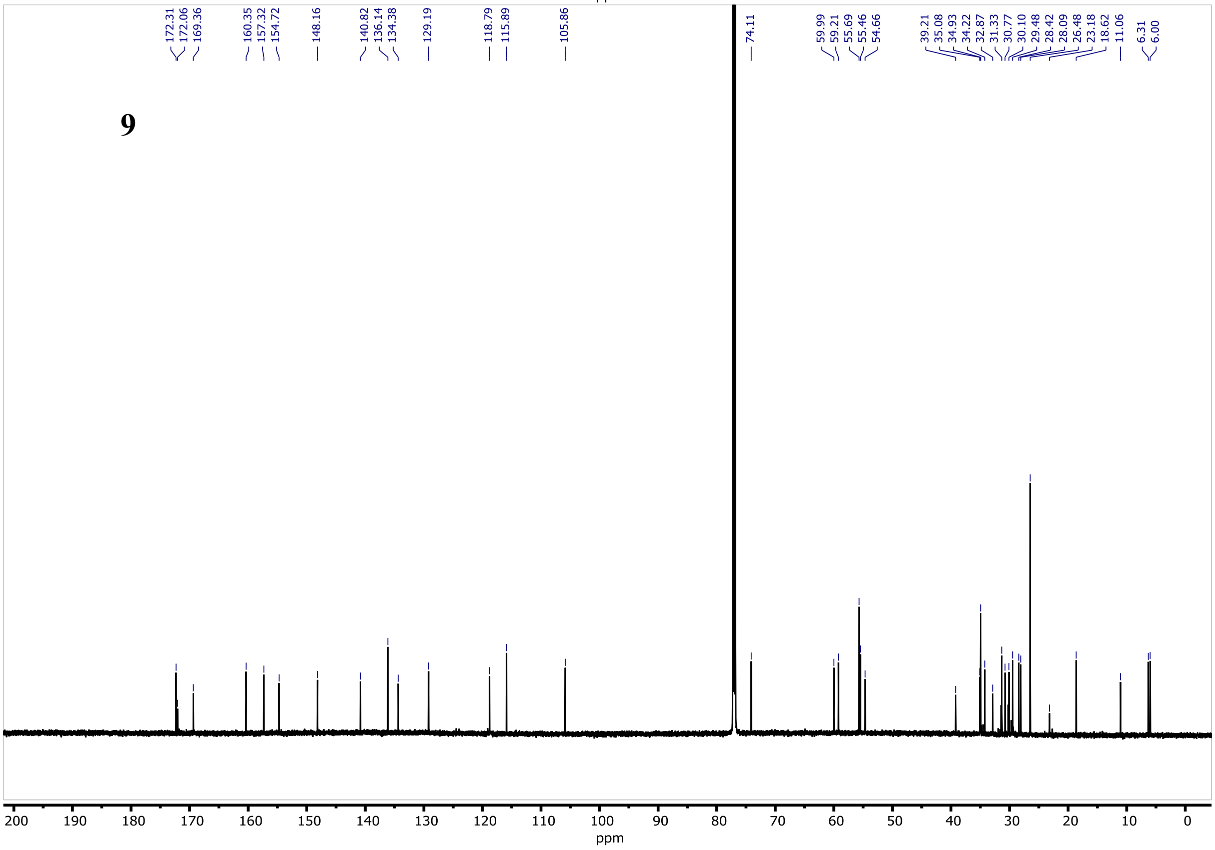

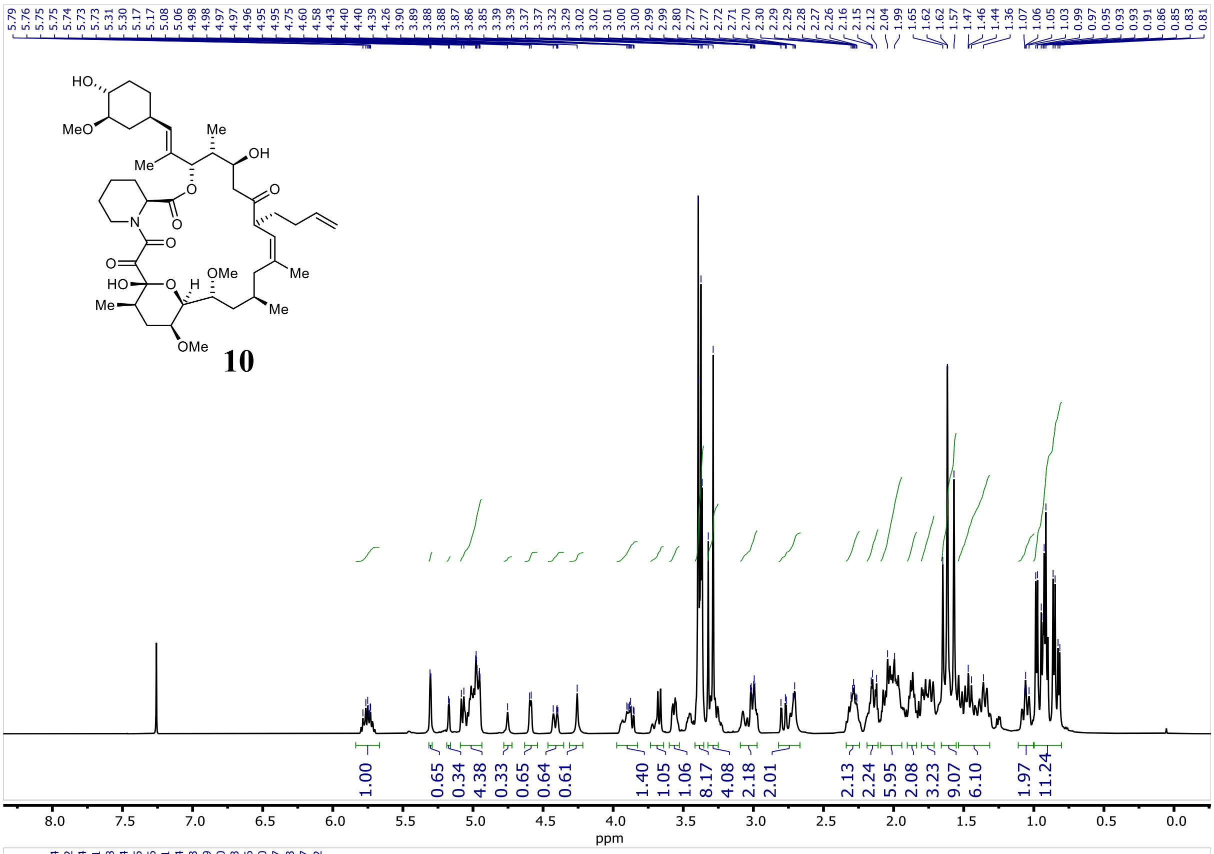

10

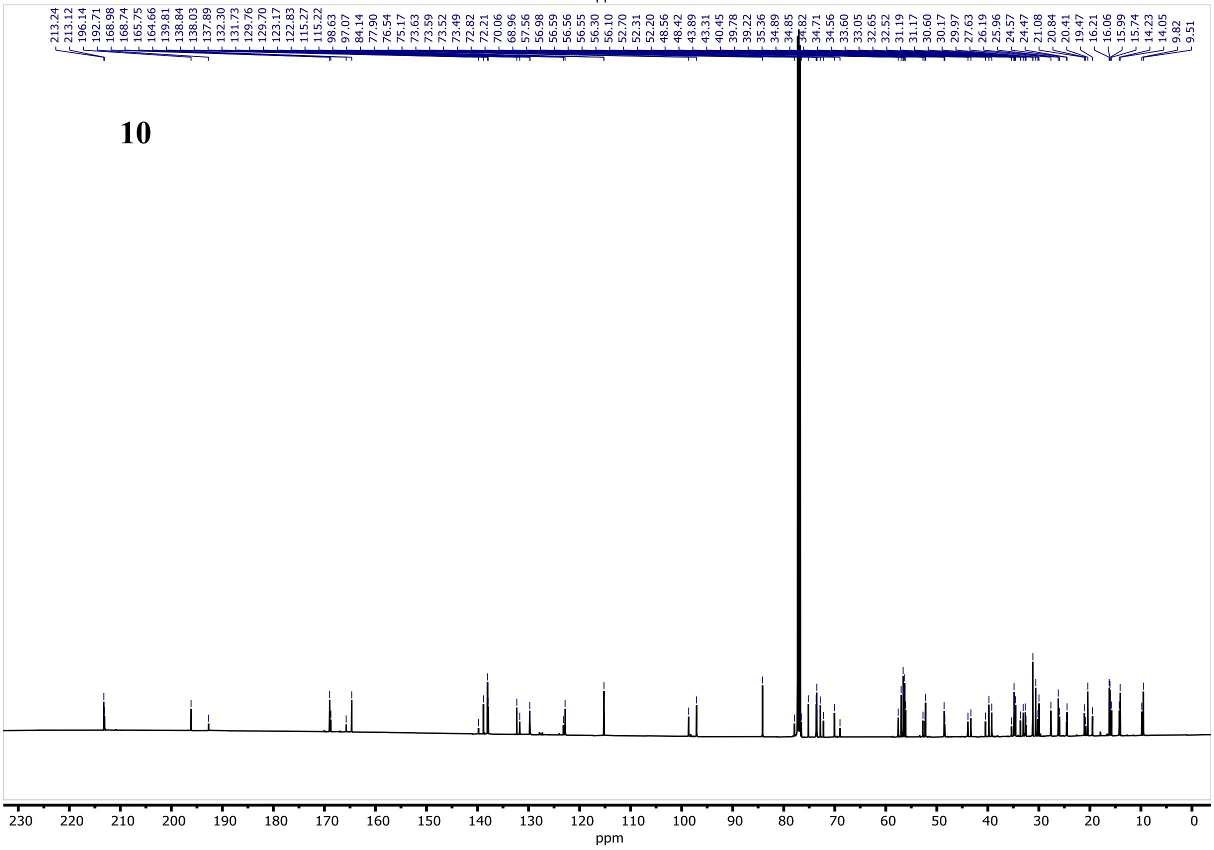

10

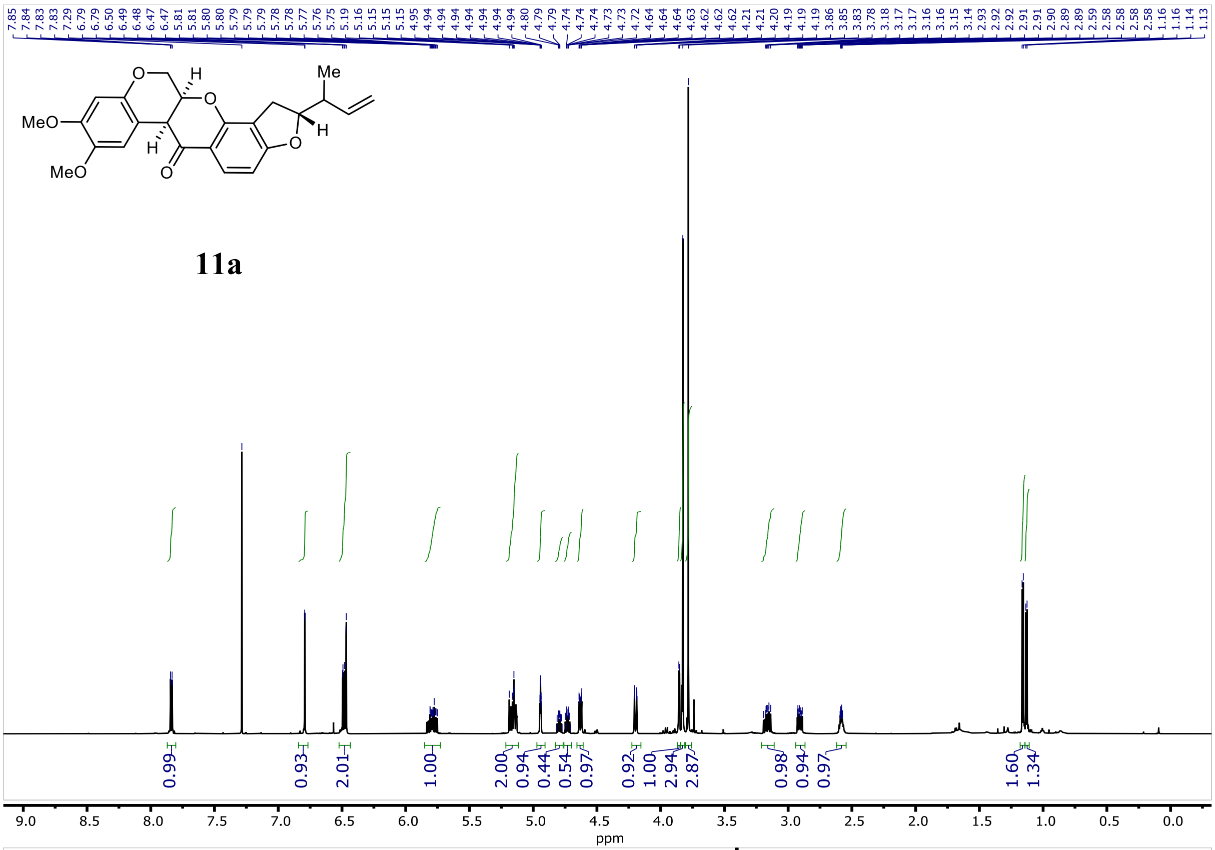
MeO
MeO
Me
H
O
11a

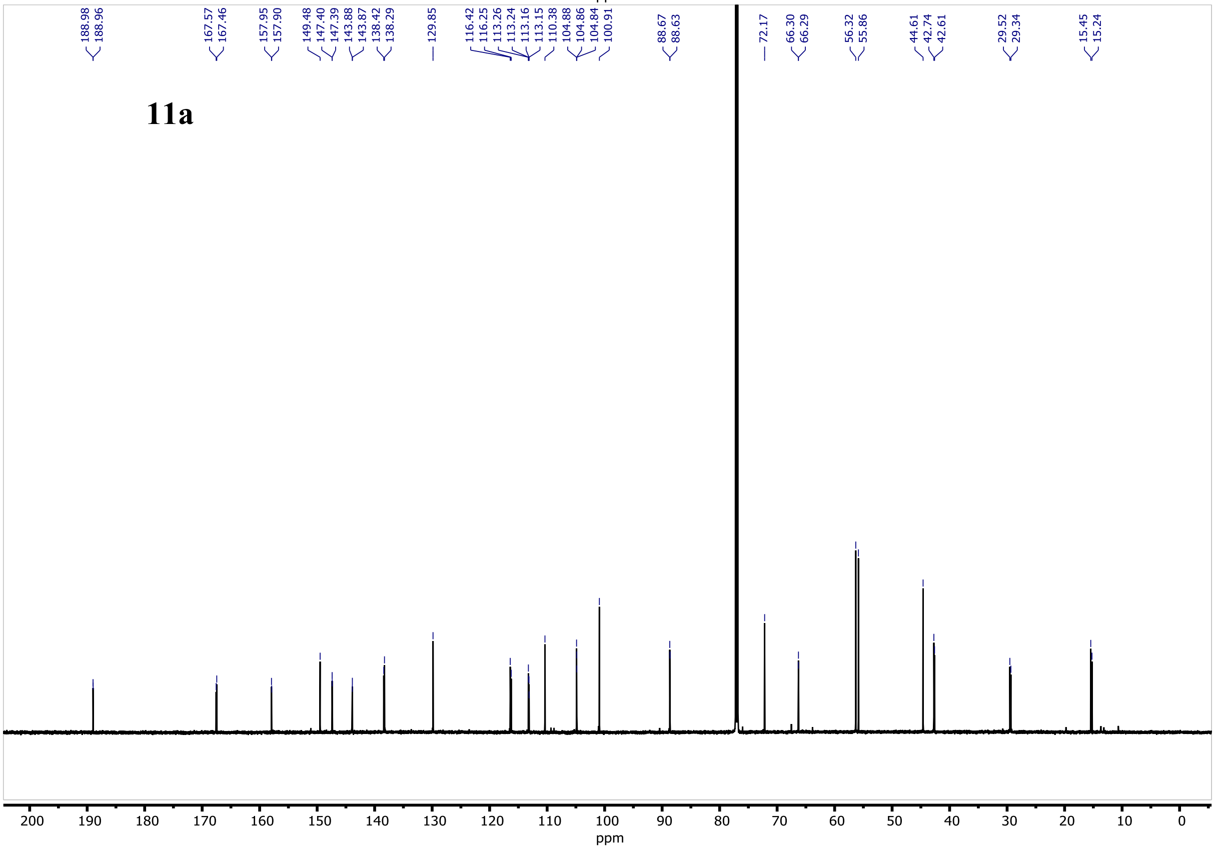
188.98
188.96
167.57
167.46
157.95
157.90
149.48
147.40
147.39
143.88
143.87
138.42
138.29
129.85
116.42
116.25
113.26
113.24
113.16
113.15
110.38
104.88
104.86
104.84
100.91
88.67
88.63
72.17
66.30
66.29
56.32
55.86
44.61
42.74
42.61
29.52
29.34
15.45
15.24
11a
ppm

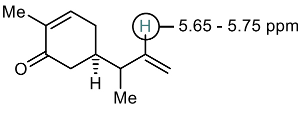
Me
H
5.65 - 5.75 ppm
O
H
Me

11b

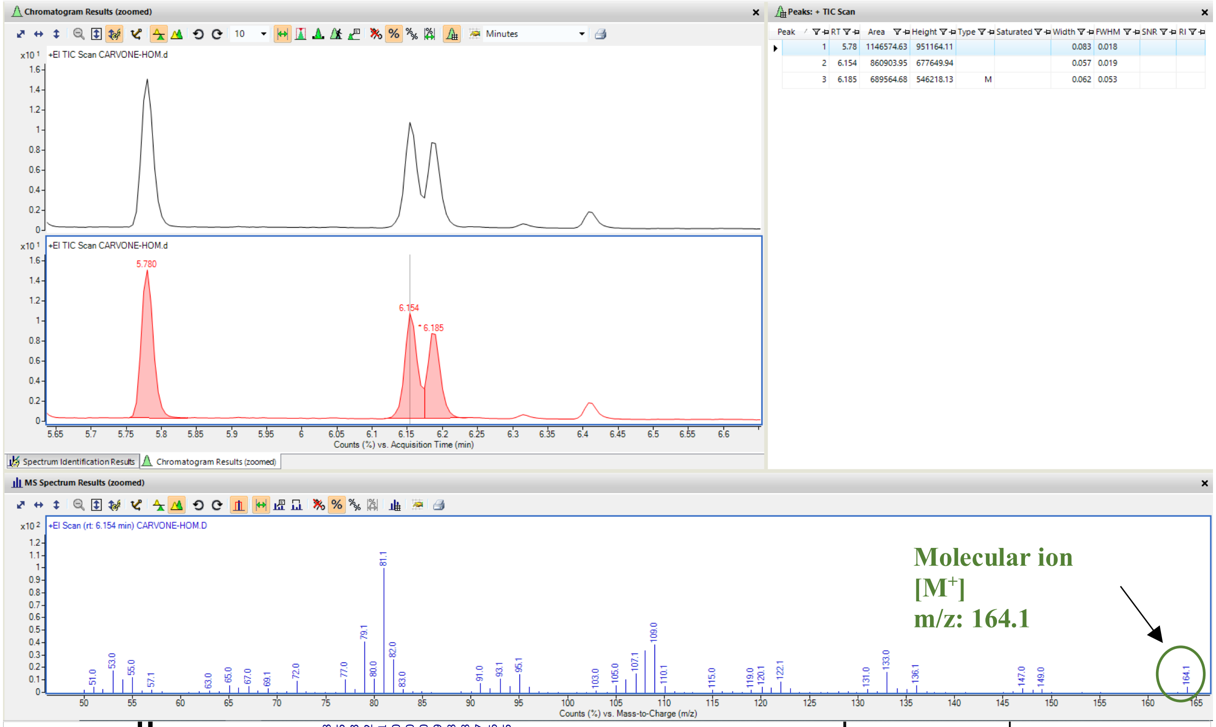
Chromatogram Results (zoomed)
+EI TIC Scan CARVONE-HOM.d
5.780
6.154
6.185
Counts (%) vs. Acquisition Time (min)
Peaks: + TIC Scan
Peak RT Area Height Type Saturated Width FWHM SNR RI
1 5.78 1146574.63 951164.11 0.083 0.018
2 6.154 860903.95 677649.94 0.057 0.019
3 6.185 689564.68 546218.13 M 0.062 0.053
MS Spectrum Results (zoomed)
+EI Scan (rt: 6.154 min) CARVONE-HOM.D
Counts (%) vs. Mass-to-Charge (m/z)
Molecular ion
[M+]
m/z: 164.1

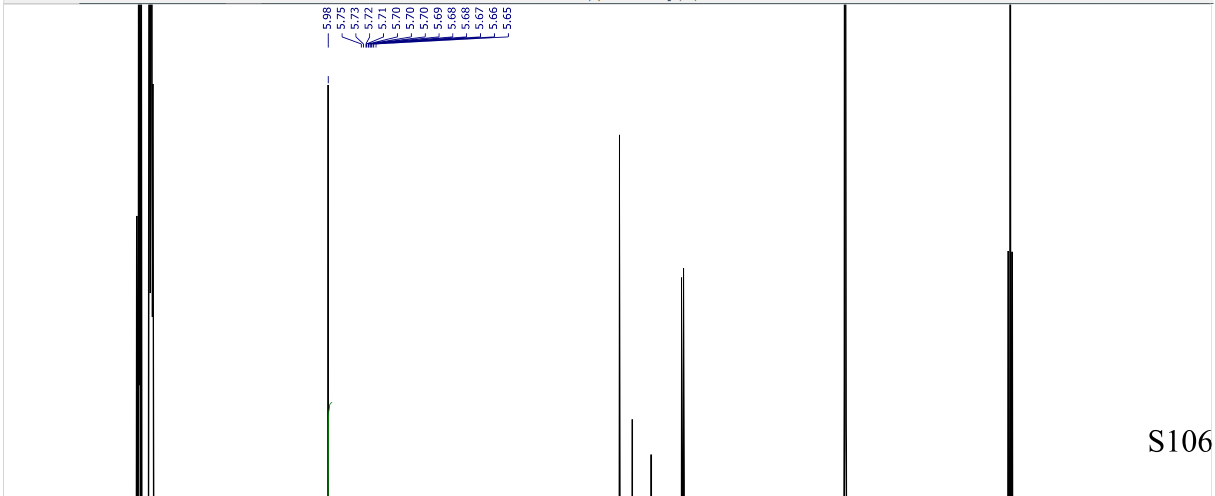
5.98
5.75
5.73
5.72
5.71
5.70
5.70
5.69
5.68
5.68
5.67
5.66
5.65

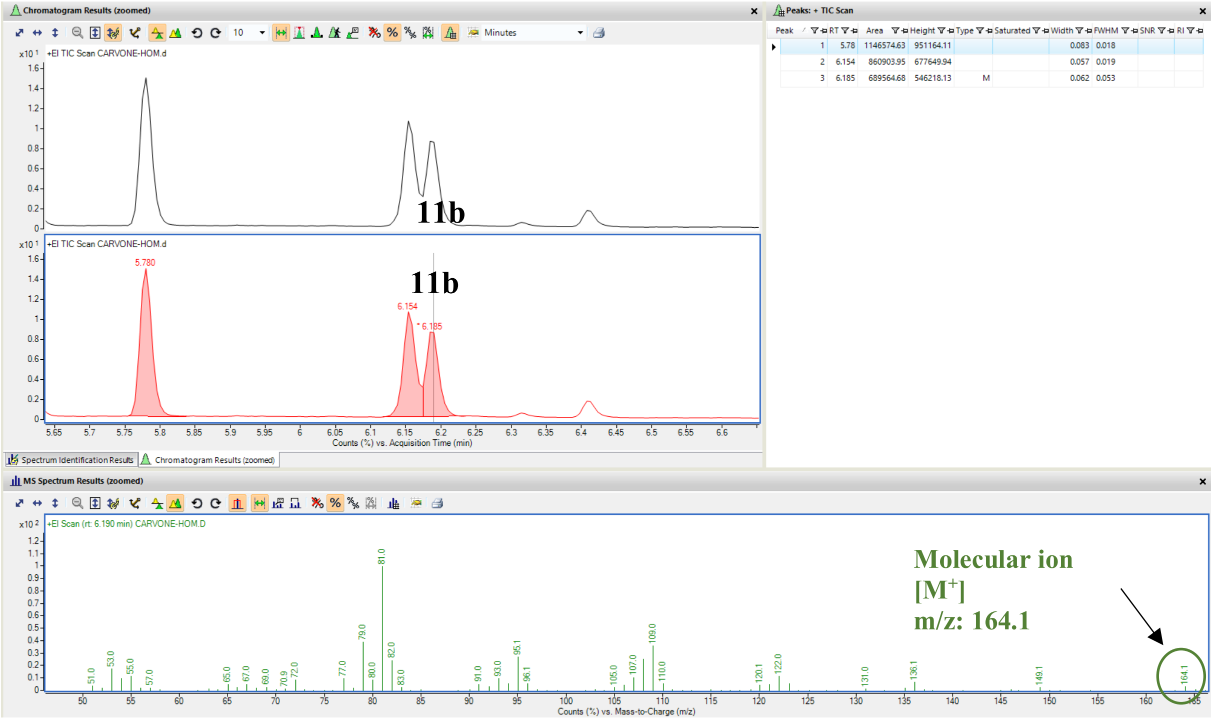
Chromatogram Results (zoomed)
+EI TIC Scan CARVONE-HOM.d
11b
5.780
6.154
6.185
Counts (%) vs. Acquisition Time (min)
Peaks: + TIC Scan
MS Spectrum Results (zoomed)
+EI Scan (rt: 6.190 min) CARVONE-HOM.D
Molecular ion
[M+]
m/z: 164.1
Counts (%) vs. Mass-to-Charge (m/z)

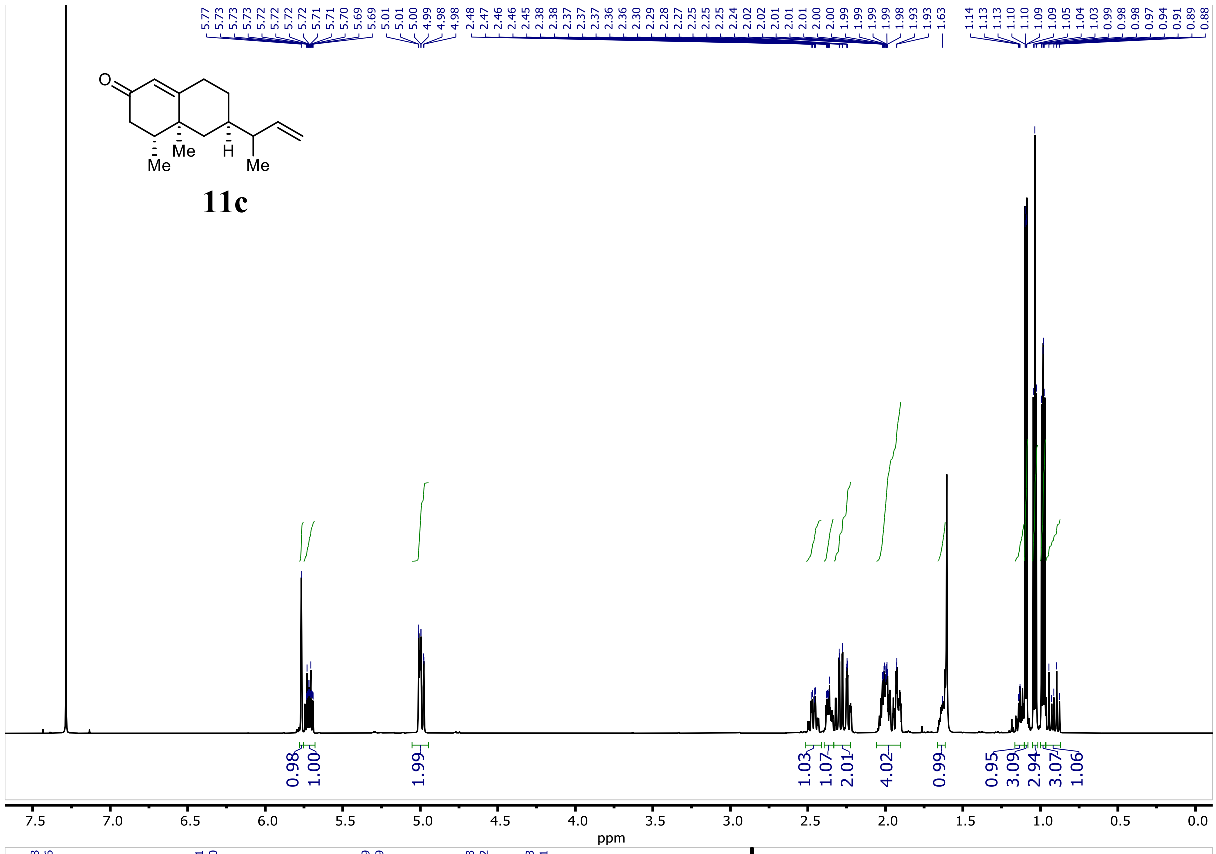

11c

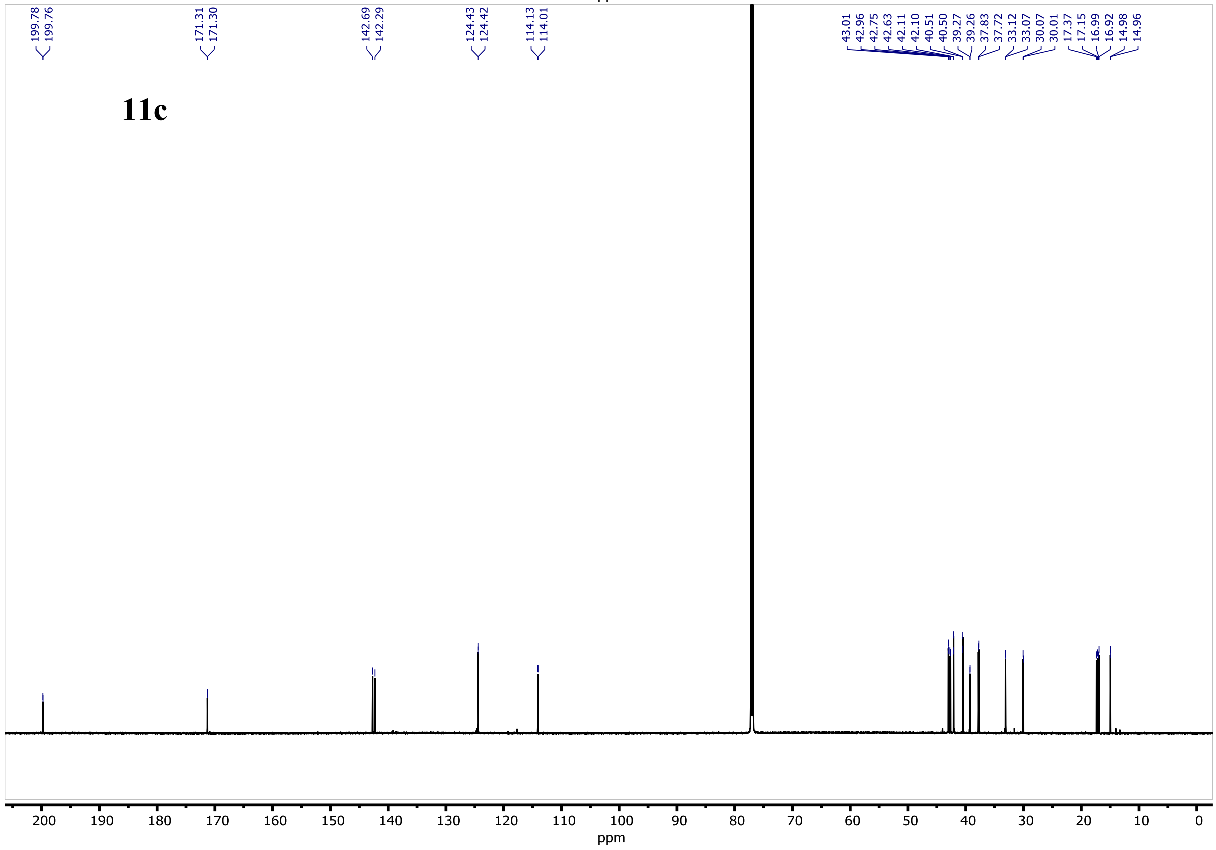

11c

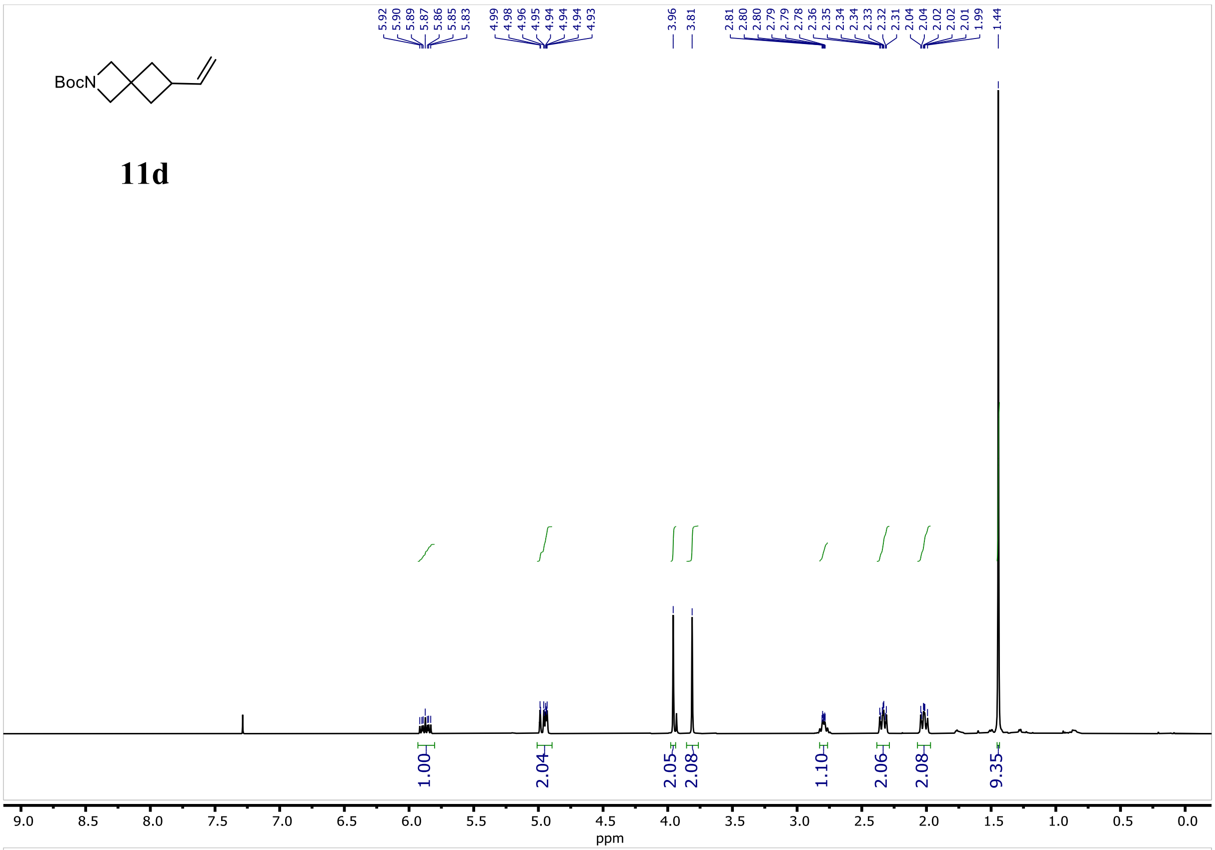
BocN
11d
5.92 5.90 5.89 5.87 5.86 5.85 5.83
4.99 4.98 4.96 4.95 4.94 4.94 4.94 4.94 4.93
3.96 3.81
2.81 2.80 2.80 2.79 2.79 2.78 2.36 2.35 2.34 2.34 2.33 2.32 2.31
2.04 2.04 2.02 2.02 2.01 1.99
1.44
1.00 2.04 2.05 2.08 1.10 2.06 2.08 9.35
9.0 8.5 8.0 7.5 7.0 6.5 6.0 5.5 5.0 4.5 4.0 3.5 3.0 2.5 2.0 1.5 1.0 0.5 0.0
ppm

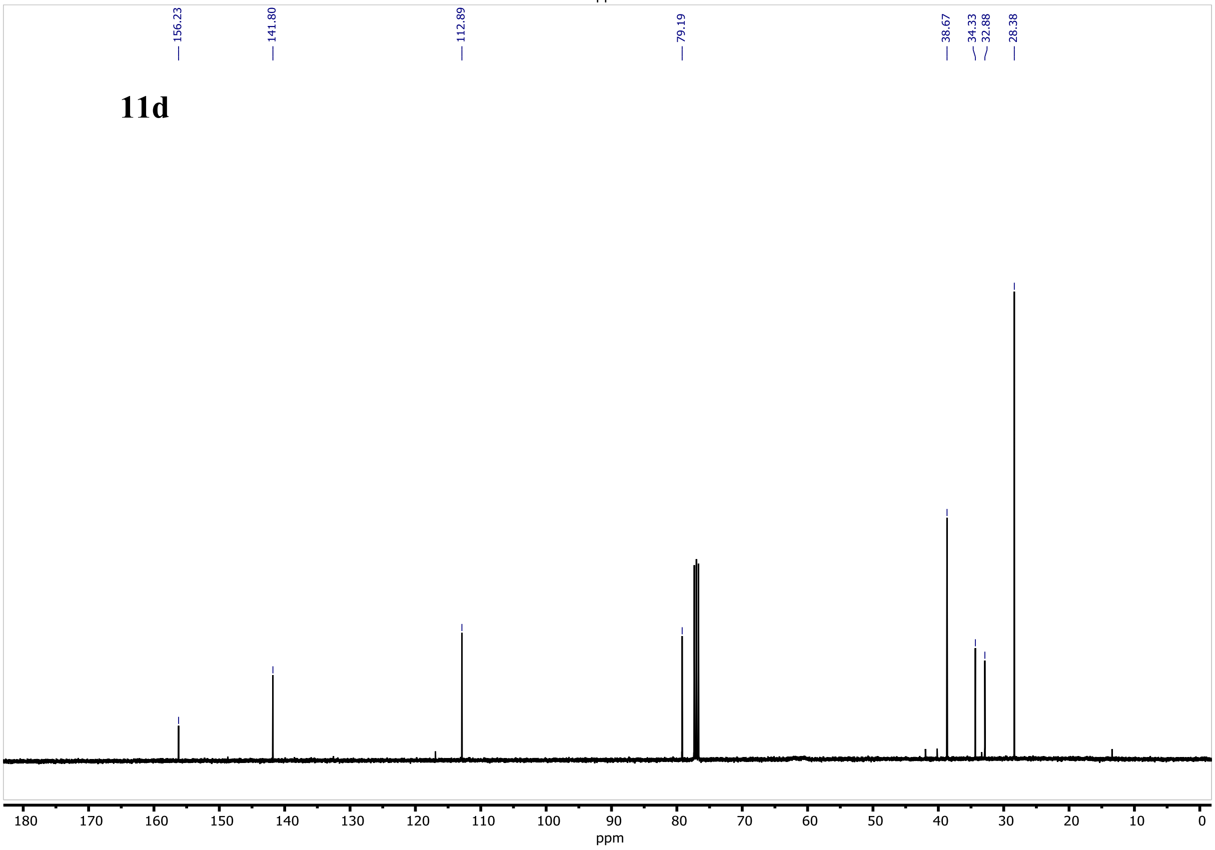
11d
156.23 141.80 112.89 79.19 38.67 34.33 32.88 28.38
180 170 160 150 140 130 120 110 100 90 80 70 60 50 40 30 20 10 0
ppm

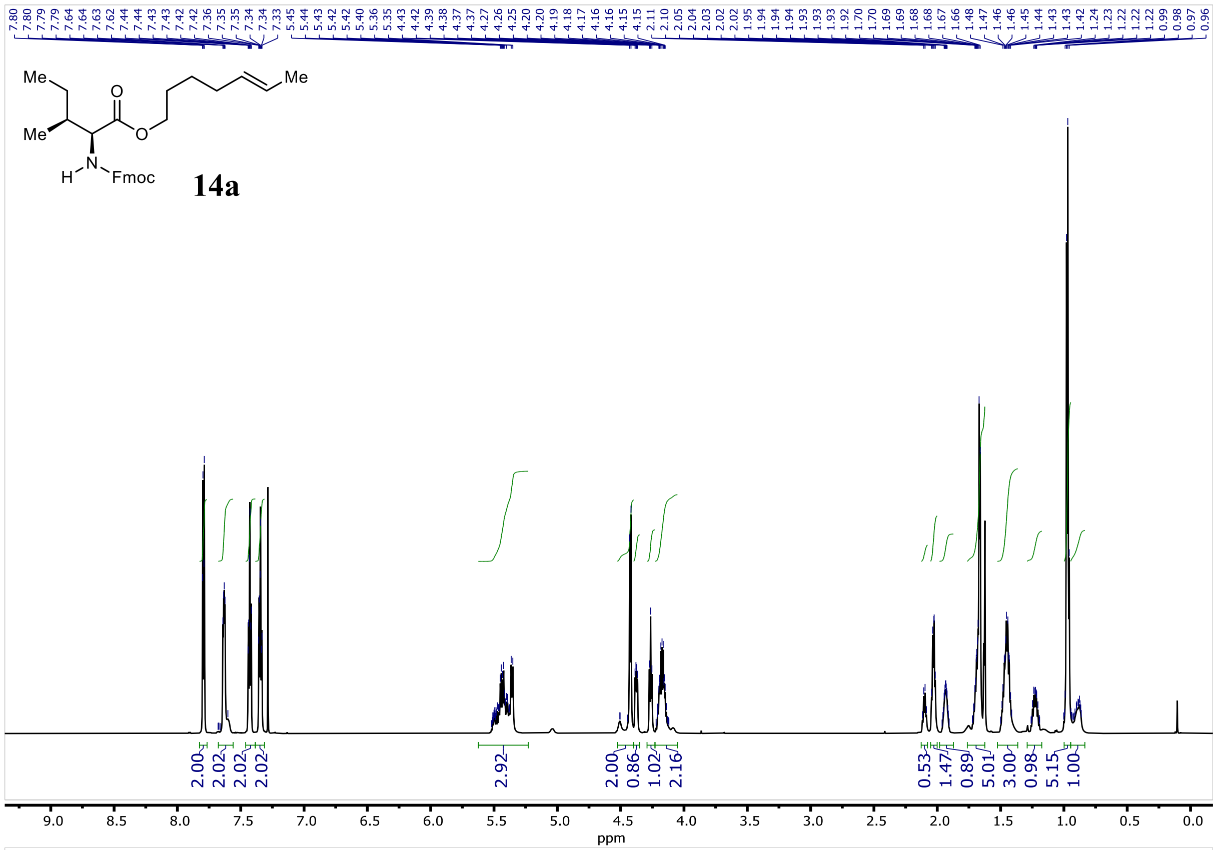

14a

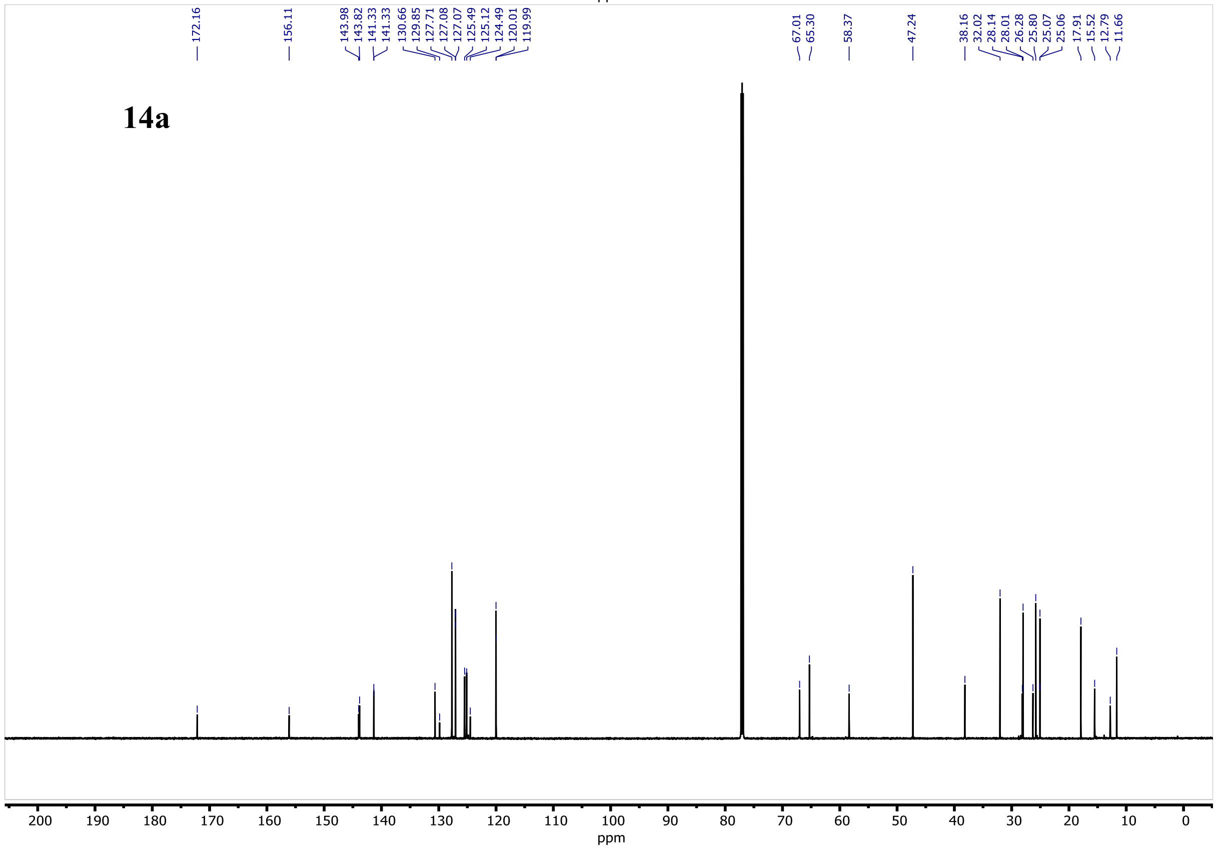

14a

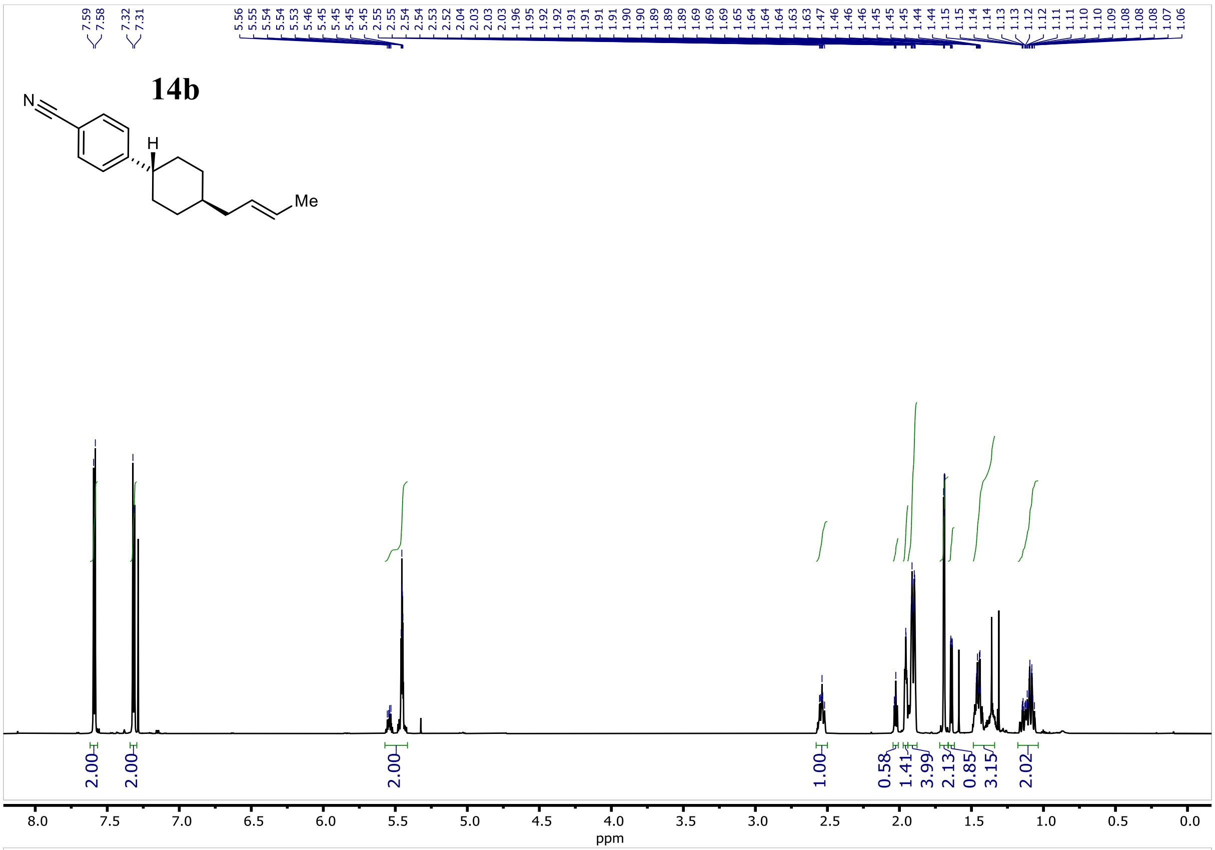
14b
N
H
Me
ppm

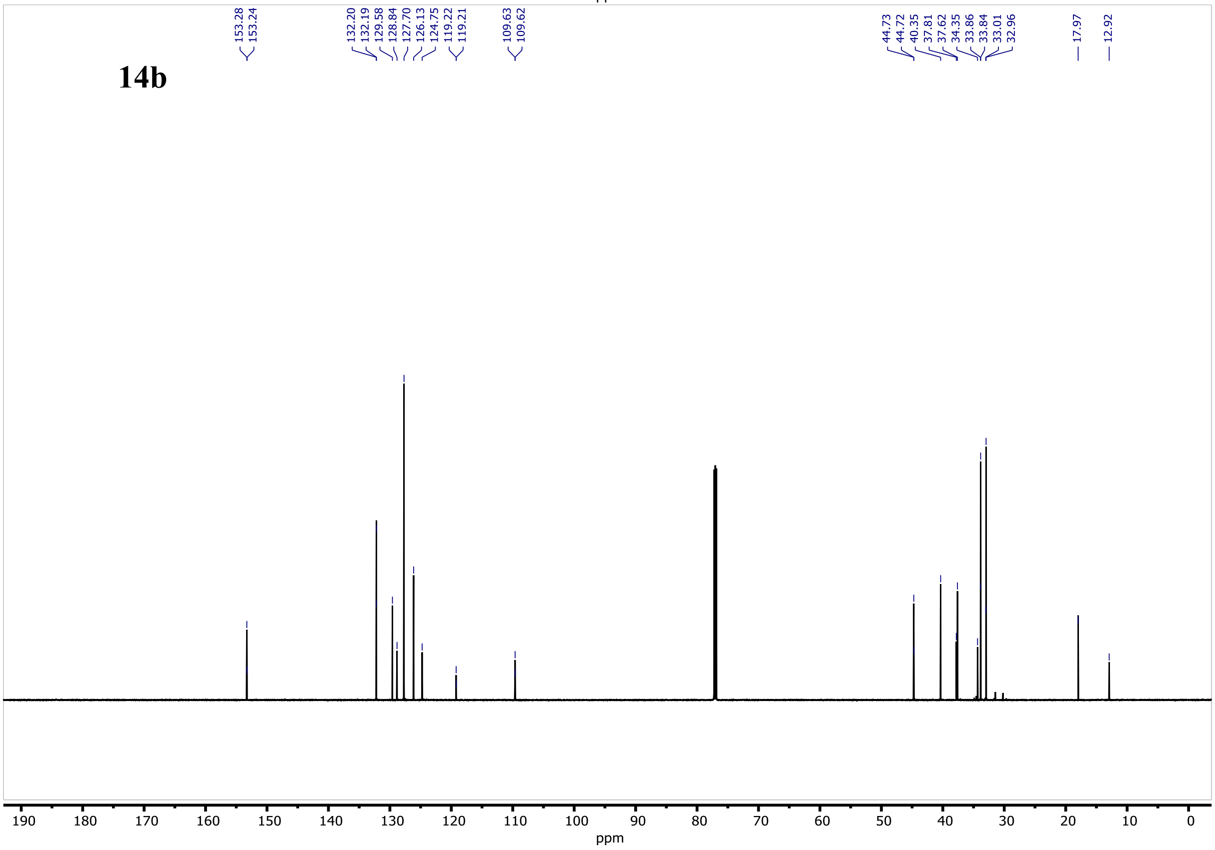
14b
ppm

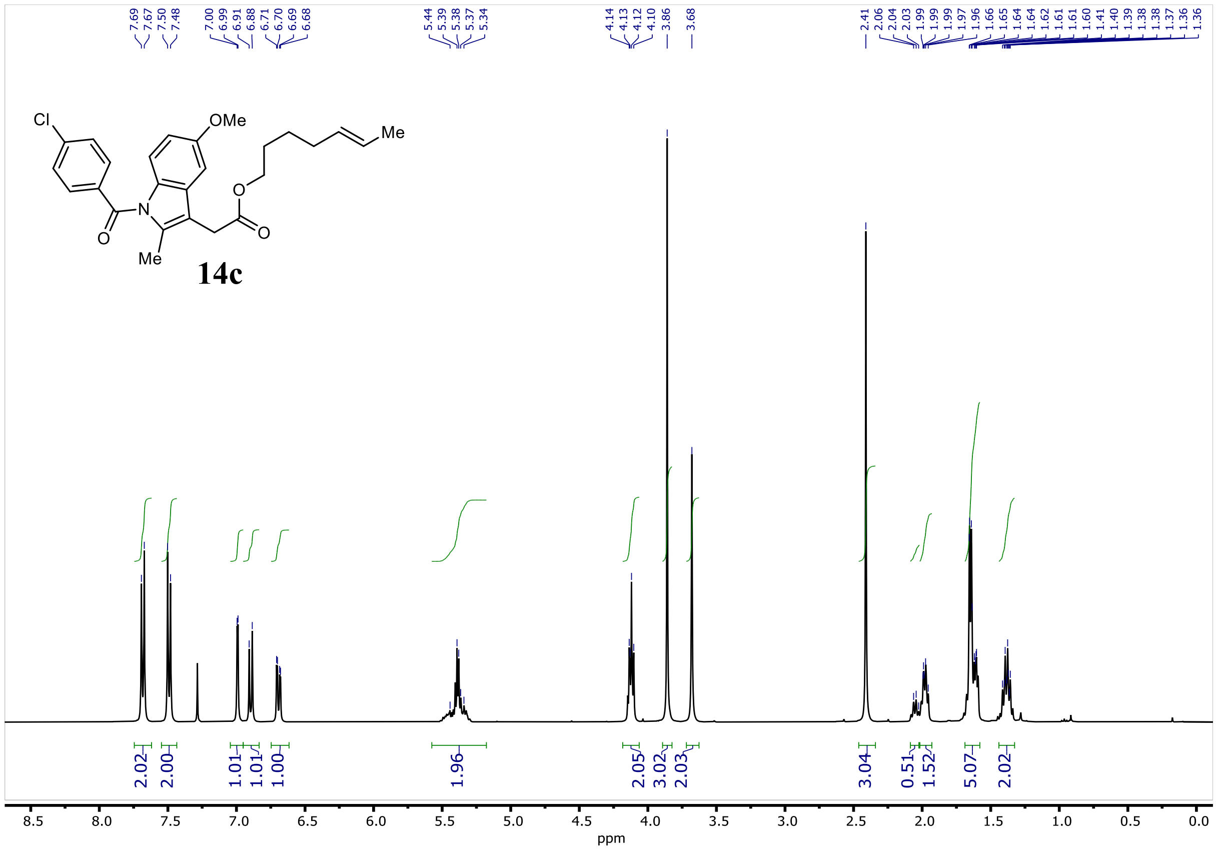
7.69 7.67 7.50 7.48 7.00 6.99 6.91 6.88 6.71 6.70 6.69 6.68 5.44 5.39 5.38 5.37 5.34 4.14 4.13 4.12 4.10 3.86 3.68 2.41 2.06 2.04 2.03 1.99 1.99 1.99 1.97 1.96 1.66 1.65 1.64 1.64 1.62 1.61 1.61 1.60 1.41 1.40 1.39 1.38 1.38 1.37 1.36 1.36
Cl
OMe
Me
N
O
O
O
Me
14c
2.02 2.00 1.01 1.01 1.00 1.96 2.05 3.02 2.03 3.04 0.51 1.52 5.07 2.02
8.5 8.0 7.5 7.0 6.5 6.0 5.5 5.0 4.5 4.0 3.5 3.0 2.5 2.0 1.5 1.0 0.5 0.0
ppm

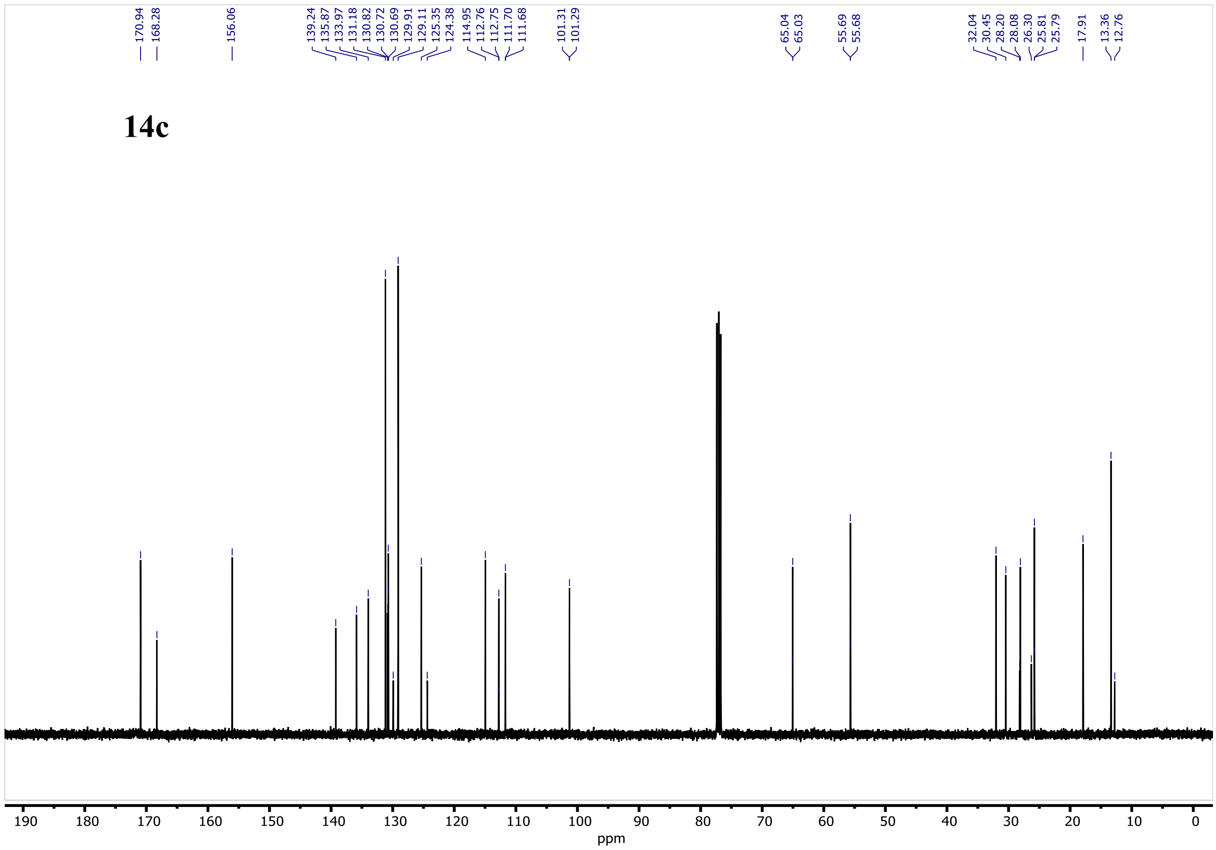
170.94 168.28 156.06 139.24 135.87 133.97 131.18 130.82 130.72 130.69 129.91 129.11 125.35 124.38 114.95 112.76 112.75 111.70 111.68 101.31 101.29 65.04 65.03 55.69 55.68 32.04 30.45 28.20 28.08 26.30 25.81 25.79 17.91 13.36 12.76
14c
190 180 170 160 150 140 130 120 110 100 90 80 70 60 50 40 30 20 10 0
ppm

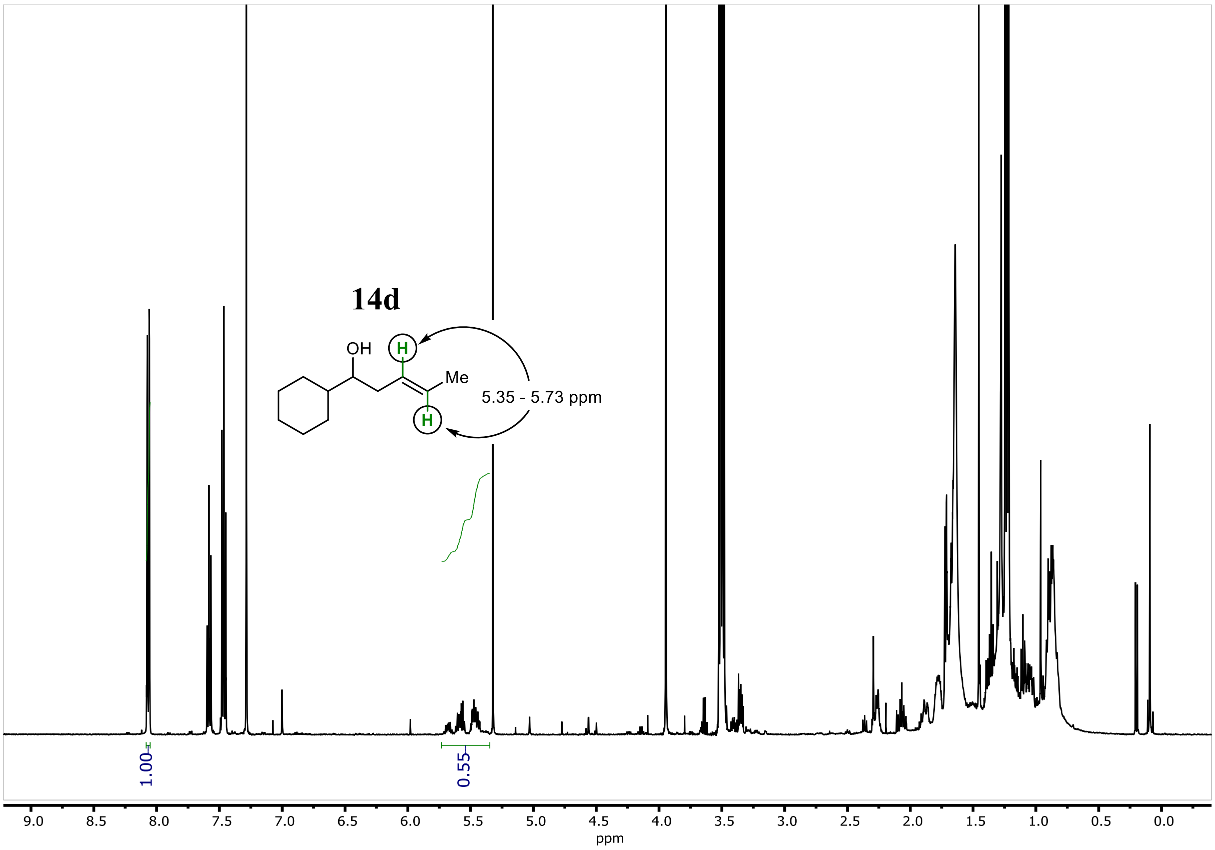
14d
OH
H
Me
5.35 - 5.73 ppm
1.00
0.55
9.0 8.5 8.0 7.5 7.0 6.5 6.0 5.5 5.0 4.5 4.0 3.5 3.0 2.5 2.0 1.5 1.0 0.5 0.0
ppm

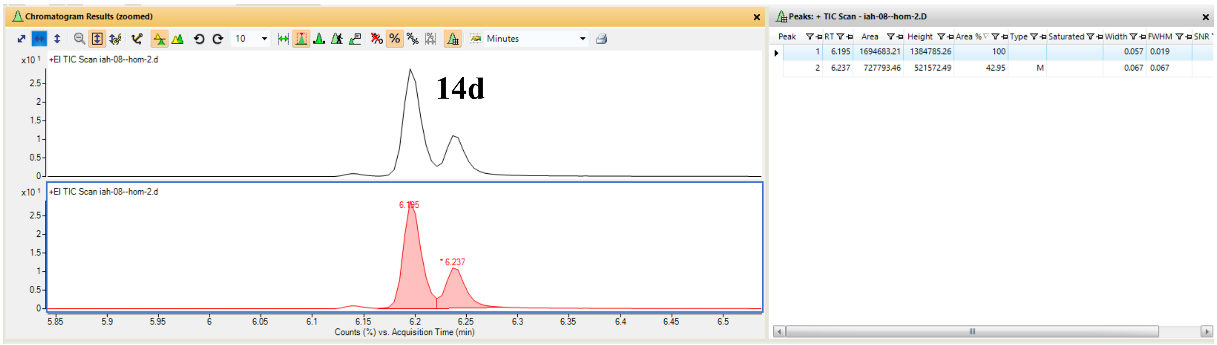
Chromatogram Results (zoomed)
+EI TIC Scan iah-08--hom-2.d
14d
6.195
6.237
Counts (%) vs. Acquisition Time (min)
Peaks: + TIC Scan - iah-08--hom-2.D
Peak RT Area Height Area % Type Saturated Width FWHM SNR
1 6.195 1694683.21 1384785.26 100 0.057 0.019
2 6.237 727793.46 521572.49 42.95 M 0.067 0.067

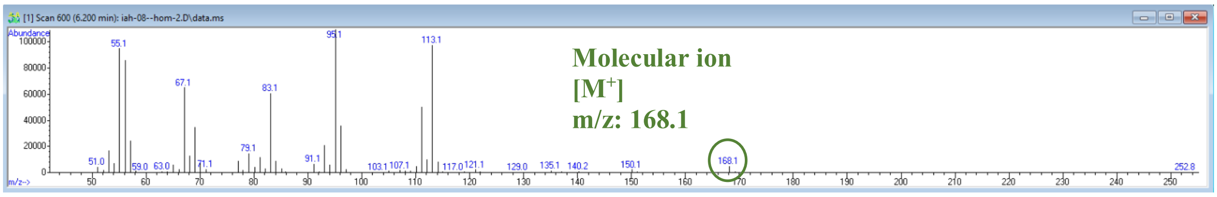
[1] Scan 600 (6.200 min): iah-08--hom-2.D\data.ms
Abundance
55.1
95.1
113.1
67.1
83.1
79.1
51.0
59.0
63.0
71.1
91.1
103.1
107.1
117.0
121.1
129.0
135.1
140.2
150.1
168.1
252.8
m/z-->
Molecular ion
[M+]
m/z: 168.1

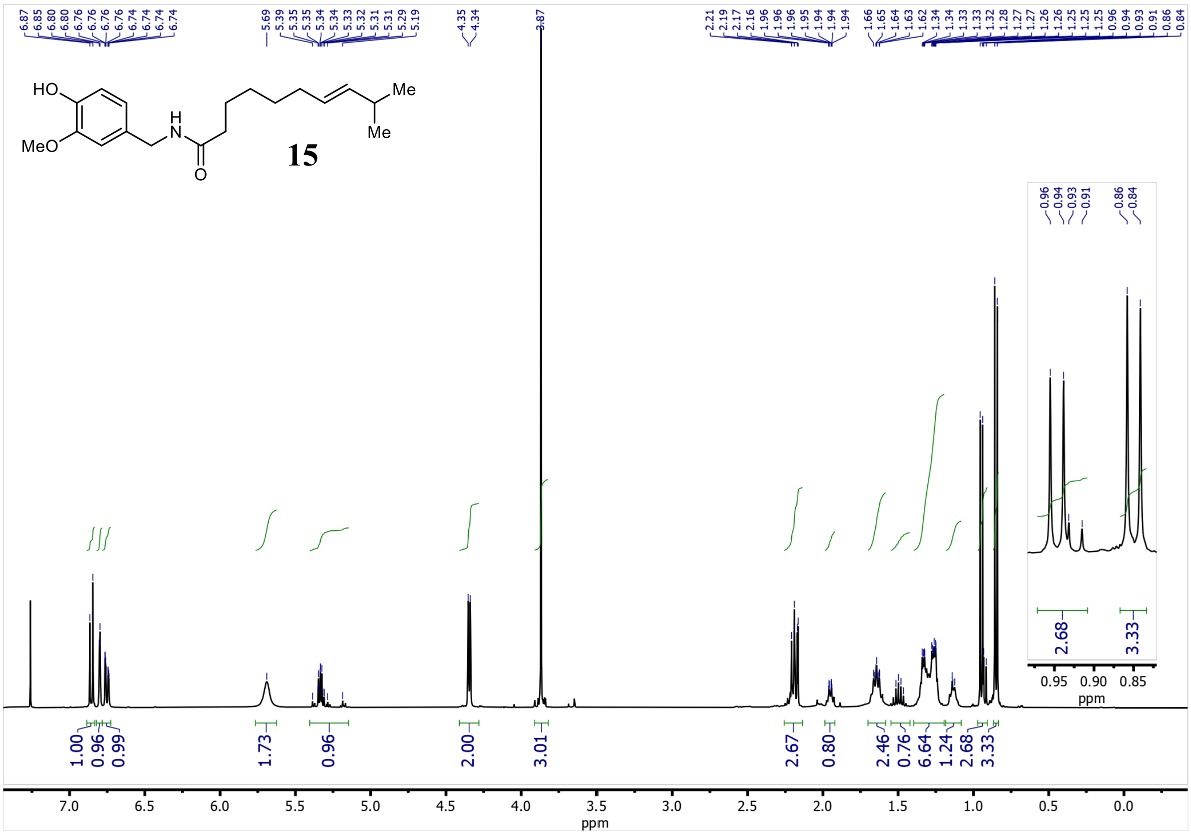

6.87 6.85 6.80 6.80 6.76 6.76 6.76 6.74 6.74 6.74
5.69 5.39 5.35 5.35 5.34 5.34 5.33 5.32 5.31 5.29 5.19
4.35 4.34
3.87
2.21 2.19 2.17 2.16 1.96 1.96 1.95 1.94 1.94 1.94
1.66 1.65 1.64 1.63 1.62 1.34 1.34 1.33 1.33 1.32 1.28 1.27 1.27 1.26 1.25 1.25 1.25 0.96 0.94 0.93 0.91 0.86 0.84
HO
MeO
H
N
O
Me
Me
15
0.96 0.94 0.93 0.91 0.86 0.84
2.68
3.33
0.95 0.90 0.85
ppm
1.00 0.96 0.99 1.73 0.96 2.00 3.01 2.67 0.80 2.46 0.76 6.64 1.24 2.68 3.33
7.0 6.5 6.0 5.5 5.0 4.5 4.0 3.5 3.0 2.5 2.0 1.5 1.0 0.5 0.0
ppm

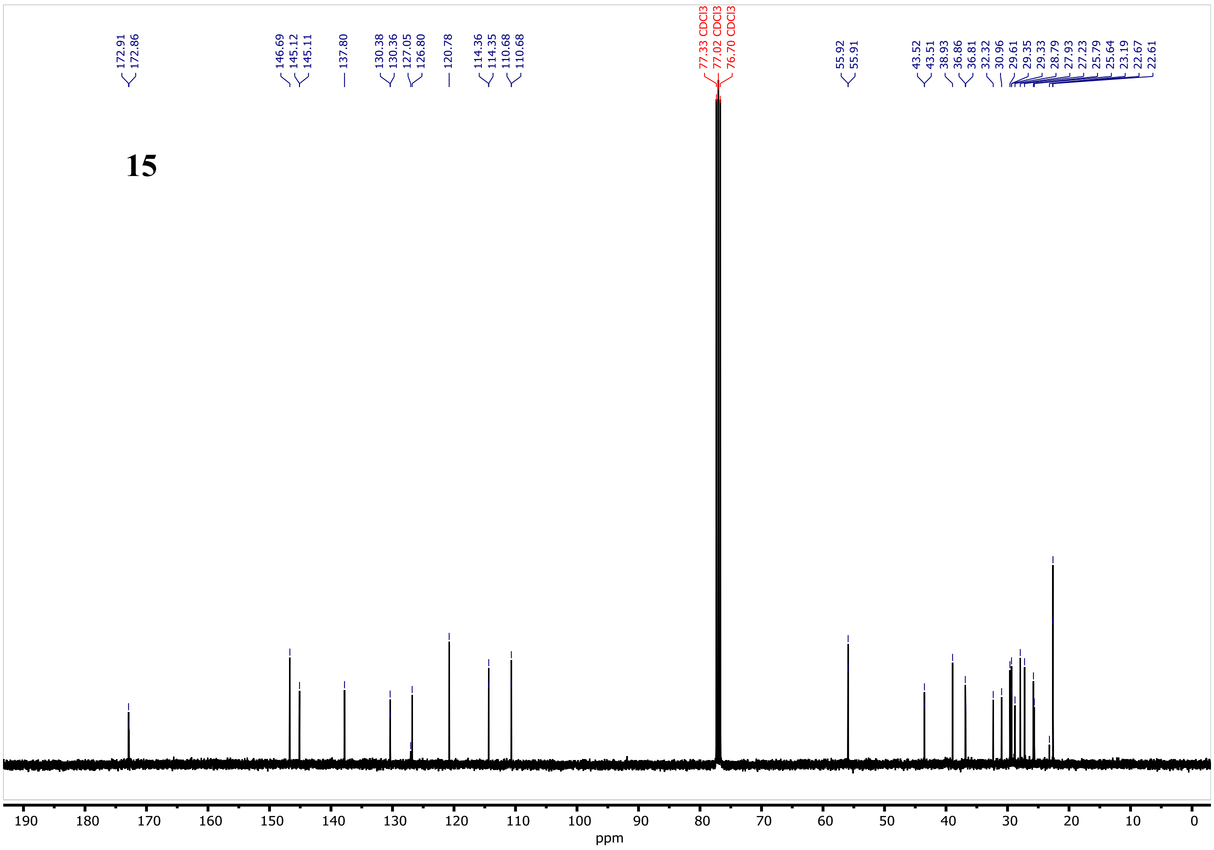

172.91 172.86
146.69 145.12 145.11
137.80
130.38 130.36 127.05 126.80
120.78
114.36 114.35 110.68 110.68
77.33 CDCl3 77.02 CDCl3 76.70 CDCl3
55.92 55.91
43.52 43.51 38.93 36.86 36.81 32.32 30.96 29.61 29.35 29.33 28.79 27.93 27.23 25.79 25.64 23.19 22.67 22.61
15
190 180 170 160 150 140 130 120 110 100 90 80 70 60 50 40 30 20 10 0
ppm

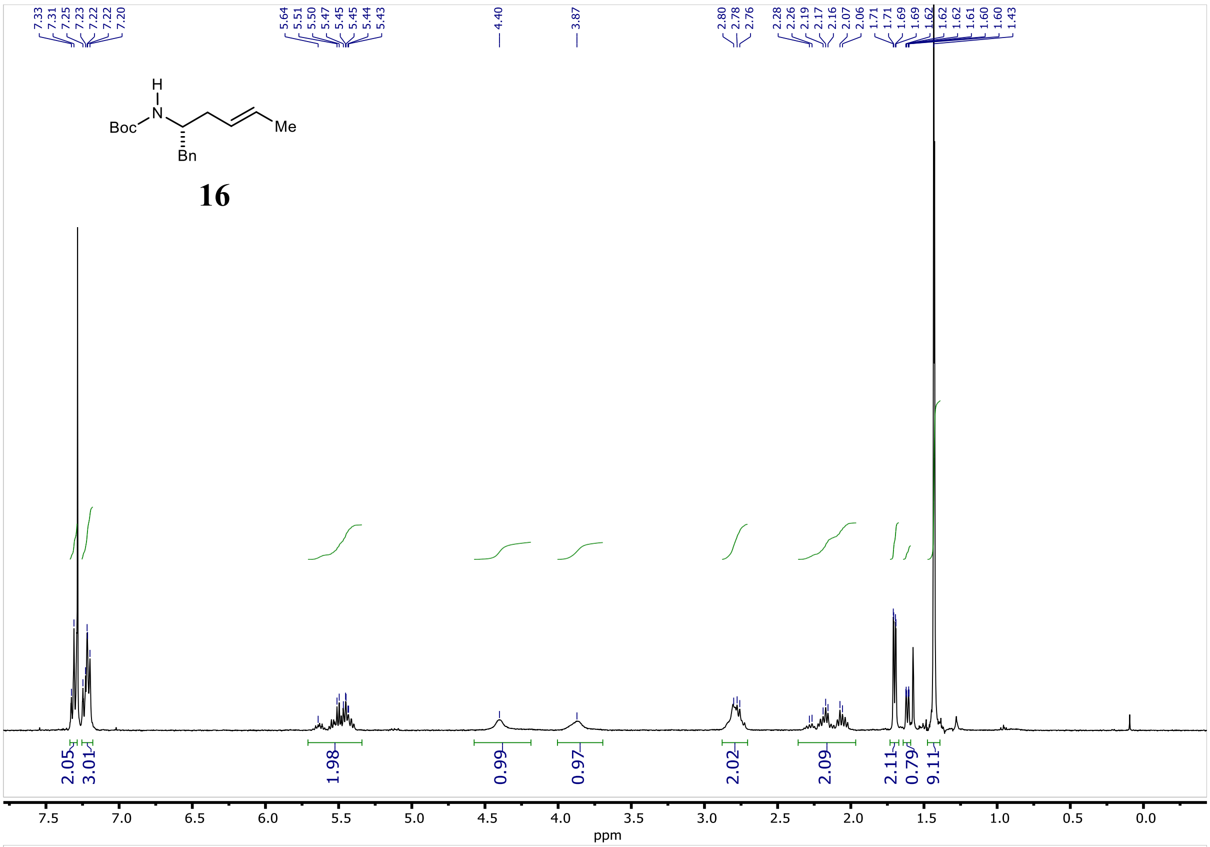
7.33
7.31
7.25
7.23
7.22
7.22
7.20
5.64
5.51
5.50
5.47
5.45
5.45
5.44
5.43
4.40
3.87
2.80
2.78
2.76
2.28
2.26
2.19
2.17
2.16
2.07
2.06
1.71
1.71
1.69
1.69
1.62
1.62
1.62
1.61
1.60
1.60
1.43
H
N
Boc
Bn
Me
16
2.05
3.01
1.98
0.99
0.97
2.02
2.09
2.11
0.79
9.11
ppm

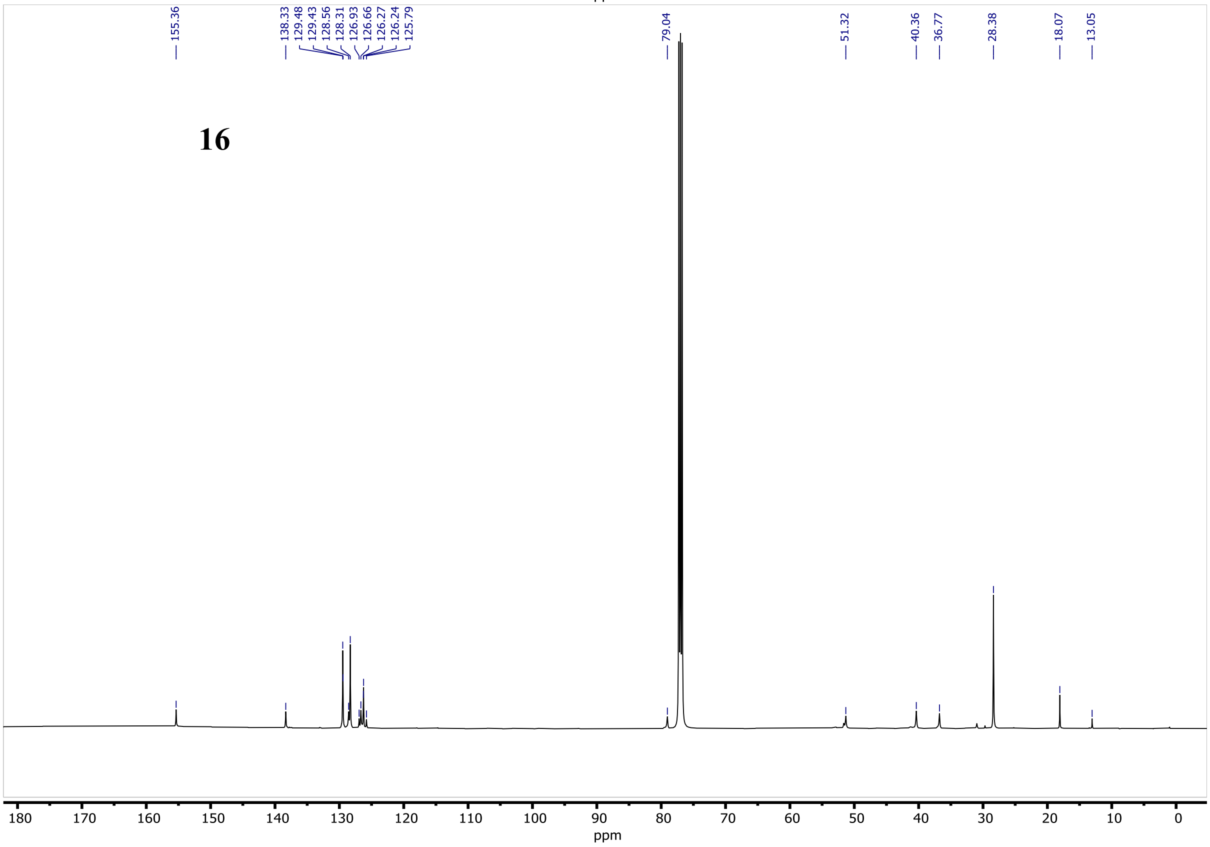
155.36
138.33
129.48
129.43
128.56
128.31
126.93
126.66
126.27
126.24
125.79
79.04
51.32
40.36
36.77
28.38
18.07
13.05
16
ppm

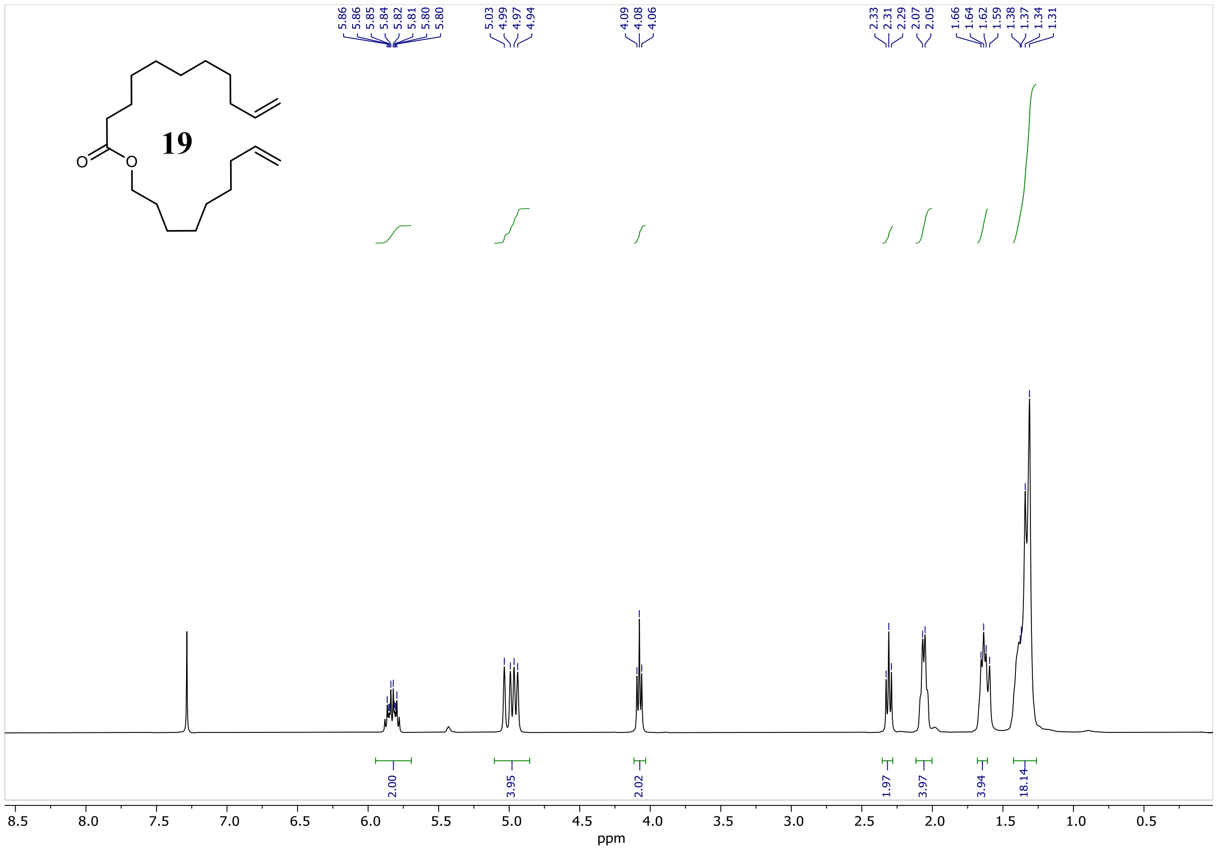
19
ppm

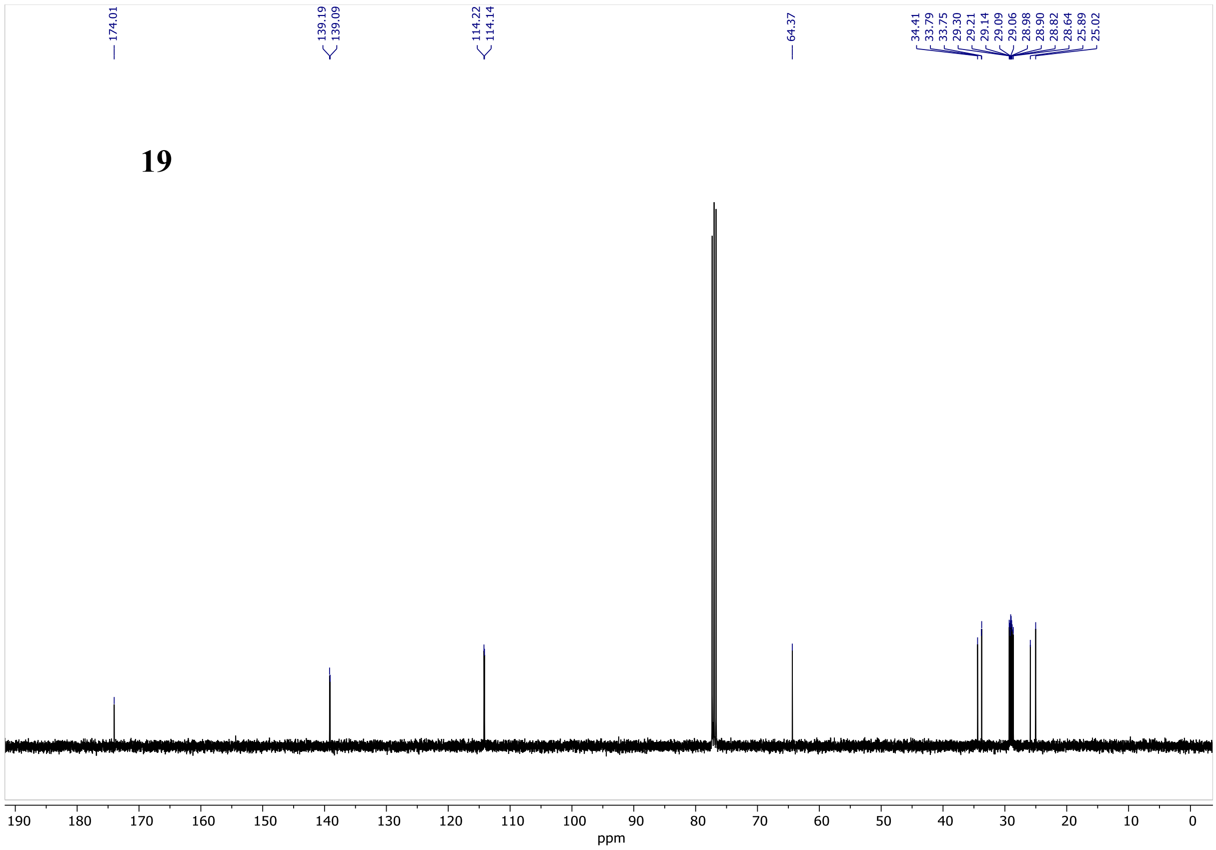
19
ppm

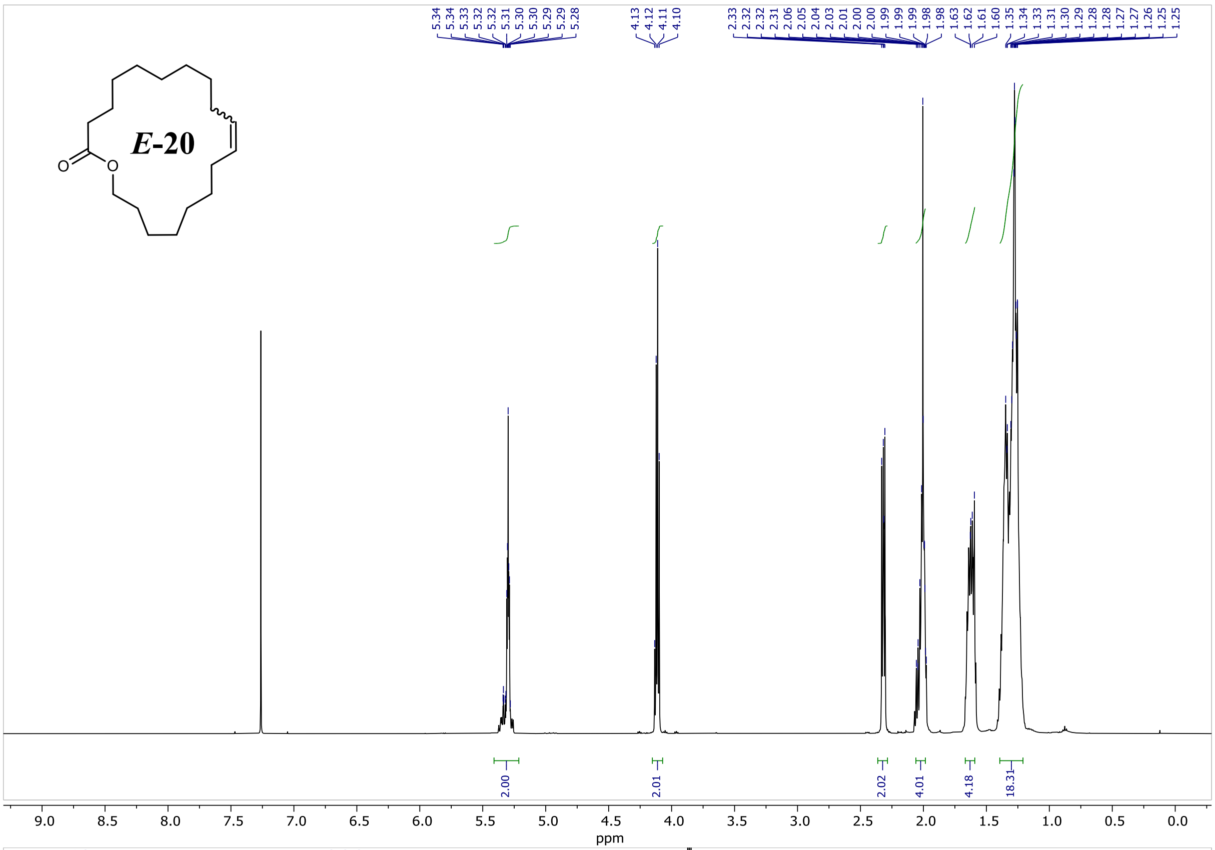
E-20
5.34 5.34 5.33 5.32 5.32 5.31 5.30 5.30 5.29 5.29 5.28
4.13 4.12 4.11 4.10
2.33 2.32 2.32 2.31 2.06 2.05 2.04 2.03 2.01 2.00 2.00 1.99 1.99 1.99 1.98 1.98
1.63 1.62 1.61 1.60 1.35 1.34 1.33 1.31 1.30 1.29 1.28 1.28 1.27 1.27 1.26 1.25 1.25
2.00 2.01 2.02 4.01 4.18 18.31
9.0 8.5 8.0 7.5 7.0 6.5 6.0 5.5 5.0 4.5 4.0 3.5 3.0 2.5 2.0 1.5 1.0 0.5 0.0
ppm

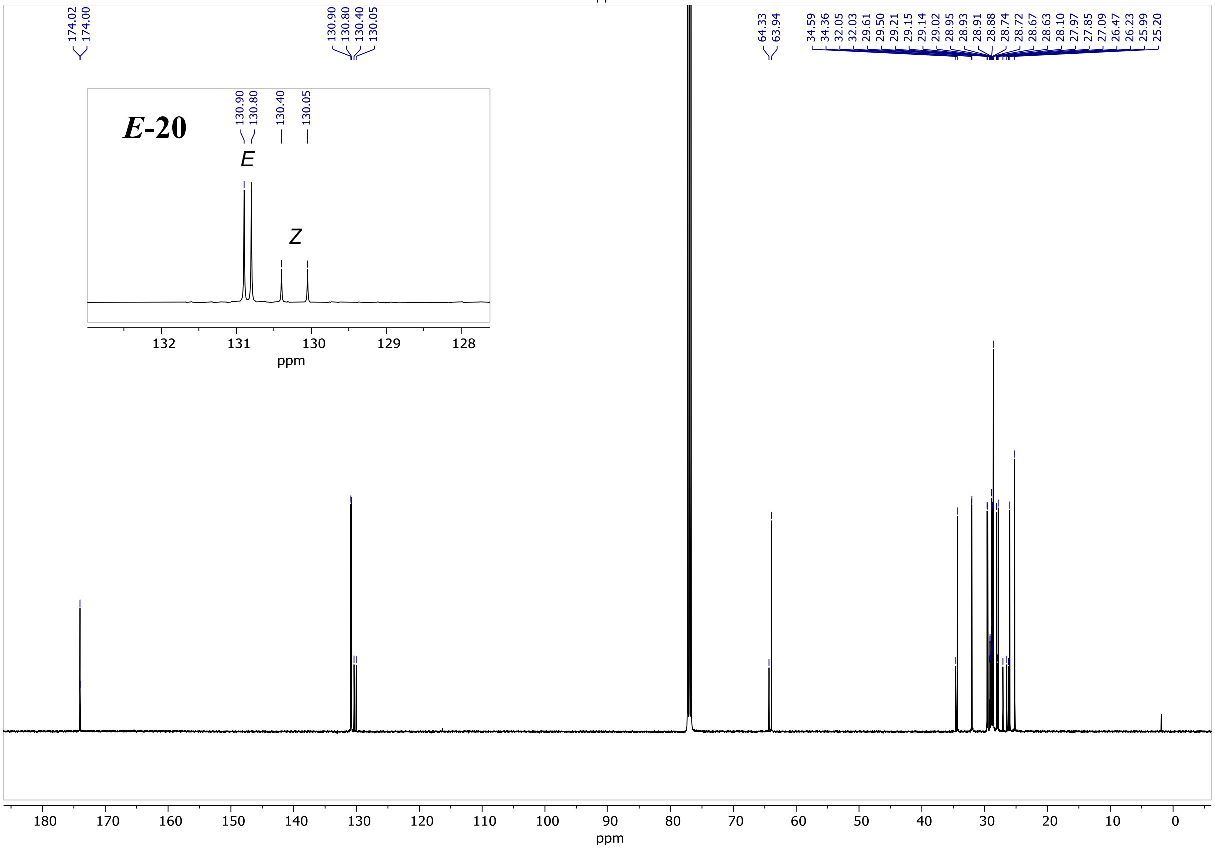
174.02 174.00
130.90 130.80 130.40 130.05
64.33 63.94
34.59 34.36 32.05 32.03 29.61 29.50 29.21 29.15 29.14 29.02 28.95 28.93 28.91 28.88 28.74 28.72 28.67 28.63 28.10 27.97 27.85 27.09 26.47 26.23 25.99 25.20
E-20
130.90 130.80 130.40 130.05
E
Z
132 131 130 129 128
ppm
180 170 160 150 140 130 120 110 100 90 80 70 60 50 40 30 20 10 0
ppm

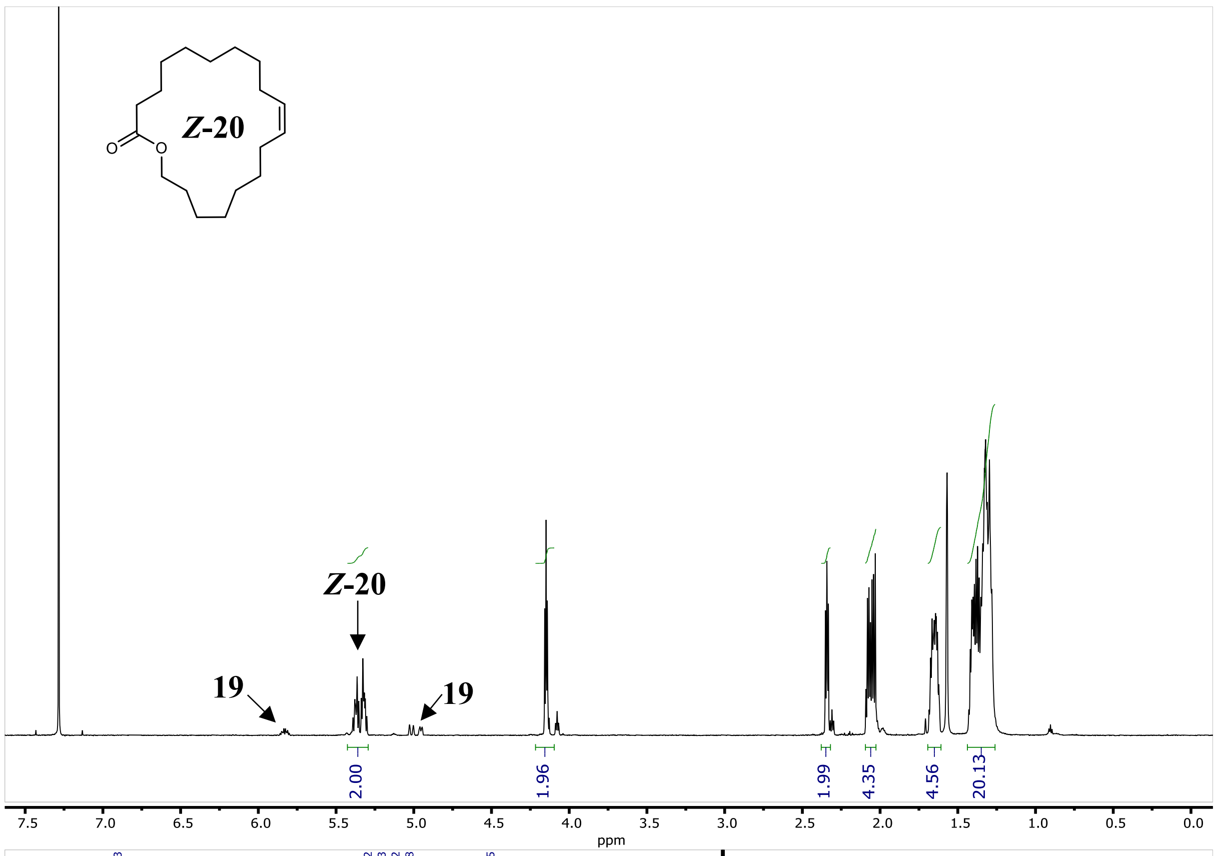

Z-20
Z-20
19
19
2.00
1.96
1.99
4.35
4.56
20.13
ppm

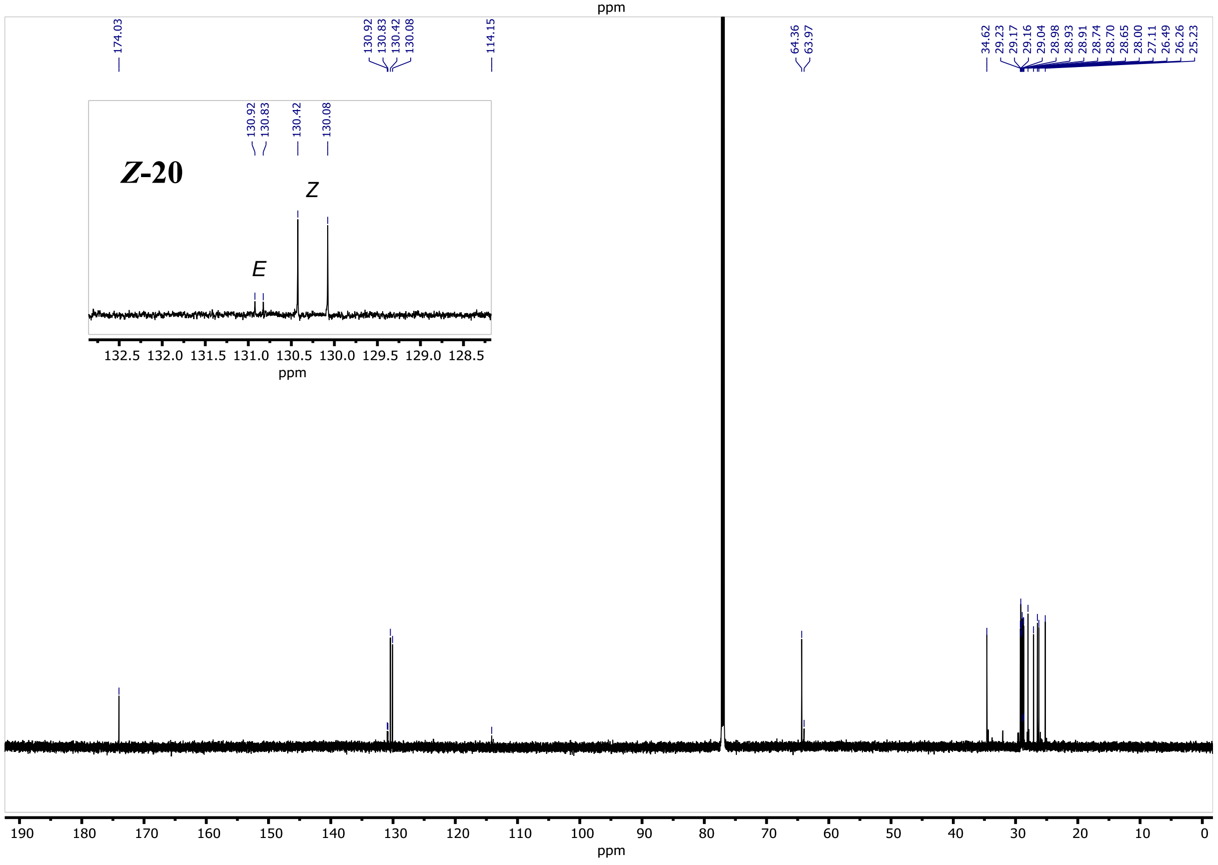

174.03
130.92
130.83
130.42
130.08
114.15
64.36
63.97
34.62
29.23
29.17
29.16
29.04
28.98
28.93
28.91
28.74
28.70
28.65
28.00
27.11
26.49
26.26
25.23
Z-20
Z
E
ppm
ppm

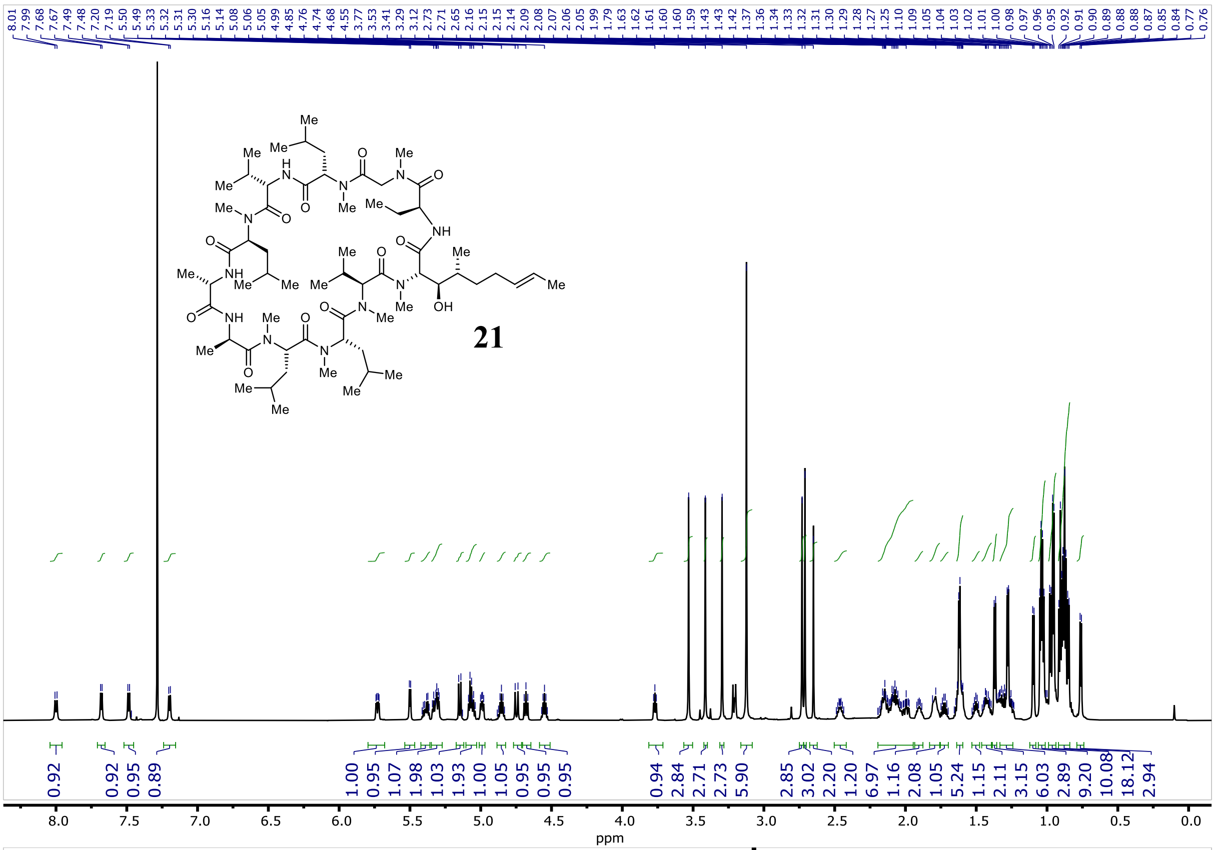

21

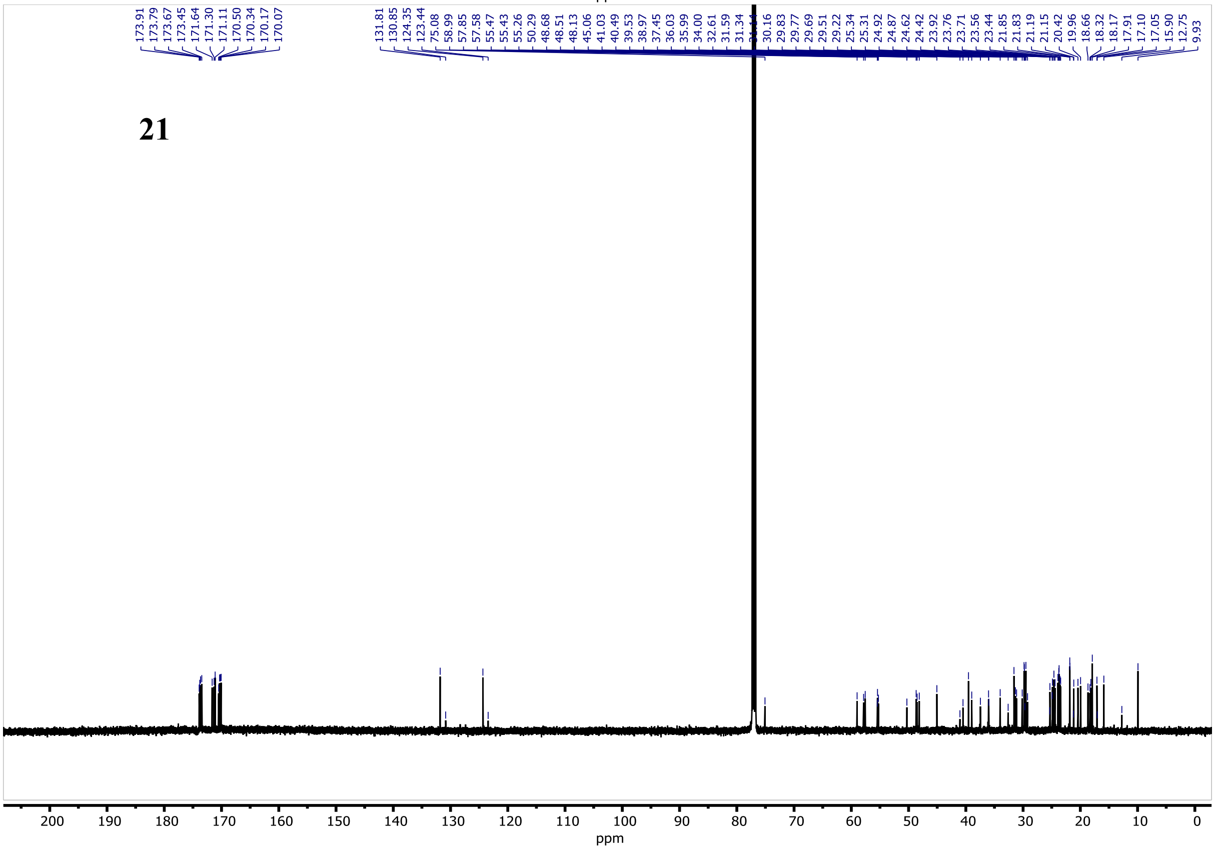

21

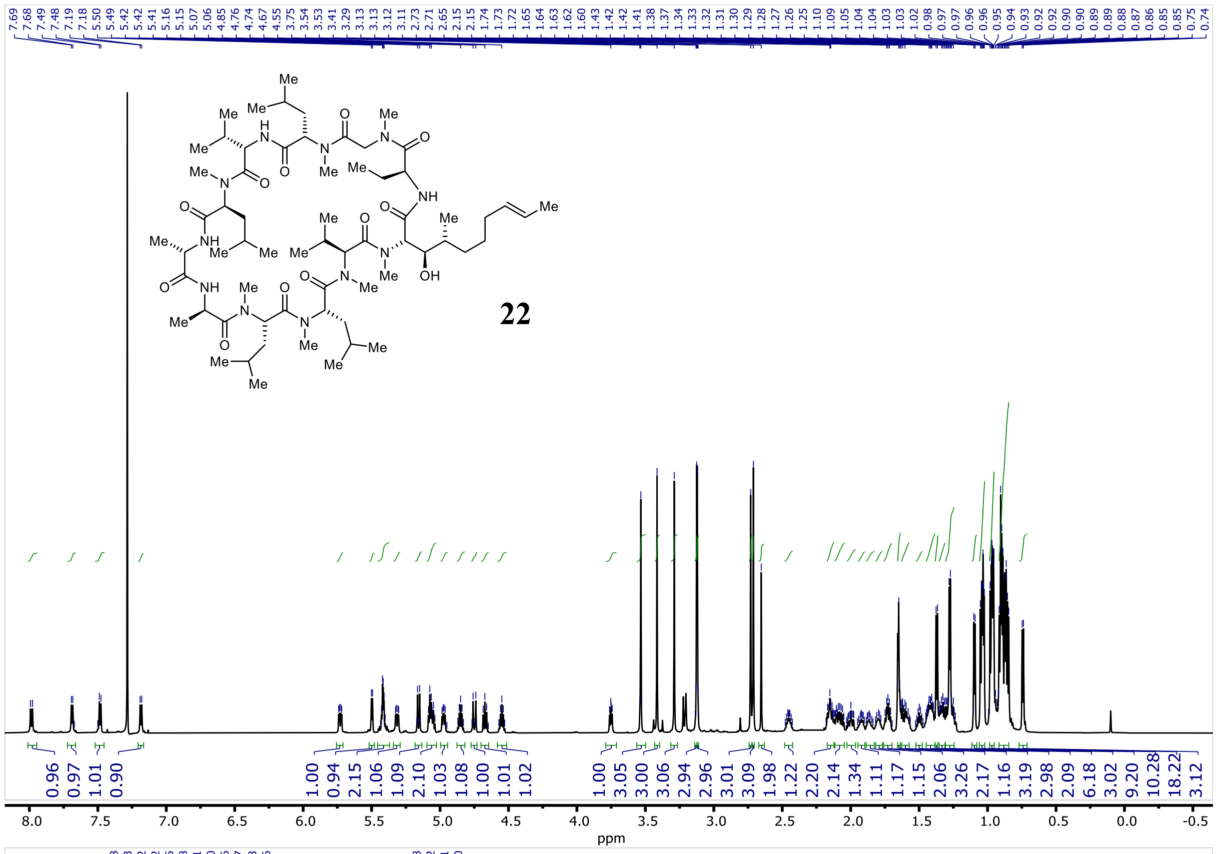

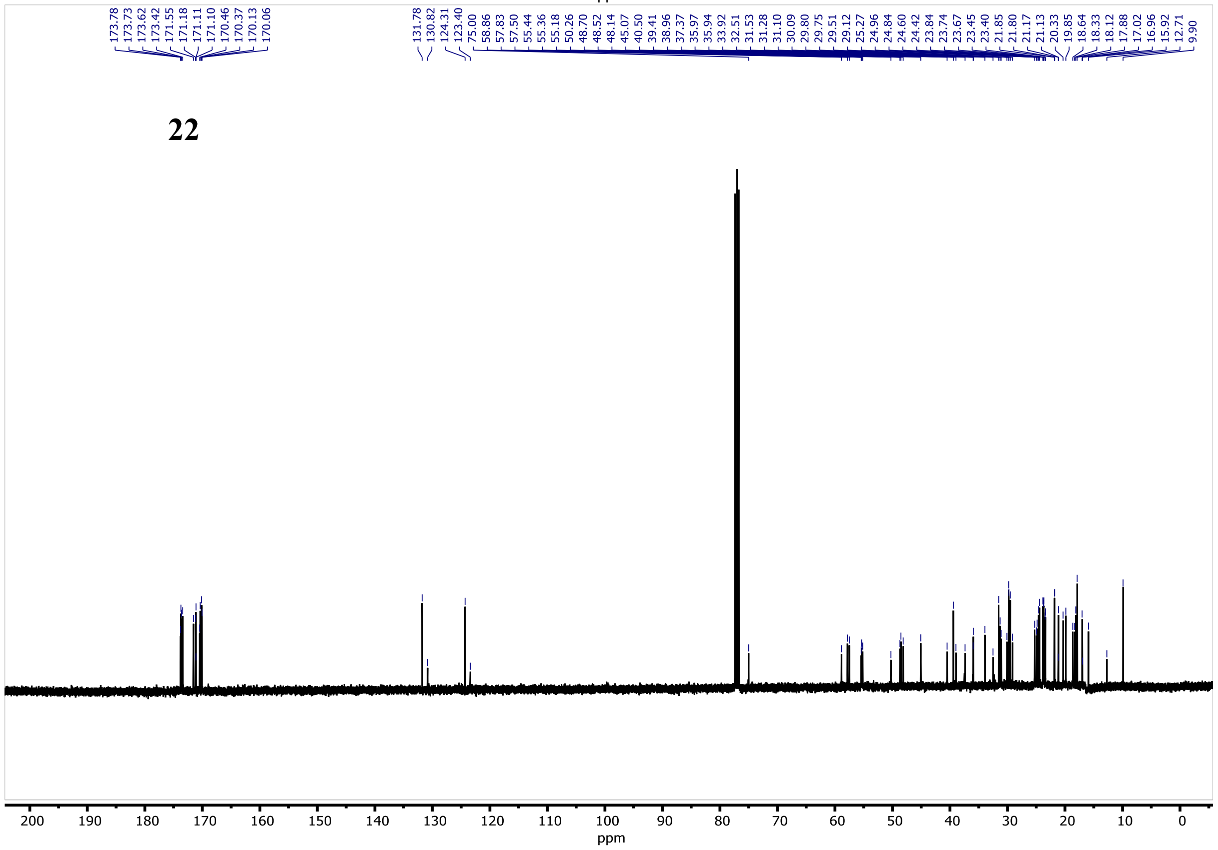

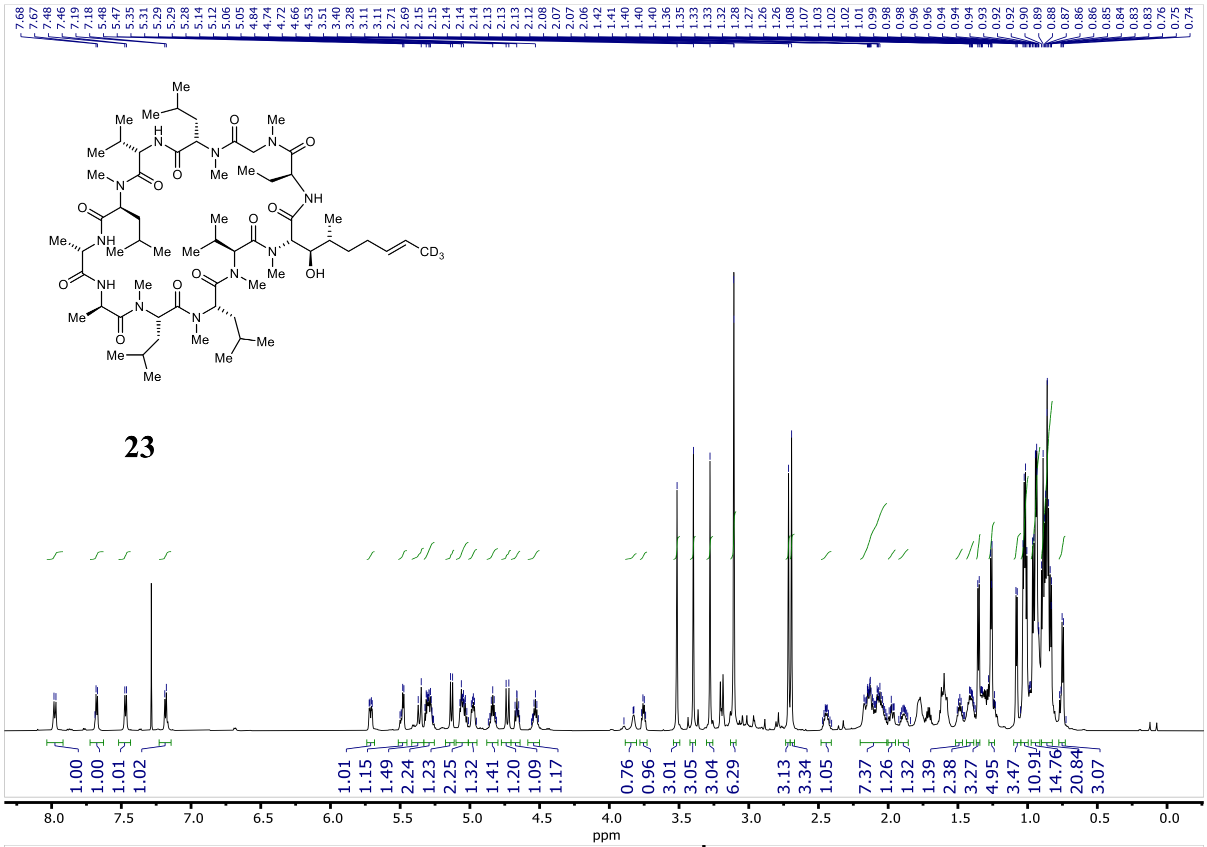

23

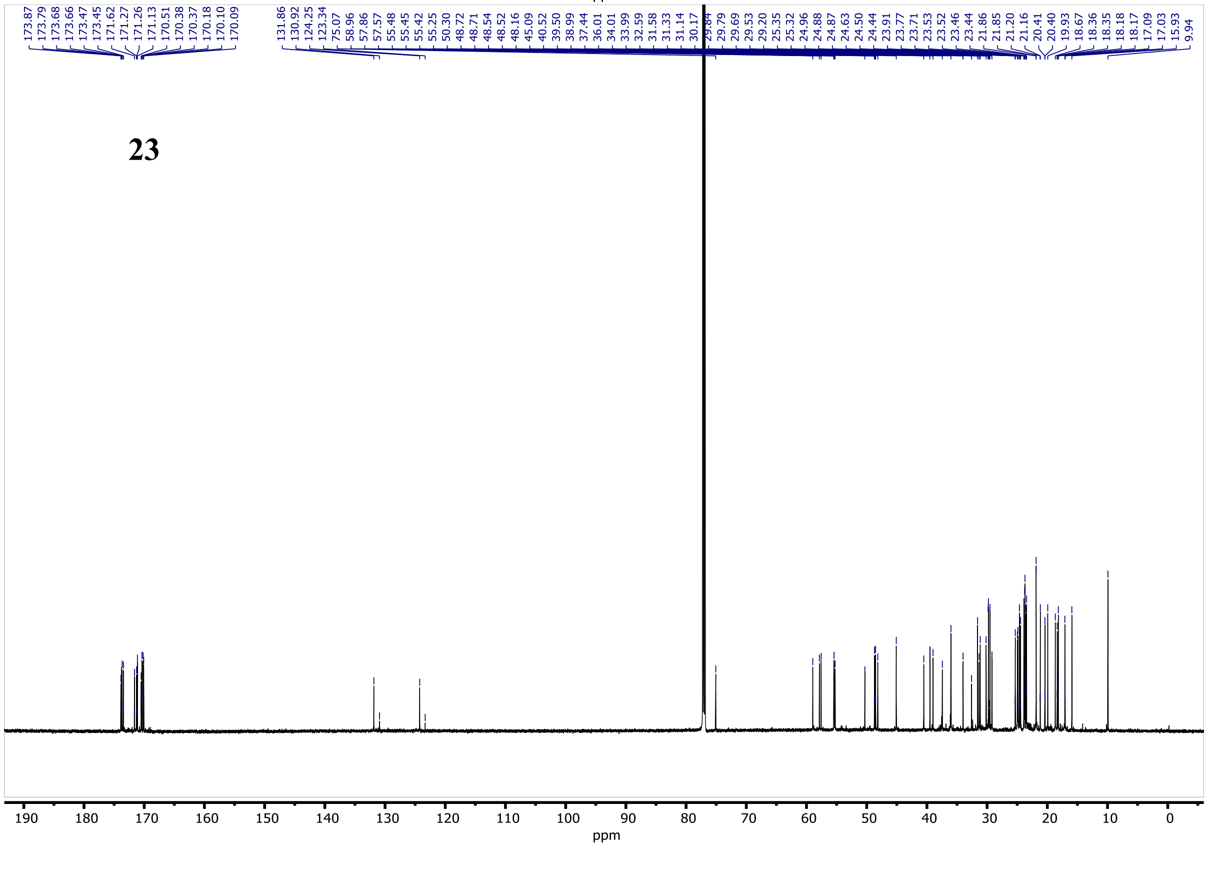

23

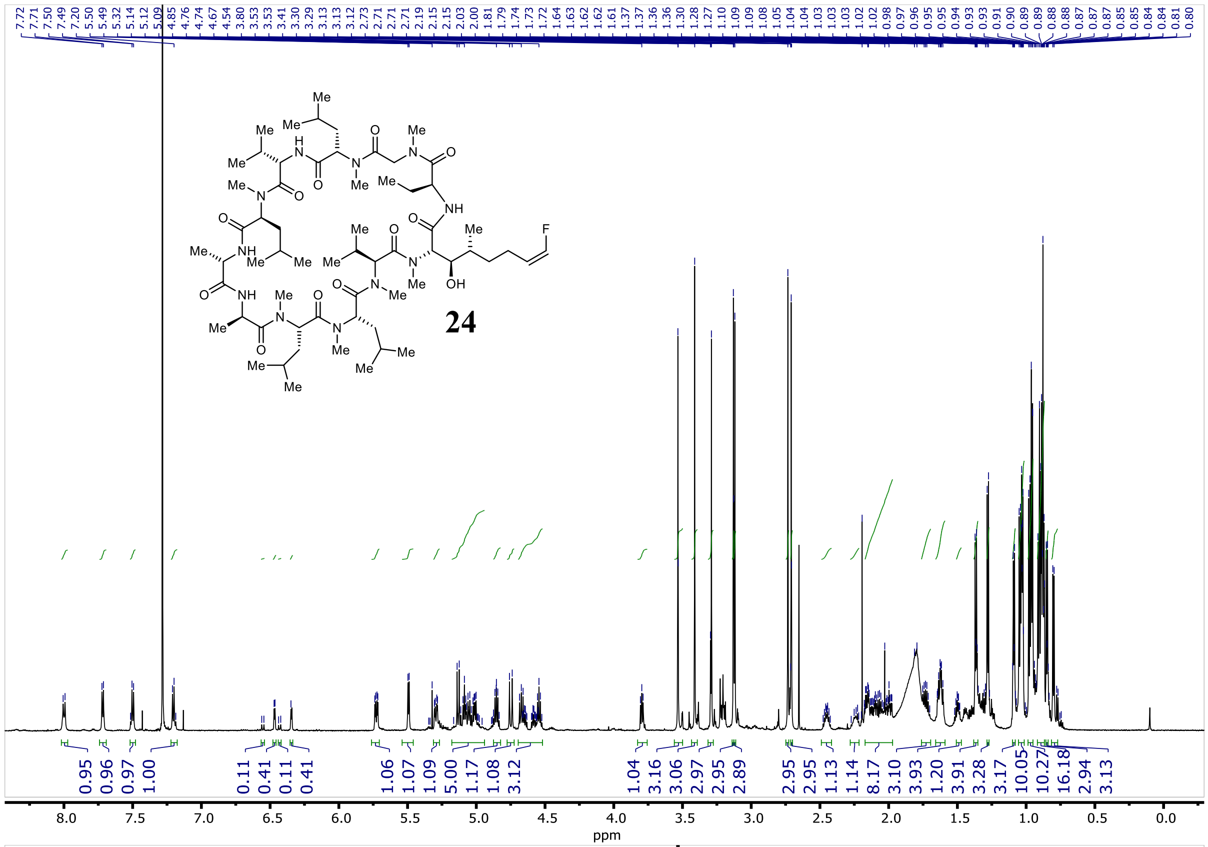
24
ppm

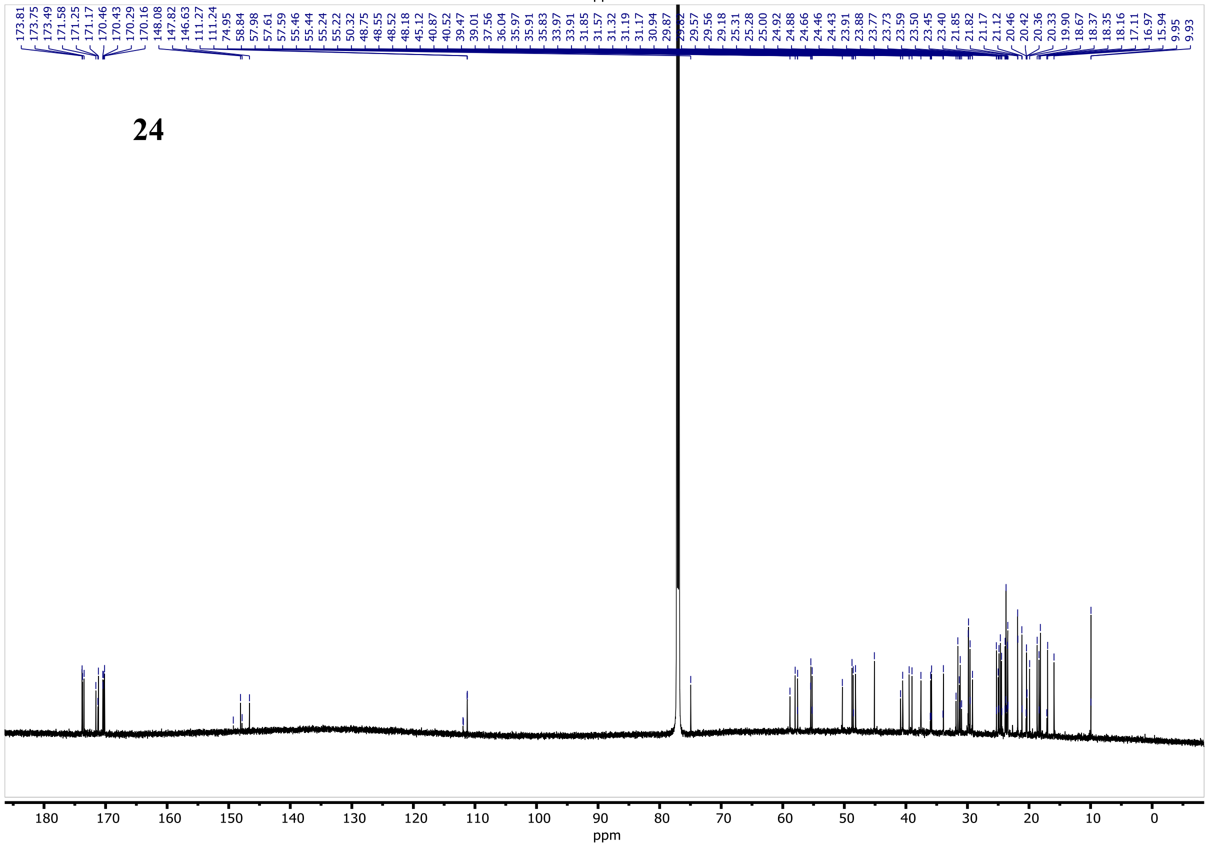
24
ppm

24

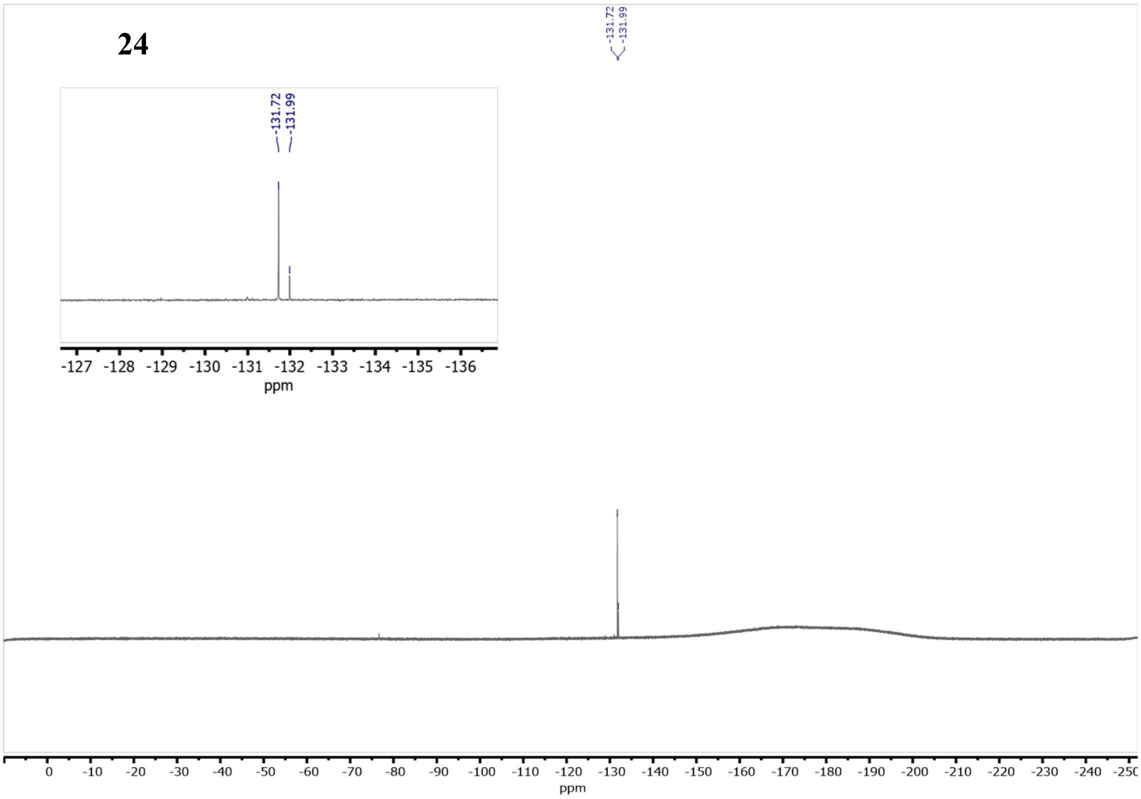
-131.72
-131.99
-127 -128 -129 -130 -131 -132 -133 -134 -135 -136
ppm
0 -10 -20 -30 -40 -50 -60 -70 -80 -90 -100 -110 -120 -130 -140 -150 -160 -170 -180 -190 -200 -210 -220 -230 -240 -250
ppm

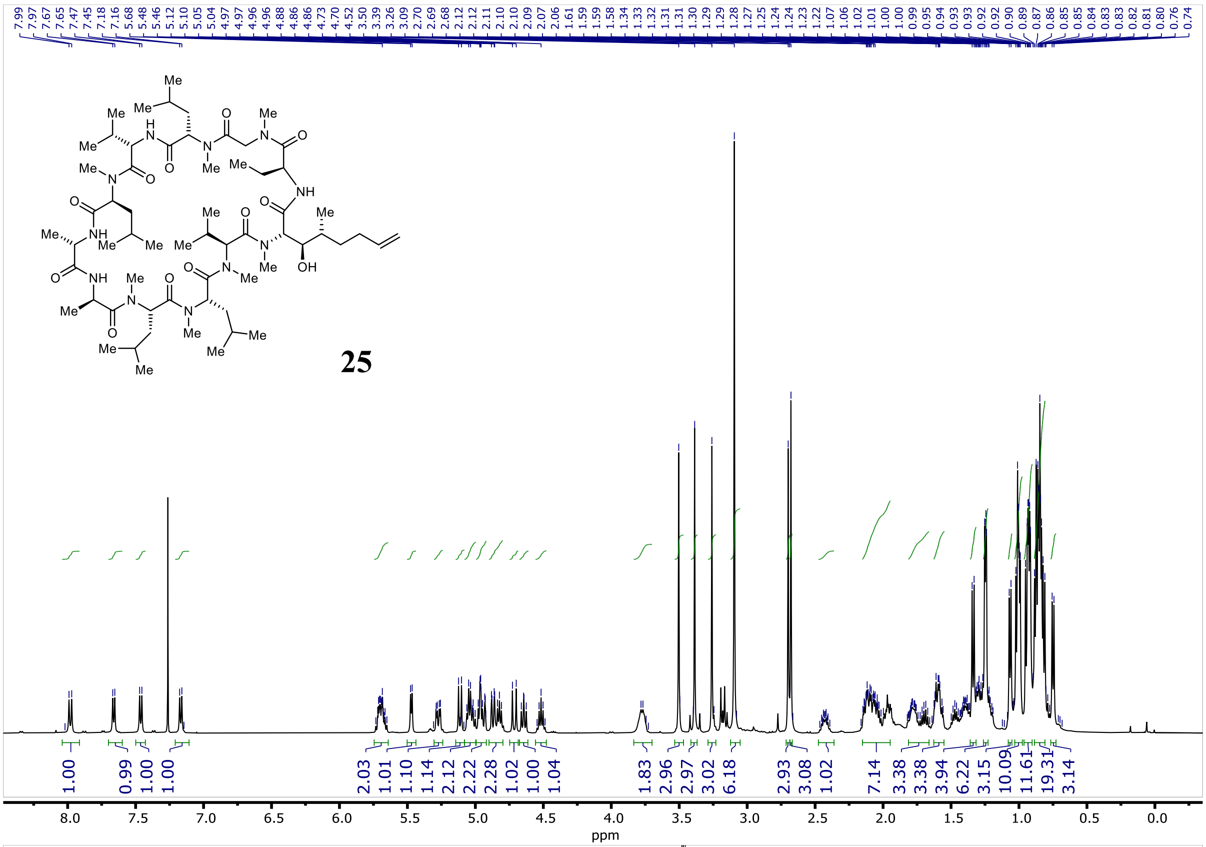

25

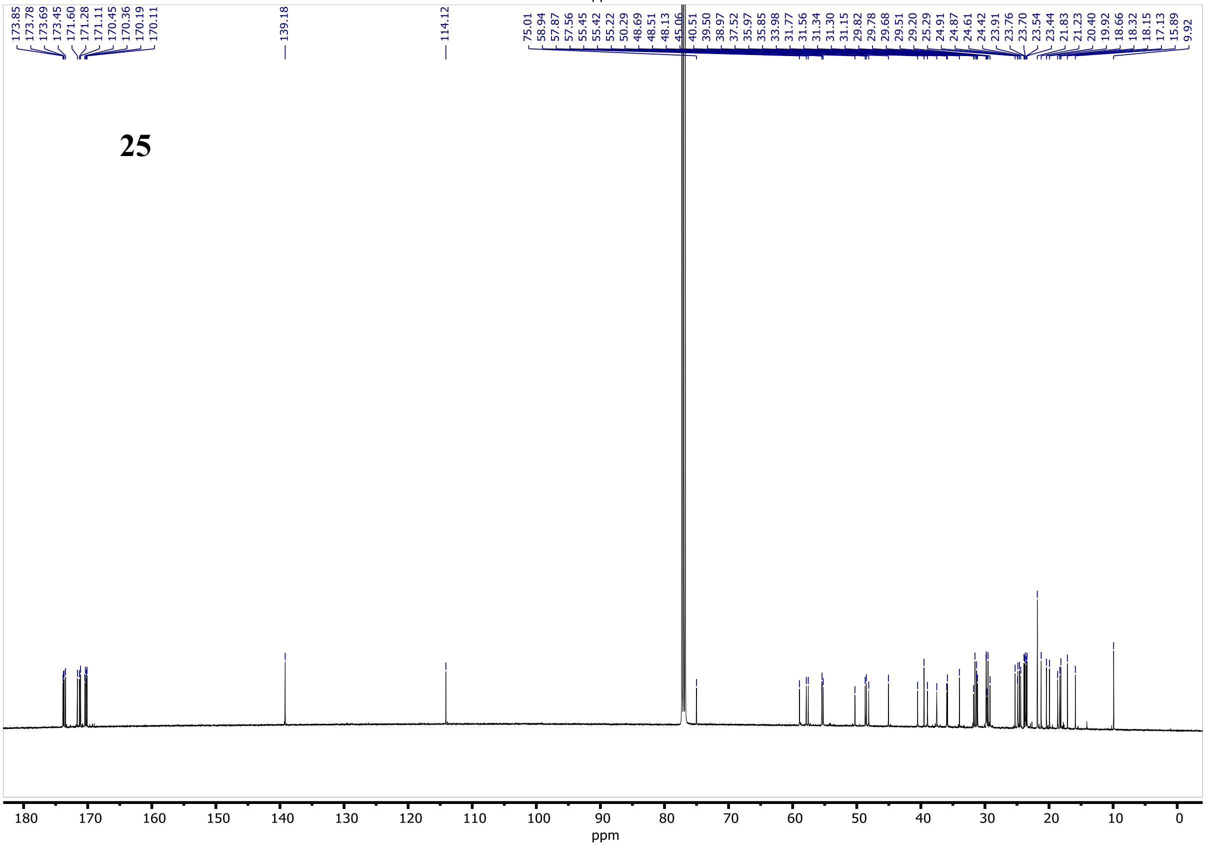

25

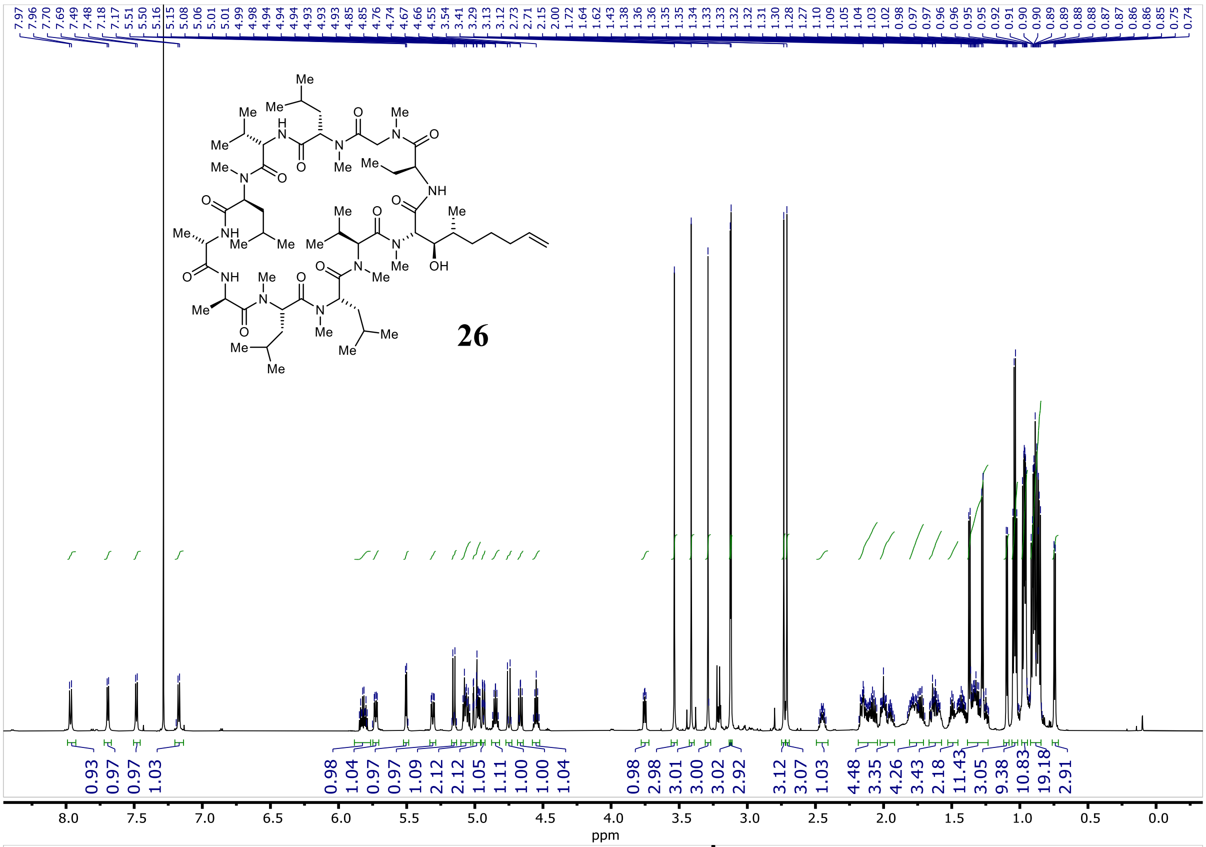
26
ppm

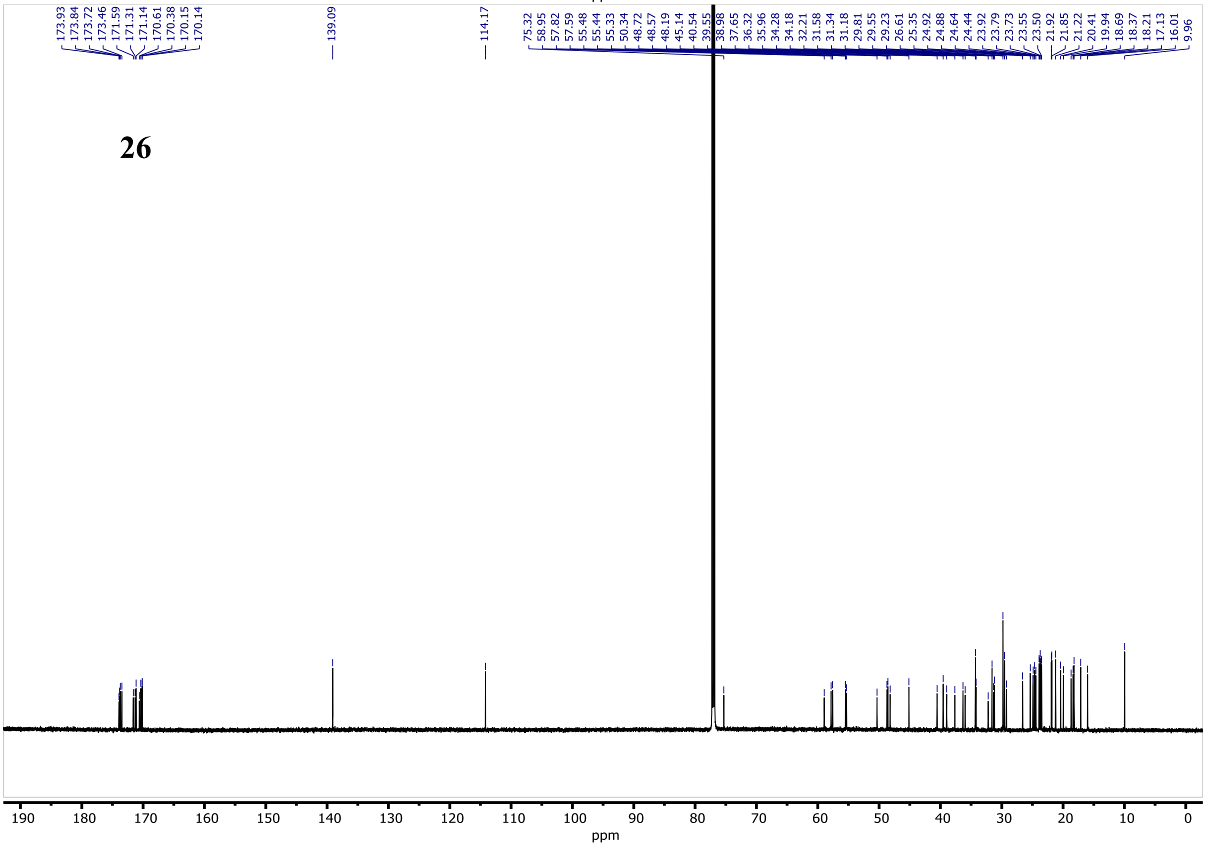
26
ppm

8. References

1. H.-S. Um, J. Min, T. An, J. Choi, C. Lee, *Org. Chem. Front.* **5**, 2158–2162 (2018).
2. J. Bruffaerts, I. Kesten, K. Buhnik-Rosenblau, A. Cohen, N. Edri, M. Cormier, Y. Zhang, G. Ho, I. Massad, H. Halfon-Verner, Y. Kashi, I. Marek, *Angew. Chem. Int. Ed*. **62**, e202306343 (2023).
3. N. S. Mahajani, J. D. Chisholm, *Org. Biomol. Chem.* **16**, 4008–4012 (2018).
4. S. M. West, J. E. Pia, S. A. L. Rousseaux, *Org. Lett.* ***24*** (32), 5869–5873 (2022).
5. A. Chrzanowska, D. Kurpios-Piec, B. Żyżyńska-Granica, E. Kiernozek-Kalińska, W. X. Lay, A. K. Ciechanowicz and M. Struga, *Eur. J. Pharmacol.* **940**, 175481 (2023).
6. A. M. Petros, J. R. Luly, H. Liang and S. W. Fesik, *J. Am. Chem. Soc.* **115**, 9920–9924 (1993).
7. P. M. Gannett, D. L. Nagel, P. J. Reilly, T. Lawson, J. Sharpe, B. Toth, *J. Org. Chem*. **53**, 1064–1071 (1988).
8. R. Gawin, A. Kozakiewicz, P. A. Guńka, Paweł Dąbrowski and Krzysztof Skowerski, *Angew. Chem., Int. Ed.*, **56**, 981–986 (2016).
9. V. M. Marx, M. B. Herbert, B. K. Keitz and R. H. Grubbs, *J. Am. Chem. Soc.*, **135**, 94–97 (2012).
10. Chemaxon, https://chemaxon.com/, (accessed 20 March 2025).
11. K. Sakai-Kato and K. Yoshida, *J. Pharm. Biomed. Anal.*, **180**, 113064 (2020).
